# Methods for Modeling Autocorrelation and Handling Missing Data in Mediation Analysis in Single Case Experimental Designs (SCEDs)

**DOI:** 10.1177/01632787211071136

**Published:** 2022-02-26

**Authors:** Emma Somer, Christian Gische, Milica Miočević

**Affiliations:** 1 Department of Psychology, 5620 McGill University, Montreal, QC, Canada; 2 Department of Psychology, 9373 Humboldt-Universitätzu Berlin, Berlin, Germany

**Keywords:** mediation analysis, single-case experiment designs, autocorrelation, missing data, small sample sizes

## Abstract

Single-Case Experimental Designs (SCEDs) are increasingly recognized as a valuable alternative to group designs. Mediation analysis is useful in SCEDs contexts because it informs researchers about the underlying mechanism through which an intervention influences the outcome. However, methods for conducting mediation analysis in SCEDs have only recently been proposed. Furthermore, repeated measures of a target behavior present the challenges of autocorrelation and missing data. This paper aims to extend methods for estimating indirect effects in piecewise regression analysis in SCEDs by (1) evaluating three methods for modeling autocorrelation, namely, Newey-West (NW) estimation, feasible generalized least squares (FGLS) estimation, and explicit modeling of an autoregressive structure of order one (AR(1)) in the error terms and (2) evaluating multiple imputation in the presence of data that are missing completely at random. FGLS and AR(1) outperformed NW and OLS estimation in terms of efficiency, Type I error rates, and coverage, while OLS was superior to the methods in terms of power for larger samples. The performance of all methods is consistent across 0% and 20% missing data conditions. 50% missing data led to unsatisfactory power and biased estimates. In light of these findings, we provide recommendations for applied researchers.

Single-Case Experiment Designs (SCEDs) are a valuable alternative to Randomized Controlled Trials (RCTs) that enable researchers to evaluate the effectiveness of an intervention at the individual level (Kazdin, 2011; Kratochwill et al., 2013; Shadish & Sullivan, 2011). The main goal of SCEDs is to determine whether there is a causal relationship between a treatment and change in the outcome variable of interest (Krasny-Pacini & Evans, 2018; Smith, 2012). For this aim, a small number of participants are repeatedly measured on variables of interest during baseline and intervention phases. Since participants serve as their own control, researchers can obtain detailed information related to changes over time, and intervention effects at the individual level can be estimated (Barlow et al., 2009).

SCEDs are used across various research fields, including occupational therapy, special education, and rehabilitation (Lane et al., 2017; Ritter et al., 2018; Smith, 2012). Given the heterogeneous nature of behavioral and psychological phenomena, SCEDs provide a valuable alternative to group level studies in populations with low incidence rates or in which analyses at a group level may overlook intervention effects present in certain subgroups (Gaynor & Harris, 2008; Maric et al., 2012). Further, the methodology is useful for evaluating a novel intervention prior to a costly and demanding RCT (Jarrett & Ollendick, 2012; Norell-Clarke et al., 2011). Finally, SCEDs present the opportunity for collaboration between clinicians and researchers, unifying research questions that emerge from clinical practice on one hand and research methodology to evaluate these questions on an individual level on the other hand (Geuke et al., 2019).

Examples of SCEDs include the AB design in which a baseline period A is followed by an intervention period B. In A_1_B_1_A_2_B_2_ designs, also known as a reversal design, the baseline phase (A_1_) is followed by the intervention phase (B_1_), the withdrawal of treatment (A_2_), and the re-introduction of the intervention (B_2_). This type of SCED is useful when changes in behavior caused by an intervention are expected to return to baseline levels once treatment is discontinued. Another common design includes the multiple-baseline design in which participants are randomized to different lengths of baseline phase A prior to introducing the intervention phase B, taking into account the effects of maturity and passage of time. For the interested reader, an extensive overview of SCEDs is provided by Tate et al. (2016) and Smith (2012).

Given the growing popularity of SCEDs as a rigorous scientific research approach, there have been efforts to establish empirical methods for evaluating the effectiveness of an intervention (Manolov & Moeyaert, 2017). Despite the prevalence of visual analysis, as described by Kratochwill et al. (2010), statistical analysis of SCED data is preferred since it is less prone to bias and subjectivity (Beeson & Robey, 2006). Efforts to empirically validate indices and effect sizes are of particular interest since it is useful to quantify the size of the intervention effect. Nevertheless, the index of choice depends on the aims of the study, and some indices may be better suited than others (Manolov & Moeyaert, 2017). Non-parametric non-overlap indices, such as non-overlap of all pairs (NAP; Parker & Vannest, 2009), improvement rate difference (IRD; Parker et al., 2009), Tau-U (Parker et al., 2011), and the percentage of non-overlapping corrected data (PNCD, Manolov & Solanas, 2009), are useful for measuring the degree of non-overlap between the baseline and treatment data. Descriptive indices, such as the percentage change index (PCI; Hershberger et al., 1999; or percentage reduction data, as referred to by Wendt, 2009), slope and level change (SLC; Solanas et al., 2010), and mean phase difference (MPD; Manolov & Solanas, 2013), quantify the change in level and slope. Parametric approaches are useful for quantifying the treatment effect size and estimating the standard error. Examples of parametric approaches are standardized mean differences (e.g., Cohen’s d, Hedge’s g; Shadish et al., 2014), regression-based effect sizes (Center et al., 1985; Swaminathan et al., 2014), multilevel modeling (Ferron et al., 2010; Moeyaert et al., 2014), and between-case standardized difference (Hedges et al., 2012, 2013).

The approach examined in this paper relies on regression-based methods, first proposed by Center et al. (1985). Piecewise regression procedures involve fitting separate models for each phase, baseline and intervention, using ordinary least squares regression (OLS). Given that the assumptions of OLS regression hold, such as the assumption that the outcome is continuous, the residuals are homoscedastic and uncorrelated, and the residuals are normally distributed with means of zero in the population, we can obtain unbiased estimates of at least two regression-based effect sizes: an immediate intervention effect (i.e., immediate change in level) and the intervention effect on the time trend. Using an AB design to illustrate the technique (Equation (1)), the intercept, $\;b_{0}$ of the piecewise regression model provides an estimate of the level of the first measurement of the outcome variable in phase A. The regression coefficients provide an estimate for the trend in phase A (i.e., the regression coefficient for the linear time variable, $b_{1}$ ), the change in level at the onset of phase B (i.e., the difference between the intercept of phase B and the predicted score if this were a score in phase A, $b_{2}$ ), and the change in the trend between the phases (i.e., the difference in linear trends between the A and B phases, $b_{3}$ ). The following equation describes the observed score (*Y*_*t*_) at time t

(1) $$Y_{t}\;=b_{0}\;+b_{1}\;{time}_{t}\;+b_{2}\;{phase}_{t}\;+b_{3}\;{phase\_time}_{t}\;+e_{t}$$

Despite the advantages of SCEDs mentioned above, there are several methodological challenges that may prevent the proliferation of SCED methodology in clinical intervention research. A common characteristic of SCEDs is serial dependency among error terms, commonly referred to as autocorrelation. Autocorrelation among consecutive error terms can be modeled via autoregressive (AR) processes, for example, an AR process of order one (AR(1). Autocorrelation is quantified by the parameter rho (*ρ*), which ranges from −1 to 1 (see Equations (7) and (8) in the section on Autocorrelation Modeling Techniques). Violating the assumption of independence of errors as required by most parametric and non-parametric approaches can result in highly inefficient estimates and inflated Type I error rates (Huitema et al., 1996). Another common attribute of designs involving repeated measures is missing data. Failing to properly account for missing data limits the generalizability of the results, threatens internal validity, and can lead to biased and inefficient estimates (Hughes et al., 2019; Little & Rubin, 2002; Peng & Chen, 2018; Rubin, 1987).

Most statistical methods for SCEDs were developed for evaluating univariate (e.g., autoregressive integrated moving averages (ARIMA) models; Box & Jenkins, 1970) and bivariate relationships (e.g., simulation modeling analysis; Borckardt et al., 2008; standardized mean difference; Borenstein, 2009; percentage of non-overlapping data; Schlosser et al., 2008). However, the approaches mentioned thus far do not provide a method for examining the mechanism through which the intervention achieves its effects for a particular client. Recent advances in statistical methods for SCEDs have focused on adapting methods for mediation analysis to the SCEDs setting, and at least three methods have been proposed (Gaynor & Harris, 2008; Geuke et al., 2019; Miočević et al., 2020). While Gaynor and Harris (2008) relied on visual analysis to assess mediation, Geuke et al. (2019) presented the joint significance test to evaluate the significance of the indirect effect. Miočević et al. (2020) were the first to introduce a method for obtaining numerical estimates and credibility intervals for the indirect effect in SCEDs.

This paper aims to examine parameter estimation in a piecewise regression model by evaluating the statistical properties of the indirect effect (1) using three different methods for handling autocorrelation in repeated measures data and (2) examining the performance of multiple imputation in the presence of data that are missing completely at random (MCAR; Rubin, 1976). The following sections describe mediation analysis for a single mediator model, and we provide more details about the methods for estimating indirect effects in piecewise regression analysis proposed by Miočević and coauthors (2020).

## Single Mediator Model

Mediation analysis is used to evaluate whether a variable acts as a mediator (*M*) transmitting the effect from the independent variable (*X*) to a dependent variable (*Y*). The effects of interest in the single mediator model can be estimated using three regression equations.

(2) $$Y=i_{1}\;+\;cX\;+\;e_{1}$$

(3) $$Y=\;i_{2}\;+c'X\;+bM\;+\;e_{2}$$

(4) $$M=i_{3}+aX\;+\;e_{3}$$

In Equations (2)–(4), *c* represents the total effect of *X* on *Y*, *c’* is the effect of *X* on *Y* adjusted for the effect of the mediator *M* (also called the direct effect), *b* quantifies the relation between the mediator (*M*) and the dependent variable (*Y*) controlling for the effect of the independent variable (*X*), and *a* captures the relationship between *X* and *M*. The terms *i*_*1*_, *i*_*2*_, and *i*_*3*_ represent intercepts, and it is assumed that the three error terms, *e*_*1*_, *e*_*2*_, and *e*_*3*_, are uncorrelated and follow normal distributions with means of zero and variances $\sigma_{e1}^{2}$ , $\sigma_{e2}^{2}$ , and $\sigma_{e3}^{2}$ (respectively).

The indirect effect can be computed as either the product of coefficients *ab* or as the difference of coefficients *c-c’*, and the two approaches are equivalent in linear models with no missing values (Mackinnon et al., 1995). The significance of the indirect effect is generally evaluated using confidence or credibility intervals. Due to the asymmetry in the distribution of the product of two normal variates (i.e., *ab*; Craig, 1936; Lomnicki, 1967; Springer & Thompson, 1966), methods that use critical values from the distribution of the product and methods that make no distributional assumptions like the bootstrap lead to confidence intervals with the highest power (MacKinnon et al., 2007; MacKinnon et al., 2002; Miočević et al., 2017; Tofighi & MacKinnon, 2011; Yuan & MacKinnon, 2009). Subsequent sections describe methods for estimating indirect effects using piecewise regression analysis in combination with several techniques to model serially correlated error terms.

## Estimating Indirect Effects in SCEDs using Piecewise Regression Analysis

In the single mediator model for SCEDs, both the mediator and outcome variables are repeatedly measured across at least two phases (i.e., the baseline phase and the intervention phase). We selected piecewise regression analysis because it allows for quantifying the change in the mediator as a result of the change in phase (*a* in Equation (4)) and change in outcome as a result of the change in the mediator (*b* in Equation (3)) controlling for the effect of phase.

Effects of interest for a single mediator model using piecewise regression analysis can be estimated using two equations (Miočević et al., 2020).^1^

(5) $$M_{t}\;=b_{0M}\;+b_{1M}\;{time}_{t}\;+b_{2M}\;{phase}_{t}\;+b_{3M}+{phase\_time}_{t}\;+e_{M,t}$$

(6) $$Y_{t}=b_{0Y}\;+b_{1Y}\;{time}_{t}\;+b_{2Y}\;{phase}_{t}\;+b_{3Y}\;{phase\_time}_{t}\;+b_{4Y}M_{t}\;+e_{Y,t}$$

As a result of the specific coding of the predictors, regression coefficients from the piecewise regression analysis provide estimates of the level of the first time point of phase A for the mediator (*b*_*0M*_) and for the outcome (*b*_*0*__*Y*_), the trend in phase A for the mediator (*b*_*1M*_) and for the outcome (*b*_*1Y*_), the change in level at the start of phase B for the mediator (*b*_*2M*_) and for the outcome (*b*_*2Y*_), and the change in trend between the two phases for the mediator (*b*_*3M*_) and for the outcome (*b*_*3Y*_). We also obtain an estimate for the effect of the mediator on the outcome at time t (*b*_*4Y*_). In their paper, Miočević and colleagues (2020) estimated the two equations in the Bayesian framework using OLS estimates of the regression coefficients as mean hyperparameters of the normal priors for intercepts and regression coefficients in Equations (5) and (6). In this paper, we opt for frequentist estimation instead.

There are two effects of phase on the mediator (*a* path in Equation (4)) in this context: the change in level (*b*_*2M*_) and the change in trend (*b*_*3M*_) between the two phases. If we define the *a* path as the change in level between the two phases, the indirect effect (the product *ab*; see Equations (3) and (4) in the section on the Single Mediator Model) of the phase change on the outcome can be quantified through the change in the level of the mediator. If we define the *a* path as the change in trend between the two phases, the indirect effect of the phase change on the outcome can be quantified through the change in the trend of the mediator. The effect of the mediator on the outcome (*b* in Equation (3)) is represented by *b*_*4Y*_ in Equation (6), and the direct effects (*c’* in Equation (3)) of phase on the outcome controlling for the mediated effect is decomposed into *b*_*2Y*_ (for the changes in level) and *b*_*3Y*_ (for the changes trend).

There are two indirect effects of interest in the piecewise regression model for SCEDs: (1) the product of coefficients *b*_*2M*_* b*_*4Y*_, representing the change in outcome variable due to the change in the level of the mediator following a change in phase, and (2) the product of coefficients *b*_*3M*_* b*_*4Y*_, representing the change in the outcome variable due to the change in the trend (slope) of the mediator following a change in phase.

## Autocorrelation Modeling Techniques

In their review of 809 single-case designs, Shadish and Sullivan (2011) found that autocorrelation ranged from −0.931 to 0.736, and they noted that autocorrelation (*ρ*) is commonly underestimated when the number of observations is small. Using a procedure to correct for negative bias, the authors found that the mean value of autocorrelation in SCEDs was estimated to equal 0.752 in the seven AB designs evaluated and 0.320 in the 64 multiple-baseline designs examined (Shadish & Sullivan, 2011). In subsequent paragraphs, we describe procedures for modeling serial dependency among error terms.

In our first approach, we estimate the regression coefficients via ordinary least squares (OLS) regression. OLS regression is a consistent estimator even in the presence of serially correlated error terms; however, OLS is no longer the best linear unbiased estimator and does not yield correct standard errors in the presence of serially correlated error terms (Davidson & MacKinnon, 2003). Therefore, we use heteroskedasticity- and autocorrelation-consistent standard errors as proposed by Newey and West (Newey & West, 1987). In this approach, the exact form of serial correlation in the error terms does not need to be specified, and the procedure also allows for heteroscedasticity. Newey-West standard errors are available in most standard software packages, for example, in the sandwich package in R (Zeileis, 2004).

In our second approach, we explicitly model the serial correlation in the error terms. For this purpose, we assume that the error terms follow an AR(1) process. Thus, we add the following equations to Equations (5) and (6)

(7) $$\;e_{M,t}=\rho_{M}e_{M,t-1}+\nu_{M,t}$$

(8) $$\;e_{Y,t}=\rho_{Y}e_{Y,t-1}+\nu_{Y,t}$$

The autocorrelation coefficients $\rho_{M}$ and $\rho_{Y}$ quantify the strength of the serial dependency and are assumed to range between −1 and +1. The error terms $\nu_{M,t}$ and $\nu_{Y,t}$ in Equations (7) and (8) are assumed to be white noise and mutually independent. This class of models is well understood (Cochrane & Orcutt, 1949; Prais & Winsten, 1954) and can be estimated using generalized least squares (GLS) estimation. The GLS estimator is the best linear unbiased estimator (Davidson & MacKinnon, 2003). However, in practice, the true population values $\rho_{M}$ and $\rho_{Y}$ of the autocorrelation coefficients are unknown and need to be estimated from the data along with the regression coefficients. This procedure is known as feasible GLS (FGLS) and yields a non-linear estimator that is no longer unbiased. Furthermore, the small sample properties of FGLS are not known analytically. However, feasible GLS is asymptotically efficient (Davidson & MacKinnon, 2003). FGLS is implemented in several software packages, for example, in the orcutt package in R (Stefano et al., 2018). Note that the computation of the FGLS estimator in the above setting can be implemented using the so-called iterative Cochrane-Orcutt procedure (Stefano et al., 2018) which tends to outperform alternative two-step procedures for computing FGLS in small samples (Verbeek, 2017).

Our third approach is based on the same regression Equations (5) and (6) combined with the AR(1) Equations (7) and (8) for the error terms. In other words, we make the same modeling assumptions as in the case of FGLS. However, we use a different estimation technique, where the exact likelihood is computed via a state-space representation of the AR(1) process, and estimates are computed by a Kalman filter. This procedure is implemented in the stats package in R (R Core Team, 2020). The advantage of this procedure over FGLS lies in the possibility of including more complex patterns of serial correlation in the error term equations (e.g., moving average components, non-stationary integrated error terms). Throughout this paper, we focus on AR(1) error terms and thus expect that the results will be similar to those obtained by FGLS. We refer to the three approaches described above as NW, FGLS, and AR(1) throughout the remainder of the paper. We compare these three approaches to a standard OLS procedure that ignores the presence of autocorrelation.

### Missing Data Handling Techniques

Missing data in SCEDs is common due to repeated measures of participants over time, resulting in noncompliance and participant attrition (Smith, 2012). There are three ways to categorize missing data: missing completely at random (MCAR), missing at random (MAR), and missing not at random (MNAR). MCAR is characterized by missing data that does not depend on observed data nor on the missing data, for example, when a random subset of participants’ self-report data is lost. MAR, on the other hand, is a function of the observed data but not a function of the missing data. In a study evaluating confidence among university-aged men and women, for example, women may feel uncomfortable when asked to rate their appearance and choose not to answer questions related to physical appearance. In this case, the participant’s gender results in nonresponse. Finally, MNAR occurs when the missingness is related to the unobserved data. When individuals with the lowest education are missing from a study evaluating educational outcomes, the missing data mechanism is MNAR. Improper handling of missing data using traditional methods such as listwise deletion and mean substitution can lead to loss of information, biased estimates, inefficiency, and introduce effects that are not supported by data (Little & Rubin, 2002). Modern approaches to handling missing data such as the expectation-maximization (EM) algorithm (developed by Dempster et al., 1977) and multiple imputation (MI) (Schafer & Graham, 2002) have gained favor over more traditional methods. Numerous studies have advocated for using maximum likelihood and EM methods for handling missing data in group multivariate designs (e.g., Horton & Kleinman, 2007; Ibrahim et al., 2005; Raghunathan, 2004). Velicer and Colby (2005a, 2005b) found that maximum likelihood is an effective strategy for handling missing data compared to listwise deletion, mean substitution, and mean of adjacent observations in time series data. In the subsequent paragraph, we describe MI in greater detail.

We examine MI proposed by Rubin (1987) for data that are MCAR in a simulation study. An imputation refers to one set of plausible values (*m*) for a missing observation, while MI represents multiple sets of plausible values (*m* > 1). When using MI, the missing value is replaced by a random sample of plausible values resulting in *m* complete datasets. The statistical analysis of interest (e.g., OLS regression) is then conducted on each *m* complete dataset separately. Finally, a single MI estimate and its standard error (SE) are estimated by combining results obtained from each *m* analysis using Rubin’s rules (Rubin, 1987). Suppose $\hat{Q}$ represents the estimate of a parameter *Q* (e.g., a regression coefficient) from the j^th^ imputed data set. The pooled estimate is given by Equation (9).

(9) $$\bar{Q}=m^{-1}\sum_{j=1}^{m}{\hat{Q}}_{j}$$

The total variance of $\bar{Q}$ , represented by *T* in Equation (12), is the weighted sum of the average within-imputation variance $\bar{U}\;$ (Equation (10)) and the between-imputation variance B (Equation (11)). The overall SE of $\bar{Q}$ is equal to the square root of *T*.

(10) $$\bar{U}=m^{-1}\sum_{j=1}^{m}{\hat{U}}_{j}$$

(11) $$B={\left(m-1\right)}^{-1}\sum_{j=1}^{m}{\left[{\hat{Q}}_{j}-\;\bar{Q}\right]}^{2}\;\;$$

(12) $$T=\bar{U}\;+\;{\left(m+1\right)}^{-1}B\;\;$$

There are two methods for conducting multivariate MI in which values are missing on multiple variables: multivariate normal imputation (MVNI) and MI by chained equations (MICE). MVNI assumes that the incomplete variables follow a multivariate normal distribution (Lee & Carlin, 2010). MICE generates separate univariate imputation models for each variable with missing data (White et al., 2011). In the present study, we evaluate MICE as a missing data handling method in order to examine how one of the most commonly used R packages for handling missing data performs when adapted to piecewise regression analysis for SCEDs (van Buuren & Groothuis-Oudshoorn, 2011). To our knowledge, this is the first study that analyzes missing data in SCEDs in the presence of autocorrelated errors. Therefore, the aim of our simulation study is to examine what would happen if researchers just continued with their standard practice in the presence of autocorrelated errors.

### Missing Data Handling in SCEDs

A review of missing data in SCED studies published by Chen et al. (2020) indicated that approximately 18% of studies (33 out of 182) contained missing data with a range of 1%–45% missing values. In general, studies reported a higher average percentage of missing values in the intervention phase (15%) compared to the baseline phase (6%) (Chen et al., 2020). Previous studies have investigated the performance of various missing data handling techniques in SCEDs (Smith et al., 2012; Peng & Chen, 2018; Chen et al., 2020; De, Michiel, Tanious, & Onghena, 2020). In a Monte Carlo simulation study, Smith et al. (2012) evaluated the performance of the EM procedure in terms of statistical power for data simulated as MCAR. Effect sizes were quantified using the standardized mean difference (Glass’s Δ). They concluded that EM is effective at handling missing data across various levels of missingness (10%, 20%, 30%, and 40%) and lag-1 autocorrelation (0, 0.2, 0.4, 0.6), except when autocorrelation is large (i.e., 0.8). Peng and Chen (2018) applied MI to missing data from a published single-case ABAB design and examined effect sizes using Tau-U. They concluded that there are several advantages to MI over ad hoc methods such as mean substitution in that it avoids potential bias that can arise from omitting participants from an analysis, takes into account the uncertainty surrounding the imputed scores, and retains the design structure of the study. Chen et al. (2020) extended the findings from Smith et al. (2012) by examining the performance of EM in terms of relative bias (RB), root-mean squared error (RMSE), and relative bias of the estimated standard error (RBESE). They estimated the baseline slope, level shift, and slope change from a piecewise regression model for data simulated under a MAR mechanism for an AB design. The authors concluded that EM is an effective strategy for missing data handling in piecewise regression analysis for SCEDs. De et al. (2020) assessed the performance of three missing data handling methods for data simulated under MCAR: (1) randomized-marker method, (2) single imputation (SI) using an autoregressive integrated moving average (ARIMA) model, and (3) MI using multivariate imputation by chained equations (MICE). De et al. (2020) computed the mean difference (MD) and nonoverlap of all pairs (NAD) as their indicators of an intervention effect. The authors concluded that the randomized-marker method is a promising missing data handling technique as it outperformed the other methods in terms of statistical power while ensuring a low Type I error rate. Only one study (i.e., Chen et al., 2020) examined the performance of missing data handling methods for piecewise regression analysis in SCEDs. In this study, we aim to determine how MI performs for piecewise regression in SCEDs when data is MCAR. We simulated data under MCAR to reflect one possible scenario in practice, namely that missing data are due to the participant not filling out the questionnaire for a given measurement occasion due to a random event that prevented them from providing data for that observation. This results in complete data on the variables time, phase, phase_time in Equations (5) and (6) because those are part of the study design, whereas if the participant fails to complete the questionnaire, data are missing on the mediator and outcome at the same measurement occasion.

## Methods

### Empirical Example

To illustrate the three approaches to modeling autocorrelation described above and compare them to OLS estimation, we apply the methods to an example data set from an AB SCEDs study. The study evaluated the effectiveness of a walking intervention for osteoarthritis in four individuals (O’Brien et al., 2016). Over 12 weeks, diary measures were taken twice daily on symptoms related to impairment (i.e., pain, pain on movement, and joint stiffness), cognitions (i.e., intentions, self-efficacy, and perceived controllability), and walking behavior (i.e., number of steps). The goal of the study was to evaluate the role of cognition in predicting outcomes in individually-tailored walking interventions for osteoarthritis. Cognitions, such as intention and self-efficacy, were hypothesized to transmit the effect of impairment on physical activity. The baseline phase was designed to obtain baseline measures and identify the cognitions that impacted walking activity in the participants. During the intervention phase, participants received an intervention that targeted the cognitions that were shown to strongly correlate with walking behavior.

We illustrate the proposed methods using data from a single participant, participant A. The number of observations was relatively even across phases, with 81 measurement occasions in the baseline phase and 89 measurement occasions in the intervention phase. For the single mediator model, the mediated effect of phase (*X*) on walking (*Y*), measured as the step count, through the intention to walk (*M*) was considered. The percentage of missing data in the study was equal to 2.37% and 3.55% for *M* and *Y*, respectively. Missing values on *M* and *Y* were imputed using the R package MICE (van Buuren & Groothuis-Oudshoorn, 2011). Next, an AR(1) model was fit to the residuals to inspect the autocorrelation. Estimates for the serial correlation among the residuals were −0.54 and −0.81 for *M* and *Y*, respectively. Parameter estimates were obtained using piecewise regression analysis, and autocorrelation was modeled according to the three proposed approaches which were compared to OLS. The analyses were conducted in R (R Core Team, 2020) using the package RMediation to compute the Monte Carlo confidence intervals (CI) for the mediated effect (Tofighi & MacKinnon, 2011).^2^ The annotated R syntax for the analyses is provided in the Supplemental Materials available at https://osf.io/ahpkm/?view_only=d9144ffbc4bc4af28df31b03164ed6b2.

Point and interval estimates are displayed in Table 1. The interval estimates for the indirect effect through the change in level for the single mediator model were consistent across methods with the significance test indicating that the 95% CIs contained zero. The interval estimates for the indirect effect through the change in trend for the four approaches to handling autocorrelation also led to the same conclusion about the significance test in that the 95% CIs contained zero. Nevertheless, there were noticeable differences in the point and interval estimates across methods. The point estimate for the indirect effect through changes in level and trend were closer to zero for FGLS and AR(1) than OLS and NW. The interval widths for both the indirect effect through changes in level and trend were substantially smaller for FGLS and AR(1) than for OLS and NW, indicating more precision in the estimates. The standard error of the point estimate for the indirect effect for changes in level and trend was largest for OLS, followed by NW. AR(1) and FGLS had smaller standard errors than both OLS and NW.

**Table 1.** Results from the Single Mediator Model Empirical Example.

	Autocorrelation Handling Method			
Parameter Estimate	OLS	NW	FGLS	AR(1)
*b*_2M_ b_*4Y*_	−311.78	−311.78	56.57	56.96
b_3M_ b_*4Y*_	−11.73	−11.73	1.82	1.83
95% CI for *b*_*2M*_* b*_*4Y*_	[−1320.40, 676.90]	[−901.03, 269.01]	[−40.21, 212.22]	[−38.72, 214.61]
95% CI for *b*_*3M*_* b*_*4Y*_	[−32.75, 8.74]	[−24.63, 0.89]	[−0.51, 5.63]	[−0.59, 5.74]
SE of *b*_*2M*_* b*_*4Y*_	507.50	297.79	64.37	64.86
SE of *b*_*3M*_* b*_*4Y*_	10.54	6.49	1.60	1.64

*Note.* Point and interval estimates of the indirect effect through the change in level and change in trend are displayed for OLS estimation and three methods for handling autocorrelation. *b*_2M_ *b*_*4Y*_ represents the indirect effect through the change in level. *b*_3M_ *b*_*4Y*_ represents the indirect effect through the change in trend. CI = confidence interval. SE = standard error.

### Simulation Studies

Simulation studies were performed to investigate the number of time points required to attain acceptable statistical properties for point and interval estimates of the indirect effect in a single mediator model. We assessed the bias, relative bias, efficiency (the standard deviation of the point estimate across replications), power, Type I error rate, coverage, and interval width. Bias and relative bias were used to assess the accuracy of the point estimate of the indirect effect. Since bias is affected by the size of the indirect effect, relative bias is the preferred measure of accuracy. Relative bias is computed as the difference between the value of the indirect effect in the population and the estimate of the indirect effect divided by the value of the indirect effect in the population. When the true indirect effect is zero, relative bias is undefined. Values of relative bias between −0.10 and 0.10 were considered acceptable (Kaplan, 1988). The standard deviation of the estimate of the indirect effect over replications was a measure of efficiency, where higher standard deviation values indicated lower efficiency. Power was defined as the proportion of confidence intervals for the indirect effect that excluded zero when the indirect effect is nonzero. Values of 0.8 and higher were considered desirable. Type I error rate was computed as the proportion of confidence intervals that excluded zero when the true value of the indirect effect was zero. A Type I error rate of 0.05 was deemed desirable, and values between 0.025 and 0.075 were acceptable (Bradley, 1978). Coverage was defined as the percentage of confidence intervals that contained the true value of the indirect effect, and values of coverage between 0.925 and 0.975 were considered close to the nominal level of 0.95 according to Bradley’s robustness criterion (1978). Interval width was defined as the difference between the upper confidence limit and the lower confidence limit. Lower interval widths represent higher precision. The R code for the simulation studies is available in the Supplemental Materials.

### Single Mediator Model: No Missing Data

A total of 1000 replications were simulated from piecewise regression models using Equations (5) and (6). *M* and *Y* were simulated as continuous variables from an AB design. Ferron et al. (2010) reported that the median number of observations in SCEDs was 24 with a range of seven to 58. To simulate realistic conditions, we evaluated sample sizes (N) of 20 and 30. We also chose to consider sample sizes larger (*N* = 60 and 100) than those typically observed in SCEDs. Although the baseline phase is typically shorter than the treatment phase in SCEDs (Shadish & Sullivan, 2011), we opted for an equal number of observations in the baseline and treatment phases to isolate the effect of autocorrelation on the performance of our methods. When *N* = 60, for example, we assigned 30 observations to the baseline phase and 30 observations to the intervention phase. Values of 0 and 2 were simulated for the *a* path defined as the change in level (*b*_*2M*_; a_level), and values of 0 and 0.2 were simulated for the *a* path defined as the change in trend between the two phases (*b*_*3M*_; a_trend). The nonzero effect sizes for the *a* paths come from the conventions in the simulation literature on SCEDs (the a_level of 2 and a_trend of 0.2 were used in, e.g., Moeyaert et al., (2013a) and Moeyaert et al., (2013b)). Values of 0 and 0.59 were chosen for the *b* path (*b*_*4Y*_). The nonzero *b* path value stems from the conventions in the simulation literature on mediation models (the value of 0.59 is considered as a large value for the *b* path in the single mediator model in, e.g., Fritz & MacKinnon (2007); MacKinnon et al,. (2004), and Wang and Preacher (2015)). We included effect sizes of 0 for the *a* and *b* paths to evaluate the Type I error rates of the methods.^3^ Four different values of *ρ* (0, 0.1, 0.5, and 0.9) were simulated, and we analyzed the simulated data using four methods: OLS, NW, FGLS, and AR(1). Statistical properties were evaluated for 128 (4 × 2 × 2 × 2 × 4) combinations of parameters.

The simulation study was carried out in R (R Core Team, 2020). To generate data for *M* and *Y*, first, a deterministic portion of *M* and *Y* was simulated based on parameter values for a given condition. Four levels of autocorrelation (*ρ*) were simulated (no AR, AR.small, AR.medium, AR.large). In the no AR(1) condition, normal residuals with means of 0 and standard deviations of 1 were added to the deterministic portion using the rnorm command. In the conditions where low, medium, and large autoregressive effects were evaluated, residuals were added to the deterministic function using the arima.sim() function based on the specified value of *ρ* (0.1, 0.5, or 0.9). For OLS estimation, estimates of the parameters in Equations (5) and (6) were obtained using the lm() function. Using NW standard errors, regression coefficients were identical to those obtained from OLS estimation. The vcovHAC() function in the R package sandwich (Zeileis, 2004) was used to estimate a HAC covariance matrix, and coeftest() in the R package lmtest (Zeileis & Hothorn, 2002) was used to obtain the estimates for the HAC standard errors. The coefficients and standard errors for FGLS estimation were obtained using the cochrane.orcutt() function in the orcutt package in R (Stefano et al., 2018). Finally, in order to fit an AR(1) model, the data were transformed into a time series object using ts(). The arima() function was used to fit a model to the time series data with an autoregressive structure of order one. Point estimates of the indirect effect *ab* using OLS and our three autocorrelation handling methods were computed through the change in level and the change in trend. The RMediation package (Tofighi & MacKinnon, 2011) was used to compute 95% confidence intervals using the Monte Carlo method with the medci() function (Mackinnon et al., 2004). Finally, statistical properties of the point and interval estimates were computed as described in the previous paragraph for each iteration of the simulation study.

### Single Mediator Model: Missing Data

The simulation for the single mediator model with missing data on *M* and *Y* was performed in a similar fashion to the simulation for the single mediator model without missing data. We considered the same parameter values for *b*_*2M*_, *b*_*3M*_, and *b*_*4Y*_, sample sizes, autoregressive values, and autoregressive handling methods as in the single mediator model without missing data. Two proportions of missing data (20% and 50%) were simulated under an MCAR condition. Statistical properties were evaluated for 256 (4 × 2 × 2 × 2 × 4 × 2) combinations of parameters.

The simulation study was carried out in R (R Core Team, 2020). The first step in data generation for *M* and *Y* was the same as in the complete case scenario. Following the simulation of *N* values of *M* and *Y* with a specific value of *ρ* (0, 0.1, 0.5, or 0.9), we used the ampute() function from the R package MICE (van Buuren & Groothuis-Oudshoorn, 2011) to remove a specified proportion of observations (20% or 50%) in both *M* and *Y* under MCAR. The mice() function was then used to impute missing values on *M* and *Y*. Following the recommendation of White et al. (2011), who proposed that the number of imputations should be at least equal to the proportion of missing data (e.g., 30% missing data requires at least 30 imputations), we requested 100 imputations, and we chose five iterations according to the recommendation of van Buuren and Groothuis-Oudshoorn (2011). OLS regression was conducted on the 100 complete data sets using the with() function, and the estimates for the regression coefficients were combined into one estimate using the pool() function. Estimates for the NW standard errors, as well as the FGLS and AR(1) estimates, were obtained using the same procedures as in the simulation study with complete cases for each imputed data set, followed by the pooling together of the estimates using the pool() function. Point and interval estimates of the indirect effect *ab* were computed following the same procedures as in the simulation study with complete cases. Finally, statistical properties of the point and interval estimates were computed for each iteration of the simulation study.

## Results

### Single Mediator Model: No Missing Data

#### Bias and Efficiency

Through the change in level, the point estimates for the indirect effect were unbiased (Figure S1 found in Supplemental Materials). The range of relative bias generally increased as the autoregressive effect increased for all autocorrelation handling methods. When *N* = 60, the four methods performed comparably in terms of relative bias across all levels of autocorrelation and parameter combinations. When *N* = 20, 30, and 100 and *ρ* = 0.9, the mean relative bias over 1000 replications was greater for OLS and NW than FGLS and AR(1). FGLS followed by AR(1) resulted in the lowest mean relative bias when the amount of simulated autocorrelation was high. In terms of efficiency, at all values of *N*, the methods performed similarly for *ρ* = 0, 0.1, and 0.5. When *ρ* = 0.9, the standard deviation across replications was greater for OLS and NW than FGLS and AR(1) which performed similarly. As the sample size increased, the standard deviation over replications generally decreased for *ρ* = 0, 0.1, and 0.5, and this effect was most noticeable for nonzero *b* paths (Figure S2A). The mean standard deviation increased as the sample size increased when *ρ* = 0.9 for OLS and NW.

The estimates of the indirect effect through changes in trend were unbiased in the majority of conditions. However, when *N* = 20 and *ρ* = 0.9, the relative bias across replications was greater than 0.10 for all autocorrelation handling methods, where OLS and NW had a higher mean relative bias than AR(1) and FGLS (Figure S1). The range of relative bias generally increased as the autocorrelation increased for all methods. As the sample size increased, the standard deviation of the point estimate decreased across all autocorrelation handling techniques, and this effect was most noticeable at *ρ* = 0.9 for nonzero *b* paths (Figure S2). The methods performed similarly for *ρ* = 0, 0.1, and 0.5 in terms of efficiency. At *ρ* = 0.9, AR(1) methods had lower standard deviation values than FGLS, NW, and OLS in most conditions, although the differences between methods were less pronounced than through changes in level. Lower values of standard deviation for AR(1) were most noticeable when *b* = 0.

### Power

**Figure 1.**
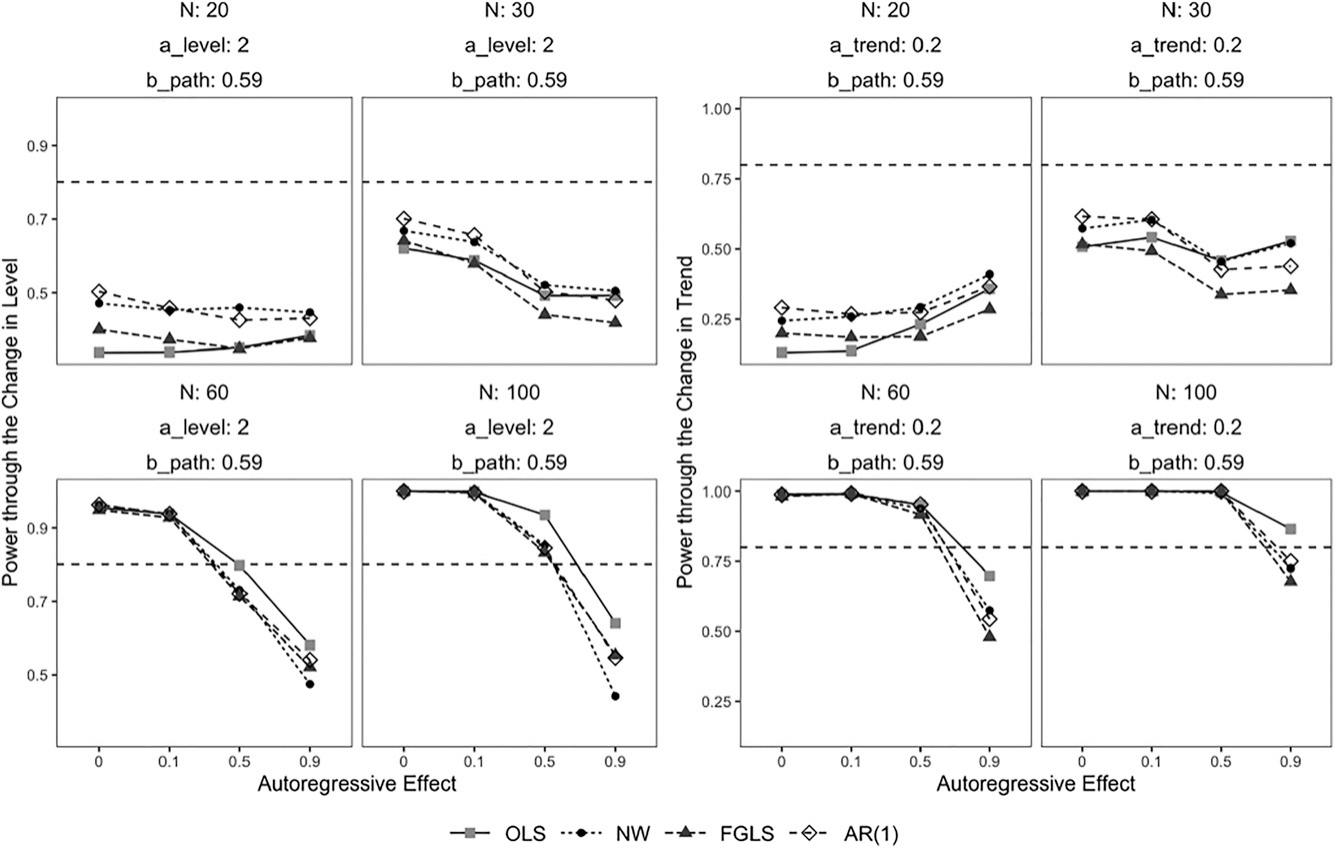
Power of the Interval Estimate of the Indirect Effect through the Change in Level and Trend *Note.* Power of the interval estimate of the indirect effect defined through the change in level and trend over 1000 replications. The dotted line represents power of 0.8. Different values for the *a* path defined as the change in trend did not impact the power for the change in level, and different values for the *a* path defined as the change in level did not impact the power for the change in trend. a_level = *a* path as the change in level. a_trend = *a* path as the change in trend. b_path = *b* path. N = sample size.

Through the change in level, power increased as the sample size increased for most parameter combinations (Figure 1). When *N* = 20 and 30, power was below 0.8 for all autocorrelation handling methods and at all levels of simulated autocorrelation. When *N* = 60 and *N* = 100 and *ρ* = 0 and 0.1, power exceeded the nominal value of 0.8. When *N* = 60 and *ρ* = 0.5, power was equal to 0.8 for OLS and below 0.8 for NW, FGLS, and AR(1). Power was unacceptable for all methods when *ρ* = 0.9 and *N* = 60 and 100. OLS had the highest values of power when *ρ* = 0.9 at large sample sizes, followed by FGLS and AR(1) which performed comparably. NW yielded the lowest power.

Through the change in trend, power increased as the sample size increased. Power was below 0.8 for small sample sizes (i.e., *N* = 20 and 30) (Figure 1). When *N* = 60 and 100 and *ρ* = 0, 0.1, and 0.5, power was above 0.8. When *ρ* = 0.9 and *N* = 60 and 100, power was below 0.8 for NW, FGLS, and AR(1). OLS yielded acceptable power at *N* = 100. FGLS had the lowest power of all the methods at *ρ* = 0.9.

### Type I Error Rate

**Figure 2.**
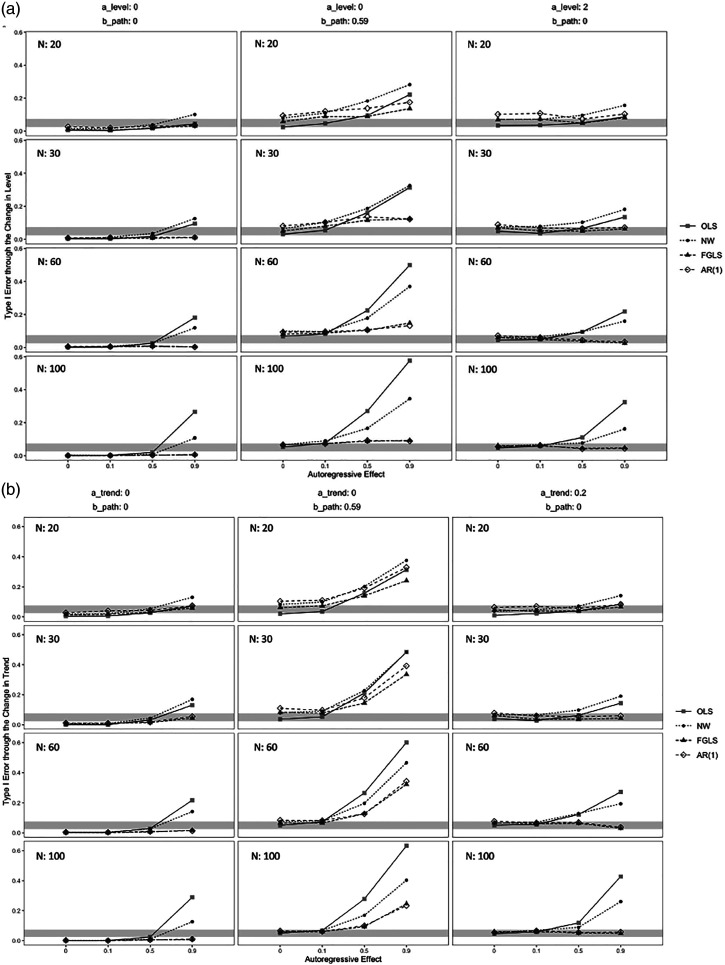
Type I Error of the Estimate of the Indirect Effect through the Change in Level and Trend. *Note.* Type I error rates of the interval estimate of the indirect effect defined as the change in level and trend over 1000 replications. The shaded area represents the acceptable range of Type I error rates between 0.025 and 0.075. Different values for the *a* path defined as the change in trend did not impact the Type I error rates for the change in level, and different values for the *a* path defined as the change in level did not impact the Type I error rates for the change in trend. a_level = *a* path as the change in level. a_trend = *a* path as the change in trend. b_path = *b* path. N = sample size.

Through the change in level, Type I error rates generally increased as the sample size increased for OLS and NW, while Type I error rates decreased or remained stable for FGLS and AR(1) at large autoregressive effects (Figure 2(a)). Type I error rates equal to or below 0.075 were observed for FGLS and AR(1) in the majority of parameter combinations when *b* = 0. When *b* = 0 and *ρ* = 0.9, OLS and NW interval estimates had Type I error rates above 0.075. When *b* = 0.59 and *ρ* = 0.5 and 0.9, Type I error rates above 0.075 were observed for all methods. OLS and NW had considerably higher Type I error rates than FGLS and AR(1) at *N* = 60 and 100 and when the autoregressive effect was medium or large.

Through the change in trend, Type I error rates generally increased for NW and OLS and increased or remained at the same level for FGLS and AR(1) as the sample size increased at large autoregressive effects (Figure 2(b)). Type I error rates equal to or below 0.075 were observed for FGLS and AR(1) for all parameter combinations when *b* = 0. When *b* = 0 and *ρ* = 0.9, OLS and NW interval estimates had Type I error rates above 0.075. When *b* = 0.59 and *ρ* = 0.5 and 0.9, all methods had excessive Type I error rates, whereas FGLS and AR(1) had lower Type I error rates than OLS and NW.

### Coverage

Through the change in level, coverage was consistent across sample sizes for FGLS and AR(1), while coverage decreased for NW and OLS as the sample size increased in the majority of conditions (Figure S3A). FGLS and AR(1) generally had coverage above 0.925 when *b* = 0. Coverage was below 0.925 for NW and OLS when *ρ* = 0.9, *b* = 0, and *N* = 60 and 100. When *ρ* = 0.9 and *b* = 0.59, coverage increased as the sample size increased for FGLS and AR(1), while coverage decreased for OLS and NW. For nonzero *b* paths and large autoregressive effects, coverage approached 0.925 for FGLS and AR(1), while coverage was well below 0.925 for OLS and NW at larger sample sizes.

Through the change in trend, coverage decreased as sample size increased for OLS and NW at high levels of simulated autocorrelation in the majority of conditions (Figure S3B). Coverage above 0.925 was obtained for FGLS and AR(1) when *b* = 0 at all values of autocorrelation and sample sizes. NW and OLS had coverage below 0.925 when *ρ* = 0.9, *b* = 0, and *N* = 30, 60, and 100. When *b* = 0.59 and *ρ* = 0.9, coverage was below 0.925 for all methods, whereas FGLS and AR(1) had higher coverage than NW and OLS.

### Interval Width

Through the change in level, interval width was larger at *N* = 20 and 30 than at *N* = 60 and 100, and the performance across methods was consistent for smaller sample sizes (Figure S4A). There were discrepancies in the performance of methods at larger sample sizes and autoregressive effects. When *b* = 0, *ρ* = 0.9, and *N* = 60 and 100, FGLS and AR(1) had smaller interval widths than OLS and NW. When *b* = 0.59, *ρ* = 0.9, and *N* = 60 and 100, the interval estimates were more precise for OLS than the other methods.

Through the change in trend, interval width decreased as the sample size increased (Figure S4B). The methods performed similarly when *ρ* = 0, 0.1, and 0.5. When *b* = 0.59, *ρ* = 0.9, and *N* = 20 and 30, FGLS had larger interval widths than the other methods.

### Single Mediator Model: Missing Data

#### Bias and Efficiency

Through the change in level, the point estimates of the indirect effect were unbiased when the proportion of missingness was 0.2 in most conditions (Figure S5A). When the proportion of missingness was large (0.5), the point estimates were biased for most combinations of parameter values. Relative bias generally decreased as the autoregressive effect increased at *N* = 60 and 100. The results were consistent across autocorrelation handling methods. The standard deviation of the point estimate generally increased as the autoregressive effect and sample size increased (Figure S6). There were no major differences across missingness proportions of 0.2 and 0.5. NW and OLS were less efficient estimators than FGLS and AR(1) in most conditions when the autoregressive effect was large, and this effect was most evident at *N* = 60 and 100.

Through the change in trend, the relative bias generally decreased as the sample size increased (Figure S5B). Relative bias was unacceptable when missing = 0.5 and *N* = 20 and 30 for all methods. When *N* = 60, the point estimates were unbiased regardless of the proportion of missing data. When *N* = 100, the estimates were biased at several levels of autocorrelation. Relative bias was generally higher for larger proportions of missingness. The results were consistent across autocorrelation handling techniques. The standard deviation of the point estimate generally increased as the autoregressive effect increased and decreased as the sample size increased (Figure S7). The results were generally consistent across proportions of missingness and autocorrelation handling method. However, when missing = 0.2, *b* path = 0, and *ρ* = 0.9, FGLS and AR(1) had lower values of standard deviation than OLS and NW.

### Power

Through the change in level, power decreased as the proportion of missing data increased, and power increased as the sample size increased (Figure S8A). Power was inadequate for all autocorrelation handling methods when *N* = 20 and 30 at both levels of missingness. When *N* = 60, missing = 0.2, and *ρ* = 0.5 and 0.9, power was below 0.8. When *N* = 60 and missing = 0.5, power was unacceptable at all levels of autocorrelation. When *N* = 100, missing = 0.2, *ρ* = 0.9, values of power were below 0.8. At *N* = 100 and a_trend = 0.2, power was below 0.8. When *N* = 100 and *ρ* = 0 and 0.1, power was above 0.8. Power was consistently higher for OLS in most conditions when *ρ* = 0.9 and *N* = 60 and 100.

Through the change in trend, power generally decreased as the proportion of missingness increased, and power increased as the sample size increased (Figure S8B). Power was inadequate for all methods when *N* = 20 and 30. Power was generally above 0.8 when missing = 0.2 and *N* = 60 and 100. When *N* = 60, missing = 0.2, and *ρ* = 0.9, power was below 0.8. When *N* = 100, missing = 0.2, and *ρ* = 0.9, power was above 0.8 for OLS and slightly below 0.8 for the other autocorrelation handling techniques. When missing = 0.5, power was below 0.8 in most conditions. However, when *N* = 100, missing = 0.5, and the a_level = 0, power was above 0.8 when *ρ* = 0.5 for all methods, and near 0.8 for *ρ* = 0, 0.1, and 0.9. Power was slightly higher for OLS than NW, FGLS, and AR(1) when *ρ* = 0.9 and *N* = 60 and 100 for all parameter combinations.

### Type I Error Rate

Through the change in level, Type I error rates were within the acceptable range or below 0.025 when *ρ* = 0 and 0.1 across both levels of missingness and for all autocorrelation handling methods (Figure S9). As the sample size increased, the Type I error rates increased when *ρ* = 0.5 and 0.9 in several conditions. When *ρ* = 0.5, *b* path = 0.59, and missing = 0.2, the Type I error rates were above 0.075 when *N* = 30, 60, and 100. When *ρ* = 0.9 and *N* = 60 and 100, the Type I error rates were unacceptable in most conditions for OLS and NW and in several conditions for FGLS and AR(1).

Through the change in trend, Type I error rates were acceptable or below 0.025 when *ρ* = 0 and 0.1 at both levels of missingness (Figure S10). As the sample size increased, the Type I error rates increased when *ρ* = 0.5 and 0.9 in several conditions across both proportions of missingness. All methods had instances of Type I error rates above 0.075 when *ρ* = 0.5 and 0.9, *N* = 60 and 100, *b* path = 0.59, and missing = 0.2 and 0.5. OLS and NW performed worse than FGLS and AR(1) at large autoregressive effects for *N* = 30, 60, and 100 in several conditions, and this effect was most noticeable for nonzero *b* paths.

### Coverage

Through the change in level, all autocorrelation handling techniques had values of coverage within or above the acceptable range when *ρ* = 0 and 0.1 (Figure S11). As the sample size increased, values of coverage generally decreased when *ρ* = 0.5 and 0.9. Coverage below 0.925 was observed in several parameter combinations when *ρ* = 0.5 and 0.9 and *N* = 30, 60, and 100, whereas OLS and NW had lower coverage than FGLS and AR(1) across both levels of missingness. A larger proportion of missingness resulted in higher coverage for OLS and NW when *ρ* = 0.9.

Through the change in trend, the methods had coverage within or above the robustness criterion for all parameter combinations when *ρ* = 0 and 0.1 (Figure S12). As the sample size increased, values of coverage generally decreased when *ρ* = 0.5 and 0.9. When missing = 0.2, *b* path = 0.59, *ρ* = 0.5 and 0.9, and *N* = 30, 60, and 100, coverage was below 0.925. OLS and NW performed worse than FGLS and AR(1).

### Interval Width

Through the change in level, interval width generally increased as the autoregressive effect and proportion of missingness increased (Figure S13). As the sample size increased, the interval width decreased. OLS had slightly smaller interval widths than NW, FGLS, and AR(1) when the autoregressive effect was large at *N* = 60 and 100.

Through the change in trend, interval width generally increased as the autoregressive effect and the percentage of missing data increased (Figure S14). As the sample size increased, the interval width decreased. When *ρ* = 0.9 and *N* = 60 and 100, OLS had consistently smaller interval widths than NW, FGLS, and AR(1).

## Discussion

In the present study, we evaluated various techniques for modeling serially correlated error terms and examined the performance of MI as a missing data handling technique for a MCAR mechanism for piecewise regression analysis in SCEDs. Specifically, we investigated the performance of the autocorrelation handling methods and MI in terms of the statistical properties of the point and interval estimates of the indirect effect for a single mediator model. After data were simulated with different values of autocorrelation (*ρ* = 0, 0.1, 0.5, and 0.9) and missing data (0% in the first simulation study and 20% and 50% in the second simulation study), the performance of the methods was assessed in terms of bias, efficiency, power, Type I error rate, coverage, and interval width.

Results from the single mediator model simulation without missing data revealed that OLS, NW, FGLS, and AR(1) generally have unbiased point estimates. The results were consistent across methods for small and medium autoregressive effects. At large autoregressive effects (i.e., *ρ* = 0.9), OLS and NW performed worse than FGLS and AR(1) in terms of relative bias for some parameter combinations. The four methods were less efficient at larger simulated autoregressive effects and smaller sample sizes. AR(1) and FGLS were more efficient estimators of the indirect effect at large autoregressive effects. Type I error rates were desirable or below 0.025 in most parameter combinations and sample sizes for AR(1) and FGLS. As sample size increased, however, Type I error rates increased for OLS and NW, particularly for large autoregressive effects. A similar pattern was observed in terms of coverage, where the performance of our methods was hindered by large autoregressive effects and sample sizes for OLS and NW. Coverage was acceptable or above 0.975 in most conditions for AR(1) and FGLS. The performance of NW and OLS in terms of Type I error rate and coverage were noticeably worse compared to AR(1) and FGLS at *ρ* = 0.9, and the discrepancy between performance increased as the sample size increased. When the sample size was small (i.e., *N* = 20 and 30), low power was achieved for all methods. At larger sample sizes (i.e., *N* = 60 and 100), satisfactory power was achieved in the majority of conditions except for when the simulated autocorrelation was substantial (*ρ* = 0.9). OLS had higher power than FGLS, AR(1), and NW in all conditions when *ρ* = 0.9 and *N* = 60 and 100.

Results from the simulation with missing data revealed that in general point estimates were unbiased when the proportion of missingness was 20%. However, the performance of the methods was unacceptable in terms of relative bias at several parameter combinations and sample sizes when a large proportion of missingness (50%) was introduced in the data. This finding is supported by Chen et al. (2020) who found a high missing rate negatively impacted the performance of EM in terms of relative bias. However, it is worth noting that the highest missing rate evaluated was 30%, and they simulated a lower missing rate for the A phase than the B phase (Chen et al., 2020). Our results indicate that the missing rate on its own affected the relative bias of the point estimate. At *N* = 60 and 100 and when the rate of missing data was equal to 20% and 50%, the relative bias generally decreased as the autoregressive effect increased. This finding is consistent with the literature demonstrating that the inclusion of auxiliary variables when the correlation among variables is high may be beneficial for the performance of multiple imputation models (Hardt et al., 2012). Thus, the inclusion of *M* and *Y* as auxiliary variables in our simulations may have improved the performance of our methods at large autoregressive effects. Chen et al. (2020) also found that relative bias was reduced for larger autoregressive effects in several conditions when the proportion of missingness in the intervention phase was equal to 20%. Consistent with the results from the simulation without missing data, the methods were less efficient at larger autoregressive effects, and the results were not negatively impacted by larger proportions of missingness. The results of Chen et al. (2020) are consistent with this finding, where the effect of the missing rate on the precision of the estimates was minimal compared to the impact of the autocorrelation. FGLS and AR(1) were more efficient estimators of the indirect effect when *ρ* = 0.9. Values of power below 0.8 were observed for all parameter combinations at *N* = 20 and 30. Power was acceptable at larger sample sizes excluding conditions when the percentage of missing data was equal to 50%. Type I error rates were acceptable for all methods when the level of simulated autocorrelation was small. However, when *ρ* = 0.5 and 0.9, Type I error rates were above 0.075 for larger sample sizes. Interestingly, Type I error rates tended to increase for medium and large autoregressive effects as the sample size increased. OLS had consistently higher Type I error rates at large autoregressive effects. Higher proportions of missingness did not negatively impact the Type I error rates. Coverage was within the acceptable range or above the upper limit of the nominal interval for small autoregressive effects. Consistent with the results of Type I error rates, coverage decreased as sample size increased for large autoregressive effects. Coverage was below 0.925 in several conditions when at *ρ* = 0.9. OLS and NW performed worse than FGLS and AR(1), and this effect was most noticeable through changes in level and at larger sample sizes. Coverage, like Type I error rates, decreased as the simulated autocorrelation increased. Interestingly, the performance of our methods improved in terms of the Type I error rate and coverage when a higher proportion of missingness was simulated. Finally, interval width increased as the percentage of missing data and autoregressive effect increased and decreased as the sample size increased.

The findings from our study revealed that (1) FGLS and AR(1) are promising methods for modeling autocorrelation, and (2) MI is a valuable missing data handling technique in piecewise regression analysis in SCEDs. The first major finding is supported by the low Type I error rates, high coverage, and high efficiency observed when low missing data rates (0% and 20%) were simulated. The second major finding is supported by the similar performance of the methods across missing data rates of 0% and 20% in terms of relative bias, efficiency, and power, and the superior performance of the methods in terms of Type I error and coverage for larger proportions of missingness when a large autoregressive effect was simulated. In light of these findings, we provide recommendations for applied researchers in subsequent paragraphs.

When acceptable Type I error rates, coverage, and high efficiency are sought out, AR(1) and FGLS would be recommended at all sample sizes, autoregressive effects, and proportion of missingness. However, the estimates were biased, and power was below 0.8 when a large proportion of missing data was simulated for all methods. When higher values of power are desired, the choice of method depends on the amount of autocorrelation in the data. OLS would be suggested when the amount of autocorrelation is medium or large. However, one should note that this choice of parameter estimation comes at the cost of an increased Type I error rate. When the autoregressive effect in the data is minimal, FGLS and AR(1) would be recommended. Procedures for estimating autocorrelation for the methods evaluated in the paper are provided in the Supplemental Materials. All autocorrelation handling methods resulted in values of power below 0.8 when the sample size was small. In order to achieve adequate power to detect indirect effects using the methods in this study, larger sample sizes (i.e., *N* = 60 and 100) are recommended. When *N* = 60 and 100, the power was near the acceptable value of 0.8 for all methods when *ρ* ranged from 0 to 0.5, and the percentage of missing data was equal to 0% and 20%.

### Limitations and Suggestions for Future Research

There are several limitations to the current study. First, it may be unrealistic to expect researchers to collect the 60 to 100 data points per participant necessary to attain adequate power to detect indirect effects. As Shadish and Sullivan (2011) noted, 90.6% of single-case design studies had less than 50 observations. Fortunately, the development of smartphones, tablets, and handheld computers has revolutionized our ability to collect data. Advancements in real-time monitoring technology have facilitated the use of ecological momentary assessment (EMA) in which researchers acquire repeated data of participants’ behaviors and experiences (Shiffman et al., 2008). EMA can readily document the behavior of an individual across time, revealing the effects of an intervention or treatment. New technologies have also promoted the use of passive real-time monitoring (Kleiman & Nock, 2017). Passive monitoring involves collecting data without requiring active participation and data entry from the individual. This enables researchers to collect data passively using features on smartphones such as screen time and social media activity (Vilardaga et al., 2014). The advantages of real-time monitoring technology in SCEDs are detailed thoroughly in Bentley et al. (2019).

Despite the finding that power increased as sample size increased when the proportion of missing data was large, power was below the nominal level, and relative bias exceeded 0.10 when 50% missing data was simulated. More methodological work is needed to develop optimal missing data handling methods to reduce bias and increase power for piecewise regression analysis in SCEDs. Several methods for multivariate data imputation have been proposed, including imputation based on maximum likelihood (MLMI; von Hippel & Bartlett, 2021) and predictive mean matching (PMM; Morris et al., 2014). Various R packages for performing imputation in time series data have been developed (Moritz et al., 2015), including the R package imputeTS (Moritz & Bartz-Beielstein, 2017) for univariate time series imputation and the R package Amelia II (Honaker et al., 2011), a bootstrap-based EM algorithm implemented for imputing missing values in multivariate time series data.

Another limitation of our study lies in the choice to consider only positive autocorrelations, yet negative autocorrelations have been reported in SCEDs (Harrington & Velicer, 2015; Parker et al., 2005). Studies have revealed differences in the performance of missing data handling methods under negative autocorrelations with time series data (Velicer & Colby, 2005a, 2005b). Future simulations should examine the performance of MI under both positive and negative autocorrelation values. The negative relationship between relative bias and autoregressive effect also warrants further investigation. Our simulation study and empirical example evaluated AB designs, and researchers may be interested in other types of SCEDs, such as multiple-baseline designs or alternating treatment designs. Shadish and Sullivan (2011) found that the multiple-baseline design is most commonly used in SCED research. However, methods for obtaining numerical estimates of indirect effects for multiple-baseline and intervention designs have yet to be described. Furthermore, we assessed a single mediator model, and often researchers are interested in evaluating more than one mediator. Future research is needed to identify optimal techniques for modeling autocorrelation and handling missing data for two mediator models. Additionally, we evaluated data that followed an MCAR mechanism, although data that is MAR, where a missing observation depends on the observed data, may be more realistic in empirical SCEDs.

Future research is needed to examine the effects of lagged and cross-lagged variables in piecewise regression models for SCEDs. The proposed method does not allow for lagged effects, for example, of the mediator $M_{t}$ at a measurement occasion t to the outcome $Y_{t+1}$ at the subsequent measurement occasion. Furthermore, we assume equidistant time intervals between measurement occasions. Mediation analysis with lagged effects can be done, for example, using multivariate time-series models (Lutkepohl, 2005), cross-lagged panel models (Usami et al., 2019; Zyphur et al., 2020), or non- and semi-parametric models for causal mediation analysis (Shpitser, 2013; Zheng & van der Laan, 2017). The assumption of equidistant time intervals can be relaxed by using continuous time models (Albert et al., 2019; Deboeck & Preacher, 2016).

In the present study, we did not distinguish between the number of time points in the baseline and intervention phase. However, it is common in SCEDs that the length of the treatment phase exceeds that of the baseline phase (Ferron et al., 2010; Shadish & Sullivan, 2011). Given that this is the first simulation study to examine methods for handling missing data and autocorrelation in piecewise regression analysis for mediation analysis SCEDs, we opted to simplify the design. Future research might examine the impact of autocorrelation, missing data, and varying the lengths of the baseline and intervention phases on the performance of MI.

## Supplemental Material

sj-pdf-1-ehp-10.1177_01632787211071136 – Supplemental Material for Methods for Modeling Autocorrelation and Handling Missing Data in Mediation Analysis in Single Case Experimental Designs (SCEDs) Click here for additional data file. Supplemental Material, sj-pdf-1-ehp-10.1177_01632787211071136 for Methods for Modeling Autocorrelation and Handling Missing Data in Mediation Analysis in Single Case Experimental Designs (SCEDs) by Emma Somer, Christian Gische and Milica Miočević in Evaluation & the Health Professions

## Conclusion

Using mediation analysis to test intervention effects in SCEDs can provide insight into the mechanisms through which interventions achieve their effects for individual participants. This paper evaluated piecewise regression analysis for a single mediator model comparing OLS to three methods for handling autocorrelation, NW, AR(1), and FGLS, and MI under various proportions (20% and 50%) of missing data. The methods were illustrated using data from a walking intervention for osteoarthritis. The simulations indicate that AR(1) and FGLS are promising techniques for modeling autocorrelation, and MI is a promising method for handling missing data in SCEDs for single mediator models. Our results suggest that sample sizes larger than those typically found in SCEDs are recommended to attain acceptable power using the methods evaluated in this study. As the number of tools facilitating data collection continues to rise, larger sample sizes necessary to detect indirect effects in SCEDs using piecewise regression analysis may become more feasible. We hope the results of our simulation studies will contribute to the current scholarship on mediation analysis in SCEDs and promote further research on autocorrelation handling and missing data handling methods in single-case studies.

## Notes

1 The time index t refers to the measurement occasion, and we assume equidistant time intervals between measurement occasions (e.g., days, weeks). Thus, X_t_, M_t_, and Y_t_ are measured at the same occasion (e.g., during the same day). However, we assume that within a measurement occasion (e.g., during a day), the cause variables are measured prior to the effect variables (e.g., X_t_ is measured before M_t_ during the same day).

2 RMediation implicitly assumes that the standard errors are consistent, which may not be the case in OLS with nonzero autocorrelation.

3 Note that the proportion of variation accounted for by a_trend, a_level, and the mediator no longer correspond to the intended values of *R*^2^ that led to labeling these effects as large; therefore, the selected effect sizes should no longer be considered as large effects.
