# Supplementary material for: Methods for Modeling Autocorrelation and Handling Missing Data in Mediation Analysis in Single Case Experimental Designs (SCEDs)
Source: Eval Health Prof. 2022 Feb 26;45(1):36–53. doi: 10.1177/01632787211071136 (PMC8980456; doi:10.1177/01632787211071136)
Supplement: sj-pdf-1-ehp-10.1177_01632787211071136 – Supplemental Material for Methods for Modeling Autocorrelation and Handling Missing Data in Mediation Analysis in Single Case Experimental Designs (SCEDs) [file sj-pdf-1-ehp-10.1177_01632787211071136.pdf]

Supplemental Figures for Methods for Modeling Autocorrelation and Handling Missing Data in Mediation Analysis in Single Case Experimental Designs (SCEDs)

<u>Figures for the Simulation Study without Missing Data</u>

Figure S1

Relative Bias of the Point Estimate of the Indirect Effect through the Change in Level and Trend

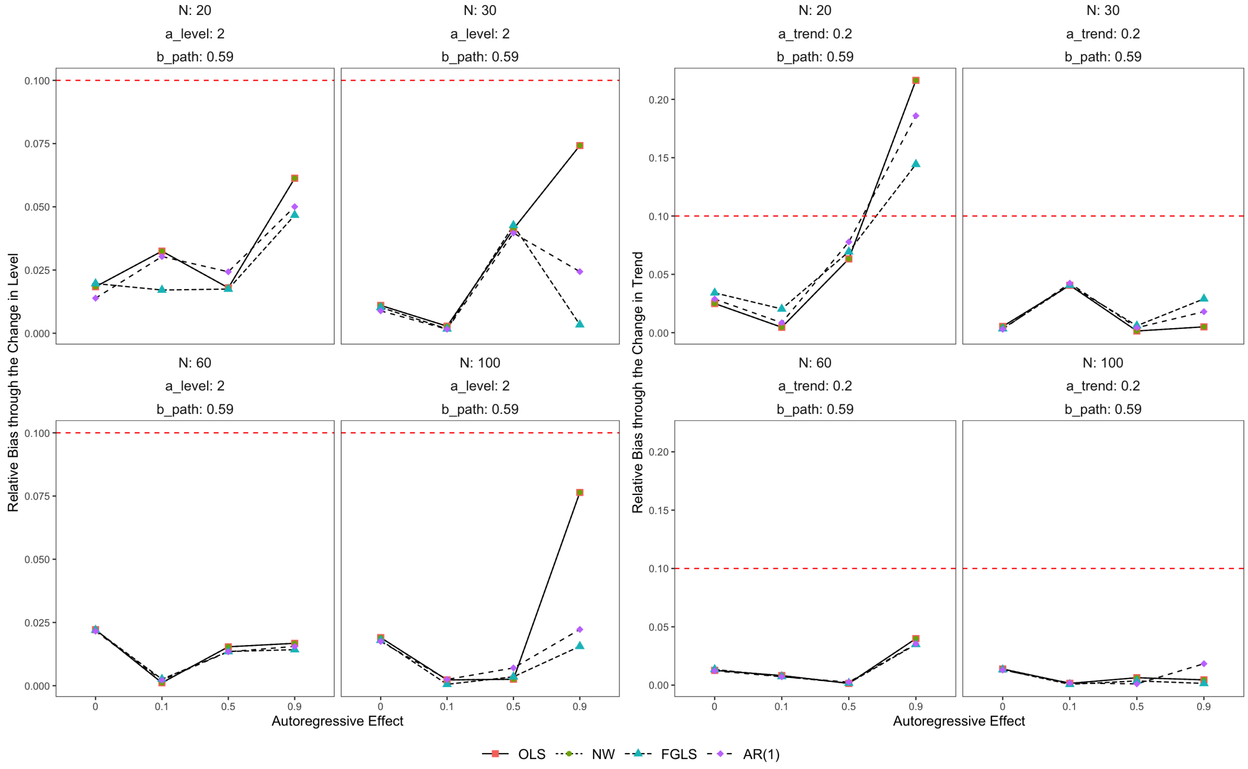

Note. The absolute values of the relative bias of the point estimate of the indirect effect defined through the change in level and trend over 1,000 replications. Different values for the *a* path defined as the change in trend did not impact the relative bias for the change in level, and different values for the *a* path defined as the change in level did not impact the relative bias for the change in trend. Horizontal dotted line represents a relative bias value of 0.10. a_level = *a* path as the change in level. a_trend = *a* path as the change in trend. b_path = *b* path. N = sample size.

Figure S2

Standard Deviation of the Indirect Effect through the Change in Level and Trend

A

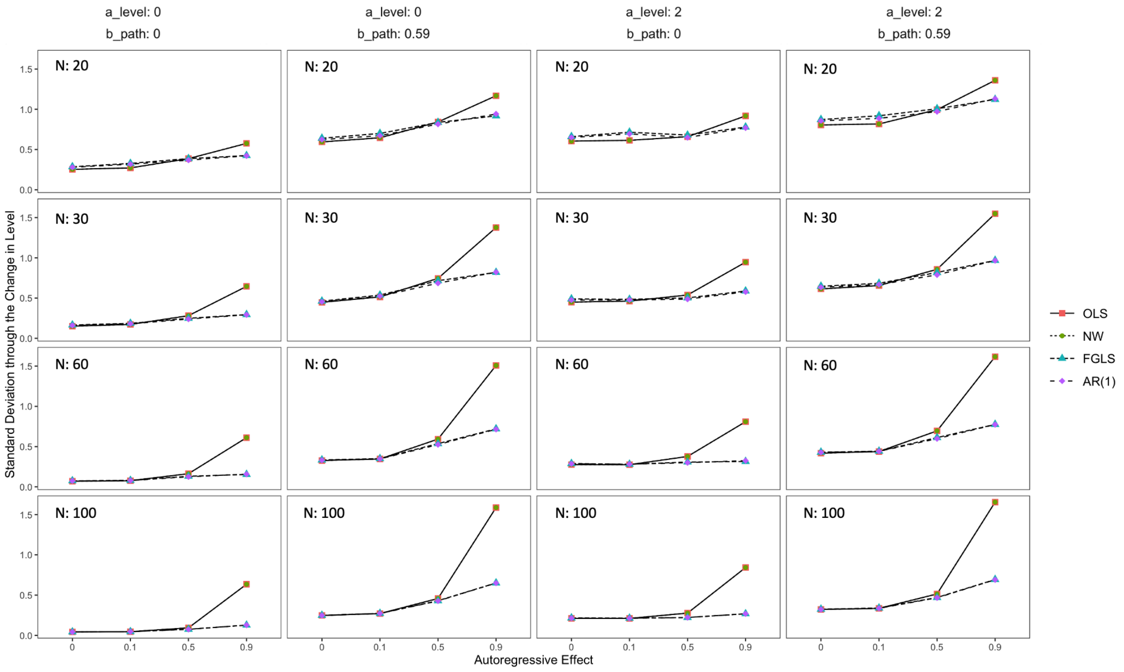

B

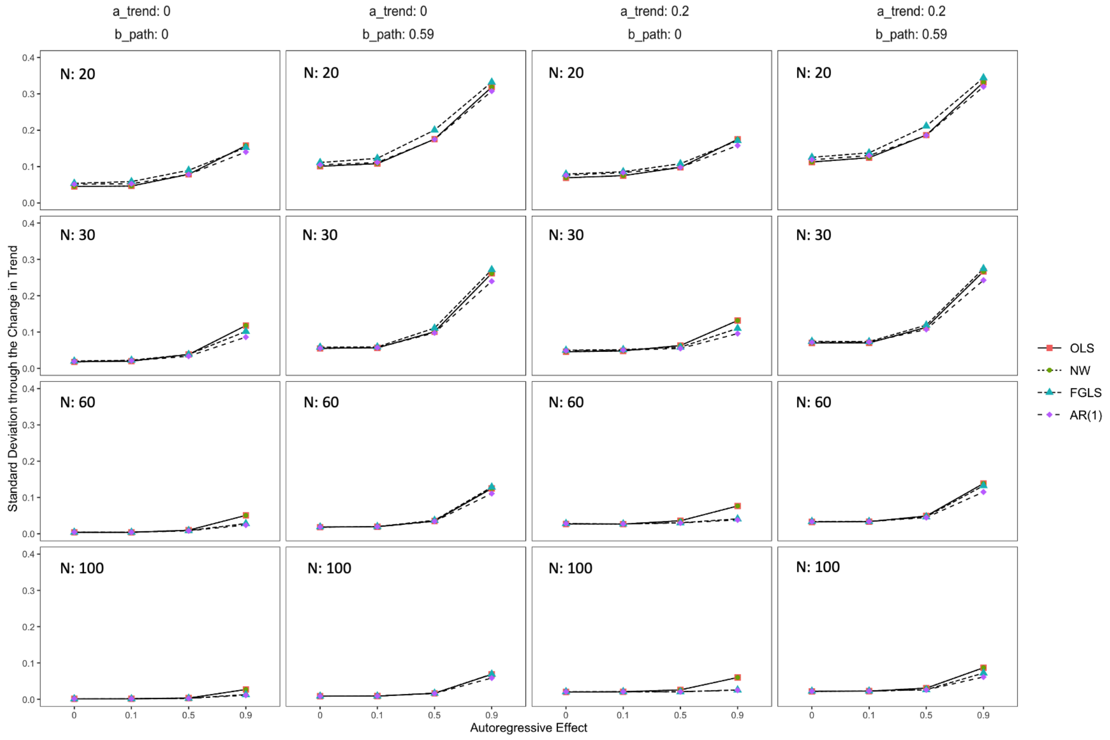

Note. Standard deviation of the point estimate of the indirect effect defined through the change in level (A) and trend (B) over 1,000 replications for a given set of parameter conditions are displayed as a measure of efficiency. a_level = *a* path as the change in level. a_trend = *a* path as the change in trend. b_path = *b* path. N = sample size.

Figure S3

Coverage of the Indirect Effect through the Change in Level and Trend

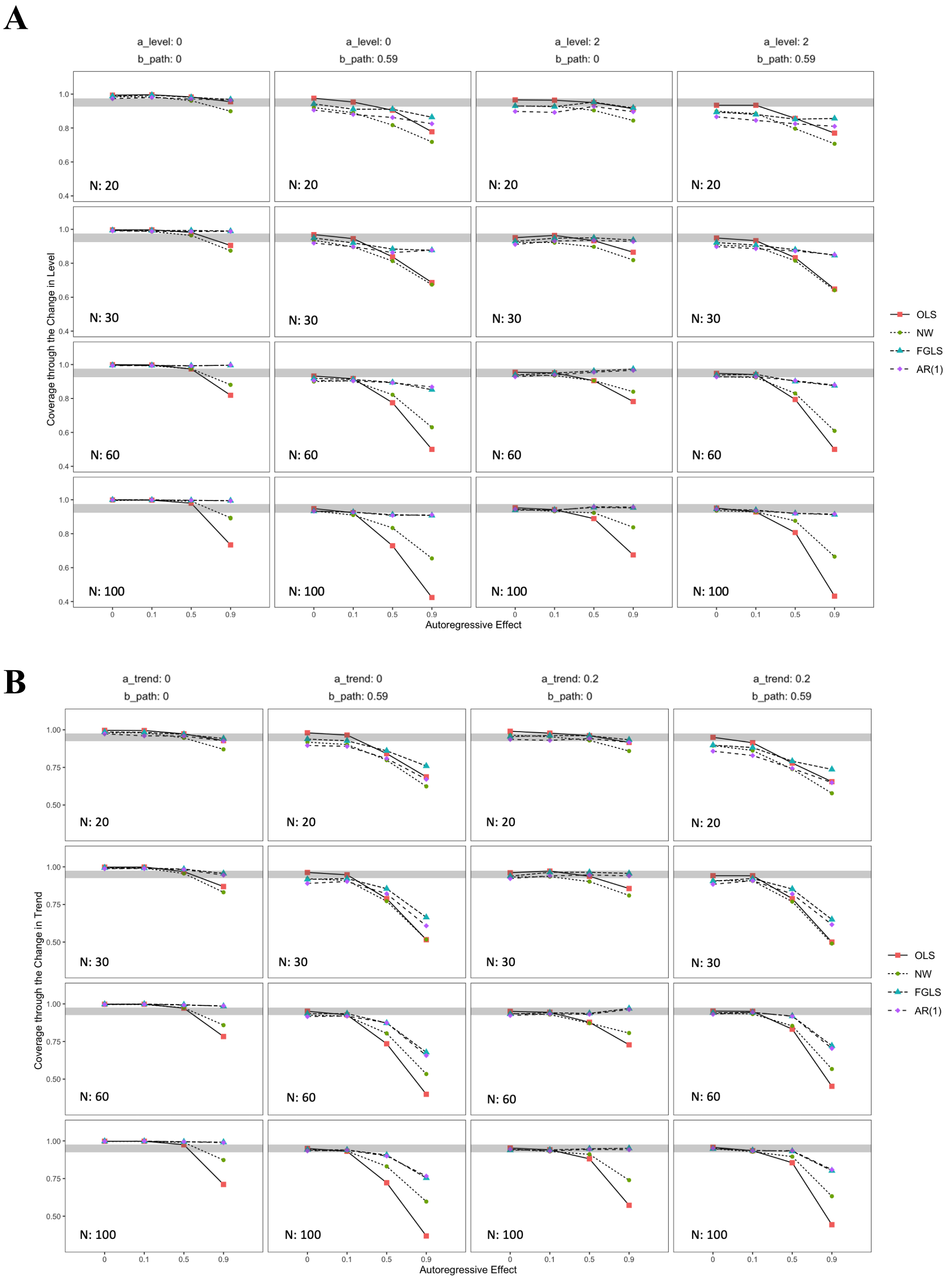

Note. Coverage of the interval estimate of the indirect effect defined through the change in level (A) and trend (B) over 1,000 replications. Shaded area represents values of acceptable coverage

between 0.925 and 0.975. a_level = *a* path as the change in level. a_trend = *a* path as the change in trend. b_path = *b* path. N = sample size.

Figure S4

Interval Width of the Indirect Effect through the Change in Level and Trend

A

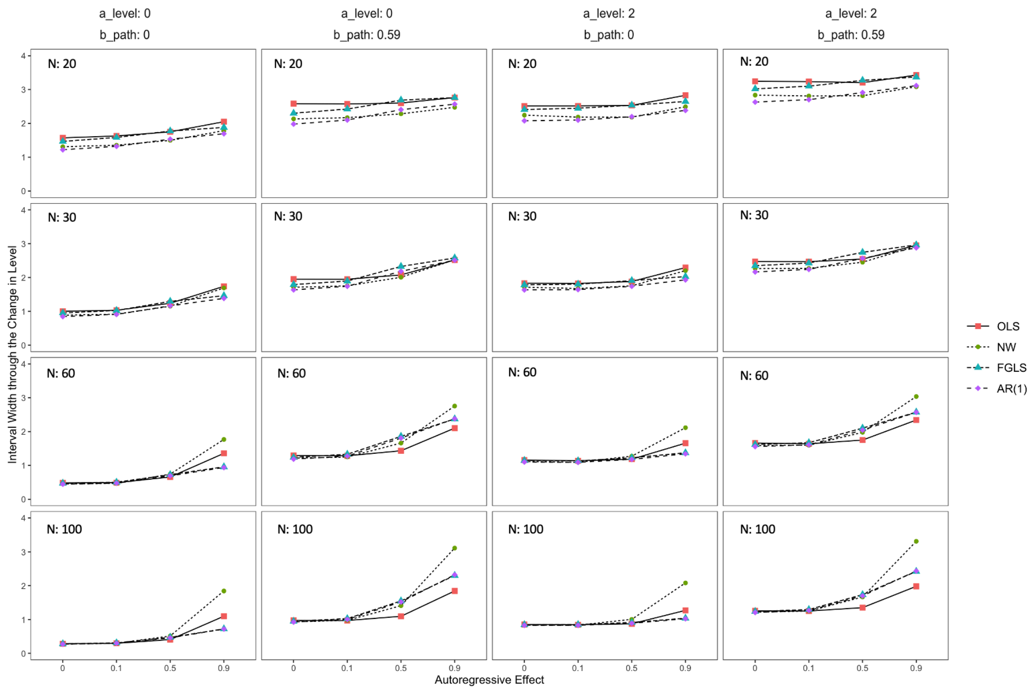

B

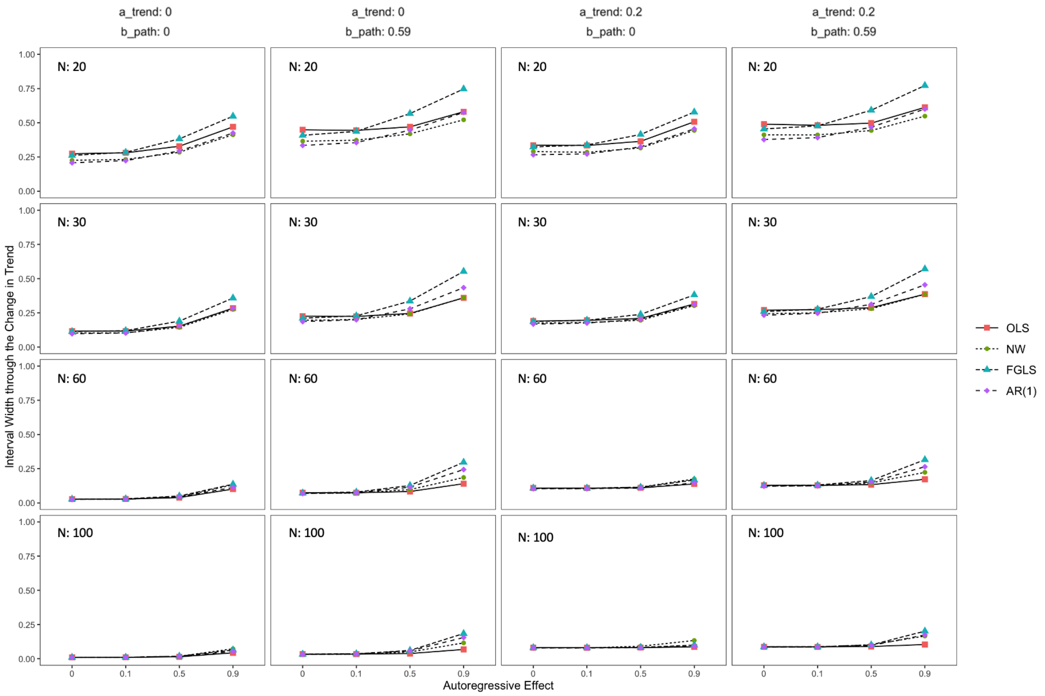

Note. Interval width of the indirect effect defined through the change in level (A) and trend (B) over 1,000 replications. a_level = *a* path as the change in level. a_trend = *a* path as the change in trend. b_path = *b* path. N = sample size.

Figures for the Simulation Study with Missing Data

Figure S5

Relative Bias of the Point Estimate of the Indirect Effect through the Change in Level and Trend

A

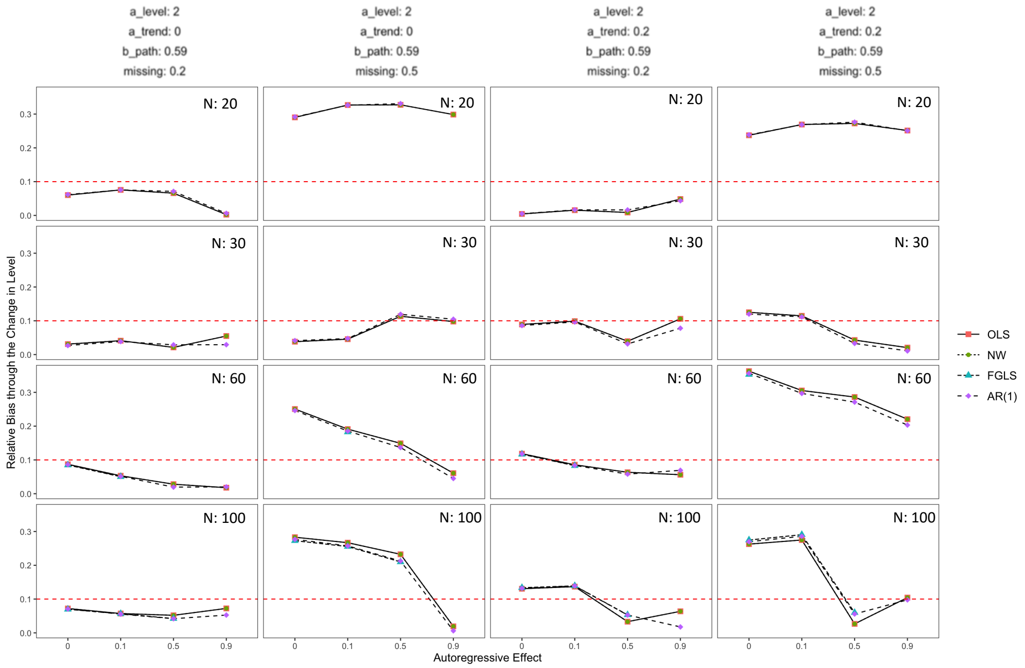

B

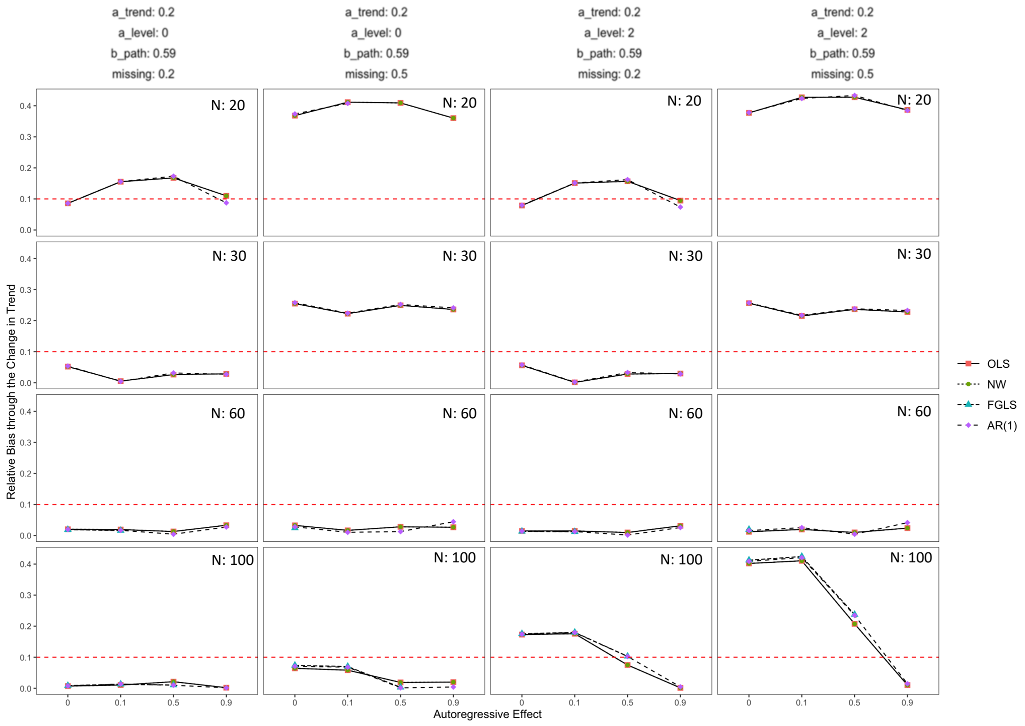

Note. The absolute values of the relative bias of the point estimate of the indirect effect defined through the change in level (A) and trend (B) over 1,000 replications for different proportions of missingness. a_level = *a* path as the change in level. Horizontal dotted line represents a relative bias value of 0.10. a_trend = *a* path as the change in trend. b_path = *b* path. N = sample size. missing = percentage of missing data.

Figure S6

Standard Deviation of the Indirect Effect through the Change in Level

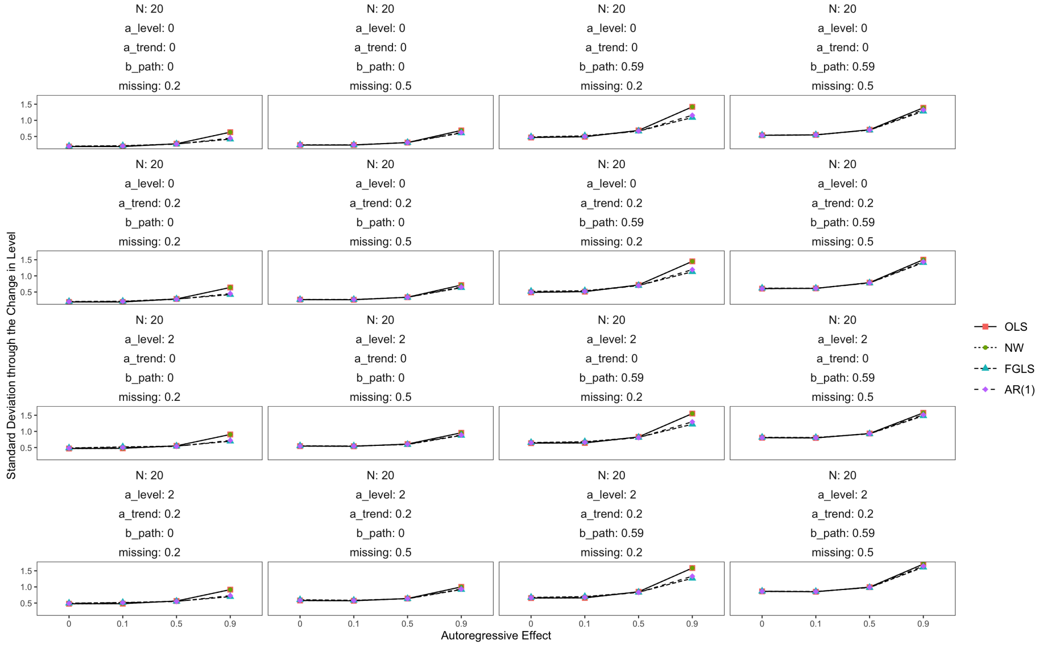

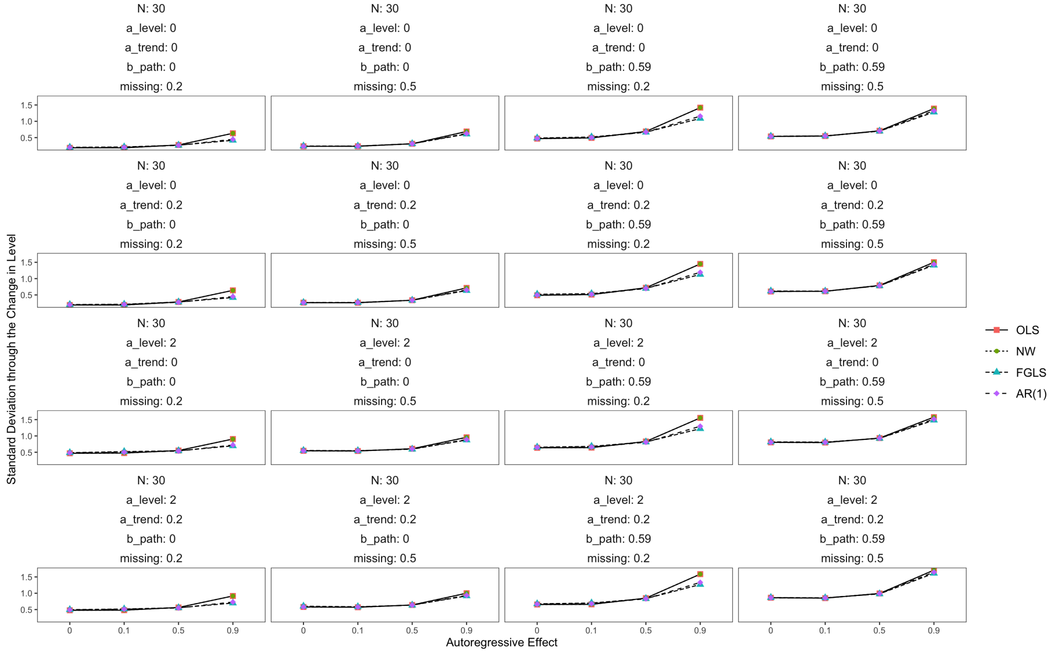

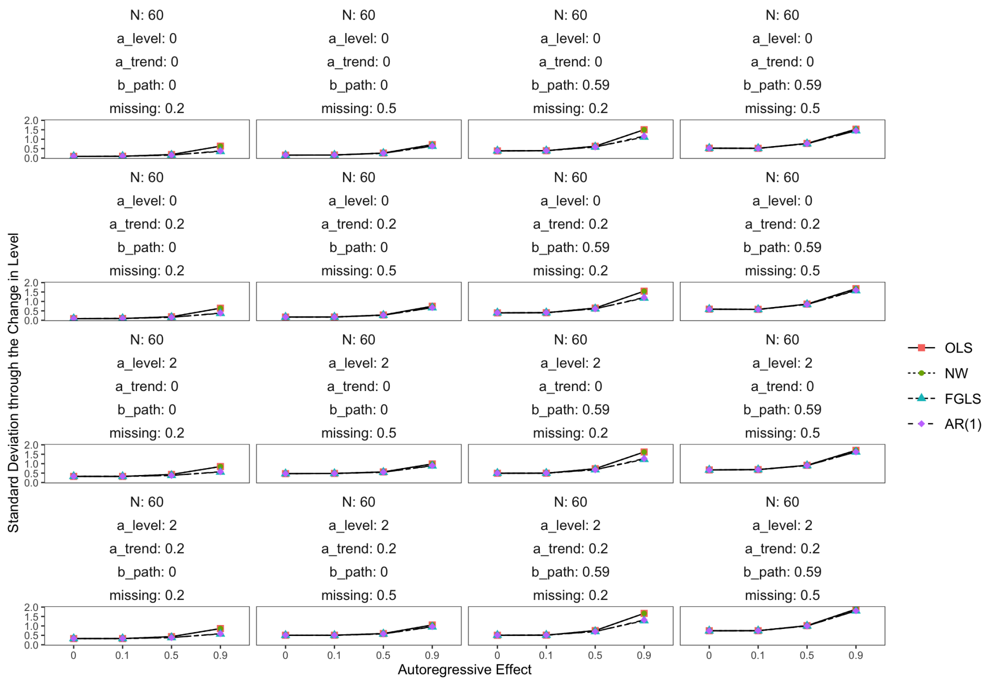

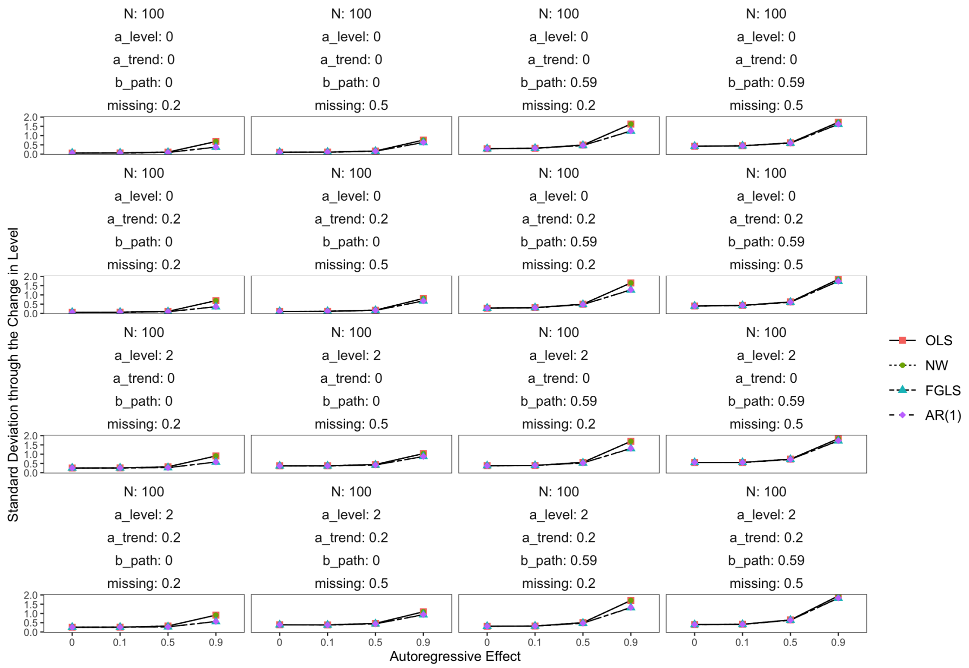

Note. Standard deviation of the point estimate of the indirect effect defined through the change in level over 1,000 replications for a given set of parameter conditions are displayed as a measure of efficiency. a_level = *a* path as the change in level. a_trend = *a* path as the change in trend. b_path = *b* path. N = sample size. missing = percentage of missing data.

Figure S7

Standard Deviation of the Indirect Effect through the Change in Trend

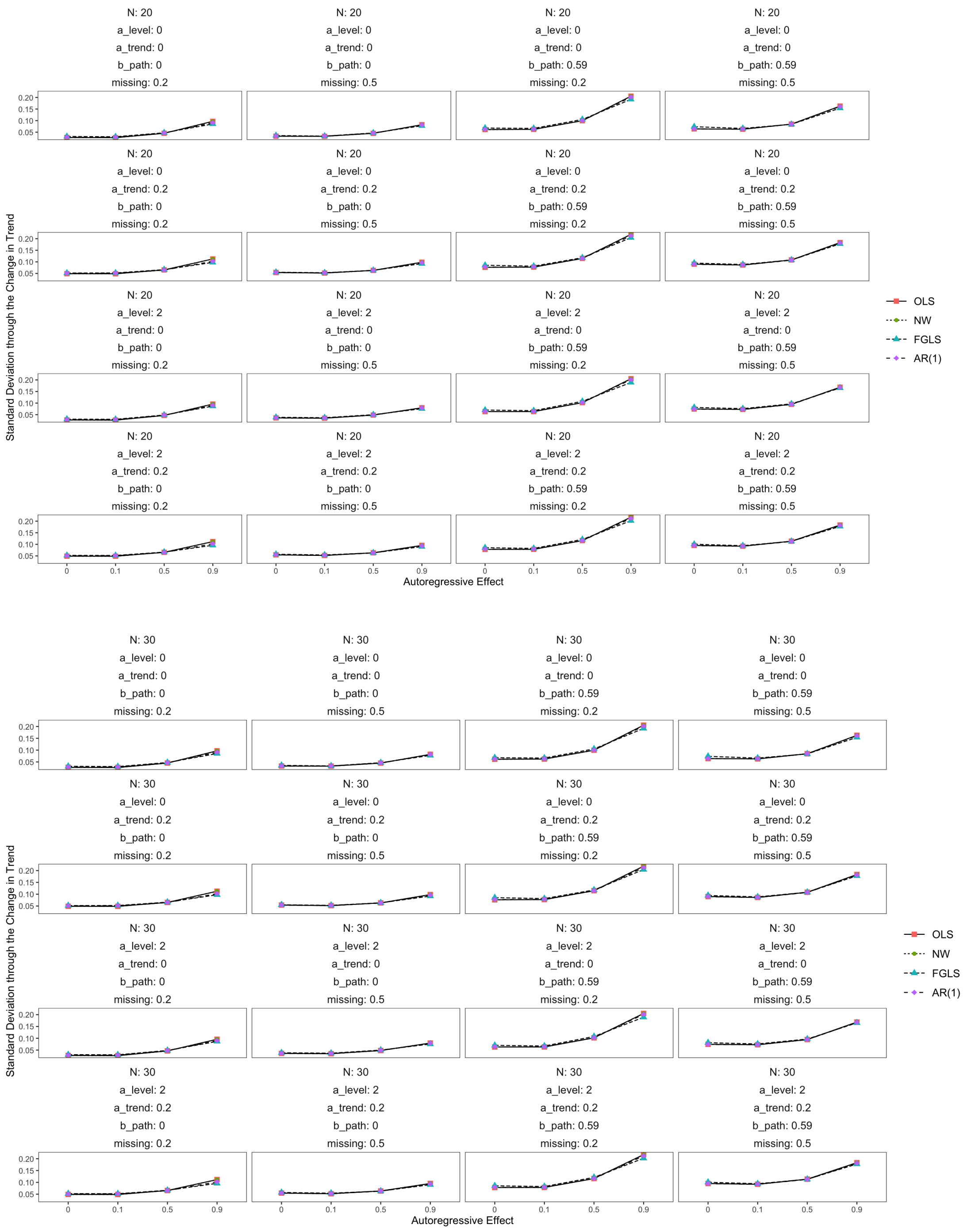

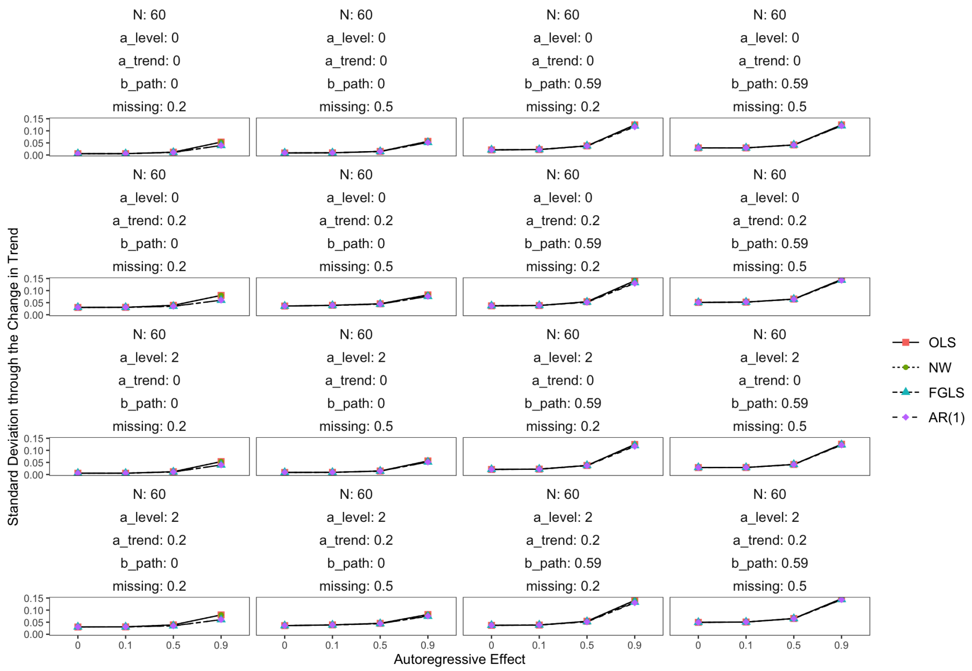

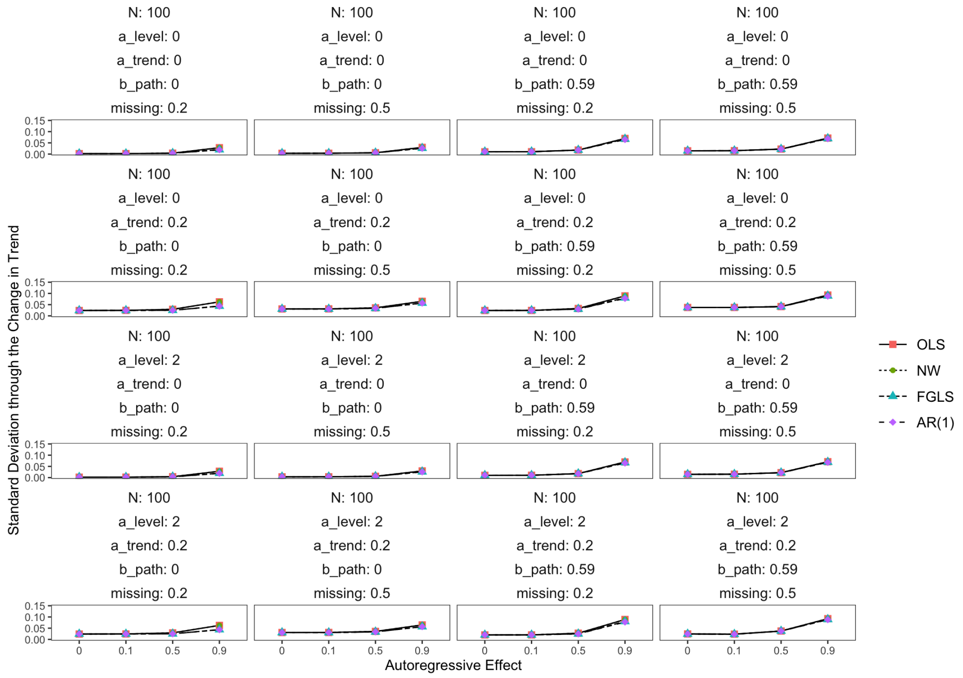

Note. Standard deviation of the point estimate of the indirect effect defined through the change in trend over 1,000 replications for a given set of parameter conditions are displayed as a measure of efficiency. a_level = *a* path as the change in level. a_trend = *a* path as the change in trend. b_path = *b* path. N = sample size. missing = percentage of missing data.

Figure S8

Power of the Interval Estimate of the Indirect Effect through the Change in Level and Trend

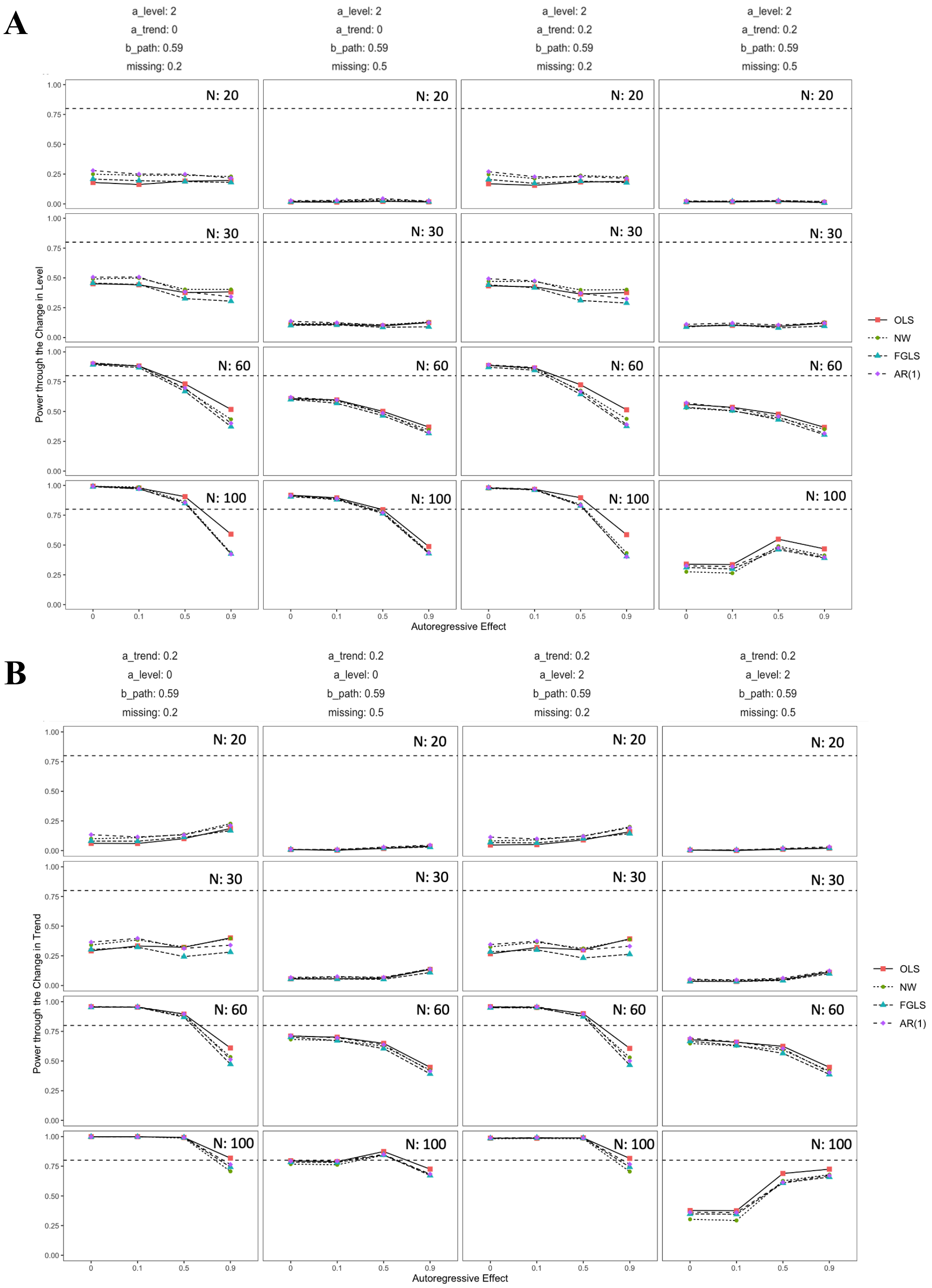

Note. Power of the interval estimate of the indirect effect defined through the change in level (A) and trend (B) over 1,000 replications for different proportions of missing data. The dotted line

represents power of 0.8. a_level = *a* path as the change in level. a_trend = *a* path as the change in trend. b_path = *b* path. N = sample size. missing = percentage of missing data.

Figure S9

Type I Error of the Estimate of the Indirect Effect through the Change in Level

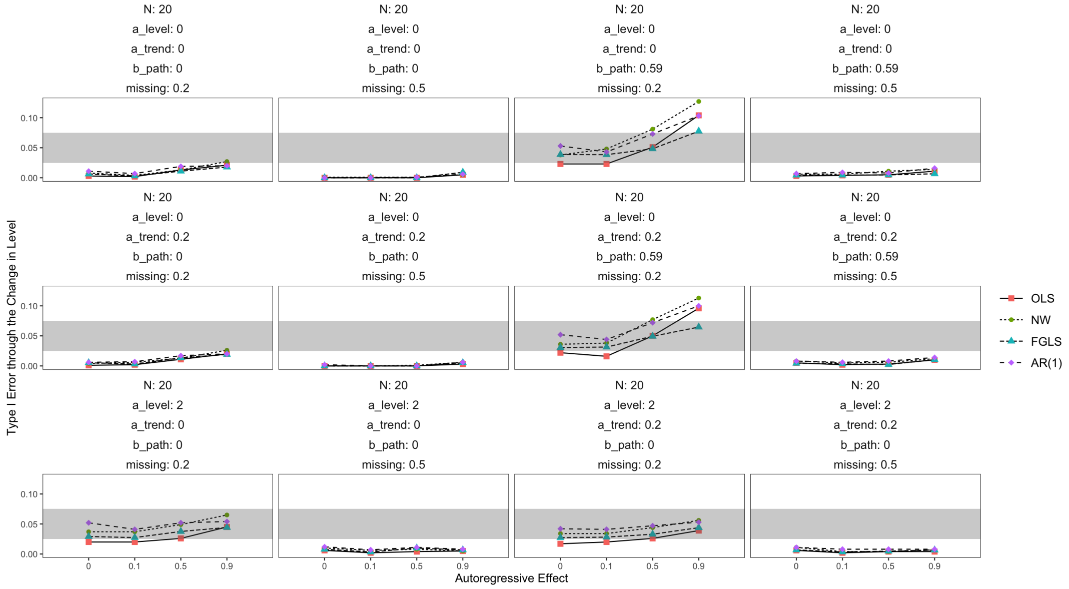

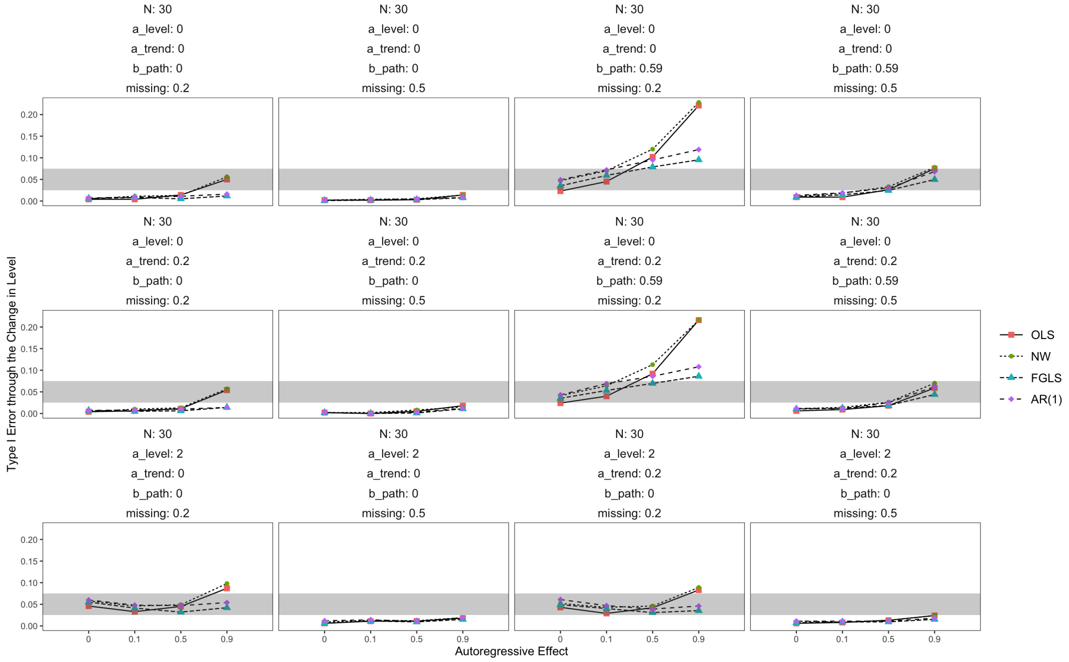

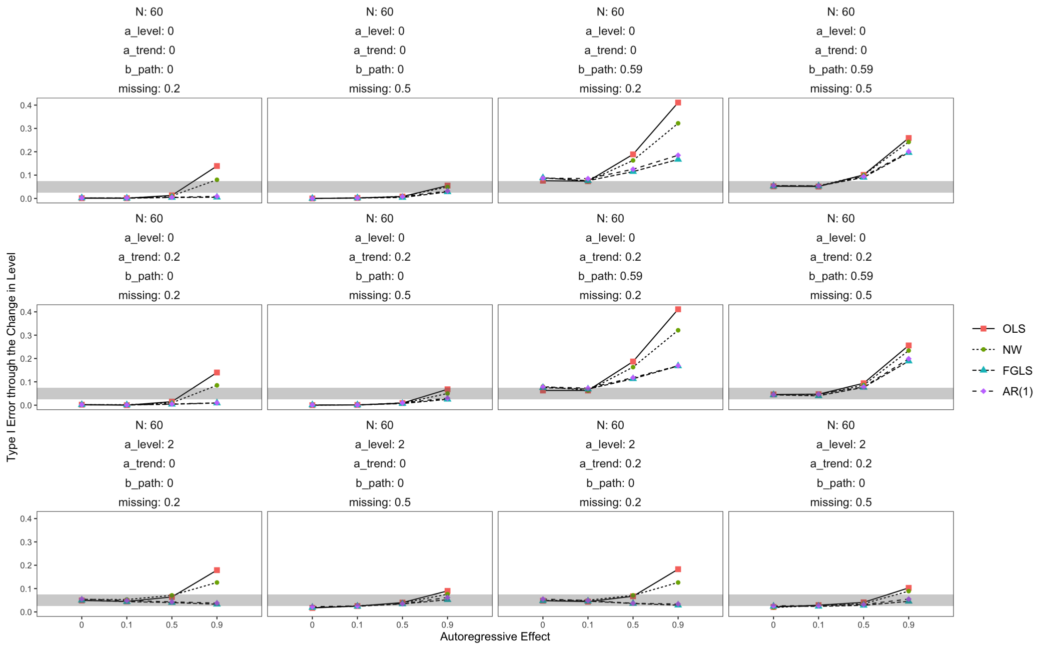

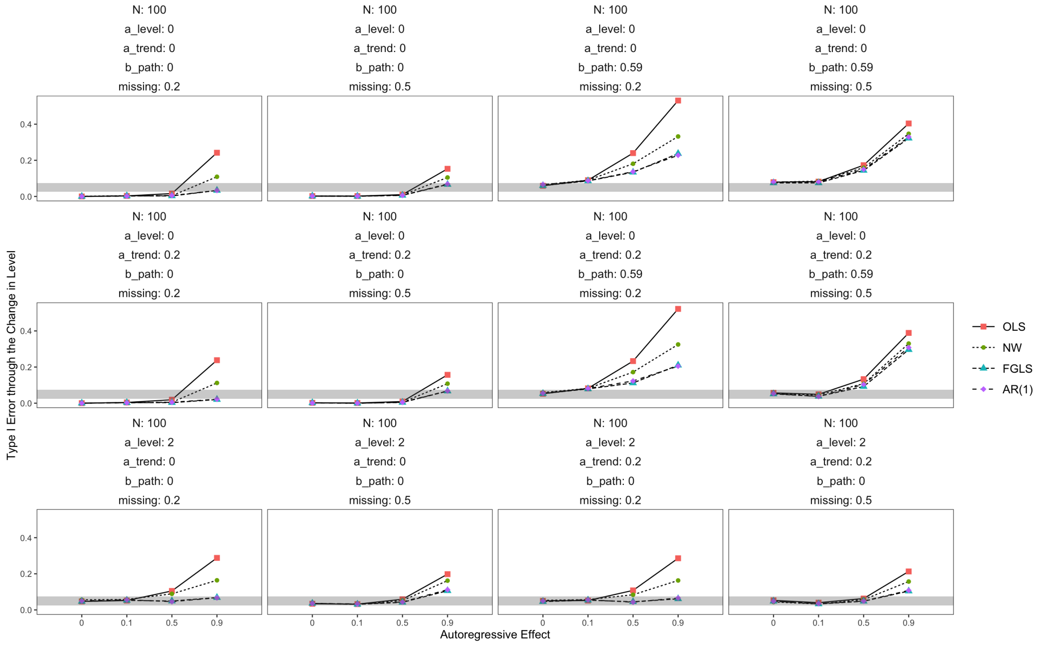

Note. Type I error rates of the interval estimate of the indirect effect defined as the change in level over 1,000 replications for different proportions of missing data. The shaded area represents the acceptable range of Type I error rates between 0.025 and 0.075. a_level = *a* path as the change in level. a_trend = *a* path as the change in trend. b_path = *b* path. N = sample size. missing = percentage of missing data.

Figure S10

Type I Error of the Estimate of the Indirect Effect through the Change in Trend

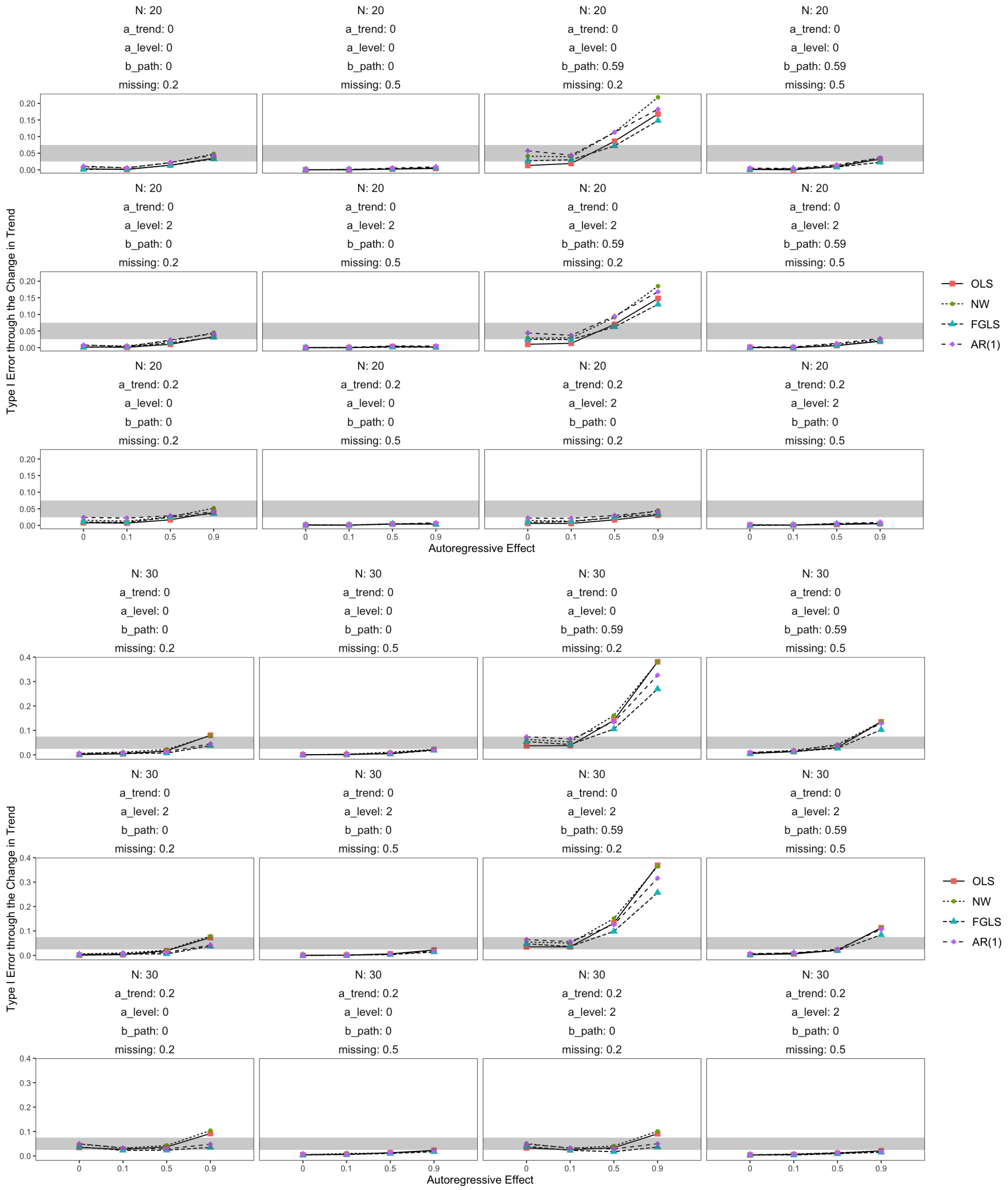

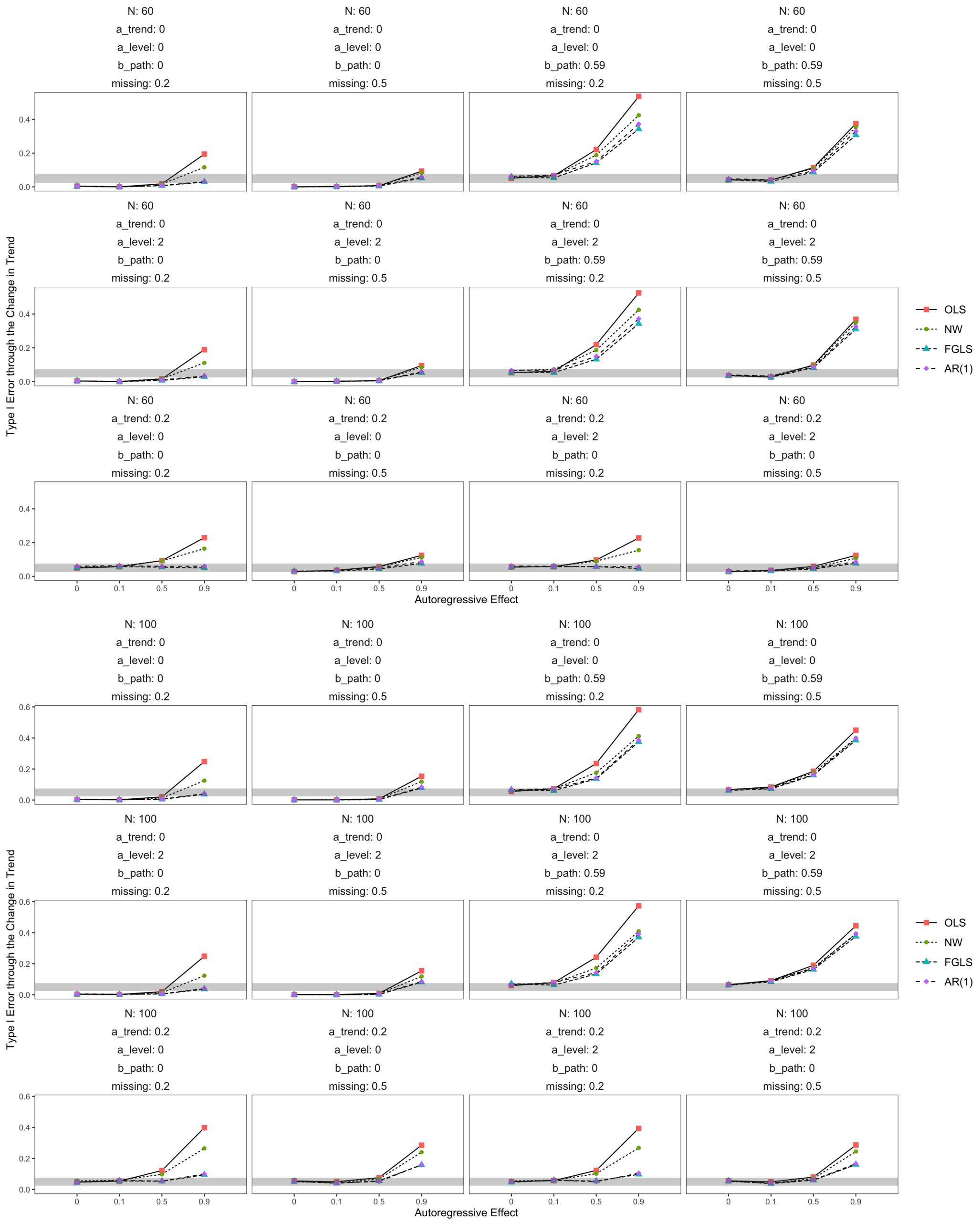

Note. Type I error rates of the interval estimate of the indirect effect defined as the change in trend over 1,000 replications for different proportions of missing data. The shaded area represents the acceptable range of Type I error rates between 0.025 and 0.075. a_level = *a* path as the change in level. a_trend = *a* path as the change in trend. b_path = *b* path. N = sample size. missing = percentage of missing data.

Figure S11

Coverage of the Indirect Effect through the Change in Level

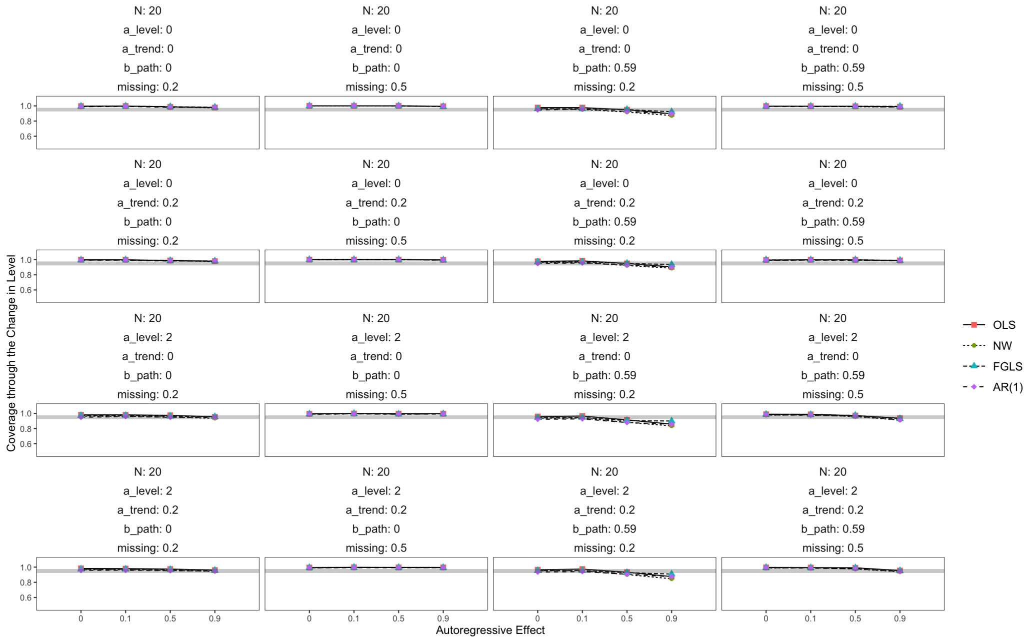

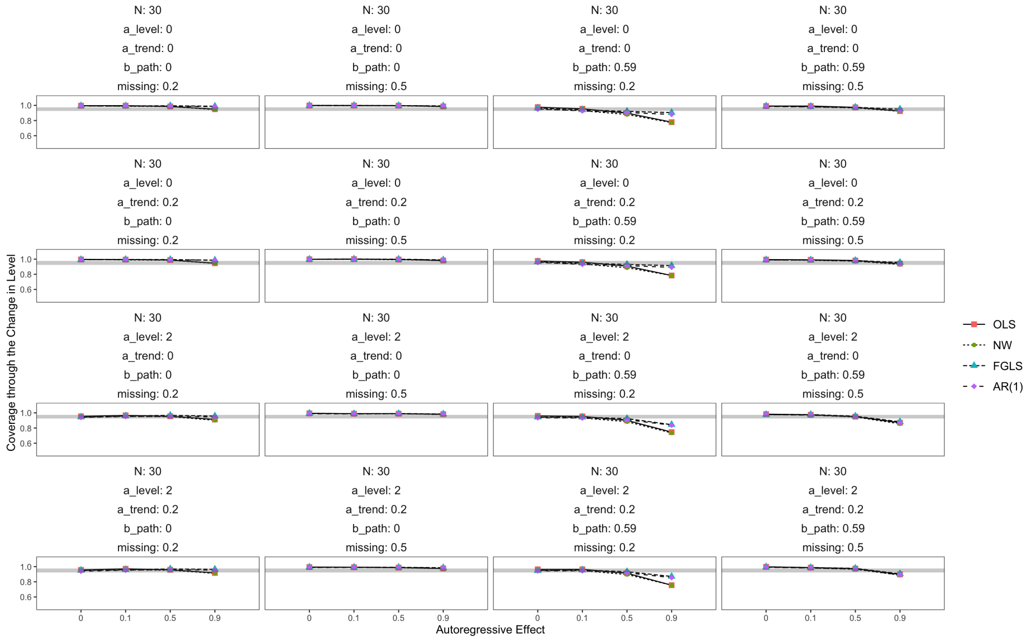

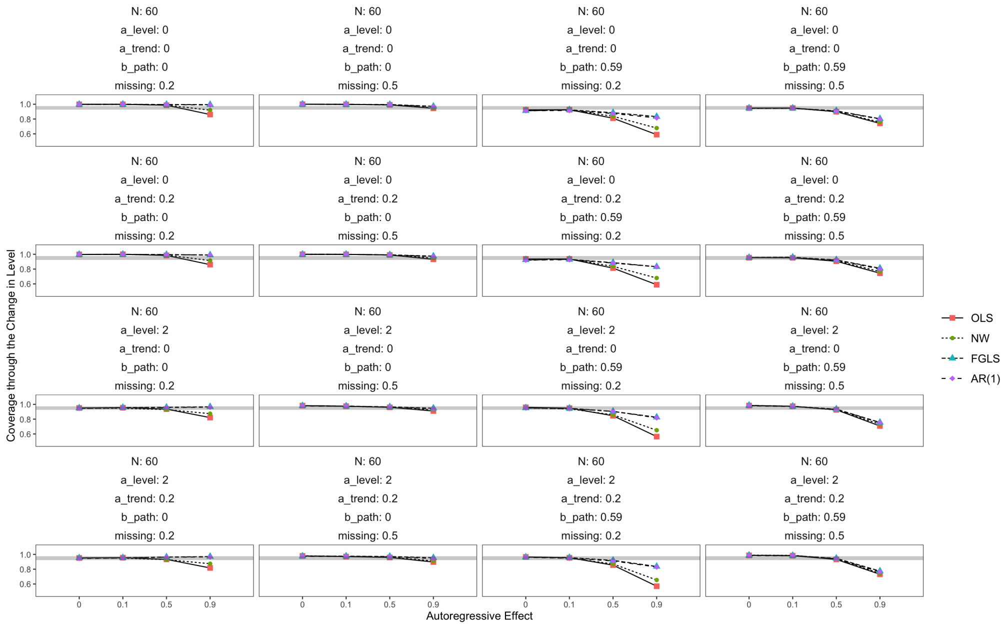

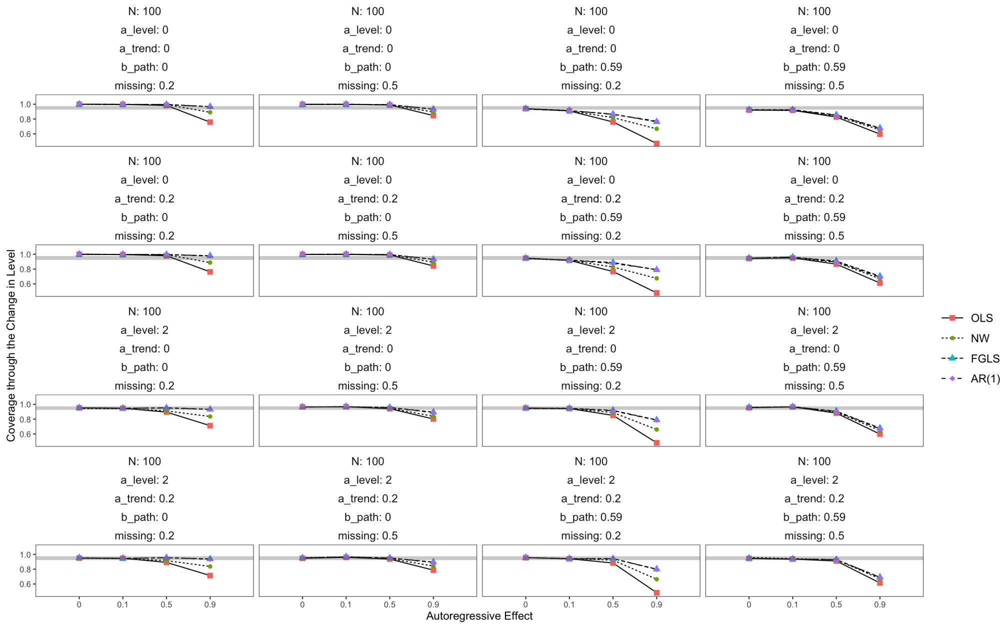

Note. Coverage of the interval estimate of the indirect effect defined through the change in level over 1,000 replications for different proportions of missing data. Shaded area represents values of acceptable coverage between 0.925 and 0.975. a_level = *a* path as the change in level. a_trend = *a* path as the change in trend. b_path = *b* path. N = sample size. missing = percentage of missing data.

Figure S12

Coverage of the Indirect Effect through the Change in Trend

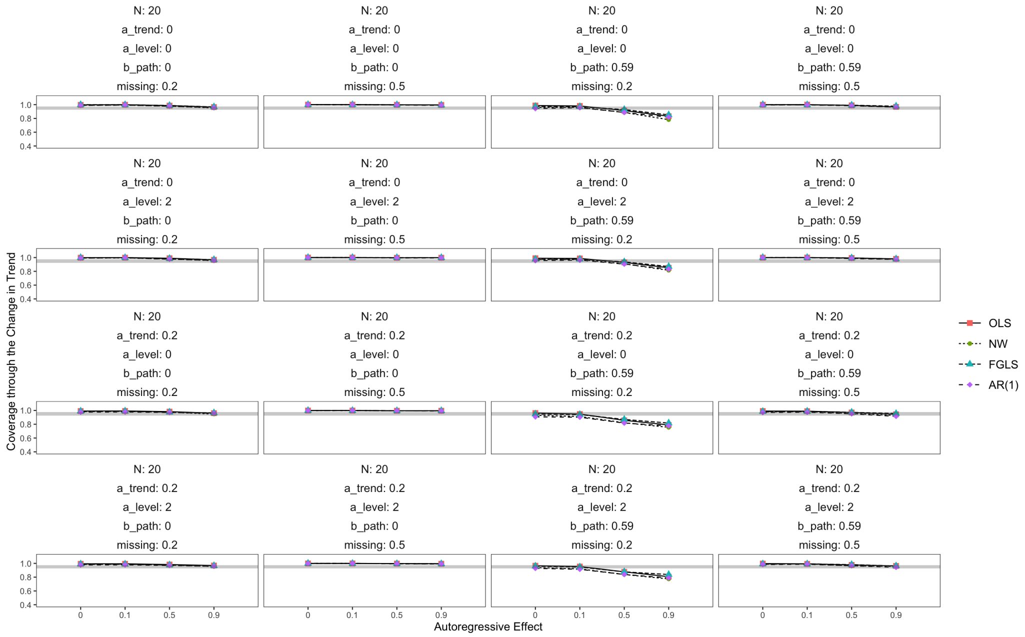

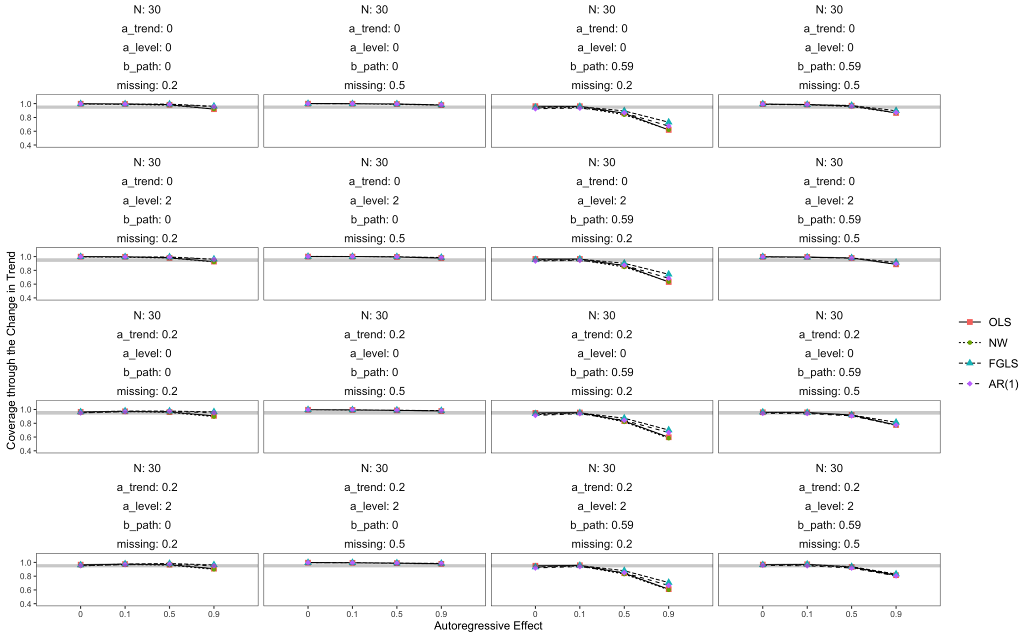

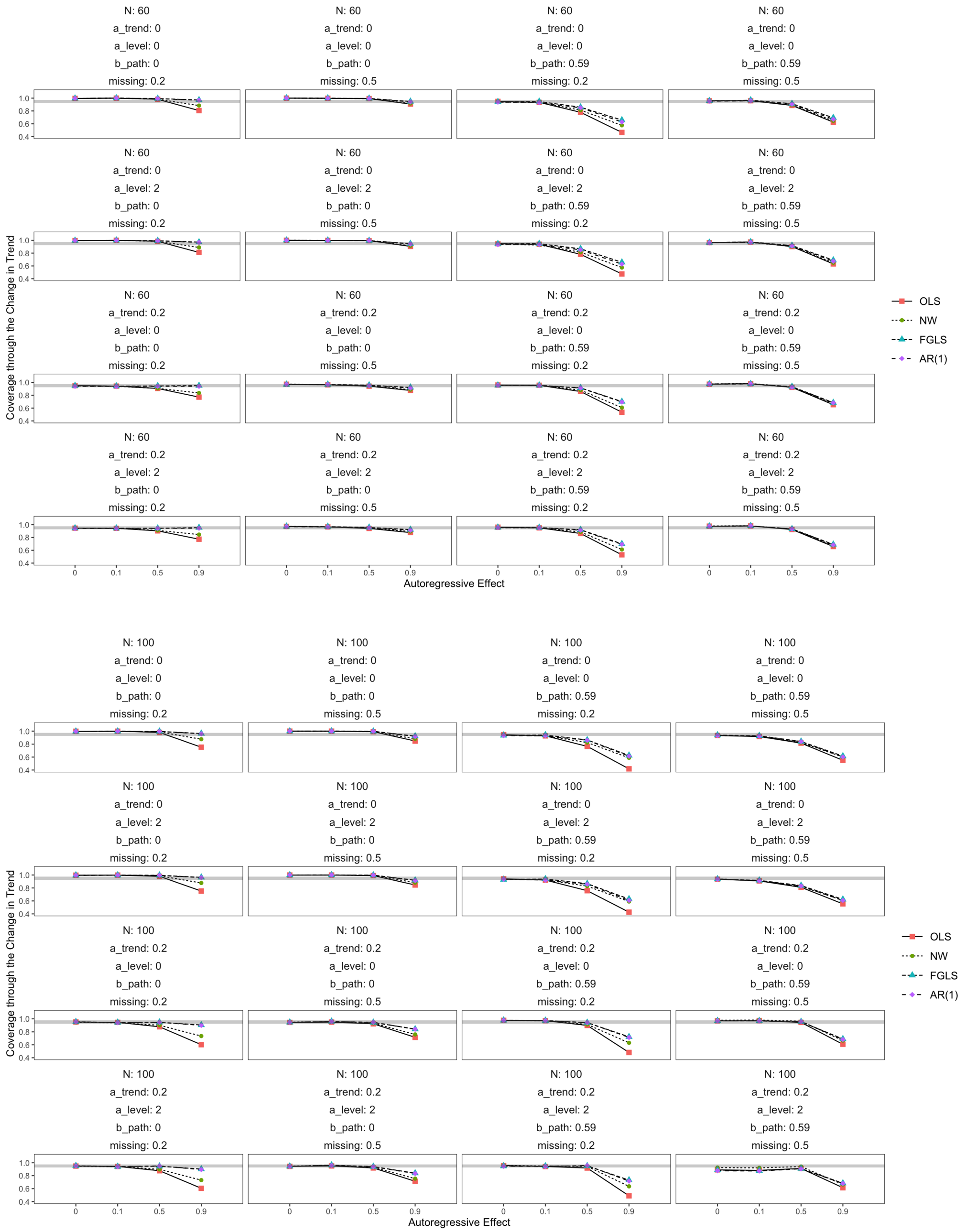

Note. Coverage of the interval estimate of the indirect effect defined through the change in trend over 1,000 replications for different proportions of missing data. Shaded area represents values of acceptable coverage between 0.925 and 0.975. a_level = *a* path as the change in level. a_trend = *a* path as the change in trend. b_path = *b* path. N = sample size. missing = percentage of missing data.

Figure S13

Interval Width of the Indirect Effect through the Change in Level

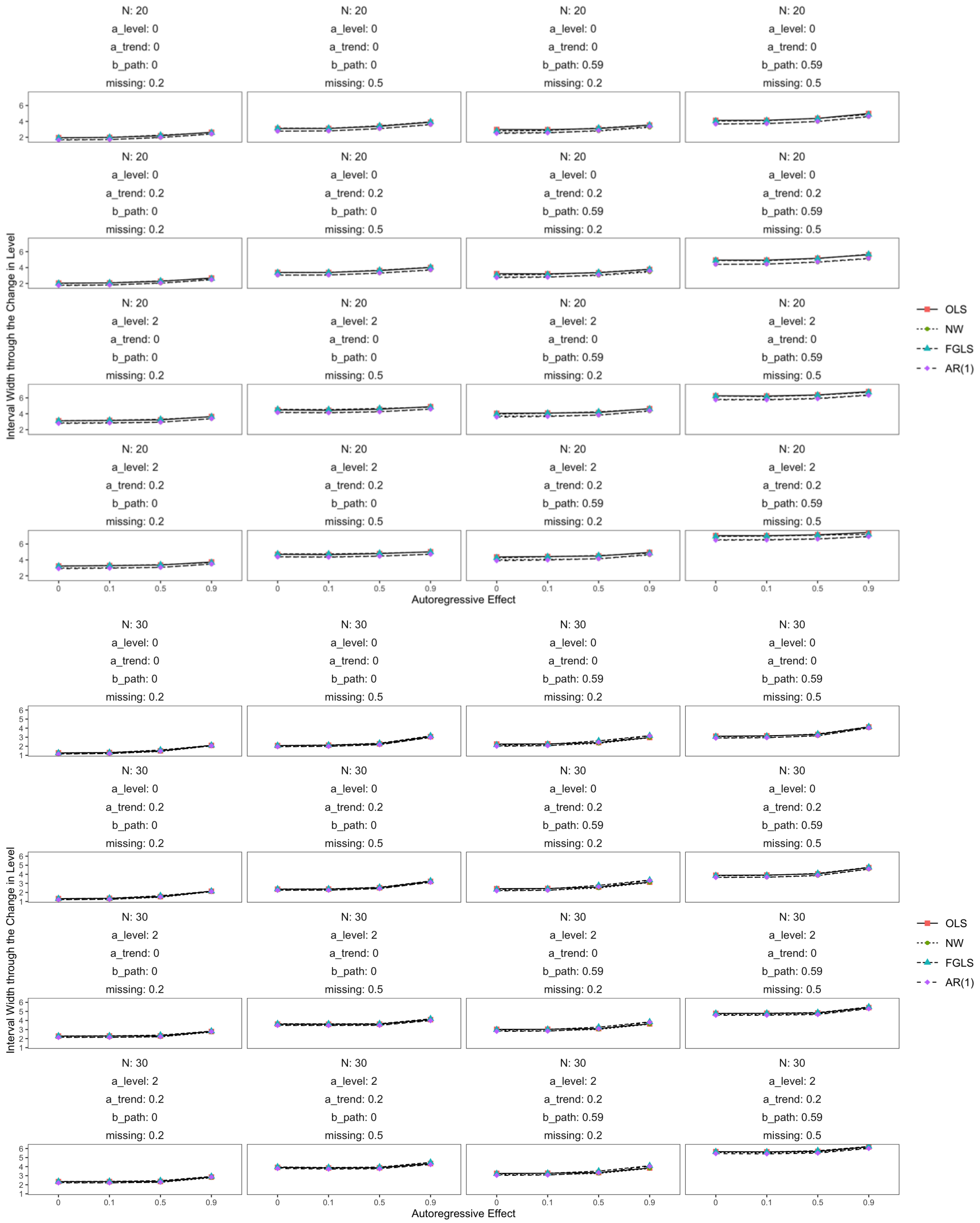

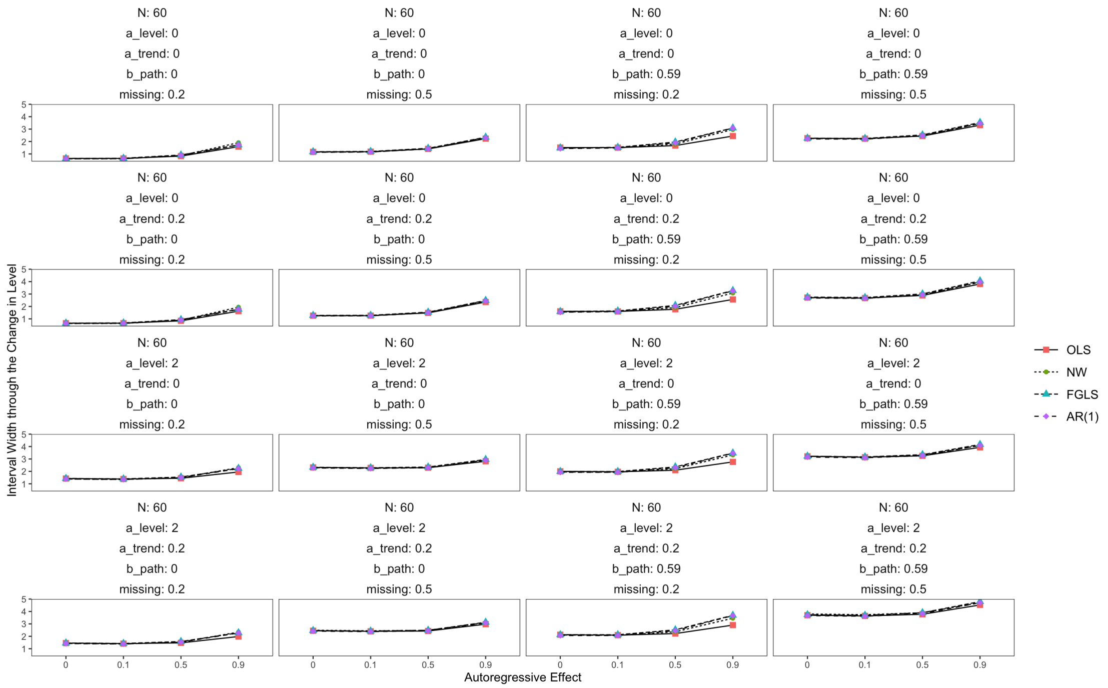

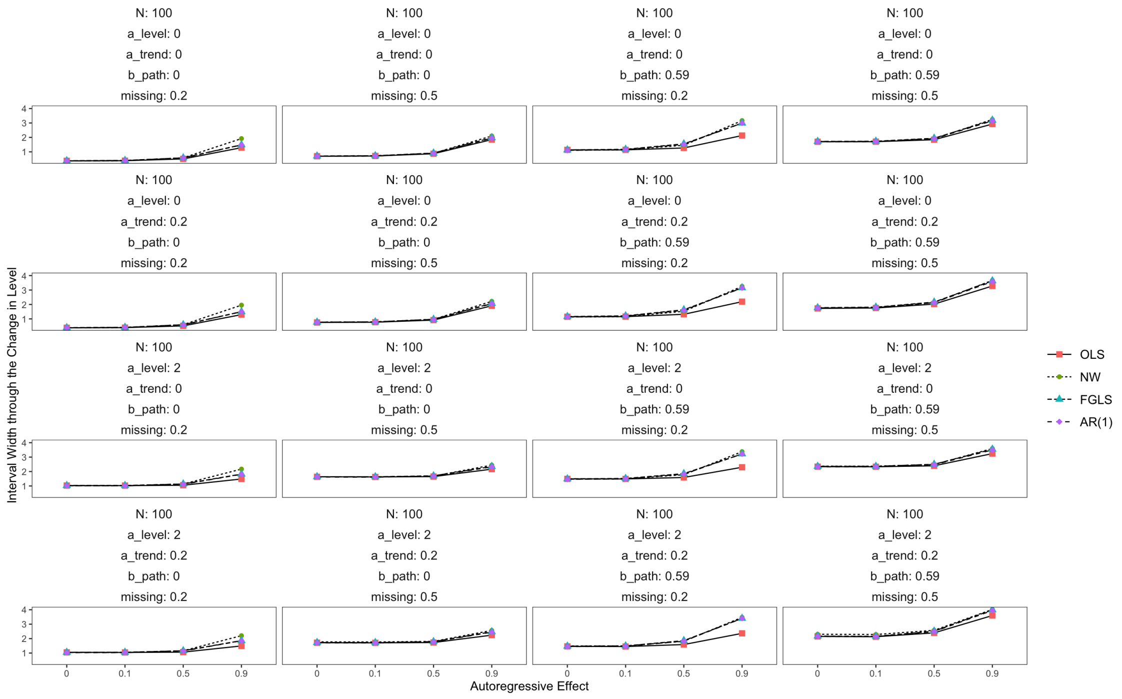

Note. Interval width of the indirect effect defined through the change in level over 1,000 replications for different proportions of missing data. a_level = *a* path as the change in level. a_trend = *a* path as the change in trend. b_path = *b* path. N = sample size. missing = percentage of missing data.

Figure S14

Interval Width of the Indirect Effect through the Change in Trend

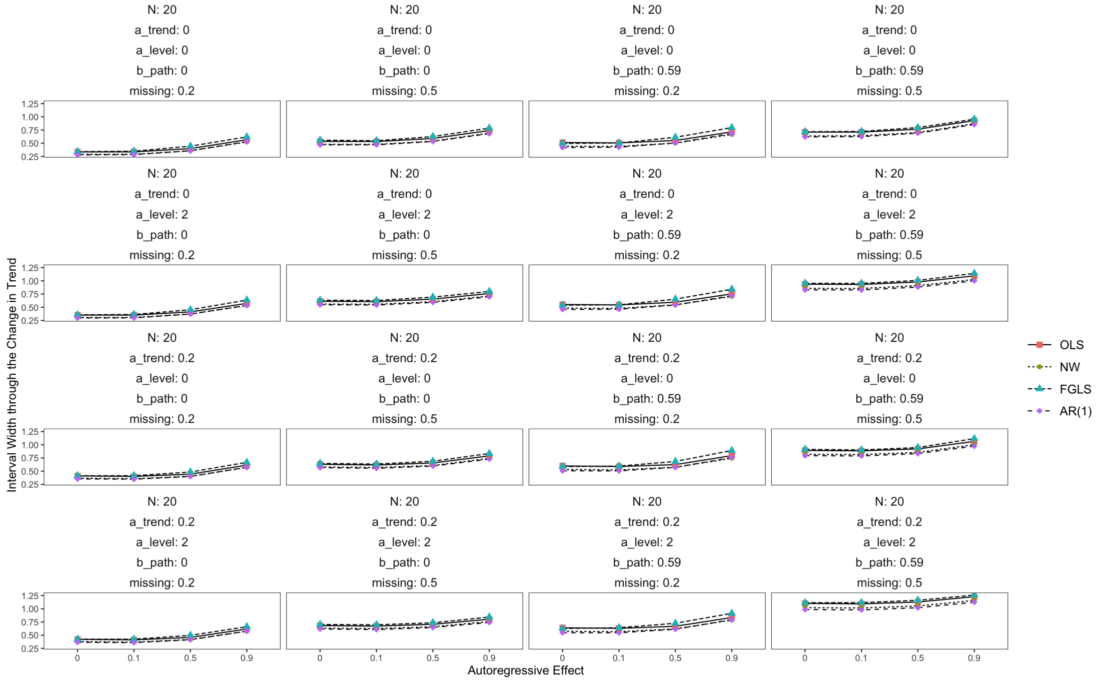

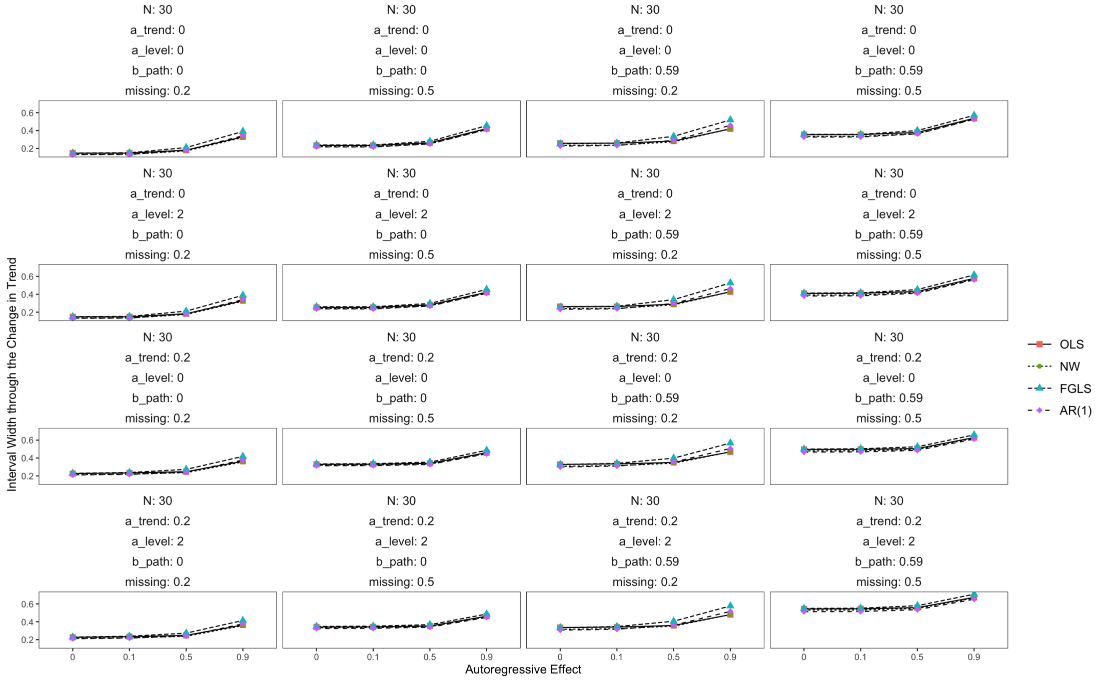

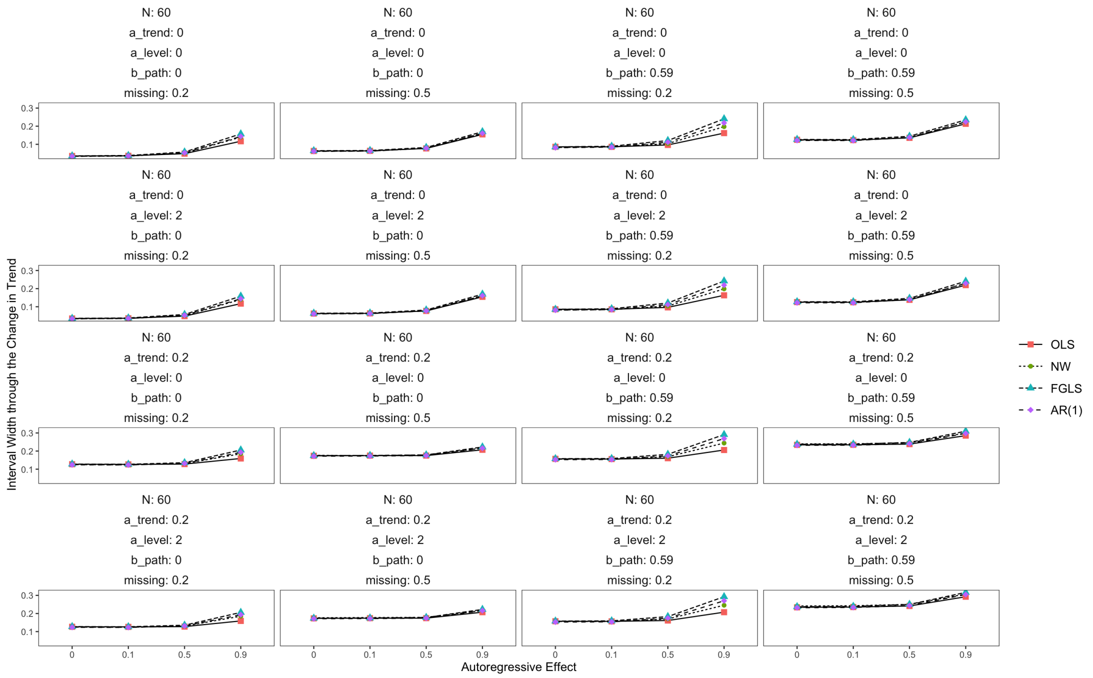

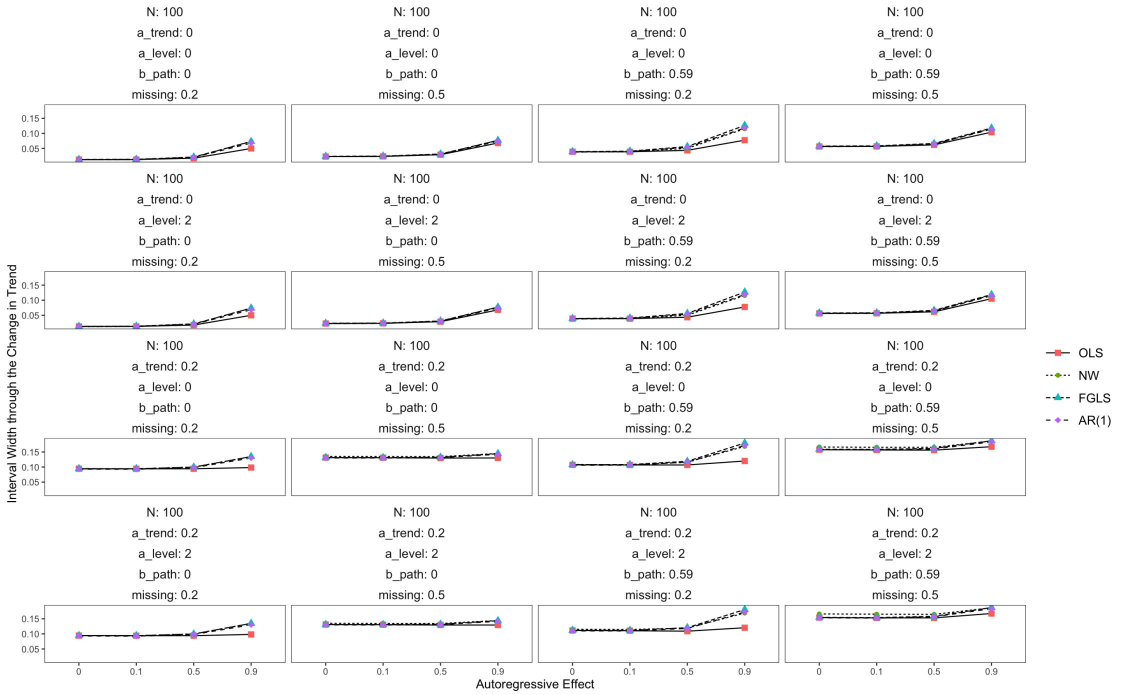

Note. Interval width of the indirect effect defined through the change in trend over 1,000 replications for different proportions of missing data. a_level = *a* path as the change in level. a_trend = *a* path as the change in trend. b_path = *b* path. N = sample size. missing = percentage of missing data.

Figures Comparing Three Levels of Missingness (0%, 20%, 50%)

Relative Bias of the Point Estimate through the Change in Level

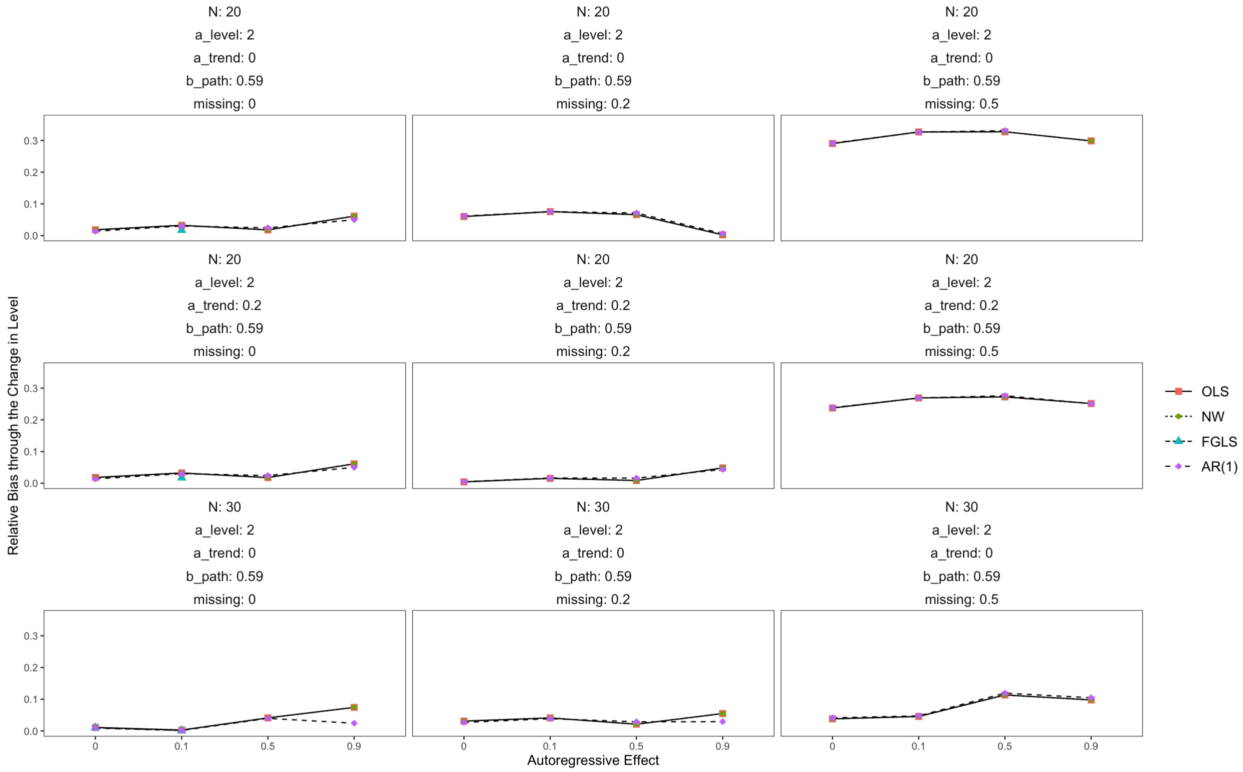

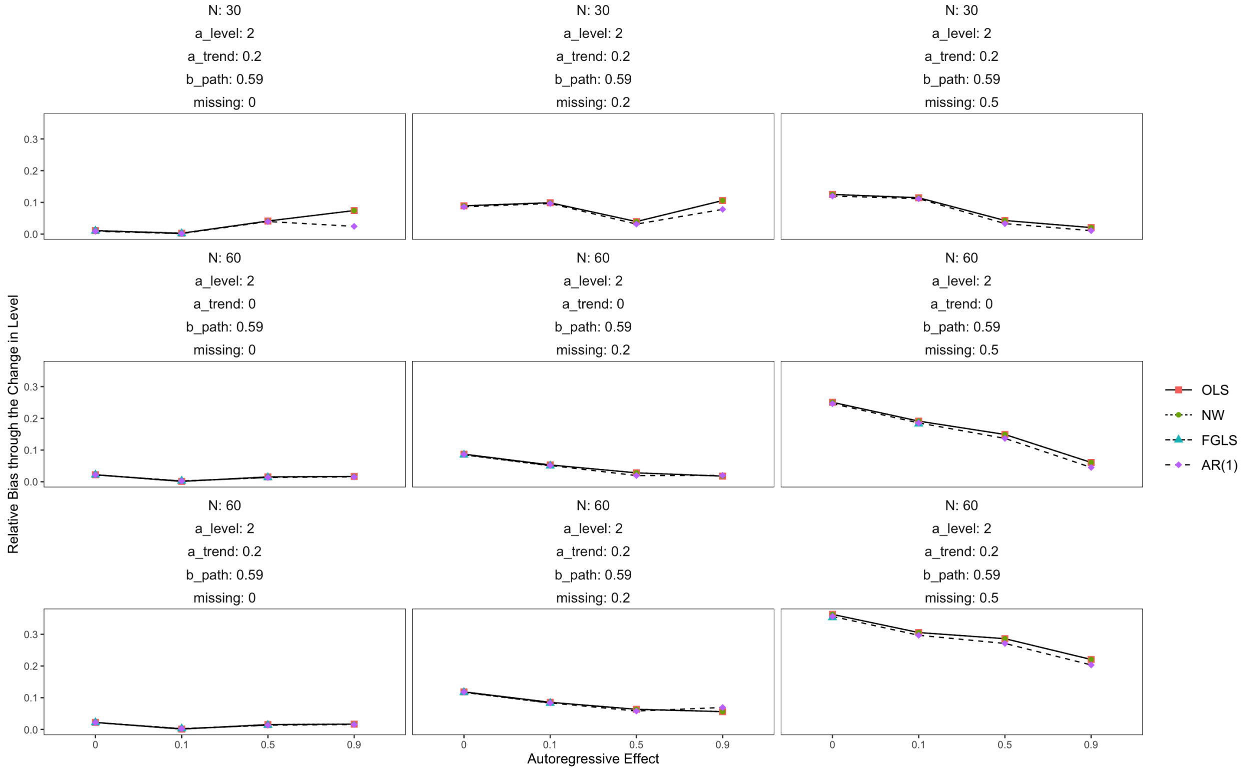

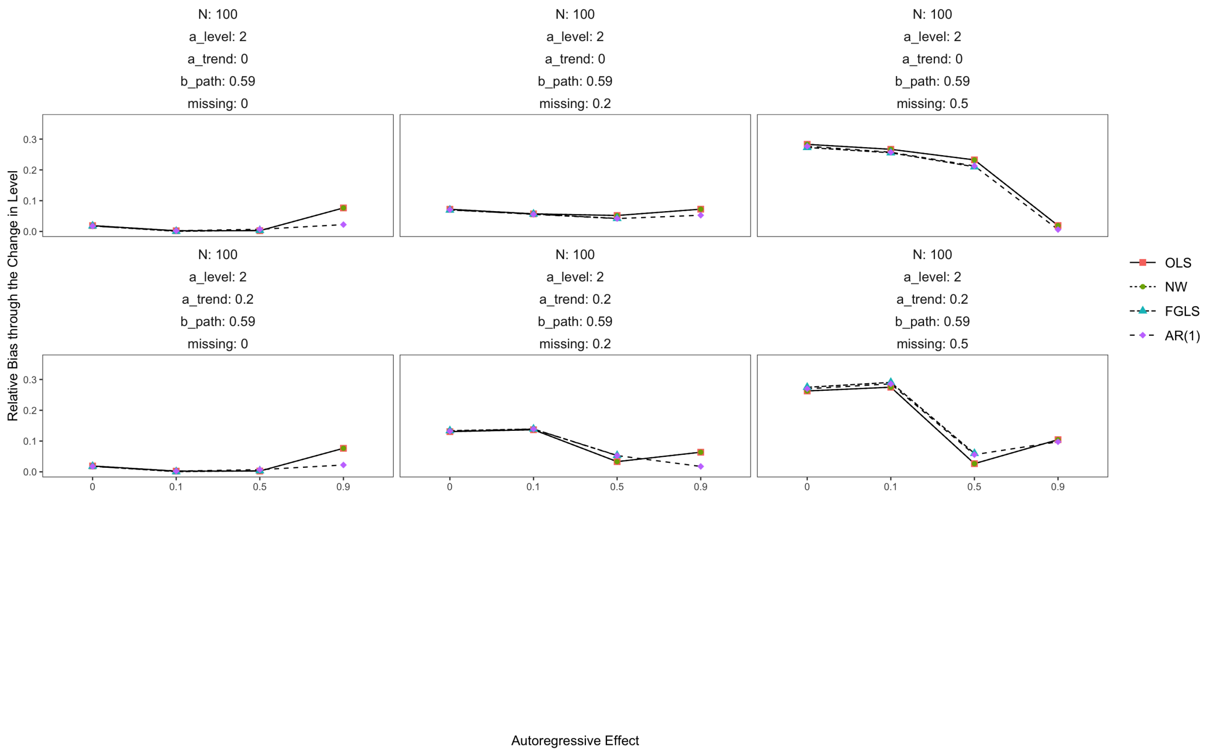

Relative Bias of the Point Estimate through the Change in Trend

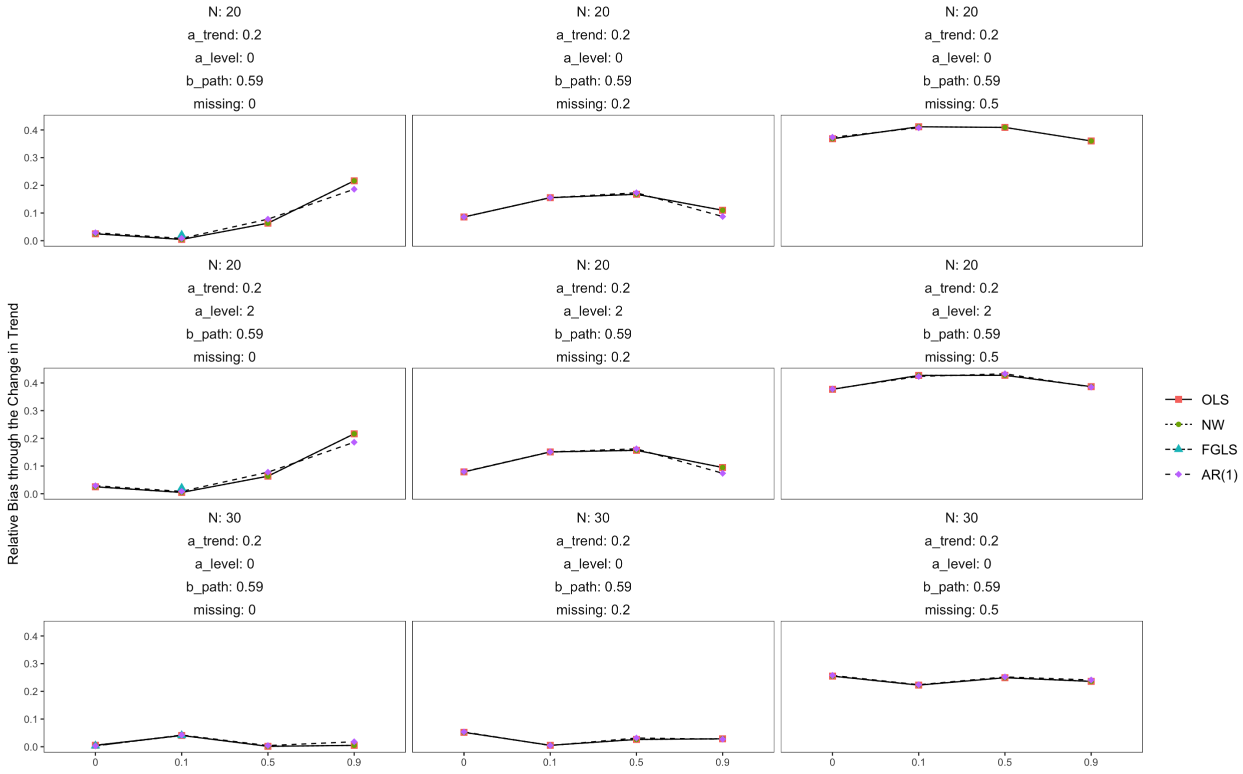

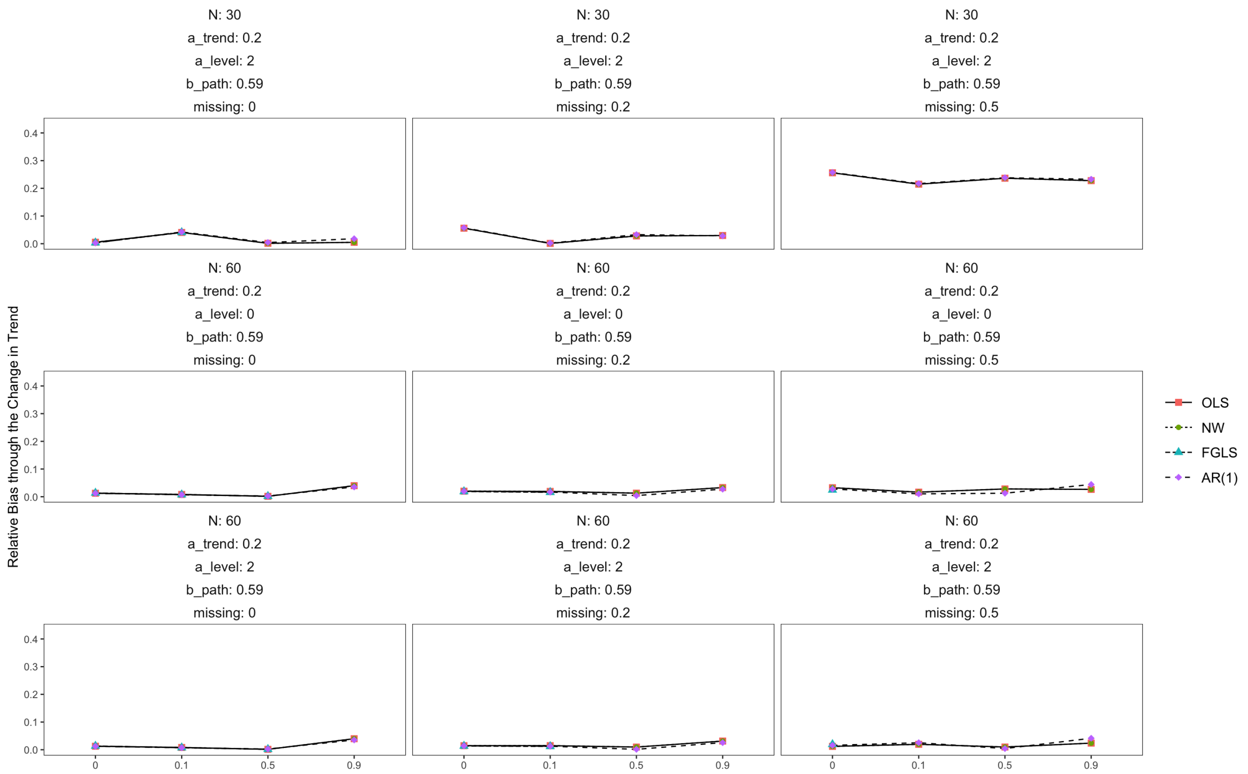
N: 30
a_trend: 0.2
a_level: 2
b_path: 0.59
missing: 0
N: 30
a_trend: 0.2
a_level: 2
b_path: 0.59
missing: 0.2
N: 30
a_trend: 0.2
a_level: 2
b_path: 0.59
missing: 0.5
N: 60
a_trend: 0.2
a_level: 0
b_path: 0.59
missing: 0
N: 60
a_trend: 0.2
a_level: 0
b_path: 0.59
missing: 0.2
N: 60
a_trend: 0.2
a_level: 0
b_path: 0.59
missing: 0.5
N: 60
a_trend: 0.2
a_level: 2
b_path: 0.59
missing: 0
N: 60
a_trend: 0.2
a_level: 2
b_path: 0.59
missing: 0.2
N: 60
a_trend: 0.2
a_level: 2
b_path: 0.59
missing: 0.5
Relative Bias through the Change in Trend
0.0
0.1
0.2
0.3
0.4
0
0.1
0.5
0.9
OLS
NW
FGLS
AR(1)

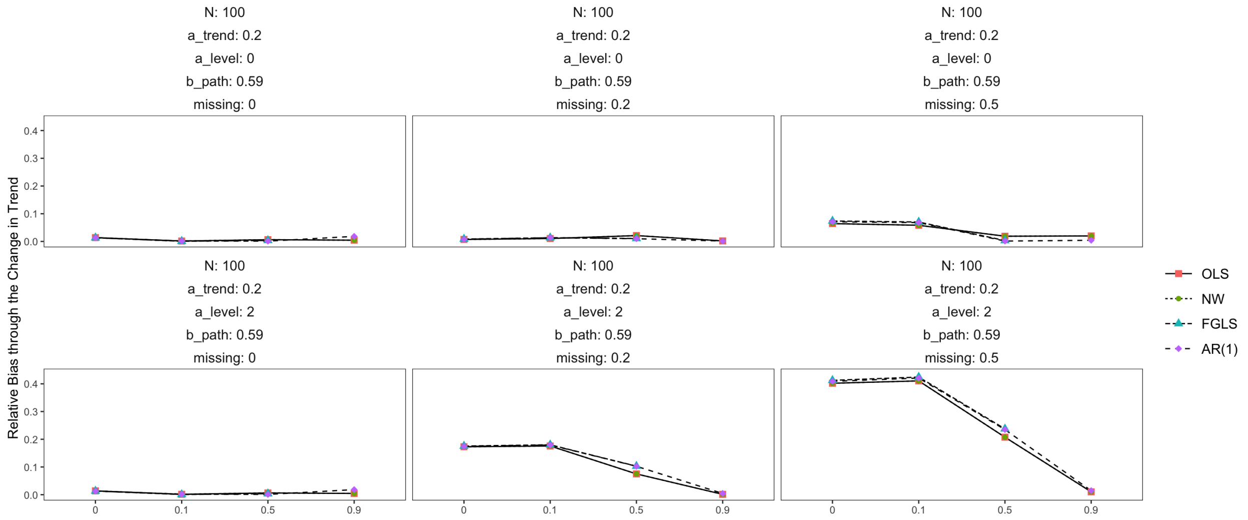
N: 100
a_trend: 0.2
a_level: 0
b_path: 0.59
missing: 0
N: 100
a_trend: 0.2
a_level: 0
b_path: 0.59
missing: 0.2
N: 100
a_trend: 0.2
a_level: 0
b_path: 0.59
missing: 0.5
N: 100
a_trend: 0.2
a_level: 2
b_path: 0.59
missing: 0
N: 100
a_trend: 0.2
a_level: 2
b_path: 0.59
missing: 0.2
N: 100
a_trend: 0.2
a_level: 2
b_path: 0.59
missing: 0.5
Relative Bias through the Change in Trend
0.0
0.1
0.2
0.3
0.4
0
0.1
0.5
0.9
OLS
NW
FGLS
AR(1)

Standard Deviation of the Indirect Effect through the Change in Level

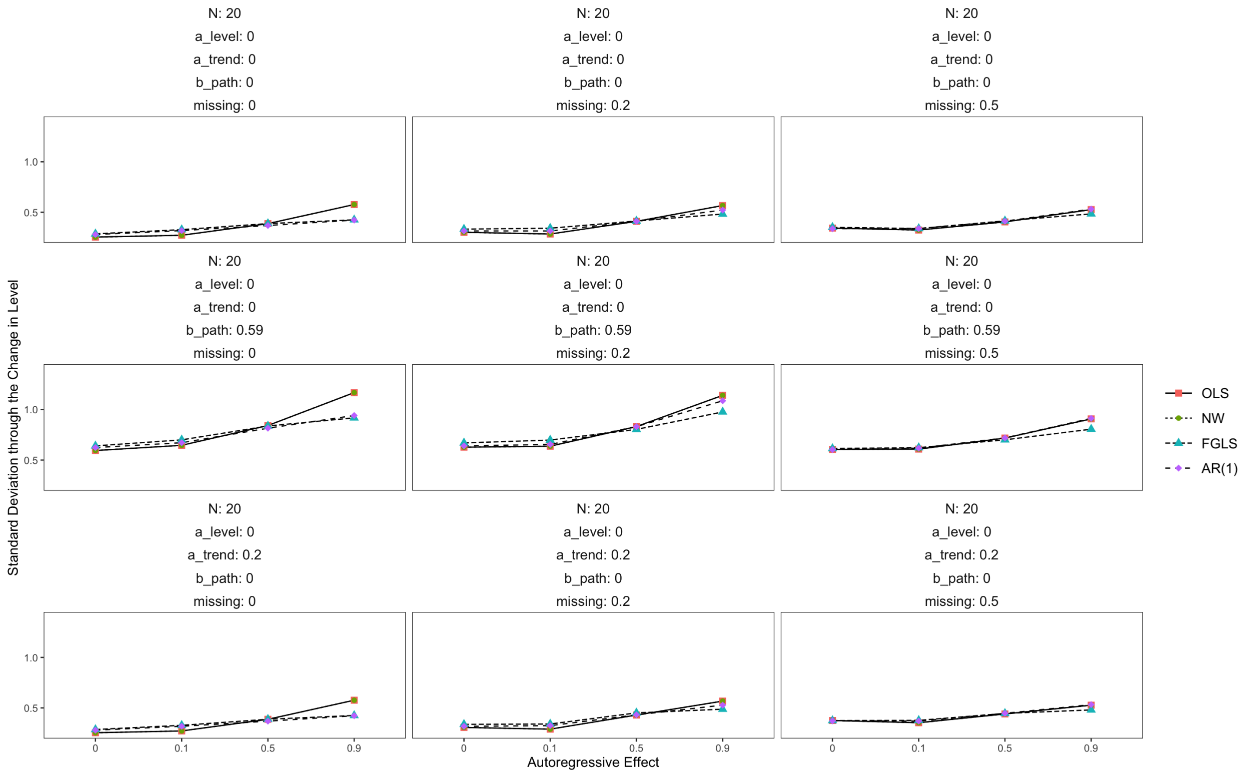

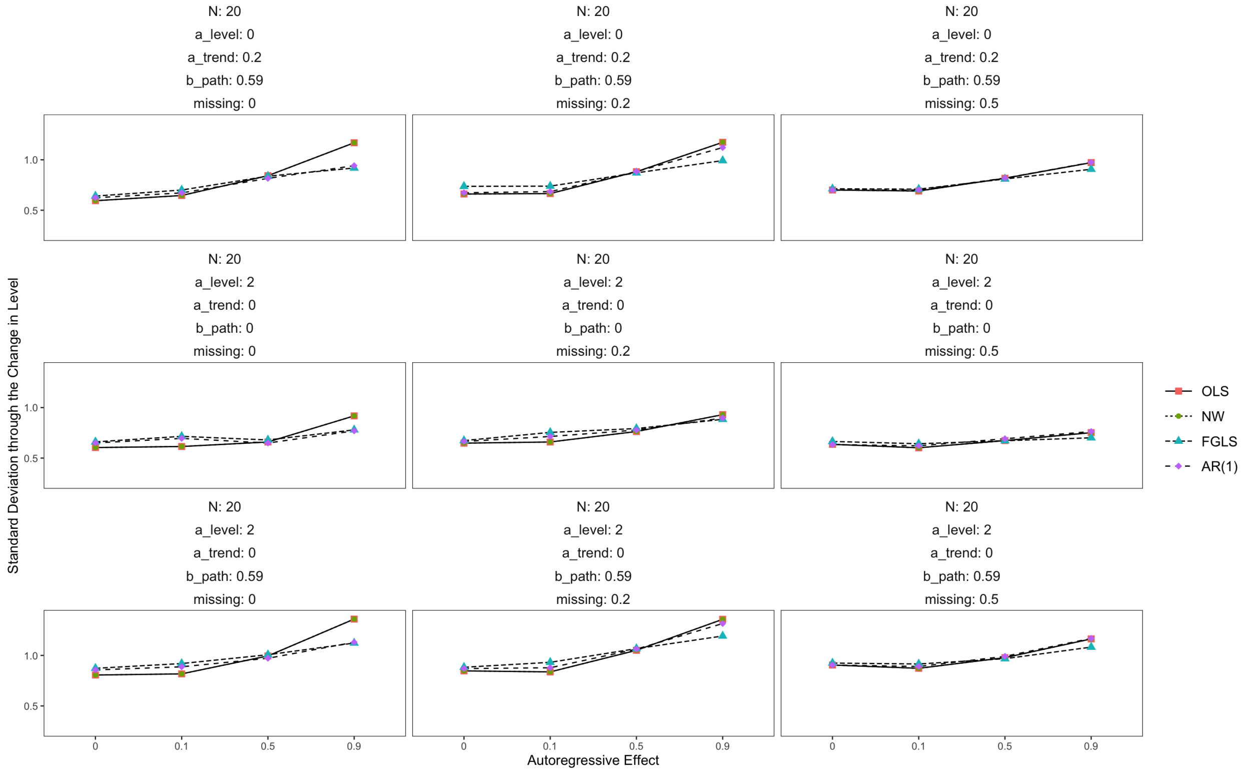

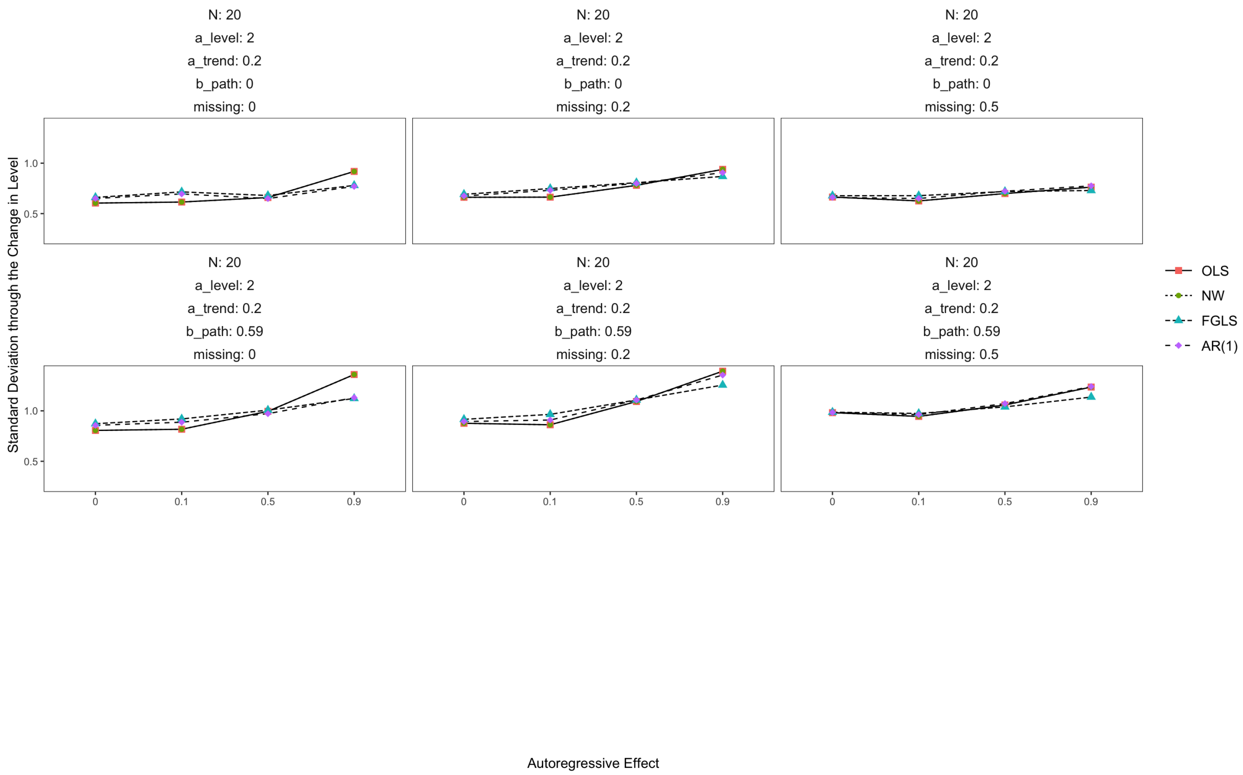
N: 20
a_level: 2
a_trend: 0.2
b_path: 0
missing: 0
N: 20
a_level: 2
a_trend: 0.2
b_path: 0
missing: 0.2
N: 20
a_level: 2
a_trend: 0.2
b_path: 0
missing: 0.5
N: 20
a_level: 2
a_trend: 0.2
b_path: 0.59
missing: 0
N: 20
a_level: 2
a_trend: 0.2
b_path: 0.59
missing: 0.2
N: 20
a_level: 2
a_trend: 0.2
b_path: 0.59
missing: 0.5
Standard Deviation through the Change in Level
1.0
0.5
0
0.1
0.5
0.9
OLS
NW
FGLS
AR(1)
Autoregressive Effect

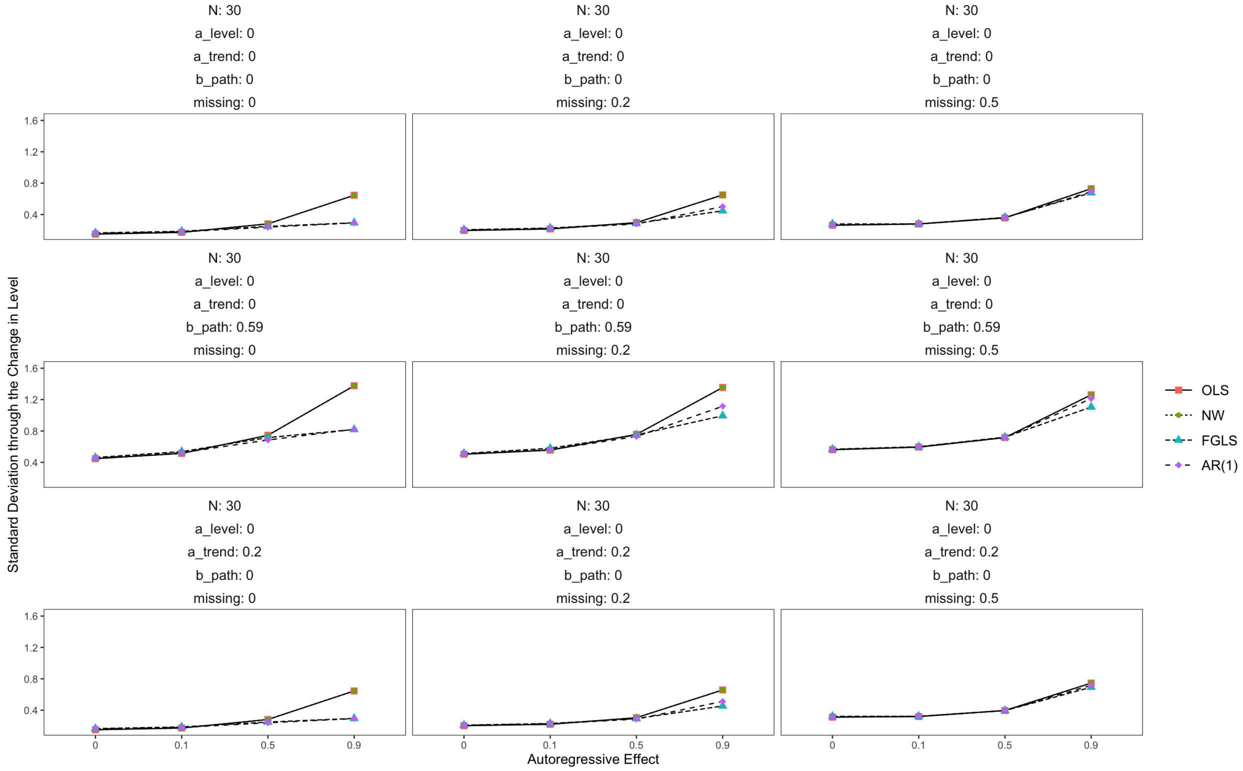
N: 30
a_level: 0
a_trend: 0
b_path: 0
missing: 0
N: 30
a_level: 0
a_trend: 0
b_path: 0
missing: 0.2
N: 30
a_level: 0
a_trend: 0
b_path: 0
missing: 0.5
N: 30
a_level: 0
a_trend: 0
b_path: 0.59
missing: 0
N: 30
a_level: 0
a_trend: 0
b_path: 0.59
missing: 0.2
N: 30
a_level: 0
a_trend: 0
b_path: 0.59
missing: 0.5
N: 30
a_level: 0
a_trend: 0.2
b_path: 0
missing: 0
N: 30
a_level: 0
a_trend: 0.2
b_path: 0
missing: 0.2
N: 30
a_level: 0
a_trend: 0.2
b_path: 0
missing: 0.5
Standard Deviation through the Change in Level
1.6
1.2
0.8
0.4
0
0.1
0.5
0.9
OLS
NW
FGLS
AR(1)
Autoregressive Effect

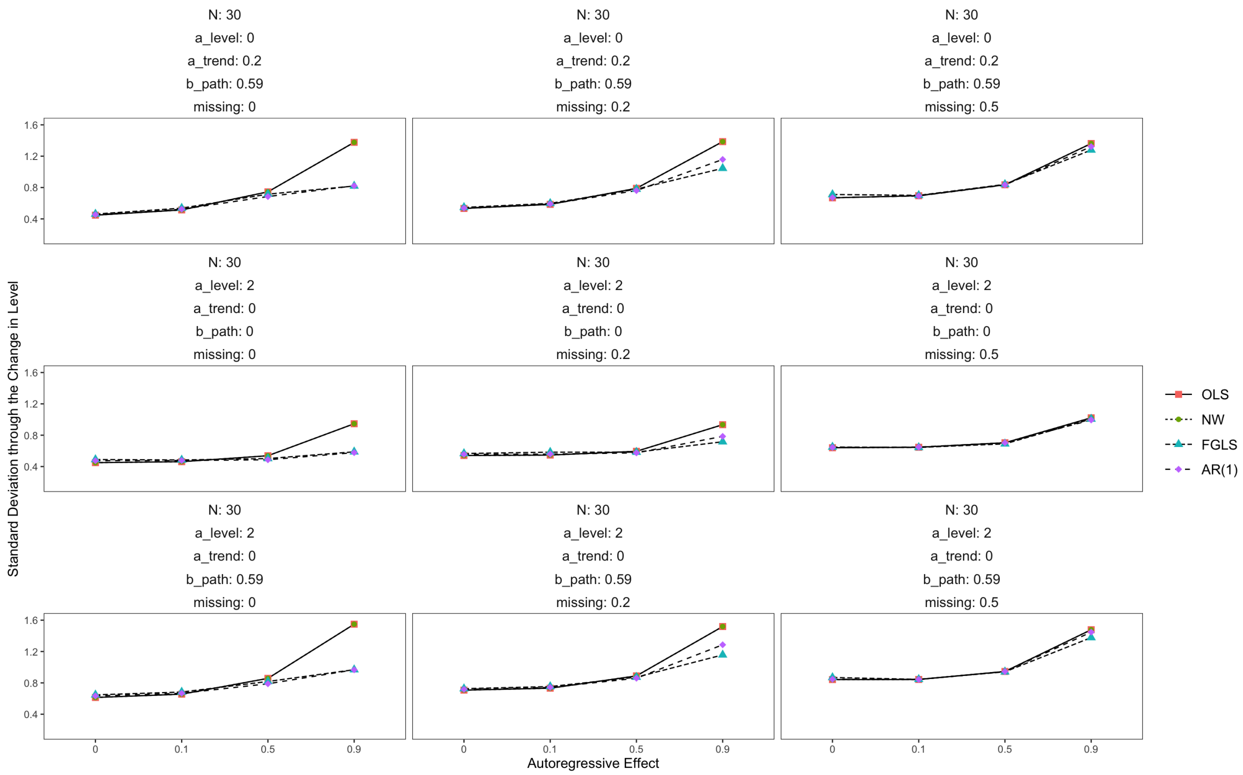
N: 30 a_level: 0 a_trend: 0.2 b_path: 0.59 missing: 0
N: 30 a_level: 0 a_trend: 0.2 b_path: 0.59 missing: 0.2
N: 30 a_level: 0 a_trend: 0.2 b_path: 0.59 missing: 0.5
N: 30 a_level: 2 a_trend: 0 b_path: 0 missing: 0
N: 30 a_level: 2 a_trend: 0 b_path: 0 missing: 0.2
N: 30 a_level: 2 a_trend: 0 b_path: 0 missing: 0.5
N: 30 a_level: 2 a_trend: 0 b_path: 0.59 missing: 0
N: 30 a_level: 2 a_trend: 0 b_path: 0.59 missing: 0.2
N: 30 a_level: 2 a_trend: 0 b_path: 0.59 missing: 0.5
1.6
1.2
0.8
0.4
0
0.1
0.5
0.9
Standard Deviation through the Change in Level
Autoregressive Effect
OLS
NW
FGLS
AR(1)

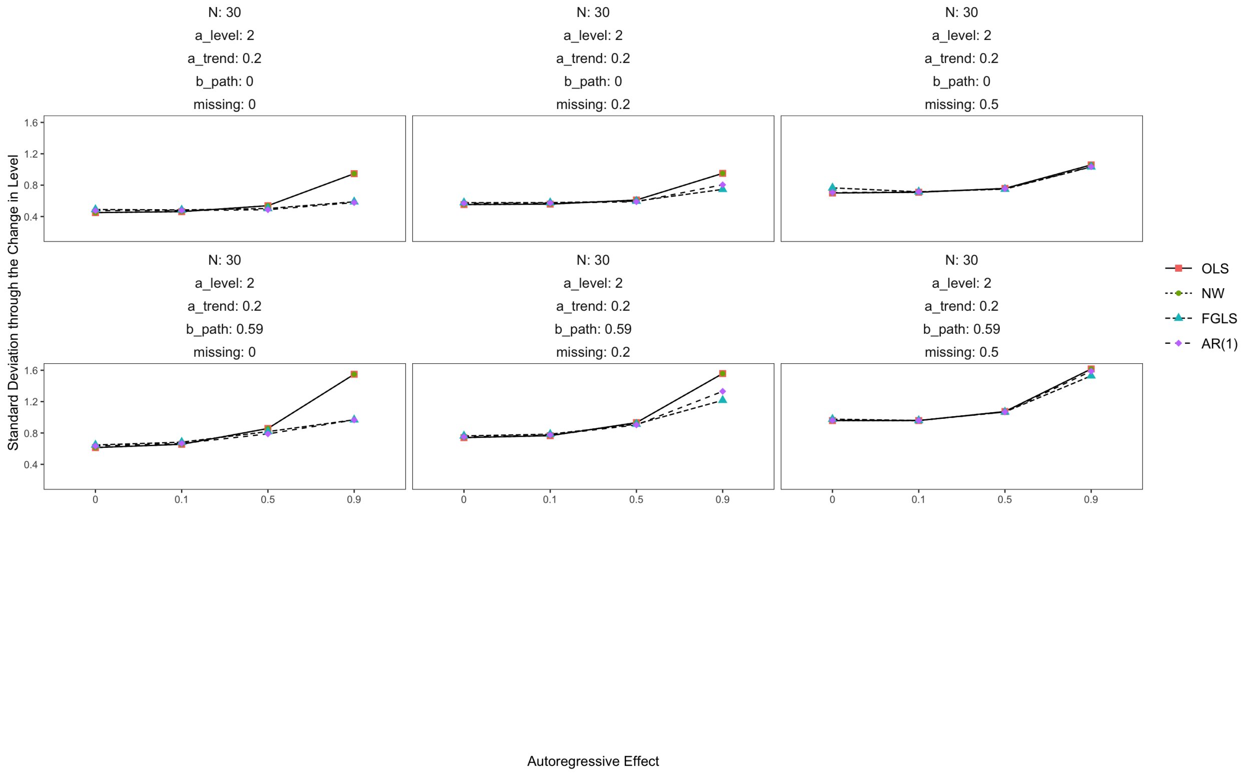
N: 30 a_level: 2 a_trend: 0.2 b_path: 0 missing: 0
N: 30 a_level: 2 a_trend: 0.2 b_path: 0 missing: 0.2
N: 30 a_level: 2 a_trend: 0.2 b_path: 0 missing: 0.5
N: 30 a_level: 2 a_trend: 0.2 b_path: 0.59 missing: 0
N: 30 a_level: 2 a_trend: 0.2 b_path: 0.59 missing: 0.2
N: 30 a_level: 2 a_trend: 0.2 b_path: 0.59 missing: 0.5
1.6
1.2
0.8
0.4
0
0.1
0.5
0.9
Standard Deviation through the Change in Level
Autoregressive Effect
OLS
NW
FGLS
AR(1)

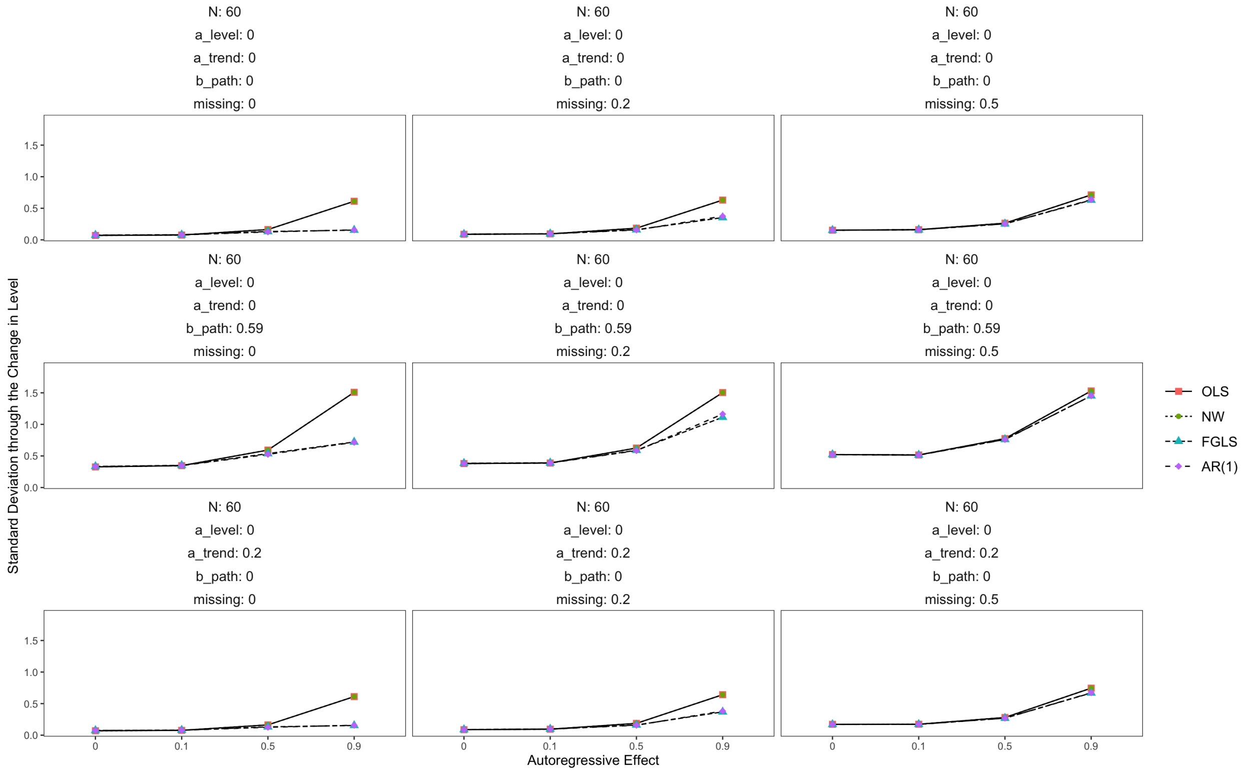
N: 60
a_level: 0
a_trend: 0
b_path: 0
missing: 0
N: 60
a_level: 0
a_trend: 0
b_path: 0
missing: 0.2
N: 60
a_level: 0
a_trend: 0
b_path: 0
missing: 0.5
N: 60
a_level: 0
a_trend: 0
b_path: 0.59
missing: 0
N: 60
a_level: 0
a_trend: 0
b_path: 0.59
missing: 0.2
N: 60
a_level: 0
a_trend: 0
b_path: 0.59
missing: 0.5
N: 60
a_level: 0
a_trend: 0.2
b_path: 0
missing: 0
N: 60
a_level: 0
a_trend: 0.2
b_path: 0
missing: 0.2
N: 60
a_level: 0
a_trend: 0.2
b_path: 0
missing: 0.5
Standard Deviation through the Change in Level
Autoregressive Effect
OLS
NW
FGLS
AR(1)

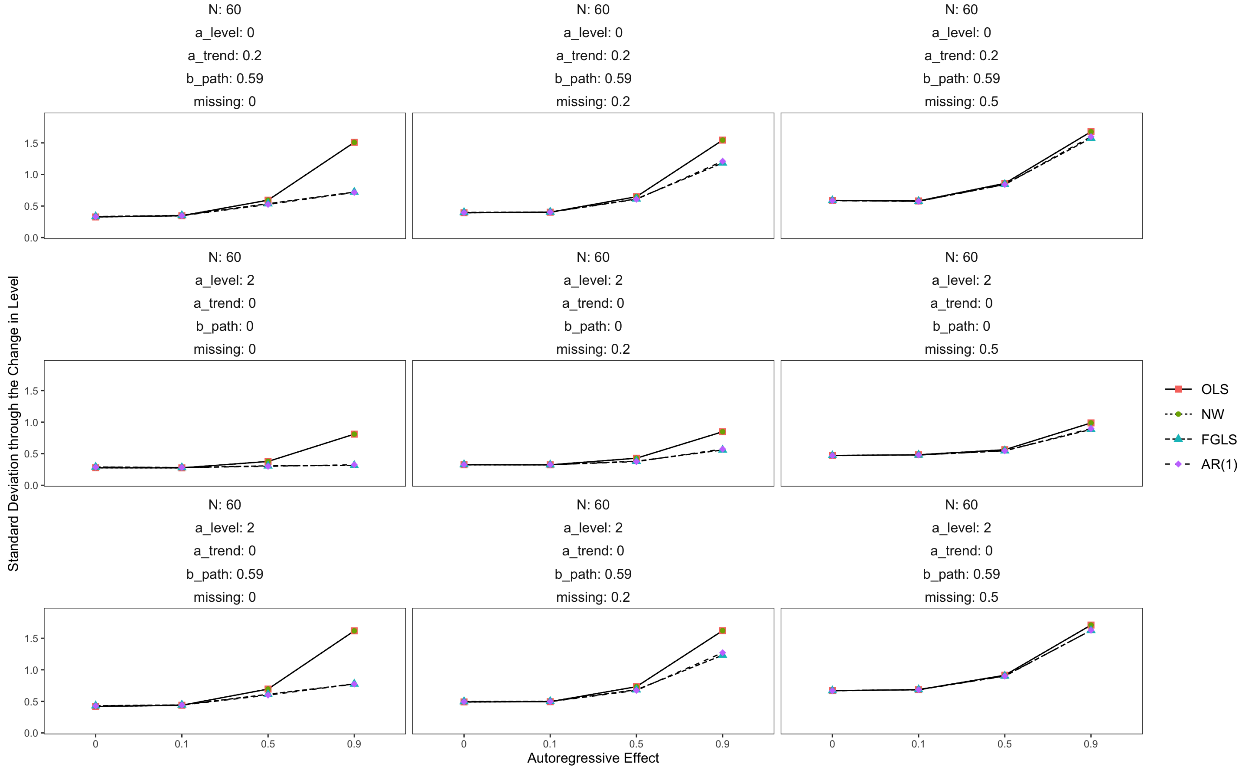
N: 60
a_level: 0
a_trend: 0.2
b_path: 0.59
missing: 0
N: 60
a_level: 0
a_trend: 0.2
b_path: 0.59
missing: 0.2
N: 60
a_level: 0
a_trend: 0.2
b_path: 0.59
missing: 0.5
N: 60
a_level: 2
a_trend: 0
b_path: 0
missing: 0
N: 60
a_level: 2
a_trend: 0
b_path: 0
missing: 0.2
N: 60
a_level: 2
a_trend: 0
b_path: 0
missing: 0.5
N: 60
a_level: 2
a_trend: 0
b_path: 0.59
missing: 0
N: 60
a_level: 2
a_trend: 0
b_path: 0.59
missing: 0.2
N: 60
a_level: 2
a_trend: 0
b_path: 0.59
missing: 0.5
Standard Deviation through the Change in Level
Autoregressive Effect
OLS
NW
FGLS
AR(1)

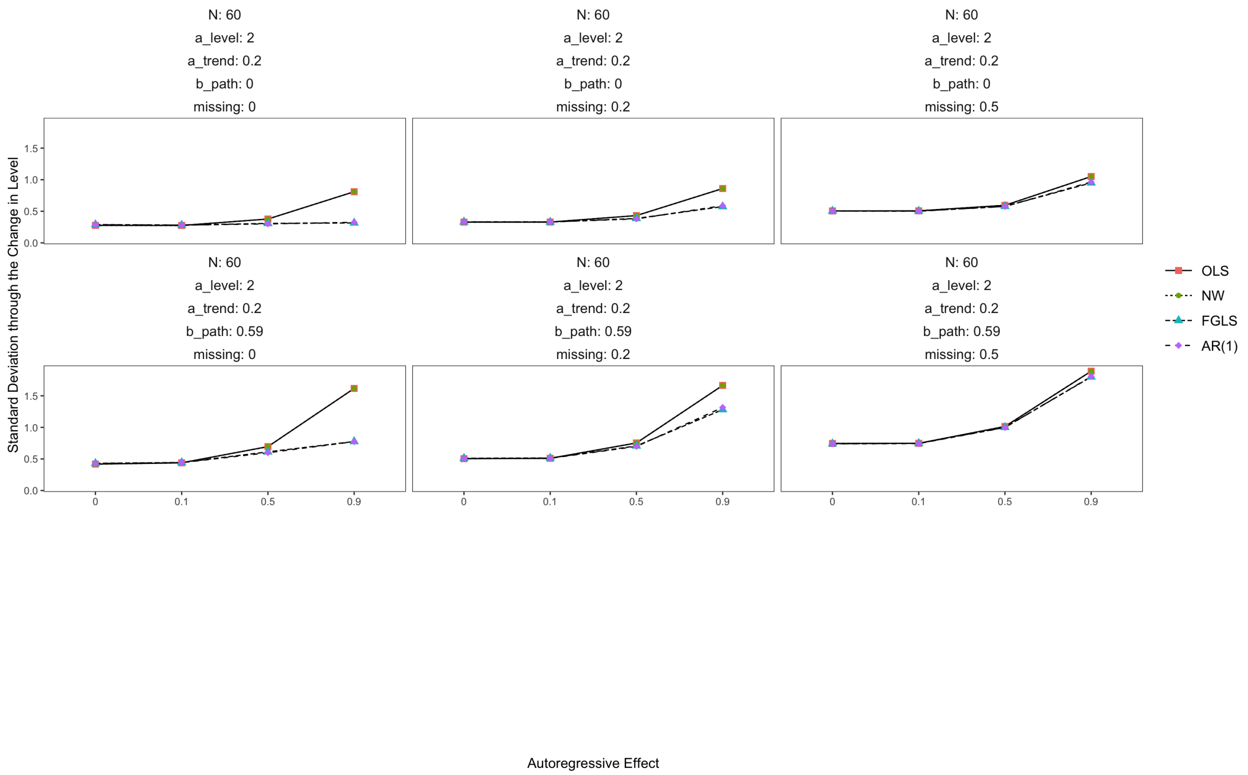
N: 60
a_level: 2
a_trend: 0.2
b_path: 0
missing: 0
N: 60
a_level: 2
a_trend: 0.2
b_path: 0
missing: 0.2
N: 60
a_level: 2
a_trend: 0.2
b_path: 0
missing: 0.5
N: 60
a_level: 2
a_trend: 0.2
b_path: 0.59
missing: 0
N: 60
a_level: 2
a_trend: 0.2
b_path: 0.59
missing: 0.2
N: 60
a_level: 2
a_trend: 0.2
b_path: 0.59
missing: 0.5
Standard Deviation through the Change in Level
0.0
0.5
1.0
1.5
0
0.1
0.5
0.9
OLS
NW
FGLS
AR(1)
Autoregressive Effect

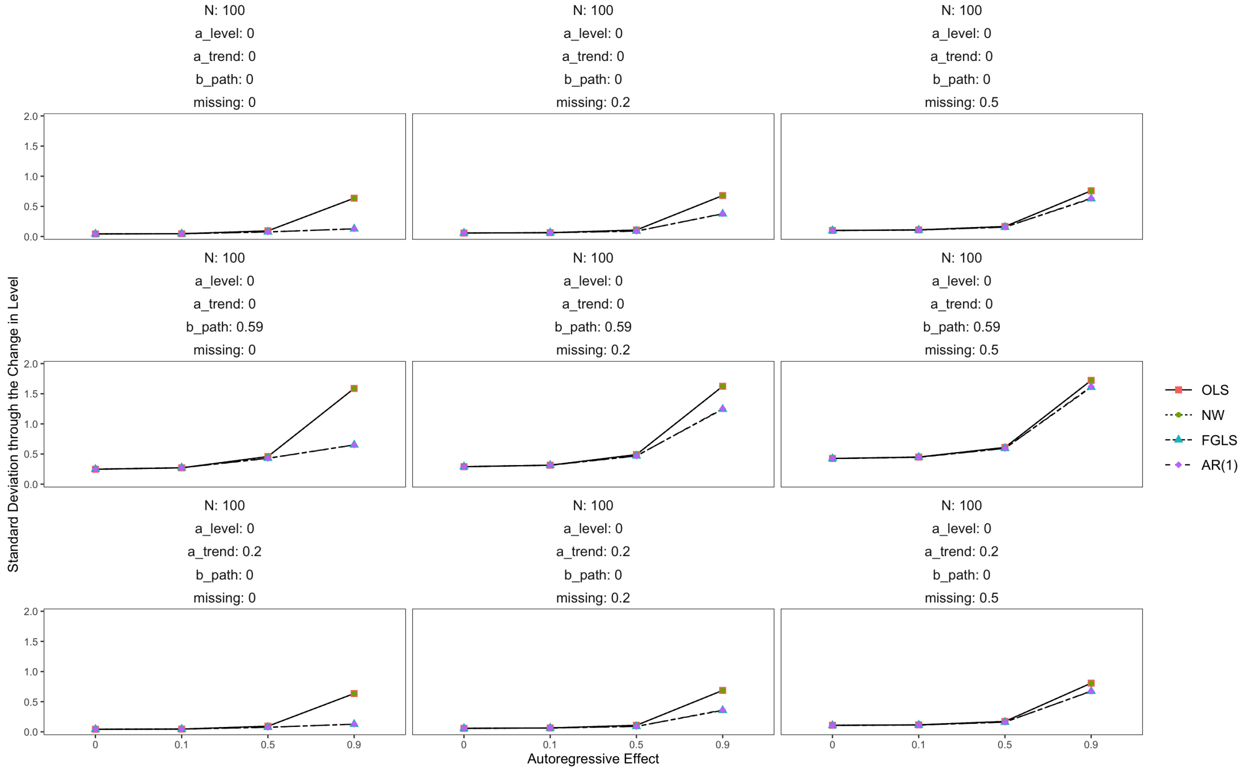
N: 100
a_level: 0
a_trend: 0
b_path: 0
missing: 0
N: 100
a_level: 0
a_trend: 0
b_path: 0
missing: 0.2
N: 100
a_level: 0
a_trend: 0
b_path: 0
missing: 0.5
N: 100
a_level: 0
a_trend: 0
b_path: 0.59
missing: 0
N: 100
a_level: 0
a_trend: 0
b_path: 0.59
missing: 0.2
N: 100
a_level: 0
a_trend: 0
b_path: 0.59
missing: 0.5
N: 100
a_level: 0
a_trend: 0.2
b_path: 0
missing: 0
N: 100
a_level: 0
a_trend: 0.2
b_path: 0
missing: 0.2
N: 100
a_level: 0
a_trend: 0.2
b_path: 0
missing: 0.5
Standard Deviation through the Change in Level
0.0
0.5
1.0
1.5
2.0
0
0.1
0.5
0.9
OLS
NW
FGLS
AR(1)
Autoregressive Effect

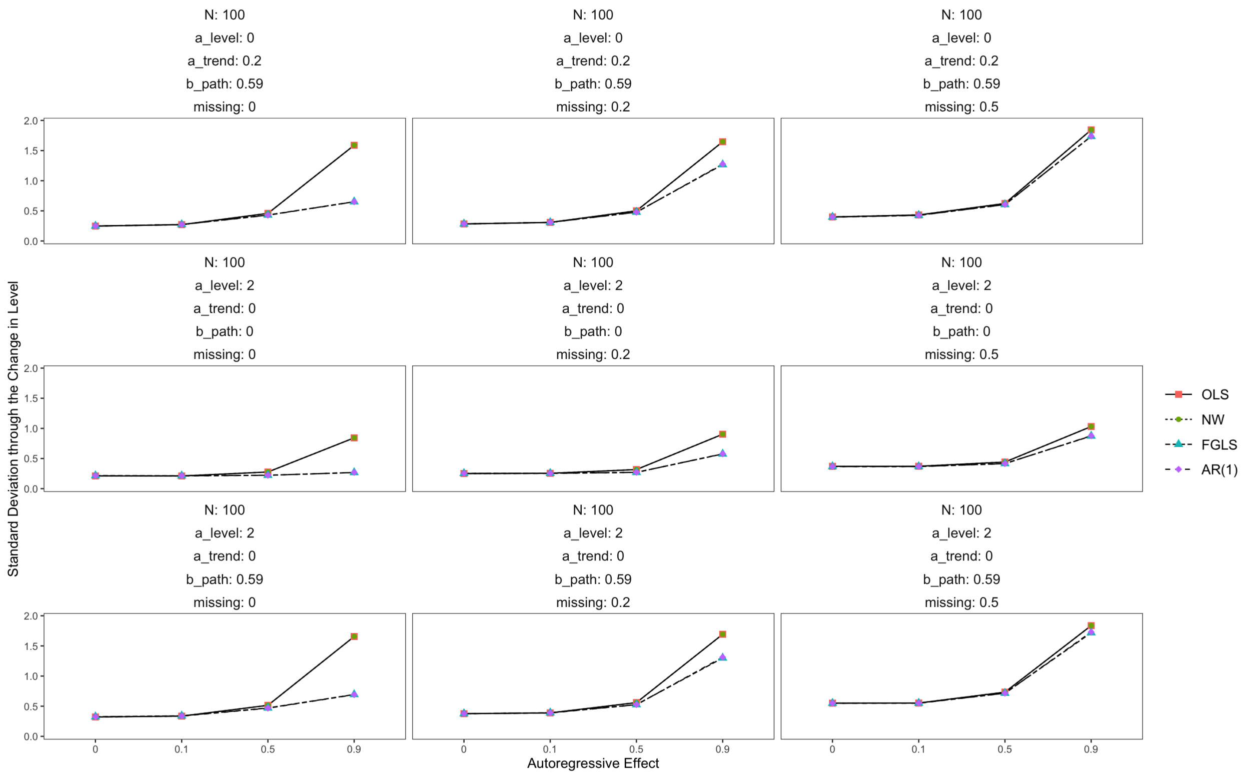

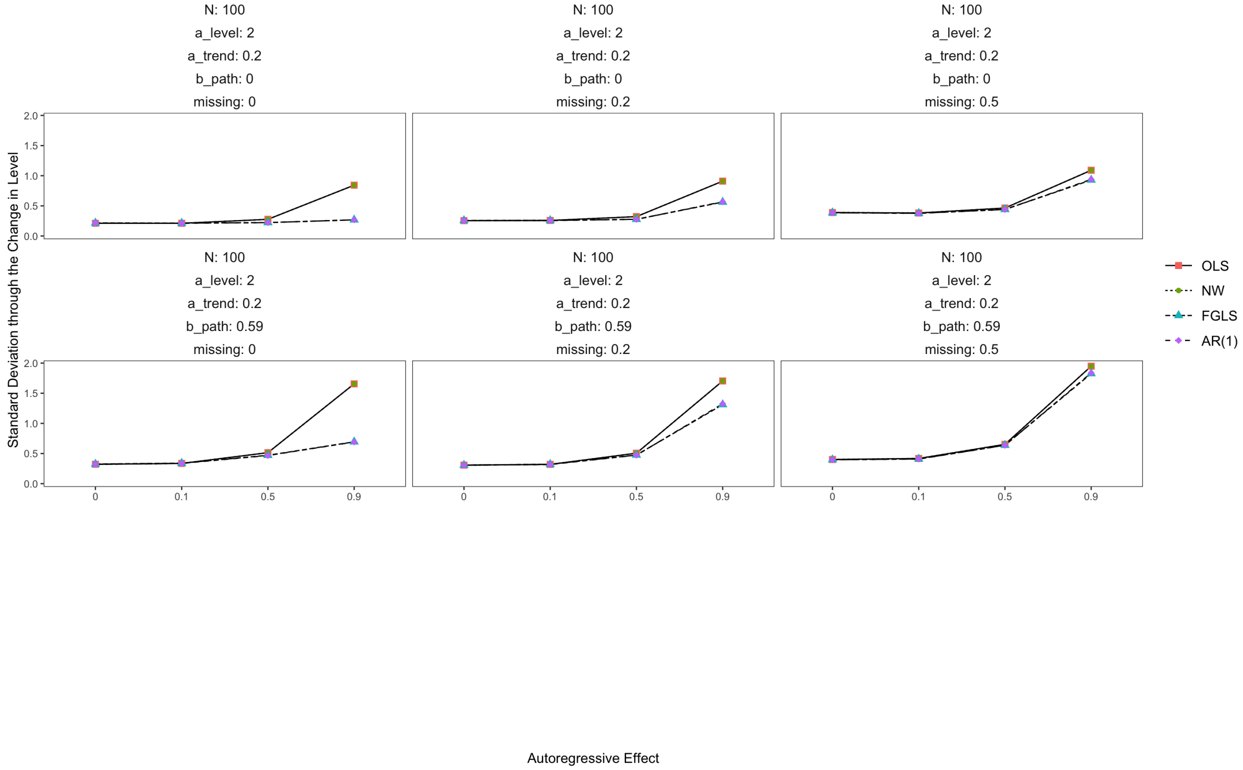

Standard Deviation of the Indirect Effect through the Change in Trend

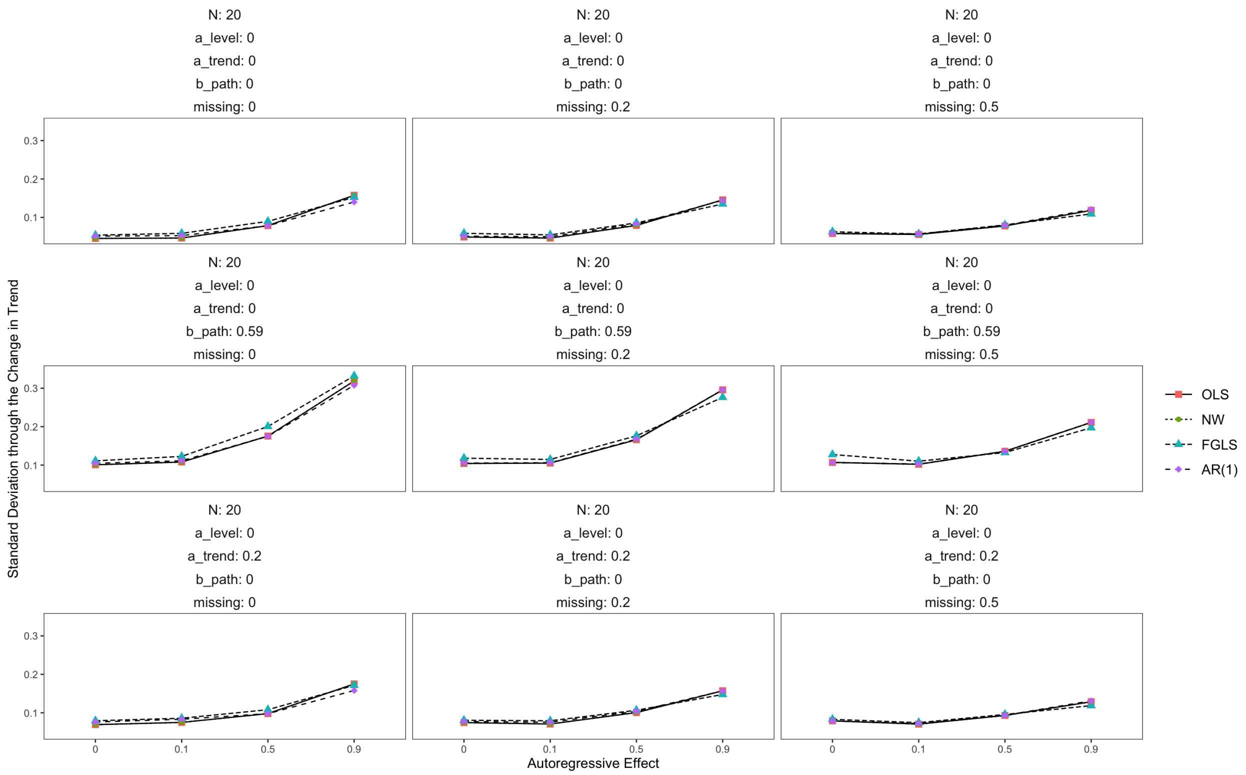
N: 20
a_level: 0
a_trend: 0
b_path: 0
missing: 0
N: 20
a_level: 0
a_trend: 0
b_path: 0
missing: 0.2
N: 20
a_level: 0
a_trend: 0
b_path: 0
missing: 0.5
N: 20
a_level: 0
a_trend: 0
b_path: 0.59
missing: 0
N: 20
a_level: 0
a_trend: 0
b_path: 0.59
missing: 0.2
N: 20
a_level: 0
a_trend: 0
b_path: 0.59
missing: 0.5
N: 20
a_level: 0
a_trend: 0.2
b_path: 0
missing: 0
N: 20
a_level: 0
a_trend: 0.2
b_path: 0
missing: 0.2
N: 20
a_level: 0
a_trend: 0.2
b_path: 0
missing: 0.5
0.1
0.2
0.3
0
0.1
0.5
0.9
Standard Deviation through the Change in Trend
Autoregressive Effect
OLS
NW
FGLS
AR(1)

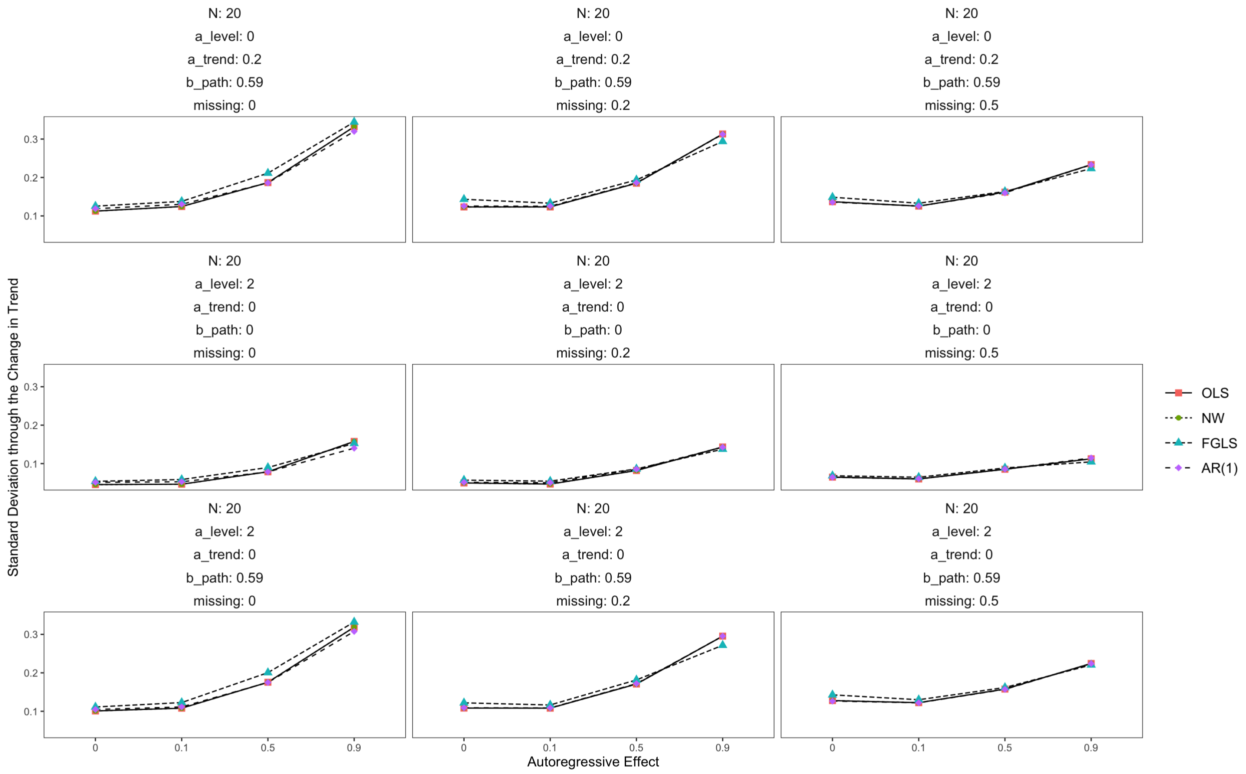
N: 20
a_level: 0
a_trend: 0.2
b_path: 0.59
missing: 0
N: 20
a_level: 0
a_trend: 0.2
b_path: 0.59
missing: 0.2
N: 20
a_level: 0
a_trend: 0.2
b_path: 0.59
missing: 0.5
N: 20
a_level: 2
a_trend: 0
b_path: 0
missing: 0
N: 20
a_level: 2
a_trend: 0
b_path: 0
missing: 0.2
N: 20
a_level: 2
a_trend: 0
b_path: 0
missing: 0.5
N: 20
a_level: 2
a_trend: 0
b_path: 0.59
missing: 0
N: 20
a_level: 2
a_trend: 0
b_path: 0.59
missing: 0.2
N: 20
a_level: 2
a_trend: 0
b_path: 0.59
missing: 0.5
0.1
0.2
0.3
0
0.1
0.5
0.9
Standard Deviation through the Change in Trend
Autoregressive Effect
OLS
NW
FGLS
AR(1)

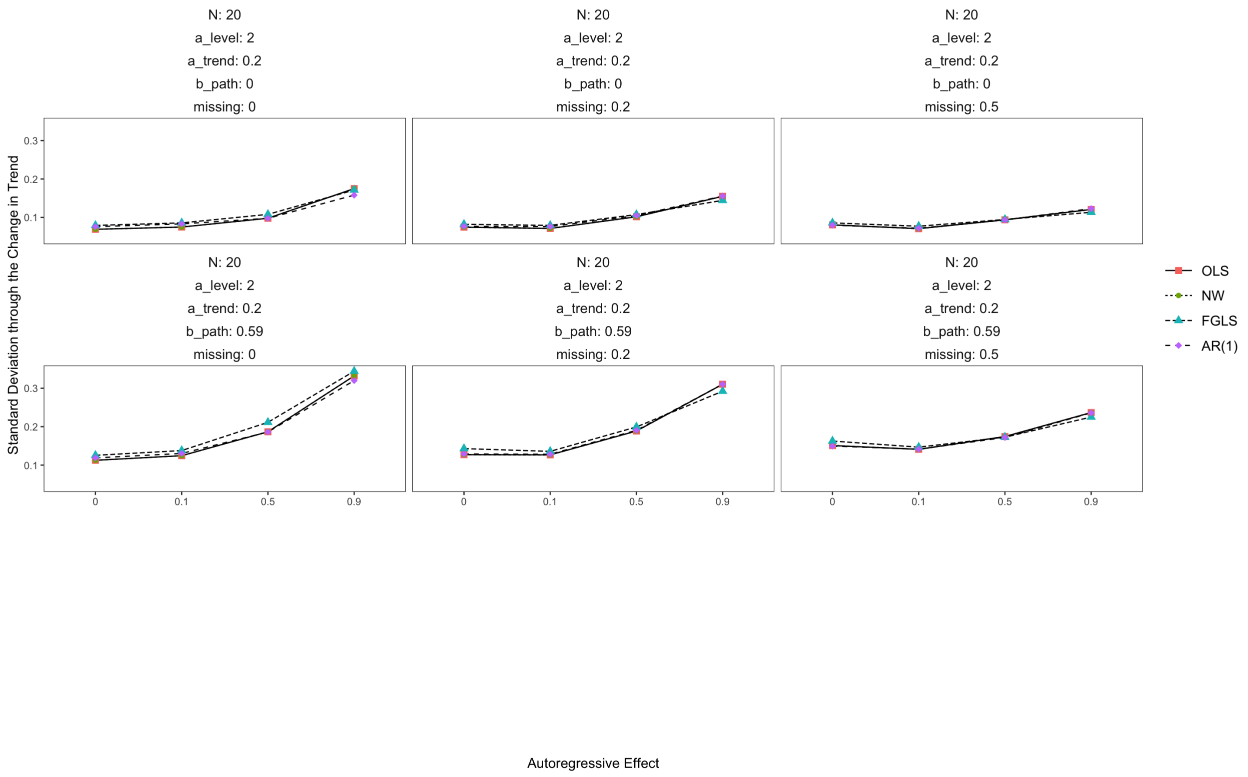
N: 20
a_level: 2
a_trend: 0.2
b_path: 0
missing: 0
N: 20
a_level: 2
a_trend: 0.2
b_path: 0
missing: 0.2
N: 20
a_level: 2
a_trend: 0.2
b_path: 0
missing: 0.5
N: 20
a_level: 2
a_trend: 0.2
b_path: 0.59
missing: 0
N: 20
a_level: 2
a_trend: 0.2
b_path: 0.59
missing: 0.2
N: 20
a_level: 2
a_trend: 0.2
b_path: 0.59
missing: 0.5
Standard Deviation through the Change in Trend
OLS
NW
FGLS
AR(1)
Autoregressive Effect

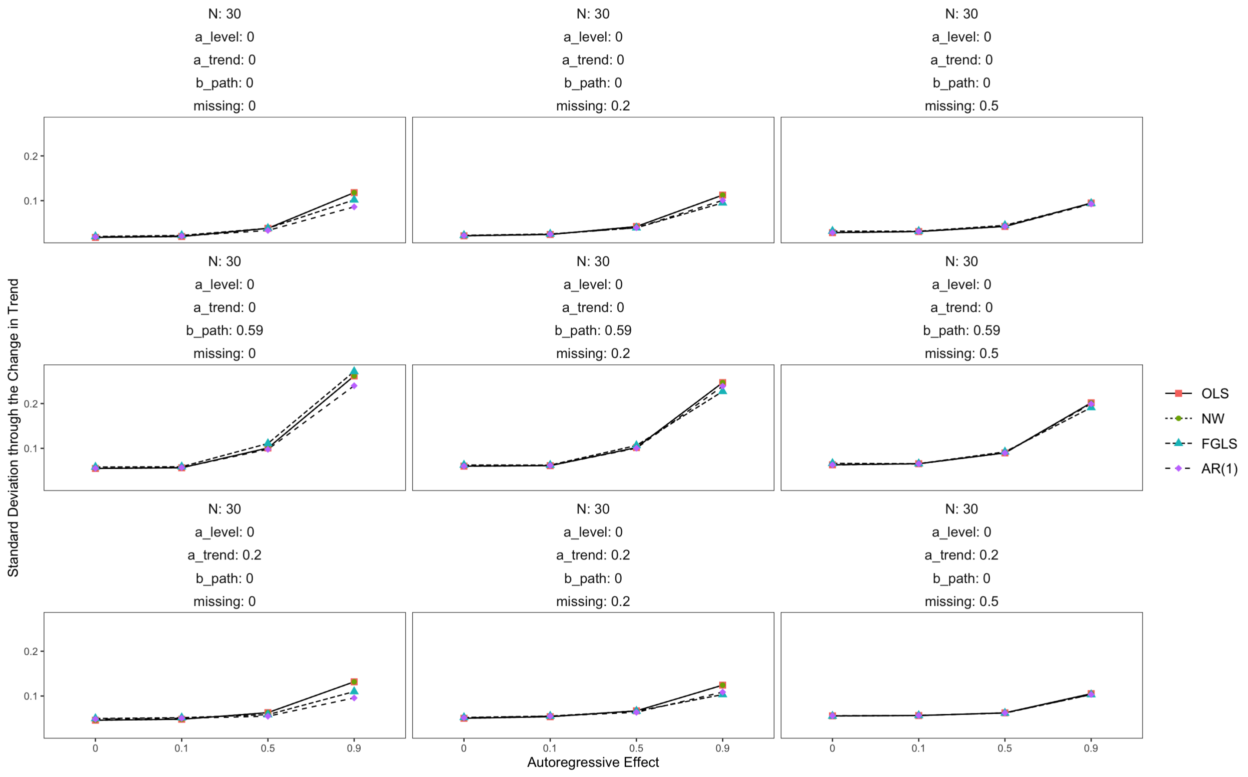
N: 30
a_level: 0
a_trend: 0
b_path: 0
missing: 0
N: 30
a_level: 0
a_trend: 0
b_path: 0
missing: 0.2
N: 30
a_level: 0
a_trend: 0
b_path: 0
missing: 0.5
N: 30
a_level: 0
a_trend: 0
b_path: 0.59
missing: 0
N: 30
a_level: 0
a_trend: 0
b_path: 0.59
missing: 0.2
N: 30
a_level: 0
a_trend: 0
b_path: 0.59
missing: 0.5
N: 30
a_level: 0
a_trend: 0.2
b_path: 0
missing: 0
N: 30
a_level: 0
a_trend: 0.2
b_path: 0
missing: 0.2
N: 30
a_level: 0
a_trend: 0.2
b_path: 0
missing: 0.5
Standard Deviation through the Change in Trend
OLS
NW
FGLS
AR(1)
Autoregressive Effect

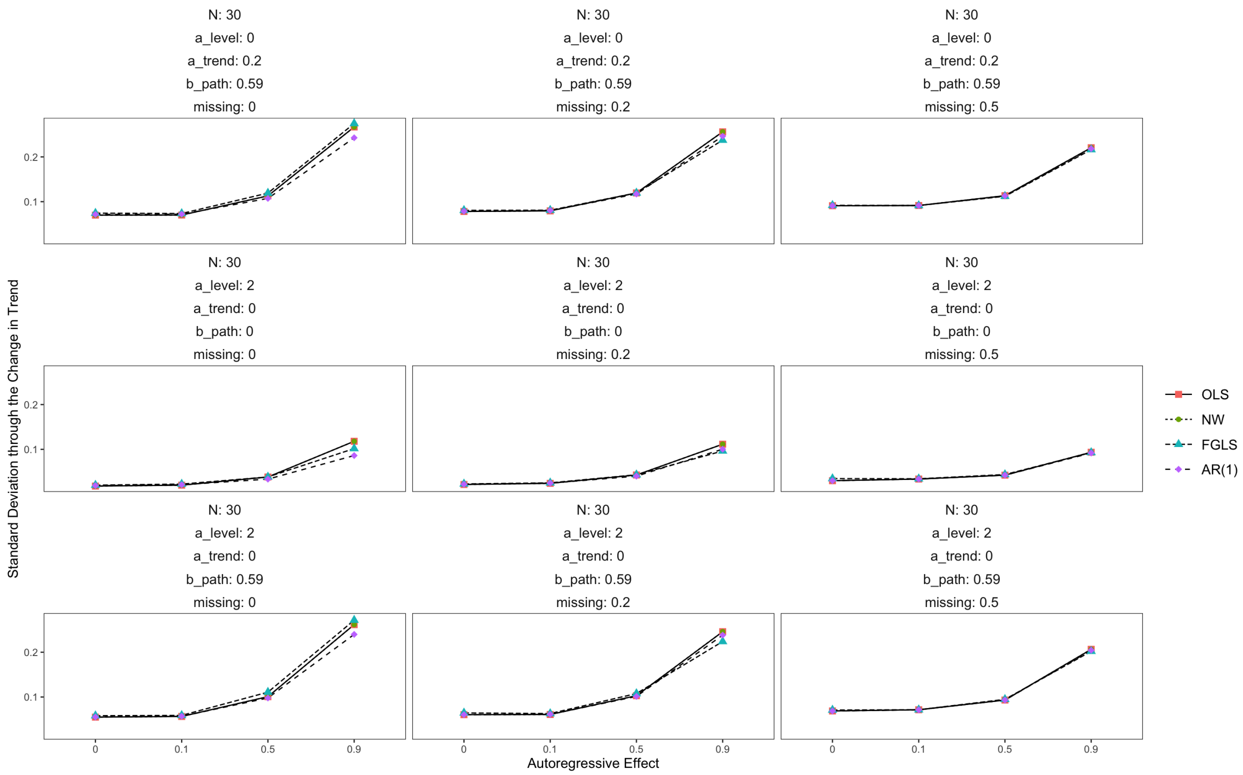
N: 30
a_level: 0
a_trend: 0.2
b_path: 0.59
missing: 0
N: 30
a_level: 0
a_trend: 0.2
b_path: 0.59
missing: 0.2
N: 30
a_level: 0
a_trend: 0.2
b_path: 0.59
missing: 0.5
N: 30
a_level: 2
a_trend: 0
b_path: 0
missing: 0
N: 30
a_level: 2
a_trend: 0
b_path: 0
missing: 0.2
N: 30
a_level: 2
a_trend: 0
b_path: 0
missing: 0.5
N: 30
a_level: 2
a_trend: 0
b_path: 0.59
missing: 0
N: 30
a_level: 2
a_trend: 0
b_path: 0.59
missing: 0.2
N: 30
a_level: 2
a_trend: 0
b_path: 0.59
missing: 0.5
Standard Deviation through the Change in Trend
0.1
0.2
0
0.1
0.5
0.9
Autoregressive Effect
OLS
NW
FGLS
AR(1)

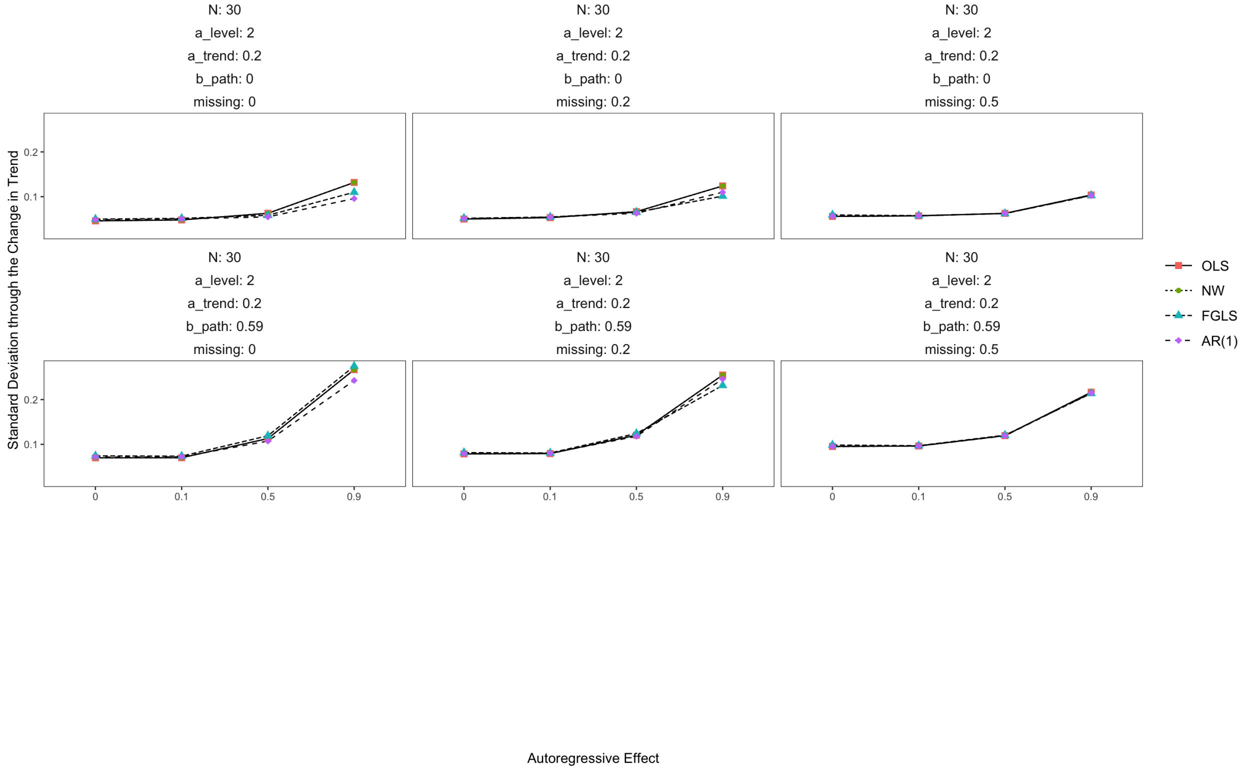
N: 30
a_level: 2
a_trend: 0.2
b_path: 0
missing: 0
N: 30
a_level: 2
a_trend: 0.2
b_path: 0
missing: 0.2
N: 30
a_level: 2
a_trend: 0.2
b_path: 0
missing: 0.5
N: 30
a_level: 2
a_trend: 0.2
b_path: 0.59
missing: 0
N: 30
a_level: 2
a_trend: 0.2
b_path: 0.59
missing: 0.2
N: 30
a_level: 2
a_trend: 0.2
b_path: 0.59
missing: 0.5
Standard Deviation through the Change in Trend
0.1
0.2
0
0.1
0.5
0.9
Autoregressive Effect
OLS
NW
FGLS
AR(1)

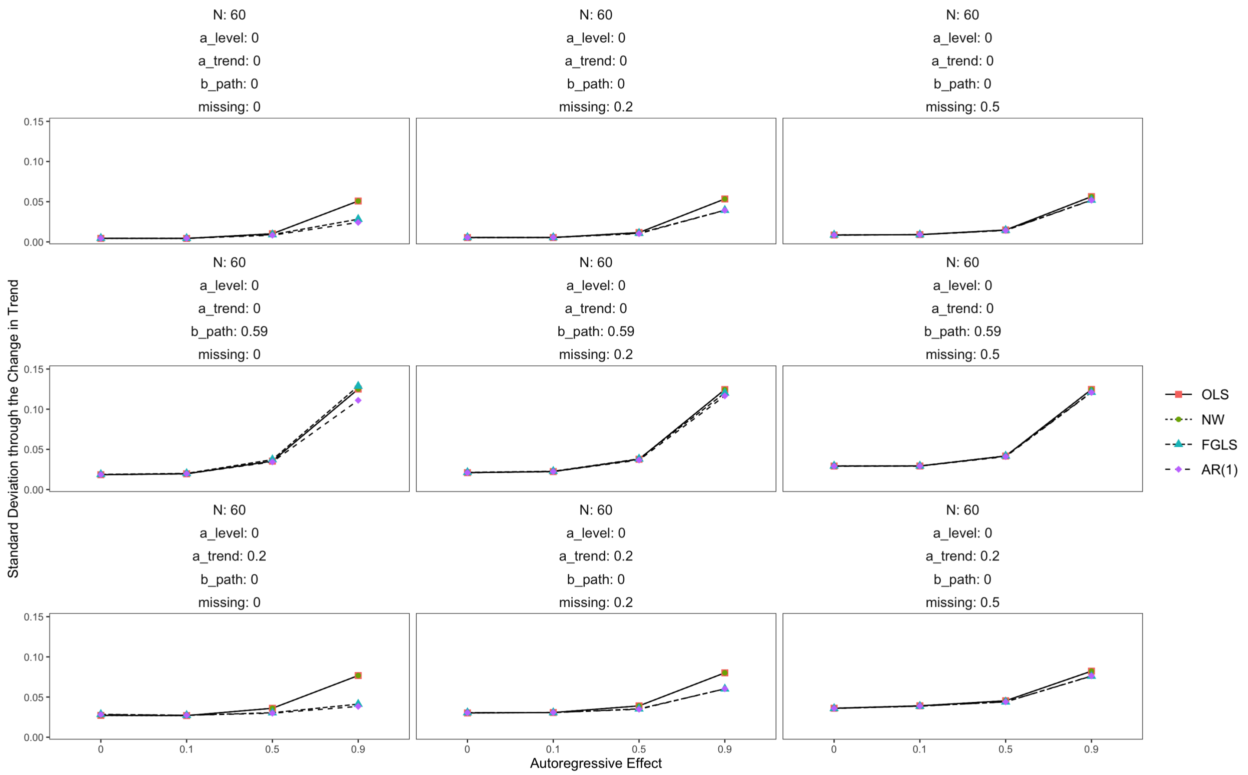
N: 60
a_level: 0
a_trend: 0
b_path: 0
missing: 0
missing: 0.2
missing: 0.5
b_path: 0.59
a_trend: 0.2
Standard Deviation through the Change in Trend
Autoregressive Effect
0.00
0.05
0.10
0.15
0
0.1
0.5
0.9
OLS
NW
FGLS
AR(1)

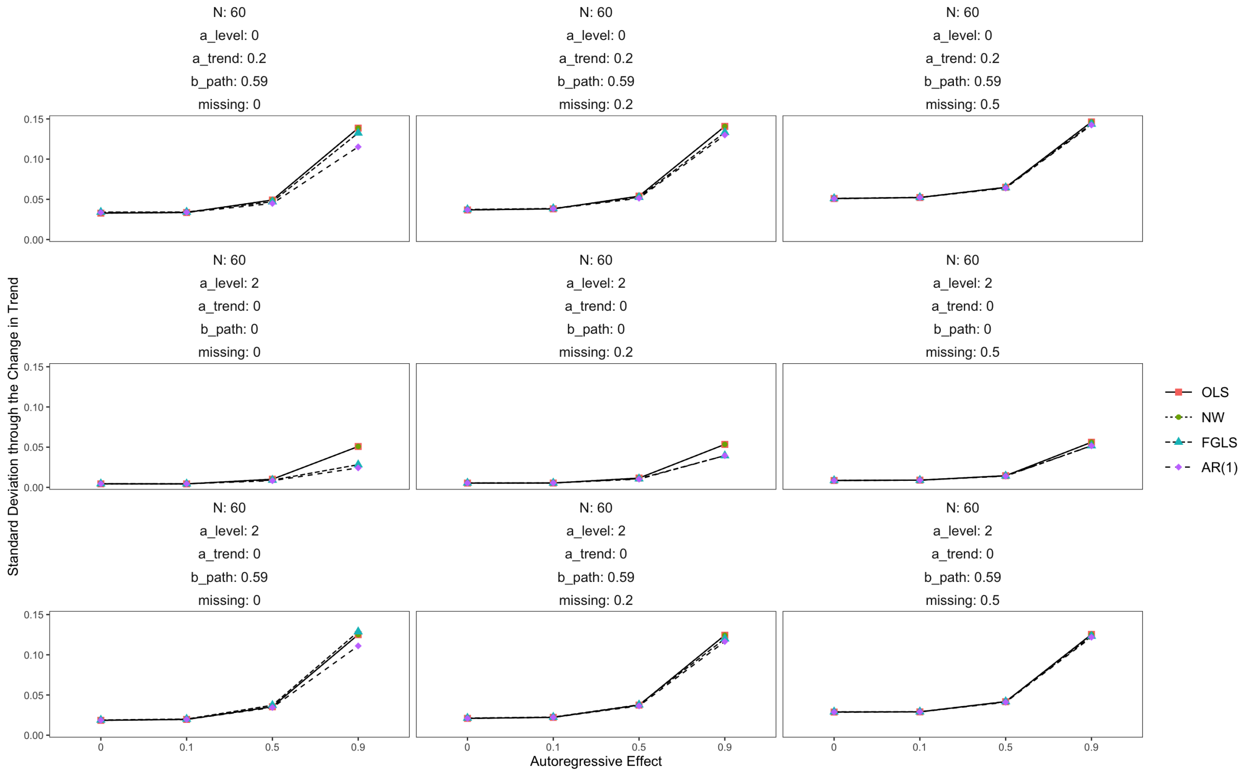
N: 60
a_level: 0
a_trend: 0.2
b_path: 0.59
missing: 0
missing: 0.2
missing: 0.5
a_level: 2
a_trend: 0
b_path: 0
Standard Deviation through the Change in Trend
Autoregressive Effect
0.00
0.05
0.10
0.15
0
0.1
0.5
0.9
OLS
NW
FGLS
AR(1)

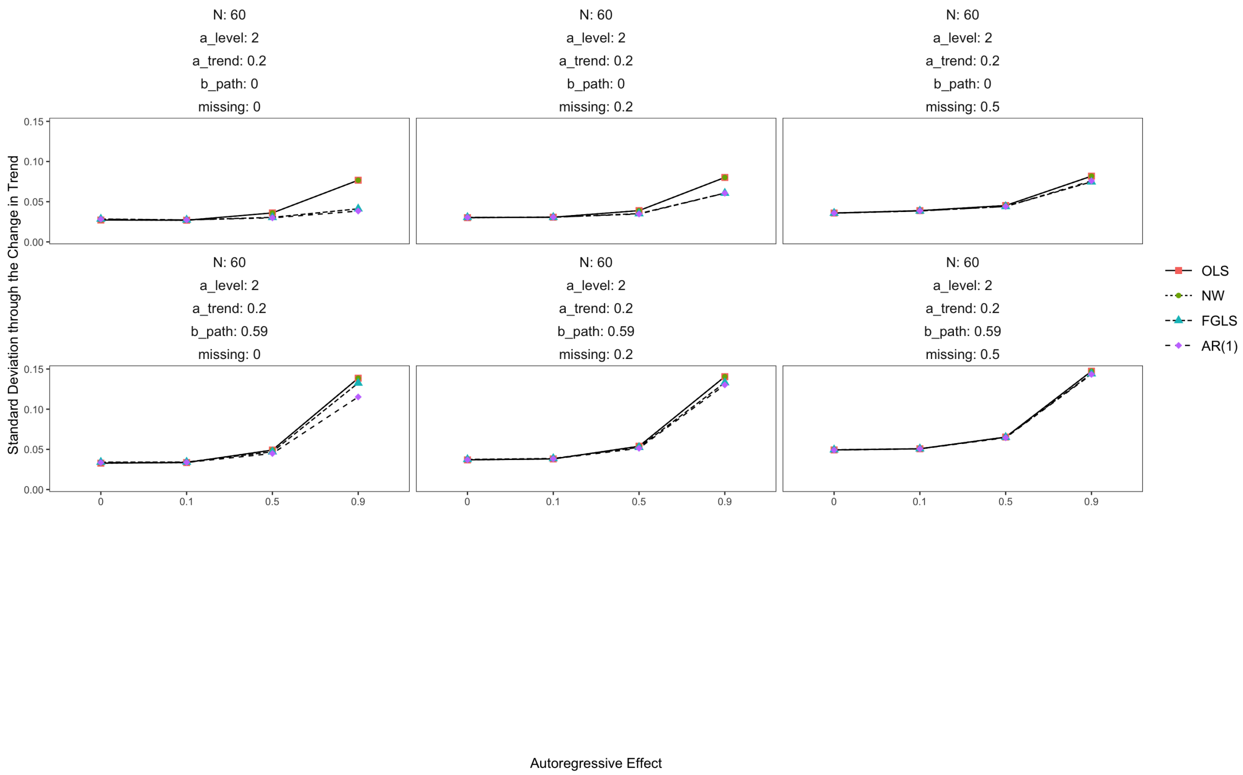
N: 60
a_level: 2
a_trend: 0.2
b_path: 0
missing: 0
N: 60
a_level: 2
a_trend: 0.2
b_path: 0
missing: 0.2
N: 60
a_level: 2
a_trend: 0.2
b_path: 0
missing: 0.5
N: 60
a_level: 2
a_trend: 0.2
b_path: 0.59
missing: 0
N: 60
a_level: 2
a_trend: 0.2
b_path: 0.59
missing: 0.2
N: 60
a_level: 2
a_trend: 0.2
b_path: 0.59
missing: 0.5
Standard Deviation through the Change in Trend
0.15
0.10
0.05
0.00
0
0.1
0.5
0.9
OLS
NW
FGLS
AR(1)
Autoregressive Effect

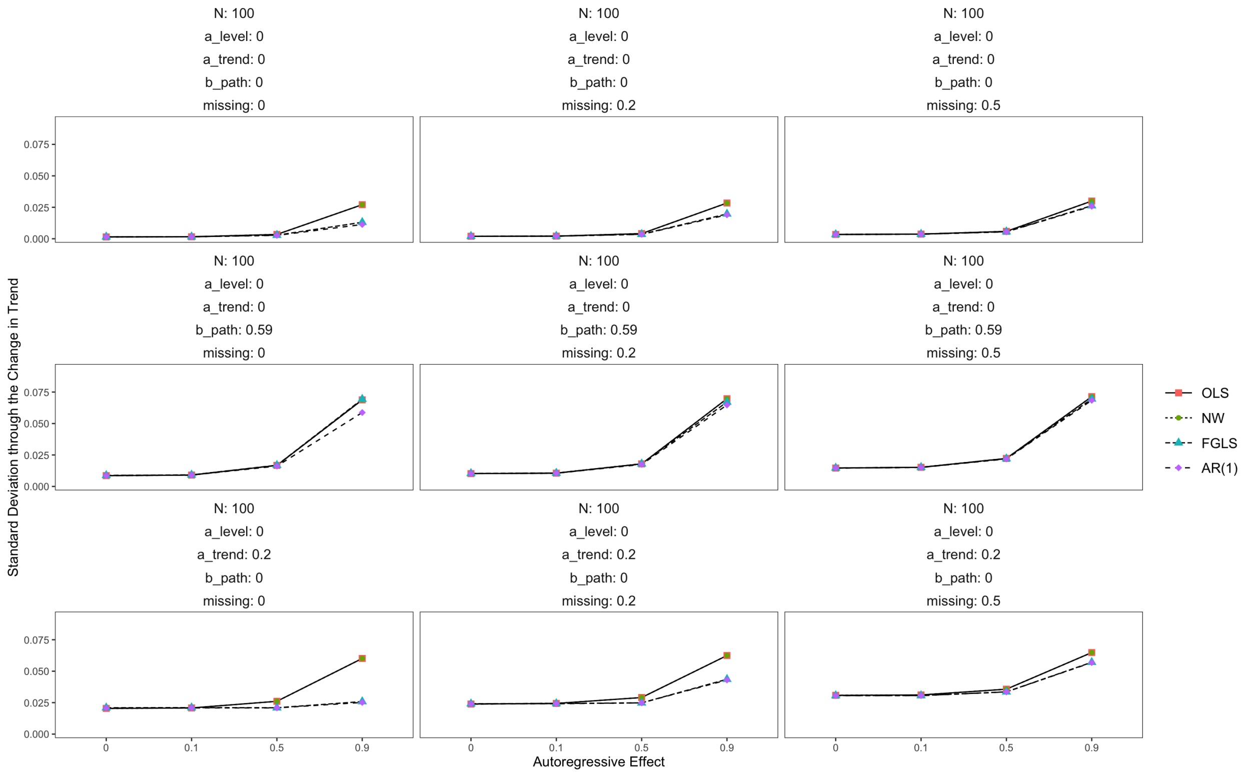
N: 100
a_level: 0
a_trend: 0
b_path: 0
missing: 0
N: 100
a_level: 0
a_trend: 0
b_path: 0
missing: 0.2
N: 100
a_level: 0
a_trend: 0
b_path: 0
missing: 0.5
N: 100
a_level: 0
a_trend: 0
b_path: 0.59
missing: 0
N: 100
a_level: 0
a_trend: 0
b_path: 0.59
missing: 0.2
N: 100
a_level: 0
a_trend: 0
b_path: 0.59
missing: 0.5
N: 100
a_level: 0
a_trend: 0.2
b_path: 0
missing: 0
N: 100
a_level: 0
a_trend: 0.2
b_path: 0
missing: 0.2
N: 100
a_level: 0
a_trend: 0.2
b_path: 0
missing: 0.5
Standard Deviation through the Change in Trend
0.075
0.050
0.025
0.000
0
0.1
0.5
0.9
OLS
NW
FGLS
AR(1)
Autoregressive Effect

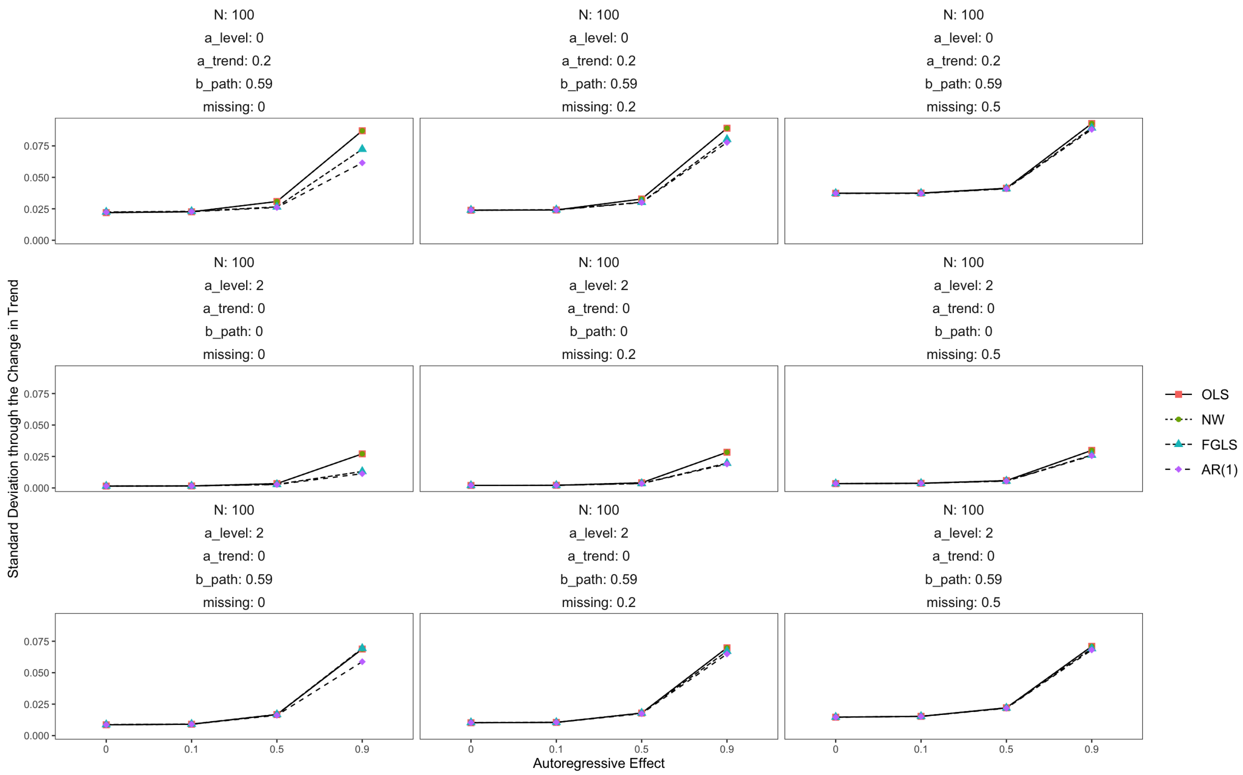
N: 100
a_level: 0
a_trend: 0.2
b_path: 0.59
missing: 0
N: 100
a_level: 0
a_trend: 0.2
b_path: 0.59
missing: 0.2
N: 100
a_level: 0
a_trend: 0.2
b_path: 0.59
missing: 0.5
N: 100
a_level: 2
a_trend: 0
b_path: 0
missing: 0
N: 100
a_level: 2
a_trend: 0
b_path: 0
missing: 0.2
N: 100
a_level: 2
a_trend: 0
b_path: 0
missing: 0.5
N: 100
a_level: 2
a_trend: 0
b_path: 0.59
missing: 0
N: 100
a_level: 2
a_trend: 0
b_path: 0.59
missing: 0.2
N: 100
a_level: 2
a_trend: 0
b_path: 0.59
missing: 0.5
0.000
0.025
0.050
0.075
0
0.1
0.5
0.9
Standard Deviation through the Change in Trend
Autoregressive Effect
OLS
NW
FGLS
AR(1)

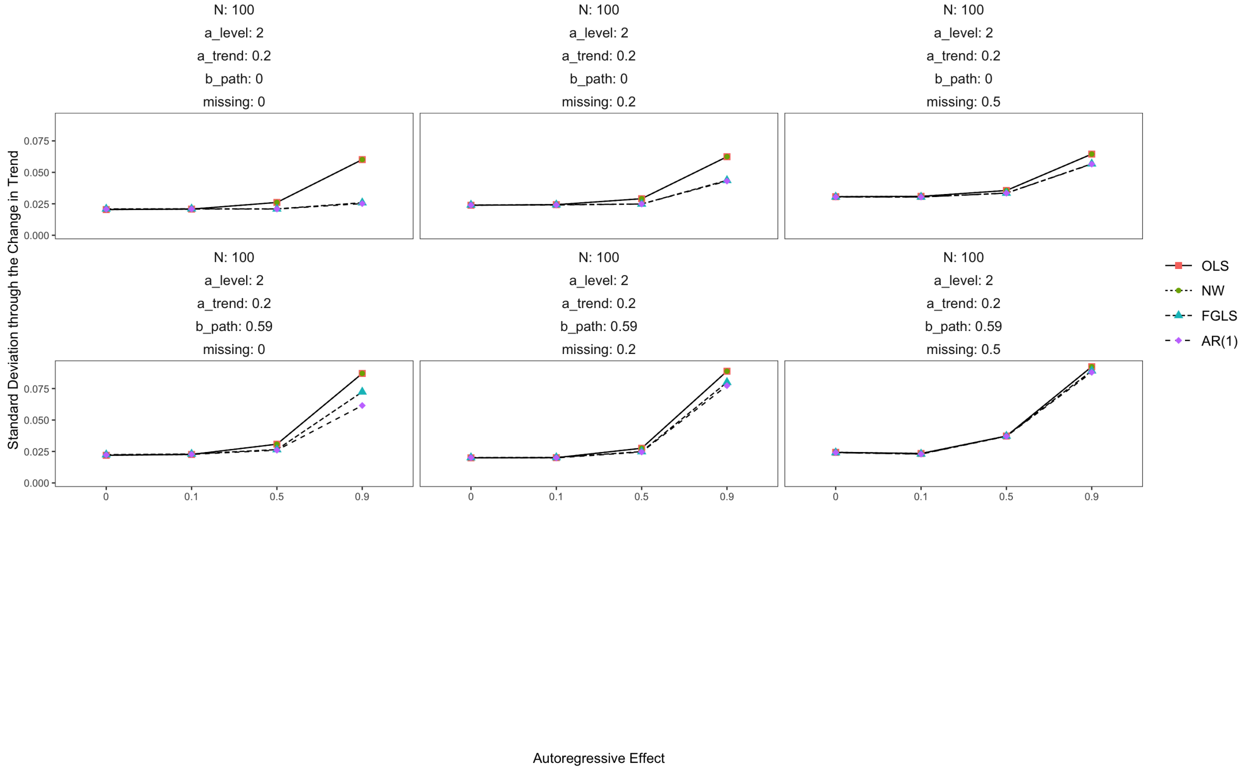
N: 100
a_level: 2
a_trend: 0.2
b_path: 0
missing: 0
N: 100
a_level: 2
a_trend: 0.2
b_path: 0
missing: 0.2
N: 100
a_level: 2
a_trend: 0.2
b_path: 0
missing: 0.5
N: 100
a_level: 2
a_trend: 0.2
b_path: 0.59
missing: 0
N: 100
a_level: 2
a_trend: 0.2
b_path: 0.59
missing: 0.2
N: 100
a_level: 2
a_trend: 0.2
b_path: 0.59
missing: 0.5
0.000
0.025
0.050
0.075
0
0.1
0.5
0.9
Standard Deviation through the Change in Trend
Autoregressive Effect
OLS
NW
FGLS
AR(1)

Power of the Interval Estimate of the Indirect Effect through the Change in Level

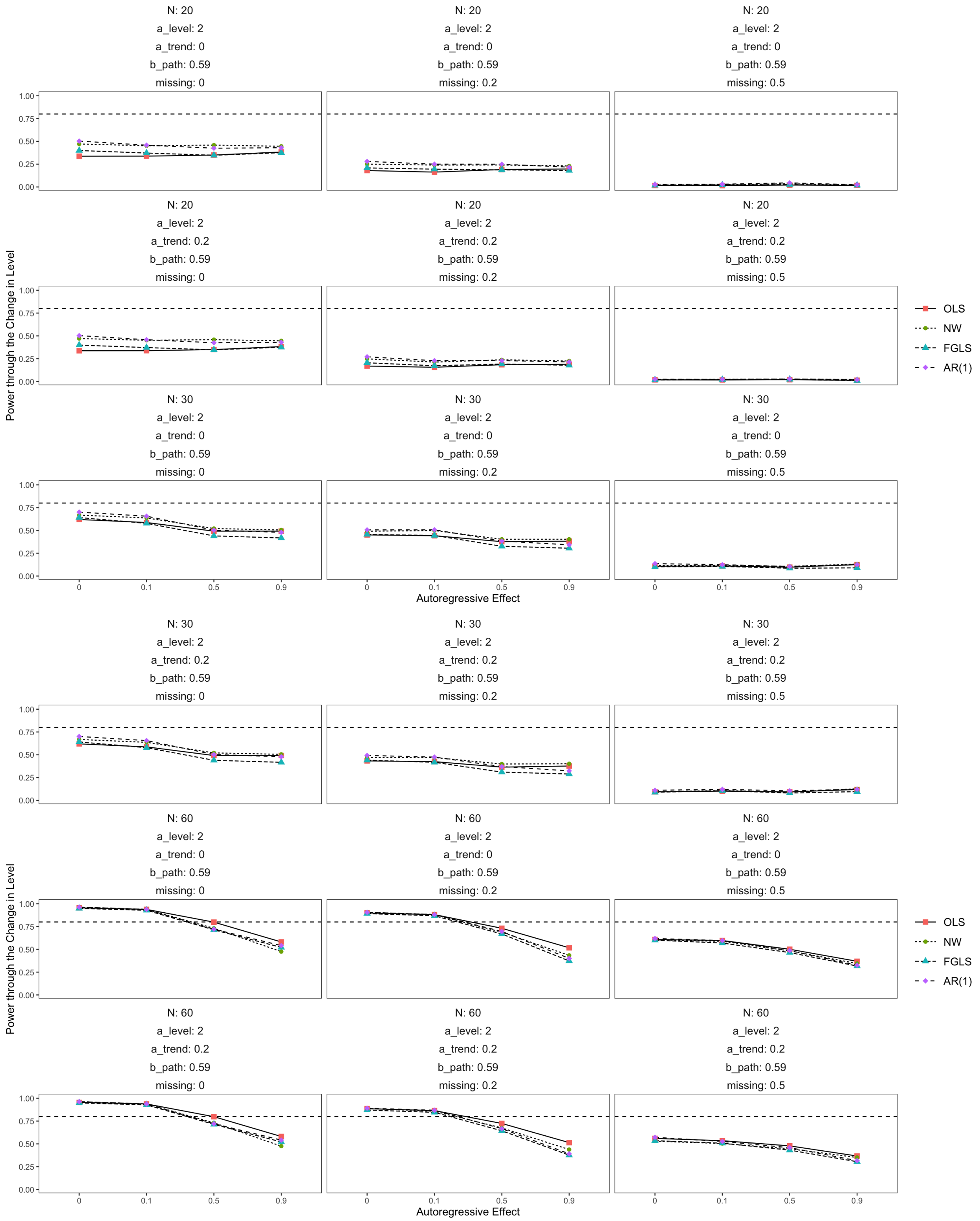

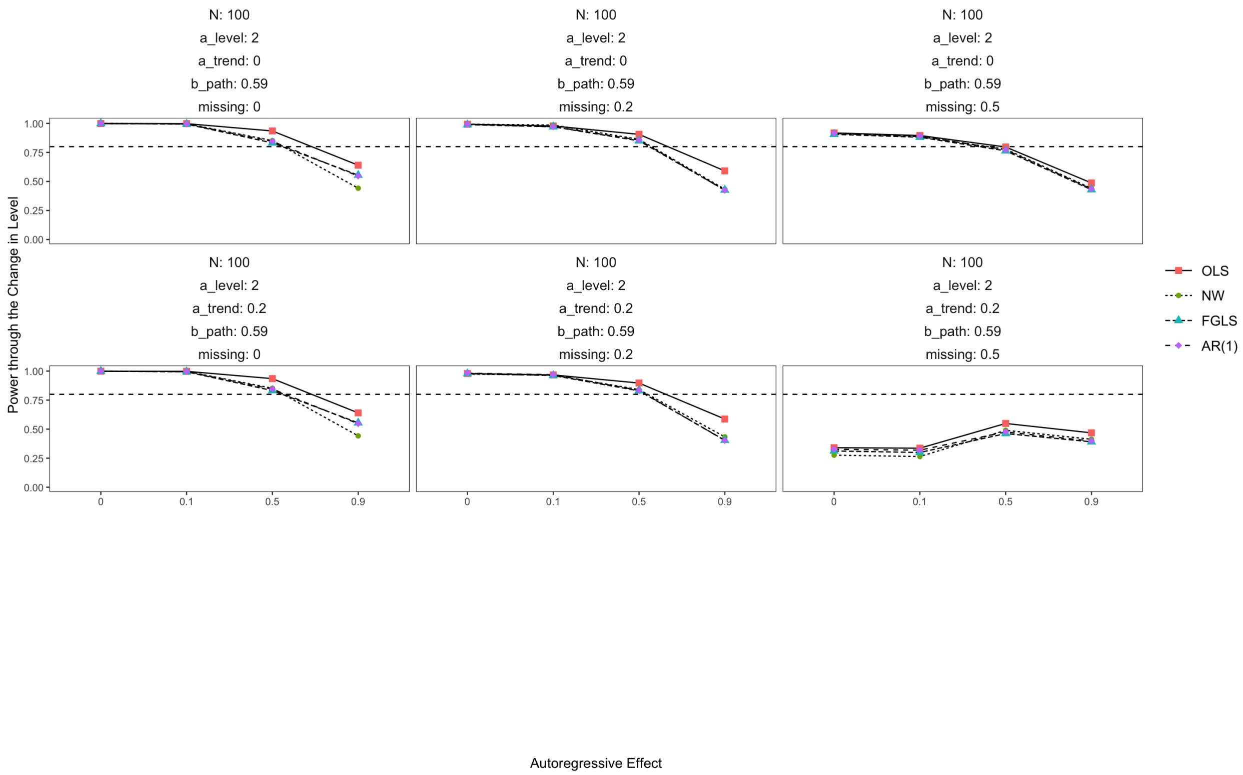

Power of the Interval Estimate of the Indirect Effect through the Change in Trend

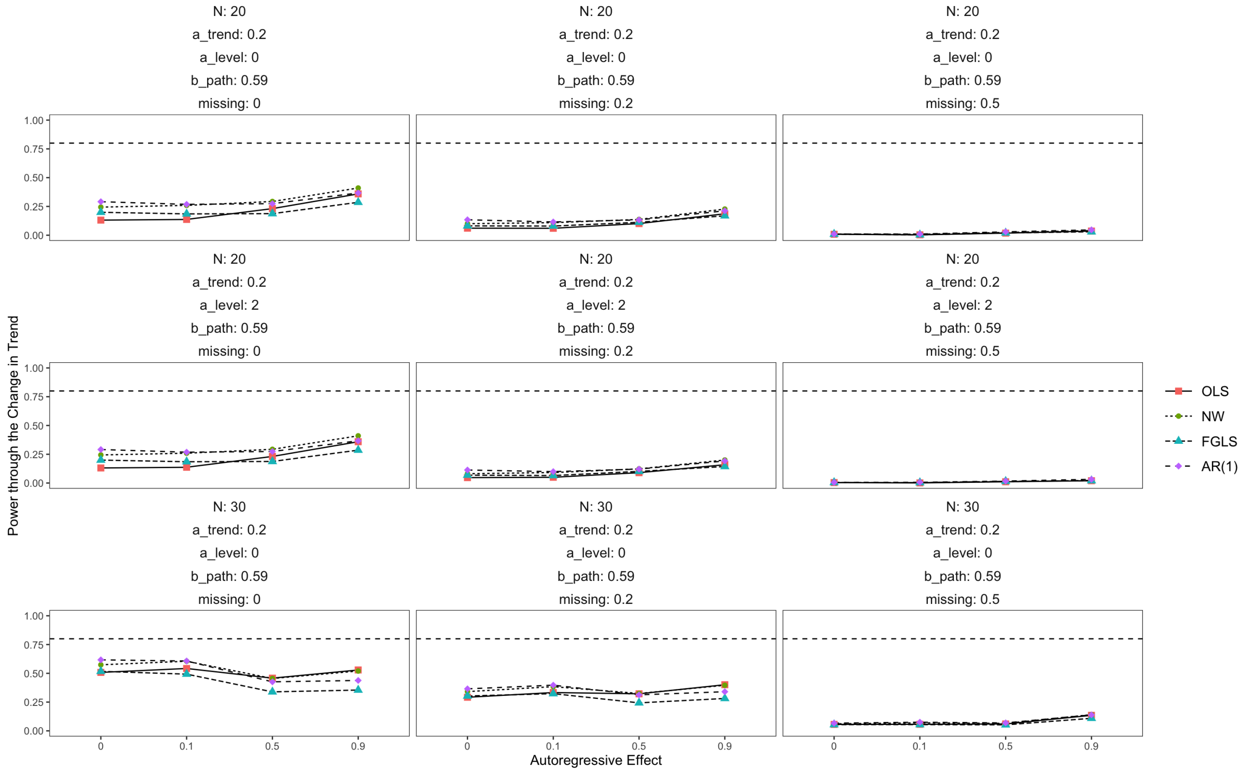

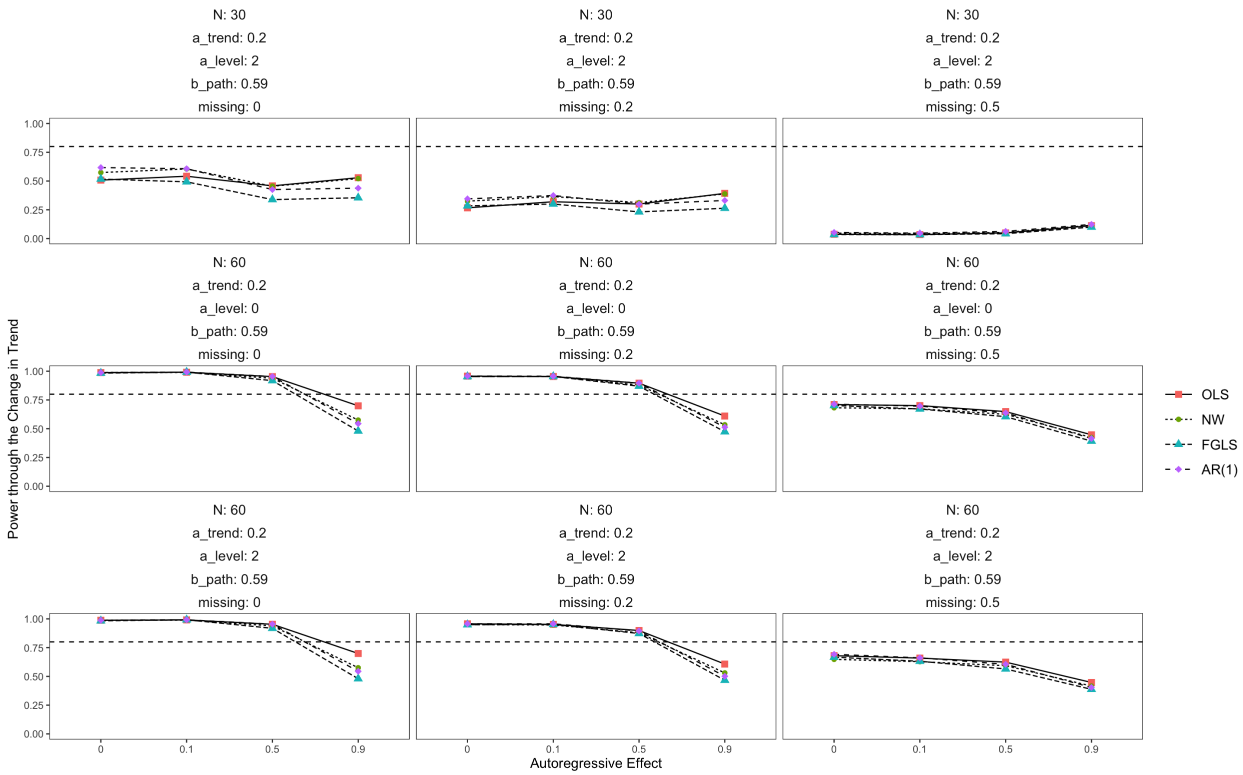
N: 30
a_trend: 0.2
a_level: 2
b_path: 0.59
missing: 0
N: 30
a_trend: 0.2
a_level: 2
b_path: 0.59
missing: 0.2
N: 30
a_trend: 0.2
a_level: 2
b_path: 0.59
missing: 0.5
N: 60
a_trend: 0.2
a_level: 0
b_path: 0.59
missing: 0
N: 60
a_trend: 0.2
a_level: 0
b_path: 0.59
missing: 0.2
N: 60
a_trend: 0.2
a_level: 0
b_path: 0.59
missing: 0.5
N: 60
a_trend: 0.2
a_level: 2
b_path: 0.59
missing: 0
N: 60
a_trend: 0.2
a_level: 2
b_path: 0.59
missing: 0.2
N: 60
a_trend: 0.2
a_level: 2
b_path: 0.59
missing: 0.5
1.00
0.75
0.50
0.25
0.00
0
0.1
0.5
0.9
Power through the Change in Trend
Autoregressive Effect
OLS
NW
FGLS
AR(1)

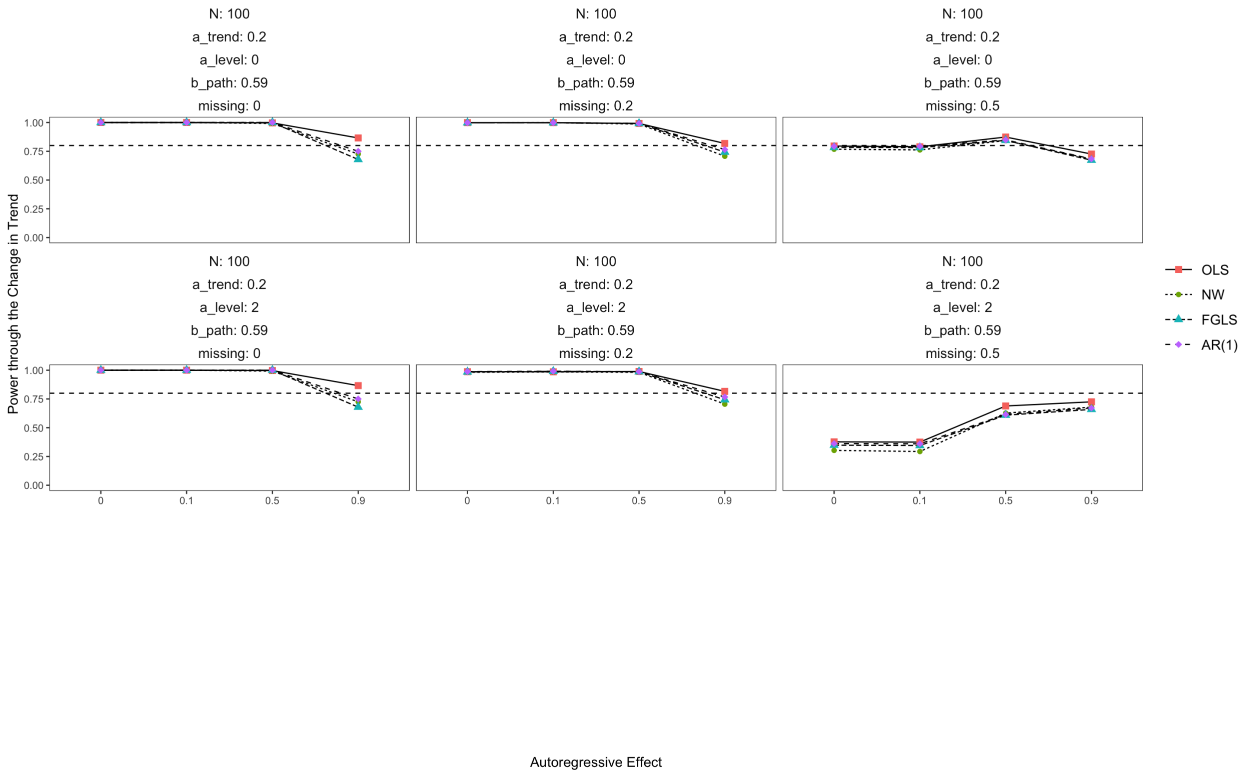
N: 100
a_trend: 0.2
a_level: 0
b_path: 0.59
missing: 0
N: 100
a_trend: 0.2
a_level: 0
b_path: 0.59
missing: 0.2
N: 100
a_trend: 0.2
a_level: 0
b_path: 0.59
missing: 0.5
N: 100
a_trend: 0.2
a_level: 2
b_path: 0.59
missing: 0
N: 100
a_trend: 0.2
a_level: 2
b_path: 0.59
missing: 0.2
N: 100
a_trend: 0.2
a_level: 2
b_path: 0.59
missing: 0.5
1.00
0.75
0.50
0.25
0.00
0
0.1
0.5
0.9
Power through the Change in Trend
Autoregressive Effect
OLS
NW
FGLS
AR(1)

Type I Error of the Interval Estimate through the Change in Level

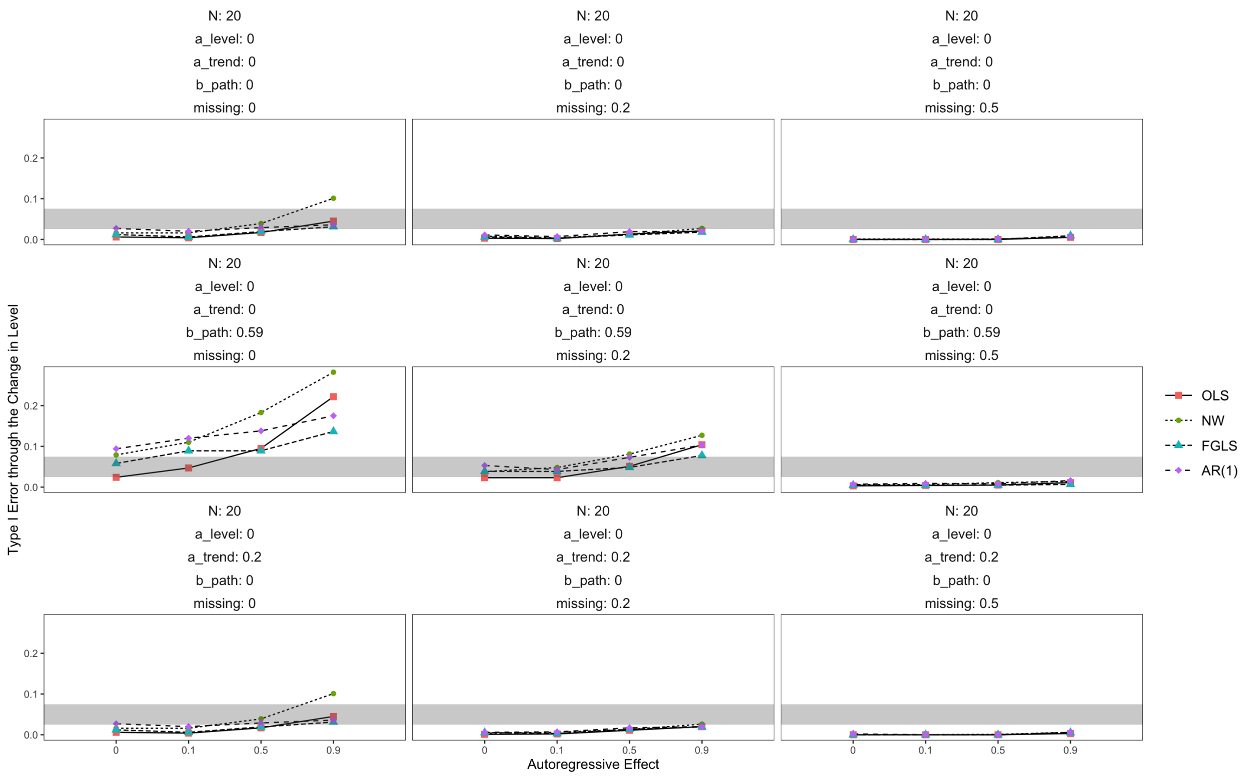

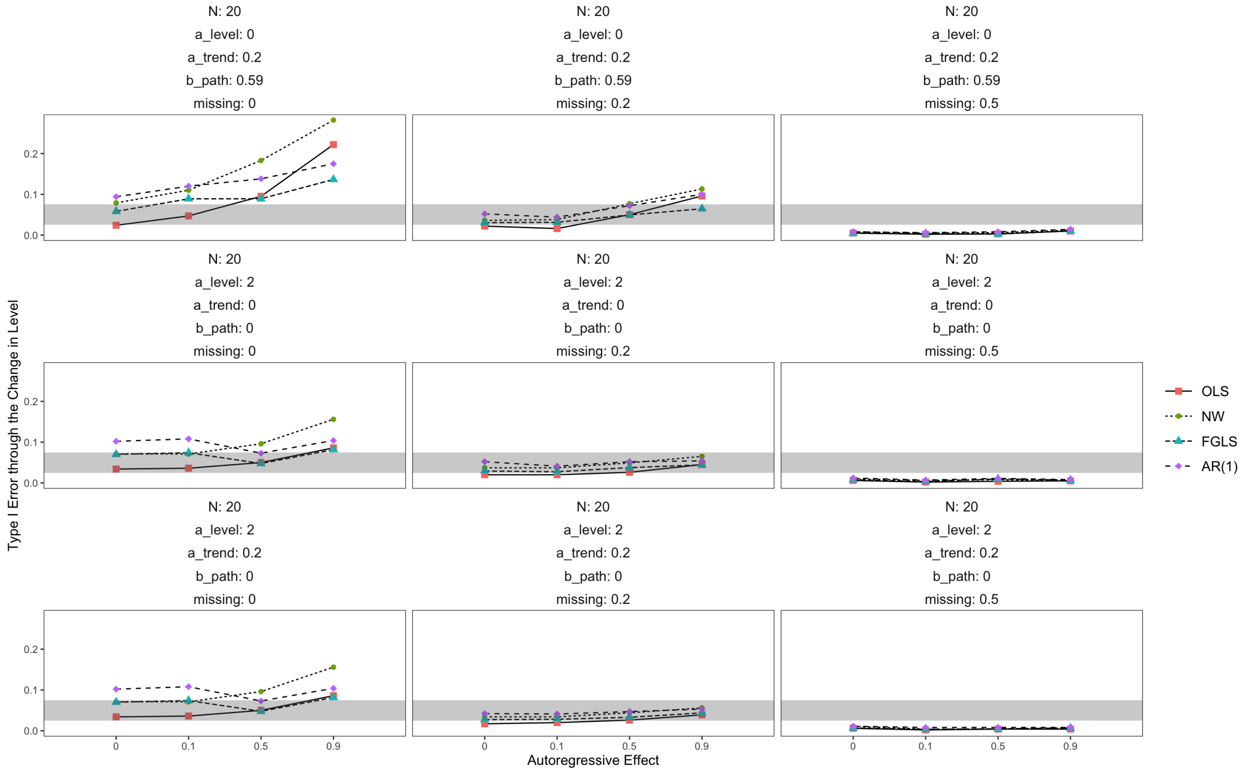

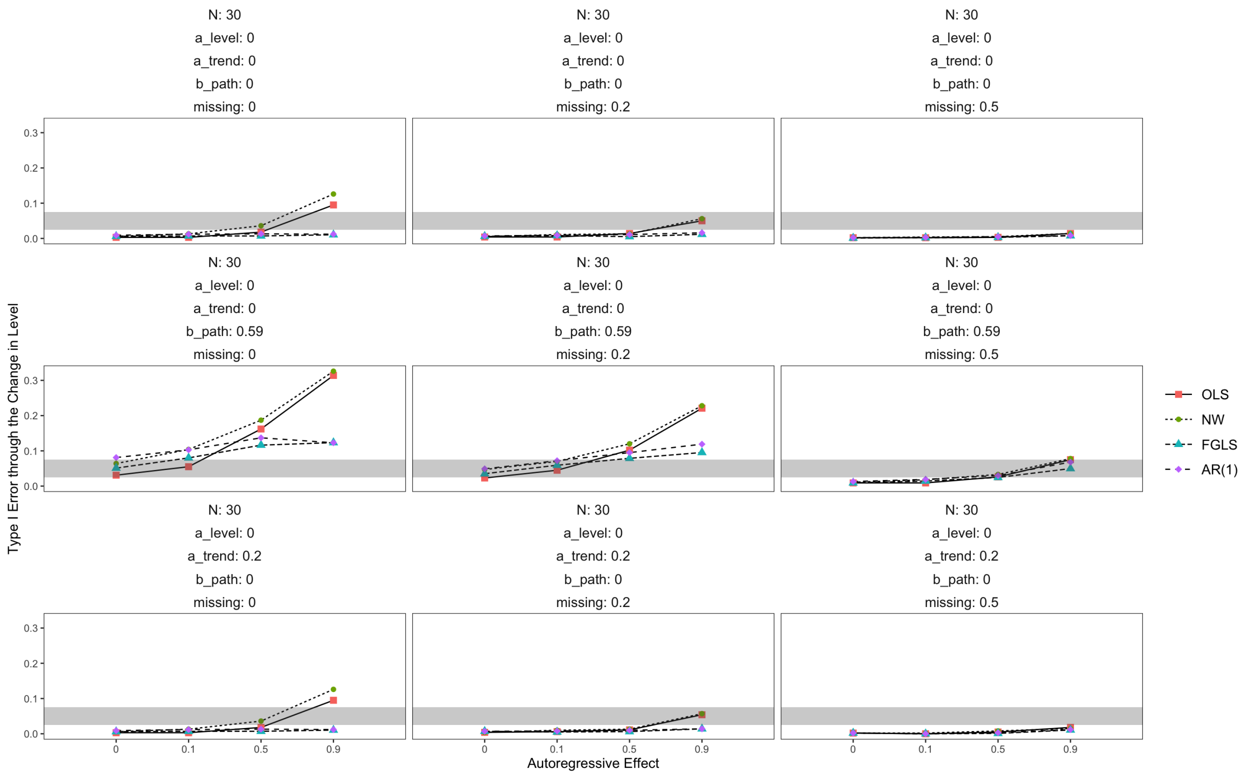
N: 30
a_level: 0
a_trend: 0
b_path: 0
missing: 0
N: 30
a_level: 0
a_trend: 0
b_path: 0
missing: 0.2
N: 30
a_level: 0
a_trend: 0
b_path: 0
missing: 0.5
N: 30
a_level: 0
a_trend: 0
b_path: 0.59
missing: 0
N: 30
a_level: 0
a_trend: 0
b_path: 0.59
missing: 0.2
N: 30
a_level: 0
a_trend: 0
b_path: 0.59
missing: 0.5
N: 30
a_level: 0
a_trend: 0.2
b_path: 0
missing: 0
N: 30
a_level: 0
a_trend: 0.2
b_path: 0
missing: 0.2
N: 30
a_level: 0
a_trend: 0.2
b_path: 0
missing: 0.5
0.0
0.1
0.2
0.3
0
0.1
0.5
0.9
Type I Error through the Change in Level
Autoregressive Effect
OLS
NW
FGLS
AR(1)

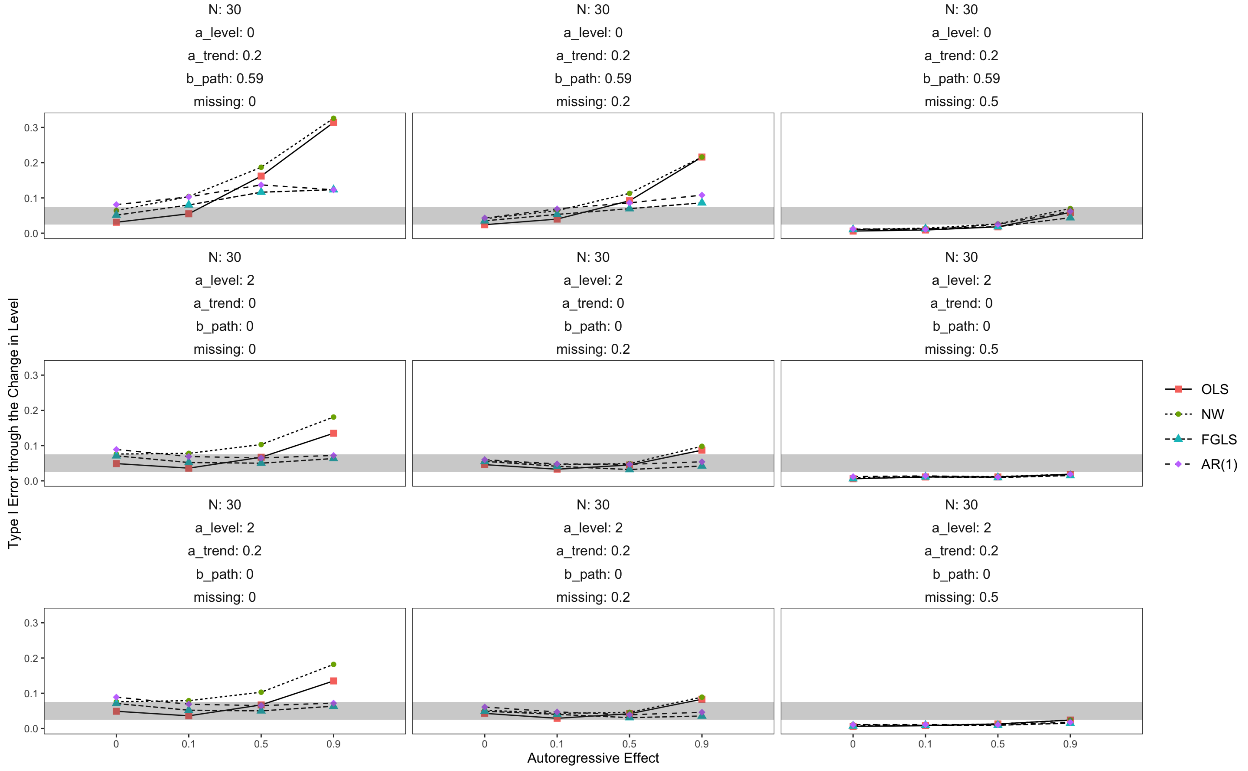
N: 30
a_level: 0
a_trend: 0.2
b_path: 0.59
missing: 0
N: 30
a_level: 0
a_trend: 0.2
b_path: 0.59
missing: 0.2
N: 30
a_level: 0
a_trend: 0.2
b_path: 0.59
missing: 0.5
N: 30
a_level: 2
a_trend: 0
b_path: 0
missing: 0
N: 30
a_level: 2
a_trend: 0
b_path: 0
missing: 0.2
N: 30
a_level: 2
a_trend: 0
b_path: 0
missing: 0.5
N: 30
a_level: 2
a_trend: 0.2
b_path: 0
missing: 0
N: 30
a_level: 2
a_trend: 0.2
b_path: 0
missing: 0.2
N: 30
a_level: 2
a_trend: 0.2
b_path: 0
missing: 0.5
0.0
0.1
0.2
0.3
0
0.1
0.5
0.9
Type I Error through the Change in Level
Autoregressive Effect
OLS
NW
FGLS
AR(1)

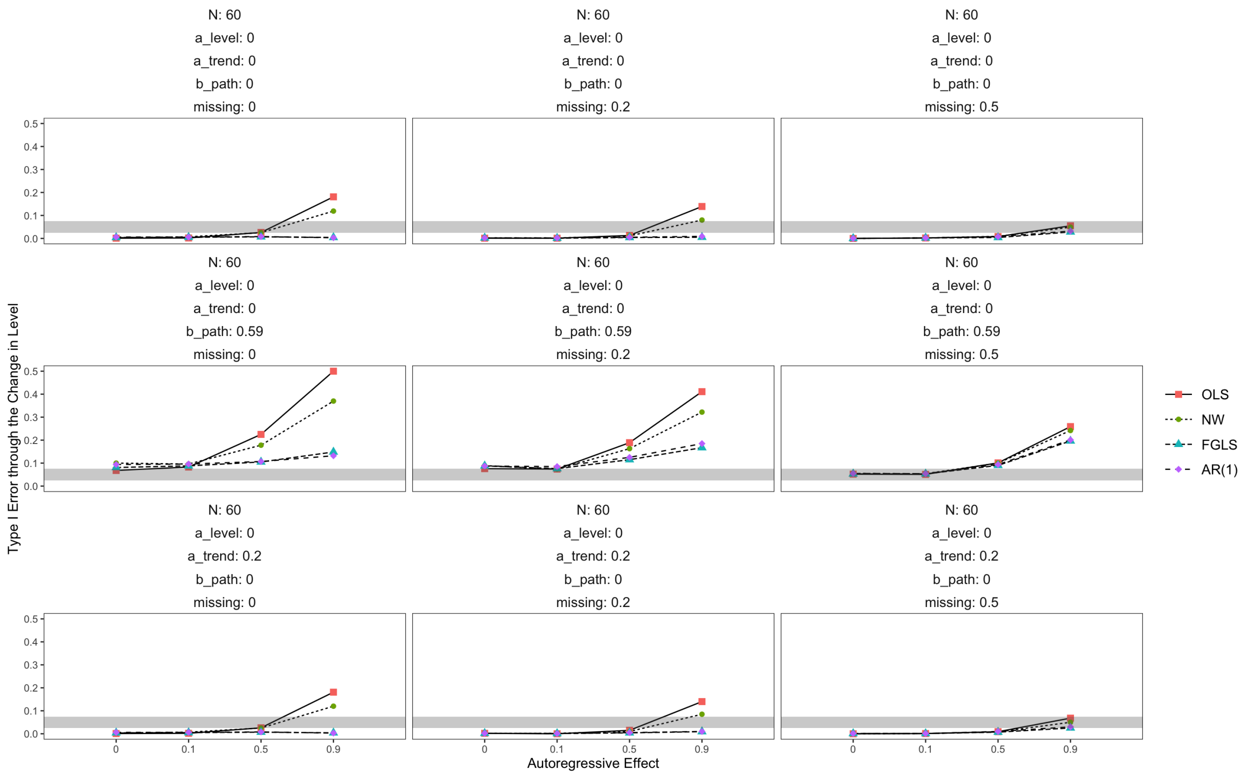
N: 60
a_level: 0
a_trend: 0
b_path: 0
missing: 0
missing: 0.2
missing: 0.5
b_path: 0.59
a_trend: 0.2
Type I Error through the Change in Level
Autoregressive Effect
0
0.1
0.5
0.9
0.0
0.1
0.2
0.3
0.4
0.5
OLS
NW
FGLS
AR(1)

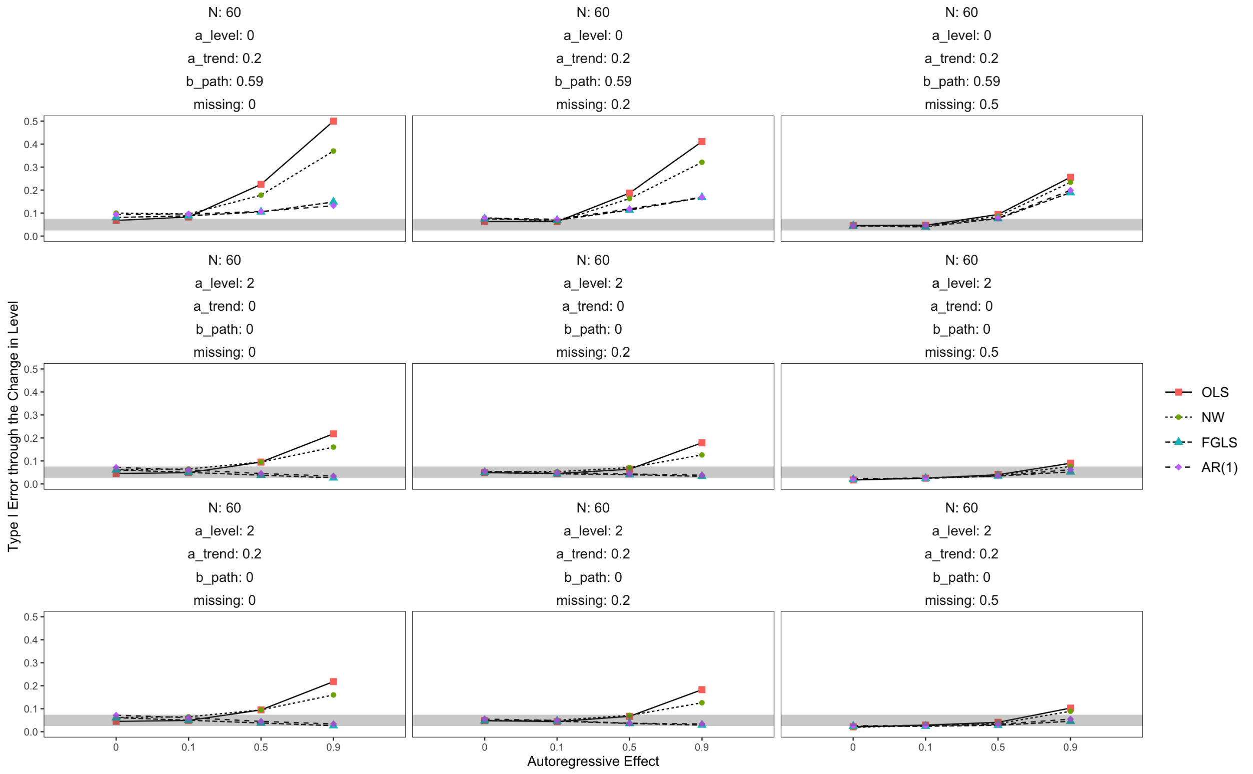
N: 60
a_level: 0
a_level: 2
a_trend: 0.2
a_trend: 0
b_path: 0.59
b_path: 0
missing: 0
missing: 0.2
missing: 0.5
Type I Error through the Change in Level
Autoregressive Effect
0
0.1
0.5
0.9
0.0
0.1
0.2
0.3
0.4
0.5
OLS
NW
FGLS
AR(1)

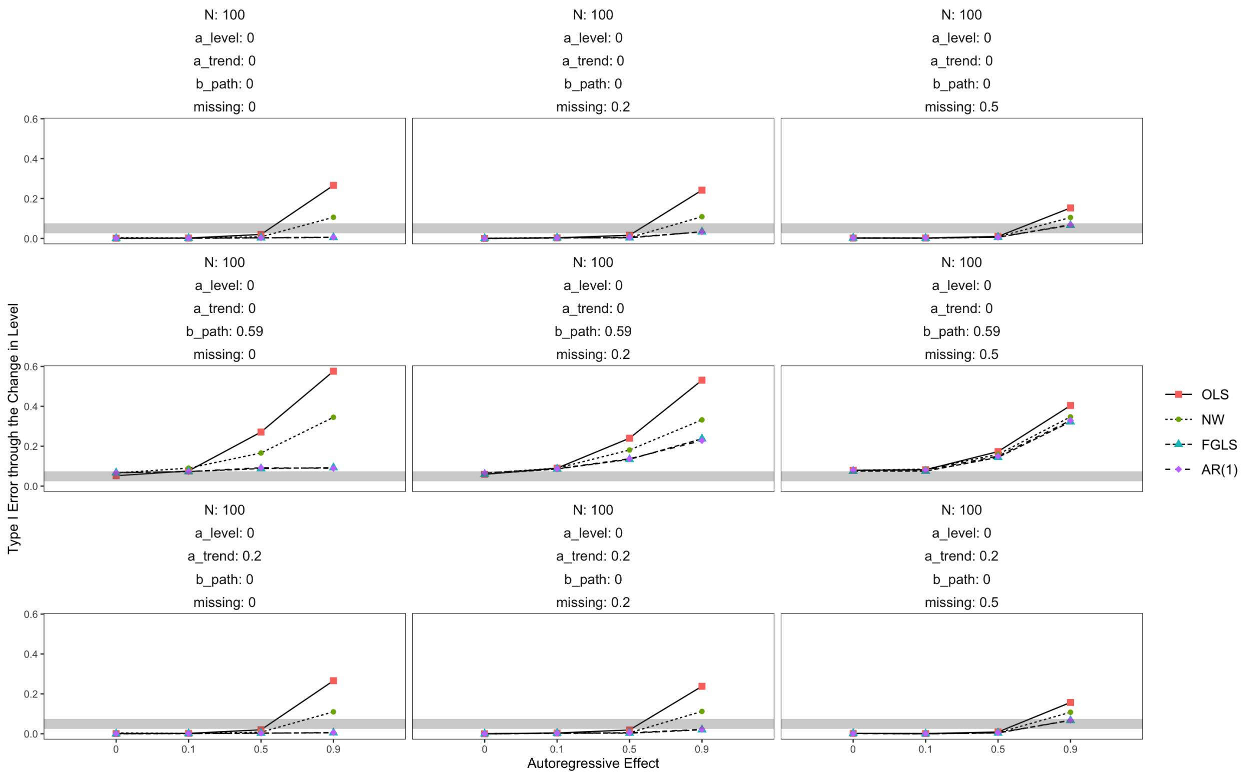

N: 100
a_level: 0
a_trend: 0
b_path: 0
missing: 0
N: 100
a_level: 0
a_trend: 0
b_path: 0
missing: 0.2
N: 100
a_level: 0
a_trend: 0
b_path: 0
missing: 0.5
N: 100
a_level: 0
a_trend: 0
b_path: 0.59
missing: 0
N: 100
a_level: 0
a_trend: 0
b_path: 0.59
missing: 0.2
N: 100
a_level: 0
a_trend: 0
b_path: 0.59
missing: 0.5
N: 100
a_level: 0
a_trend: 0.2
b_path: 0
missing: 0
N: 100
a_level: 0
a_trend: 0.2
b_path: 0
missing: 0.2
N: 100
a_level: 0
a_trend: 0.2
b_path: 0
missing: 0.5
Type I Error through the Change in Level
Autoregressive Effect
0.0
0.2
0.4
0.6
0
0.1
0.5
0.9
OLS
NW
FGLS
AR(1)

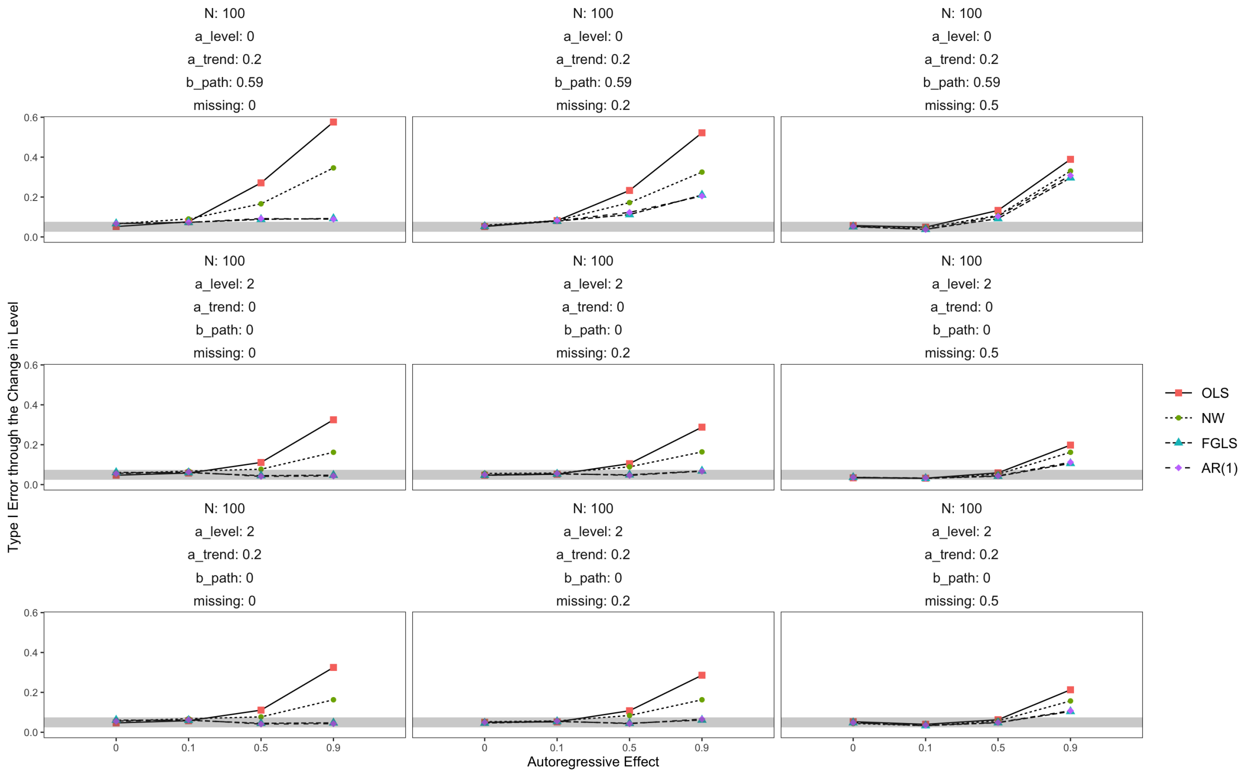

N: 100
a_level: 0
a_trend: 0.2
b_path: 0.59
missing: 0
N: 100
a_level: 0
a_trend: 0.2
b_path: 0.59
missing: 0.2
N: 100
a_level: 0
a_trend: 0.2
b_path: 0.59
missing: 0.5
N: 100
a_level: 2
a_trend: 0
b_path: 0
missing: 0
N: 100
a_level: 2
a_trend: 0
b_path: 0
missing: 0.2
N: 100
a_level: 2
a_trend: 0
b_path: 0
missing: 0.5
N: 100
a_level: 2
a_trend: 0.2
b_path: 0
missing: 0
N: 100
a_level: 2
a_trend: 0.2
b_path: 0
missing: 0.2
N: 100
a_level: 2
a_trend: 0.2
b_path: 0
missing: 0.5
Type I Error through the Change in Level
Autoregressive Effect
0.0
0.2
0.4
0.6
0
0.1
0.5
0.9
OLS
NW
FGLS
AR(1)

Type I Error of the Interval Estimate through the Change in Trend

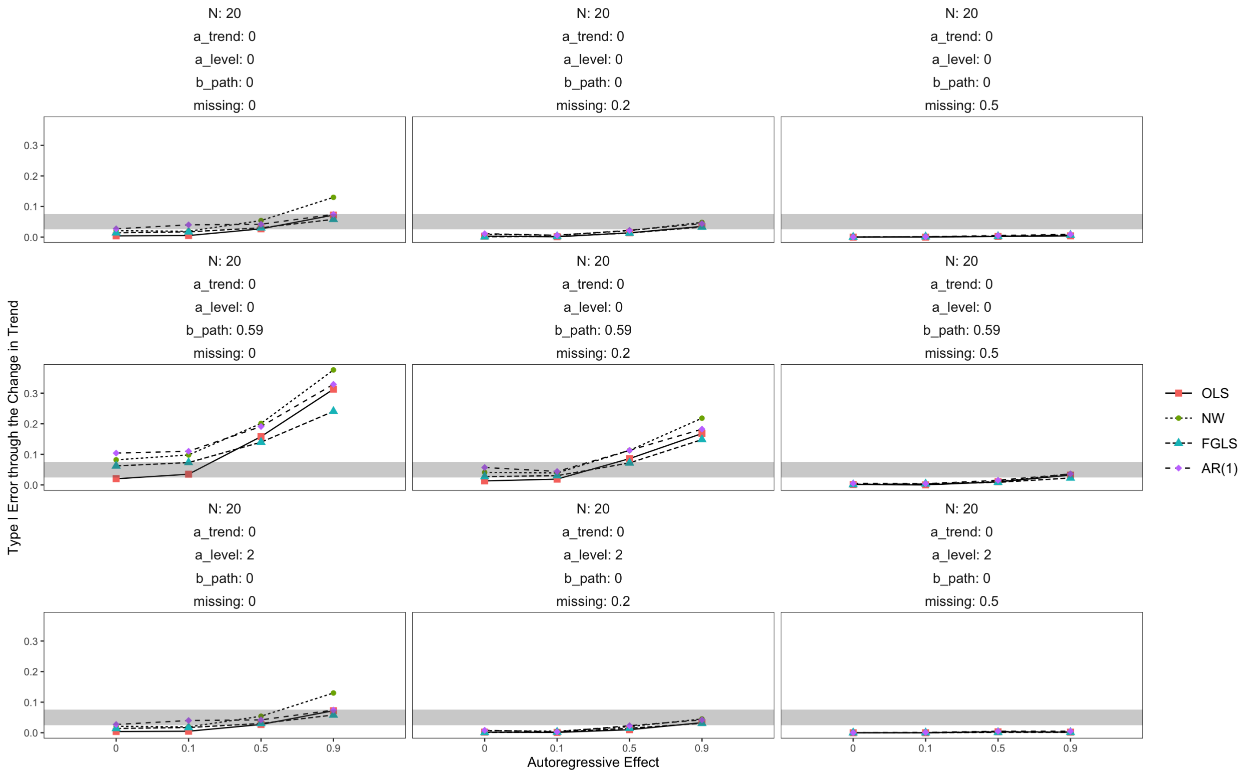

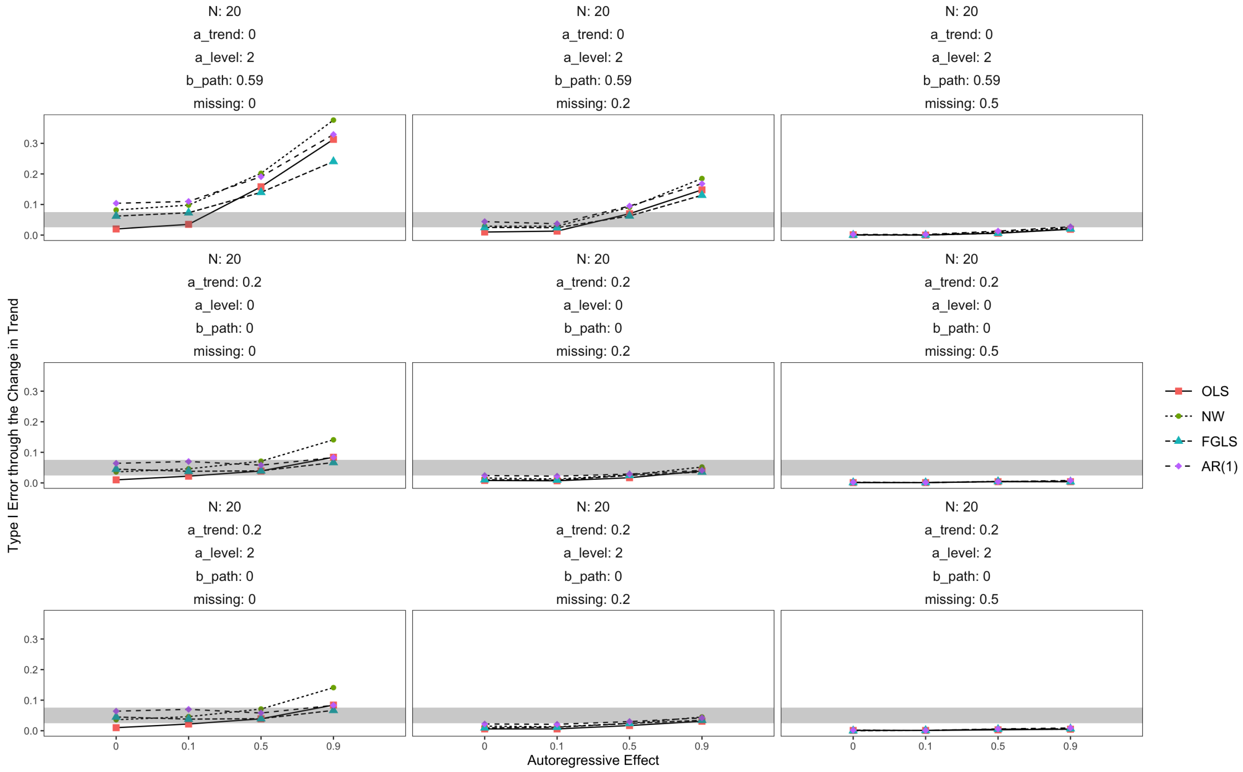

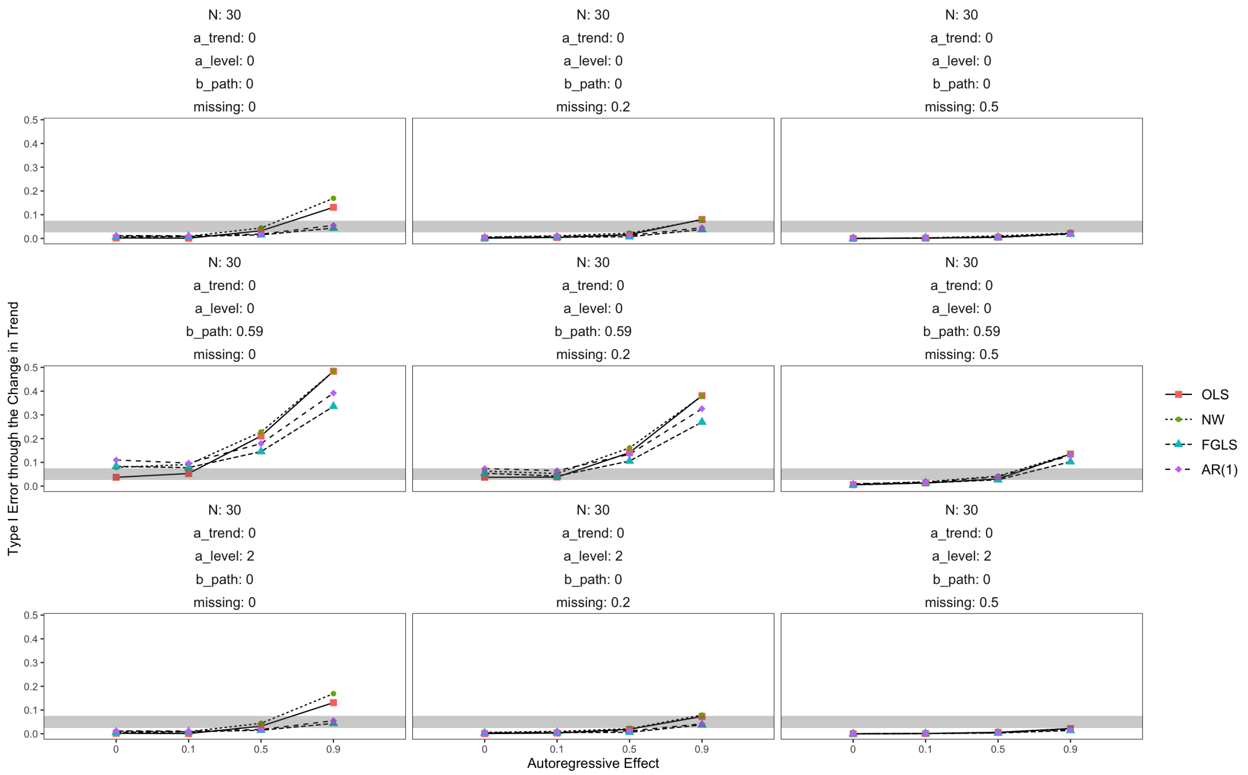
N: 30
a_trend: 0
a_level: 0
b_path: 0
missing: 0
N: 30
a_trend: 0
a_level: 0
b_path: 0
missing: 0.2
N: 30
a_trend: 0
a_level: 0
b_path: 0
missing: 0.5
N: 30
a_trend: 0
a_level: 0
b_path: 0.59
missing: 0
N: 30
a_trend: 0
a_level: 0
b_path: 0.59
missing: 0.2
N: 30
a_trend: 0
a_level: 0
b_path: 0.59
missing: 0.5
N: 30
a_trend: 0
a_level: 2
b_path: 0
missing: 0
N: 30
a_trend: 0
a_level: 2
b_path: 0
missing: 0.2
N: 30
a_trend: 0
a_level: 2
b_path: 0
missing: 0.5
Type I Error through the Change in Trend
0.0
0.1
0.2
0.3
0.4
0.5
0
0.1
0.5
0.9
Autoregressive Effect
OLS
NW
FGLS
AR(1)

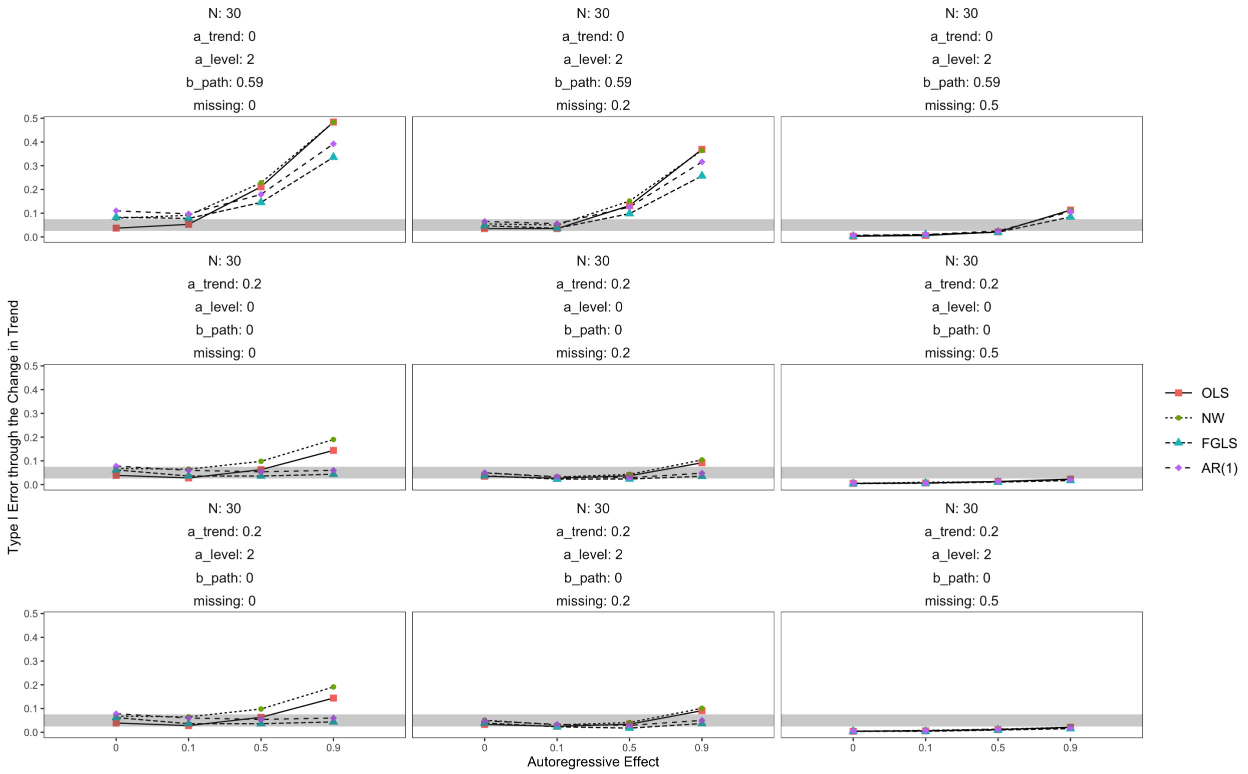
N: 30
a_trend: 0
a_level: 2
b_path: 0.59
missing: 0
N: 30
a_trend: 0
a_level: 2
b_path: 0.59
missing: 0.2
N: 30
a_trend: 0
a_level: 2
b_path: 0.59
missing: 0.5
N: 30
a_trend: 0.2
a_level: 0
b_path: 0
missing: 0
N: 30
a_trend: 0.2
a_level: 0
b_path: 0
missing: 0.2
N: 30
a_trend: 0.2
a_level: 0
b_path: 0
missing: 0.5
N: 30
a_trend: 0.2
a_level: 2
b_path: 0
missing: 0
N: 30
a_trend: 0.2
a_level: 2
b_path: 0
missing: 0.2
N: 30
a_trend: 0.2
a_level: 2
b_path: 0
missing: 0.5
Type I Error through the Change in Trend
0.0
0.1
0.2
0.3
0.4
0.5
0
0.1
0.5
0.9
Autoregressive Effect
OLS
NW
FGLS
AR(1)

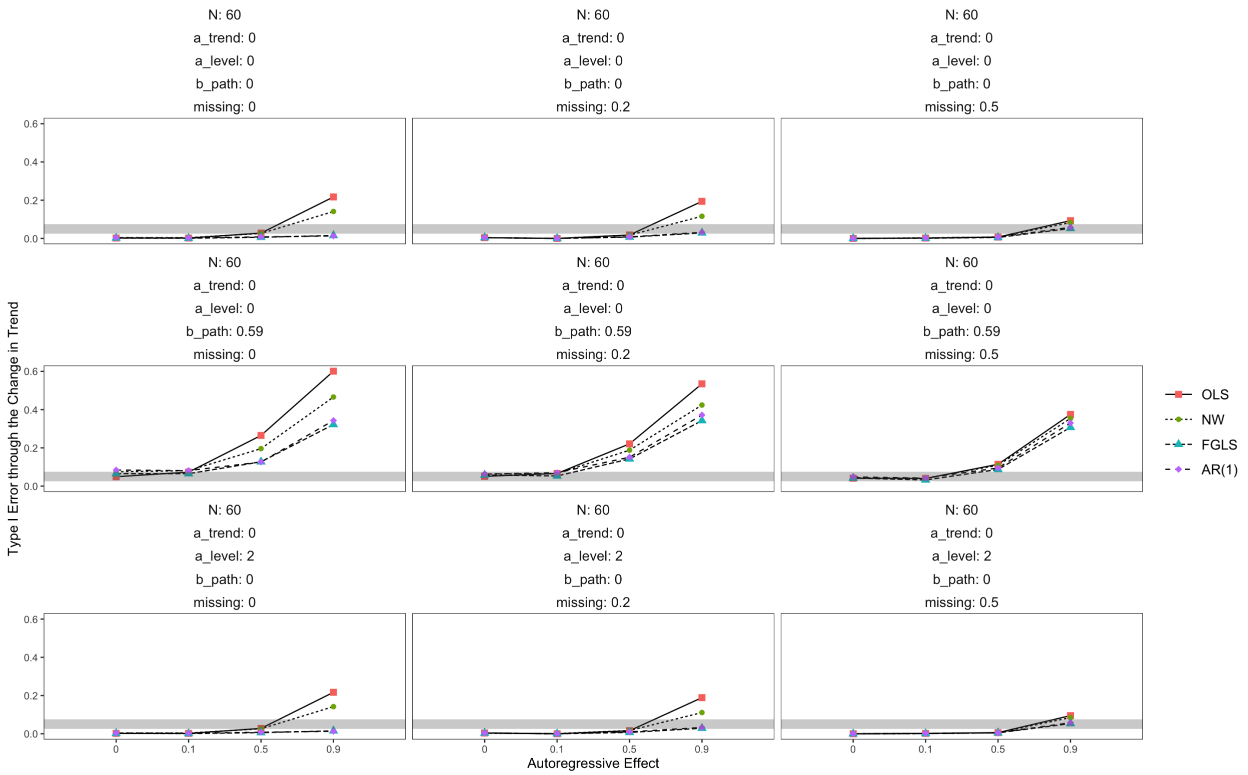
N: 60
a_trend: 0
a_level: 0
b_path: 0
missing: 0
missing: 0.2
missing: 0.5
b_path: 0.59
a_level: 2
Type I Error through the Change in Trend
Autoregressive Effect
0.0
0.2
0.4
0.6
0
0.1
0.5
0.9
OLS
NW
FGLS
AR(1)

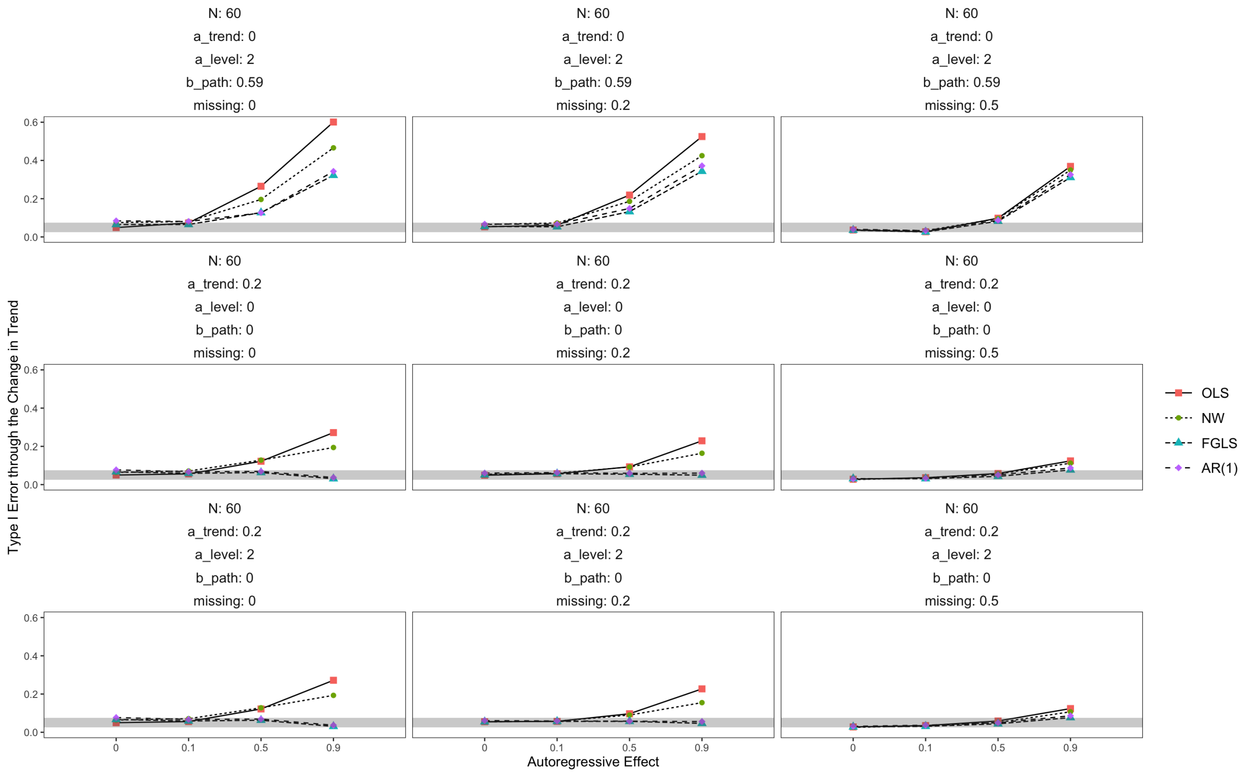
N: 60
a_trend: 0
a_level: 2
b_path: 0.59
missing: 0
missing: 0.2
missing: 0.5
a_trend: 0.2
a_level: 0
b_path: 0
Type I Error through the Change in Trend
Autoregressive Effect
0.0
0.2
0.4
0.6
0
0.1
0.5
0.9
OLS
NW
FGLS
AR(1)

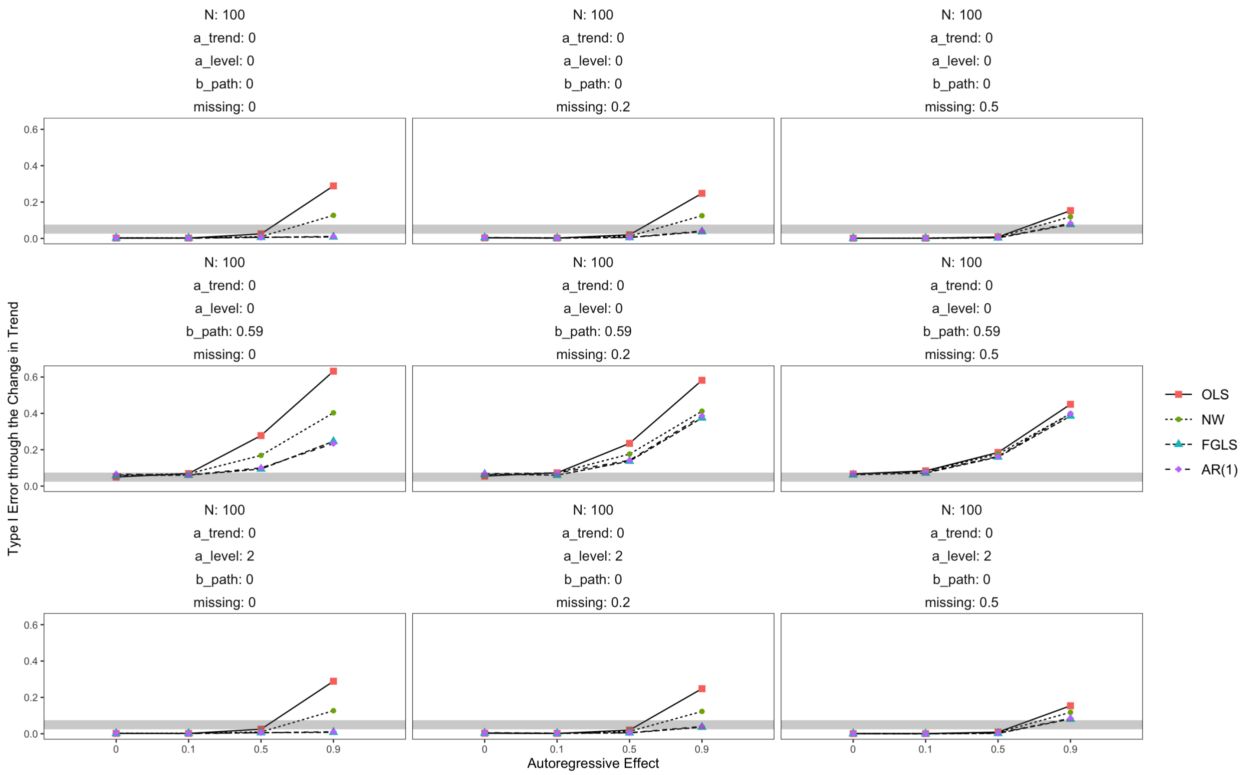
N: 100
a_trend: 0
a_level: 0
b_path: 0
missing: 0
N: 100
a_trend: 0
a_level: 0
b_path: 0
missing: 0.2
N: 100
a_trend: 0
a_level: 0
b_path: 0
missing: 0.5
N: 100
a_trend: 0
a_level: 0
b_path: 0.59
missing: 0
N: 100
a_trend: 0
a_level: 0
b_path: 0.59
missing: 0.2
N: 100
a_trend: 0
a_level: 0
b_path: 0.59
missing: 0.5
N: 100
a_trend: 0
a_level: 2
b_path: 0
missing: 0
N: 100
a_trend: 0
a_level: 2
b_path: 0
missing: 0.2
N: 100
a_trend: 0
a_level: 2
b_path: 0
missing: 0.5
Type I Error through the Change in Trend
0.0
0.2
0.4
0.6
0
0.1
0.5
0.9
Autoregressive Effect
OLS
NW
FGLS
AR(1)

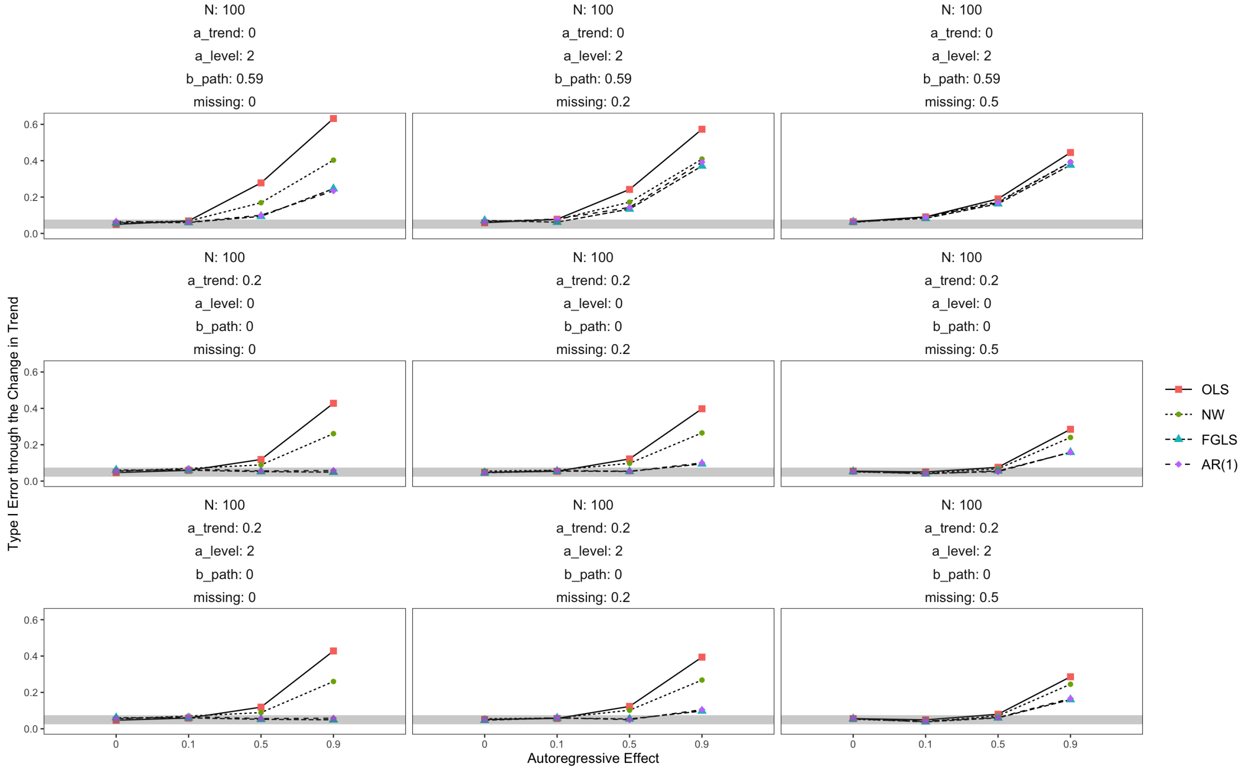
N: 100
a_trend: 0
a_level: 2
b_path: 0.59
missing: 0
N: 100
a_trend: 0
a_level: 2
b_path: 0.59
missing: 0.2
N: 100
a_trend: 0
a_level: 2
b_path: 0.59
missing: 0.5
N: 100
a_trend: 0.2
a_level: 0
b_path: 0
missing: 0
N: 100
a_trend: 0.2
a_level: 0
b_path: 0
missing: 0.2
N: 100
a_trend: 0.2
a_level: 0
b_path: 0
missing: 0.5
N: 100
a_trend: 0.2
a_level: 2
b_path: 0
missing: 0
N: 100
a_trend: 0.2
a_level: 2
b_path: 0
missing: 0.2
N: 100
a_trend: 0.2
a_level: 2
b_path: 0
missing: 0.5
Type I Error through the Change in Trend
0.0
0.2
0.4
0.6
0
0.1
0.5
0.9
Autoregressive Effect
OLS
NW
FGLS
AR(1)

Coverage of the Indirect Effect through the Change in Level

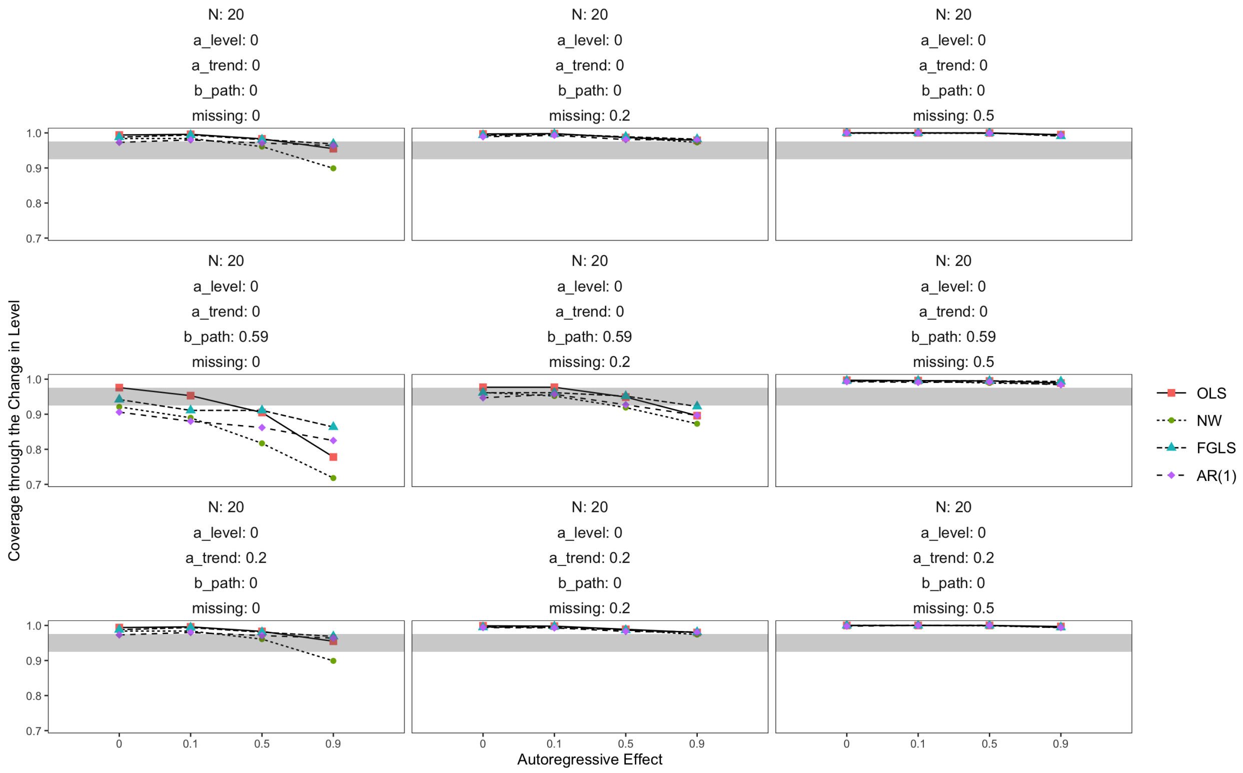

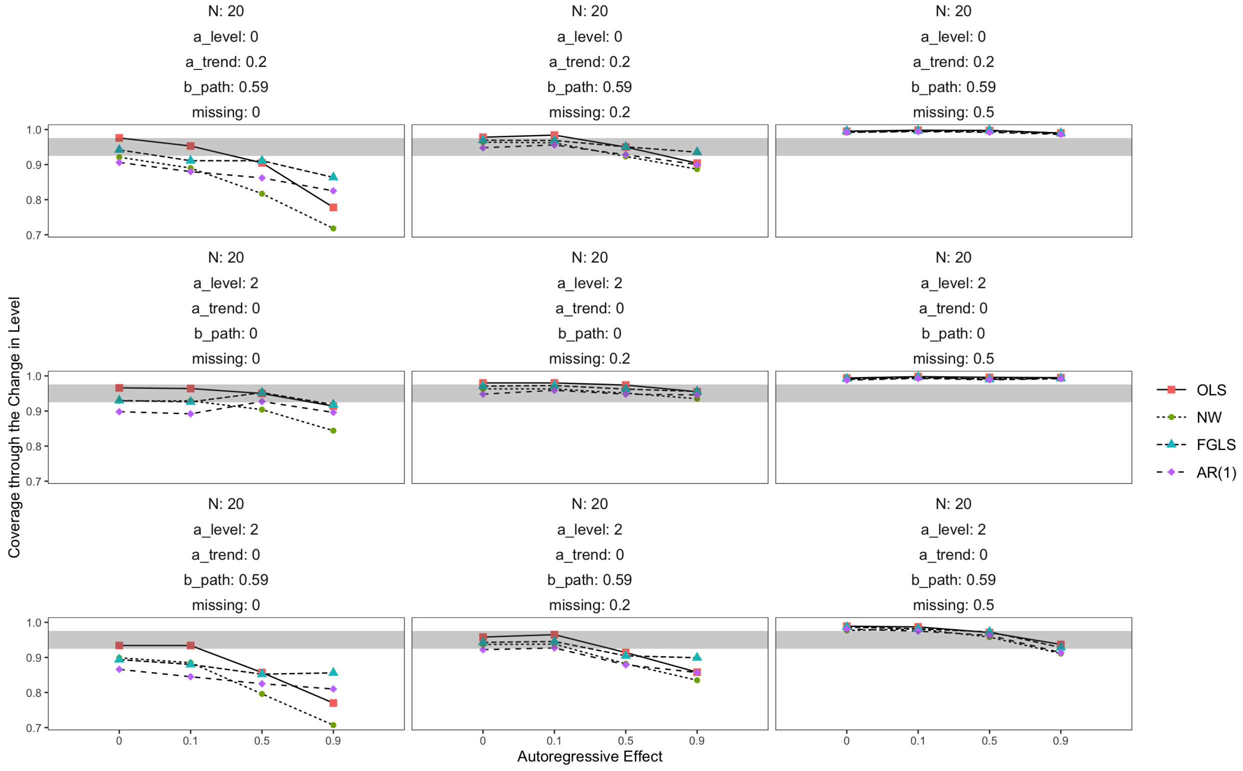

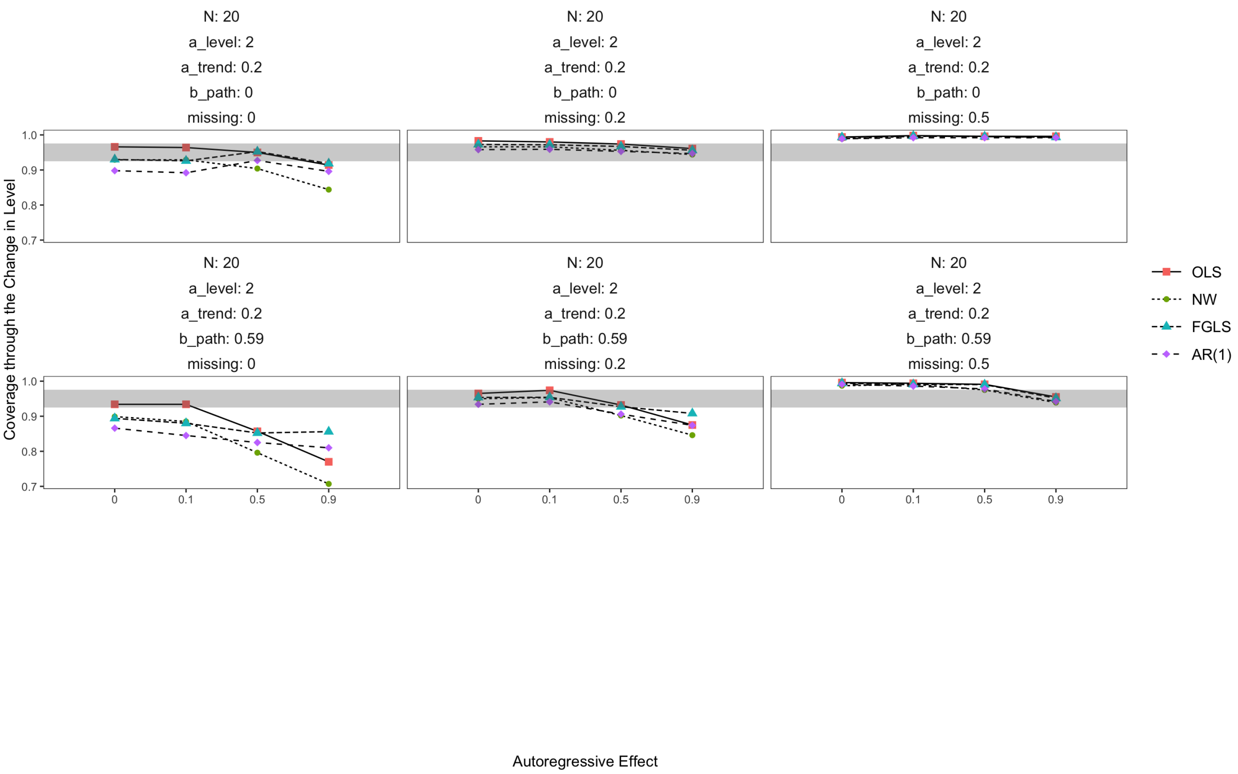
N: 20
a_level: 2
a_trend: 0.2
b_path: 0
missing: 0
missing: 0.2
missing: 0.5
b_path: 0.59
Coverage through the Change in Level
1.0
0.9
0.8
0.7
0
0.1
0.5
0.9
OLS
NW
FGLS
AR(1)
Autoregressive Effect

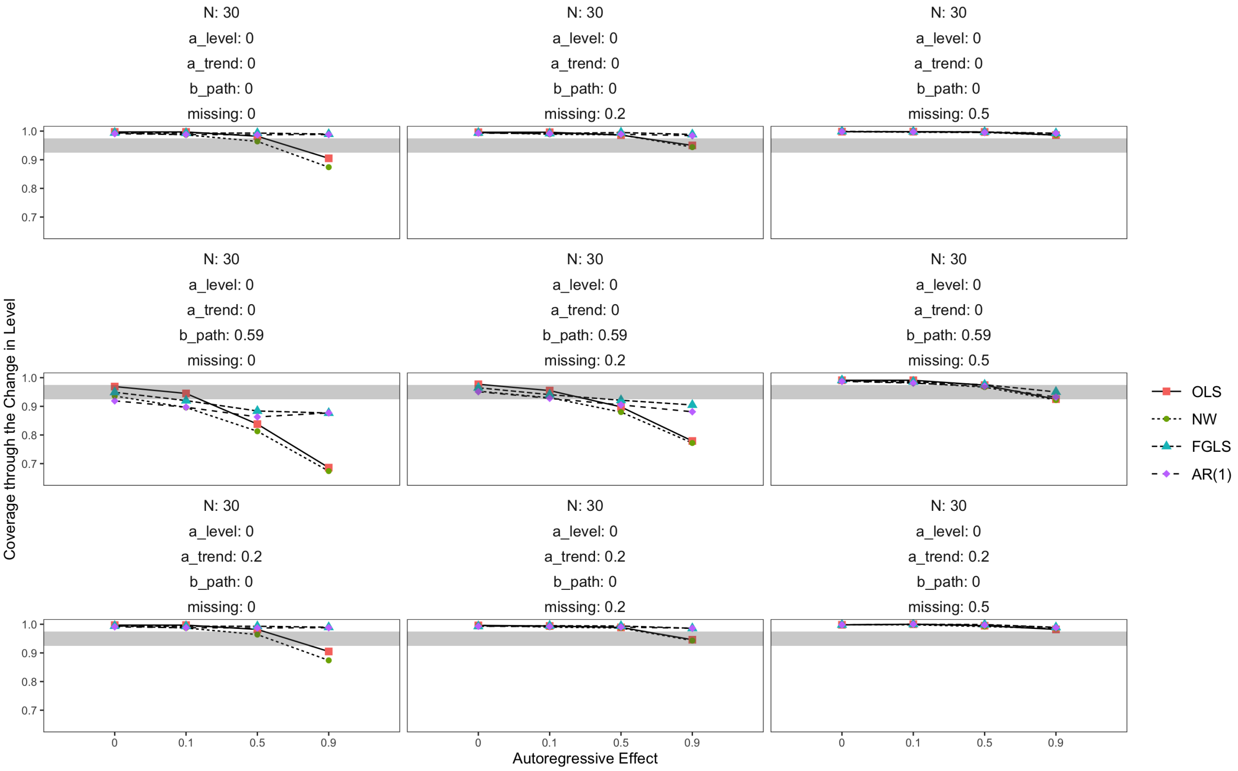
N: 30
a_level: 0
a_trend: 0
a_trend: 0.2
b_path: 0
b_path: 0.59
missing: 0
missing: 0.2
missing: 0.5
Coverage through the Change in Level
1.0
0.9
0.8
0.7
0
0.1
0.5
0.9
OLS
NW
FGLS
AR(1)
Autoregressive Effect

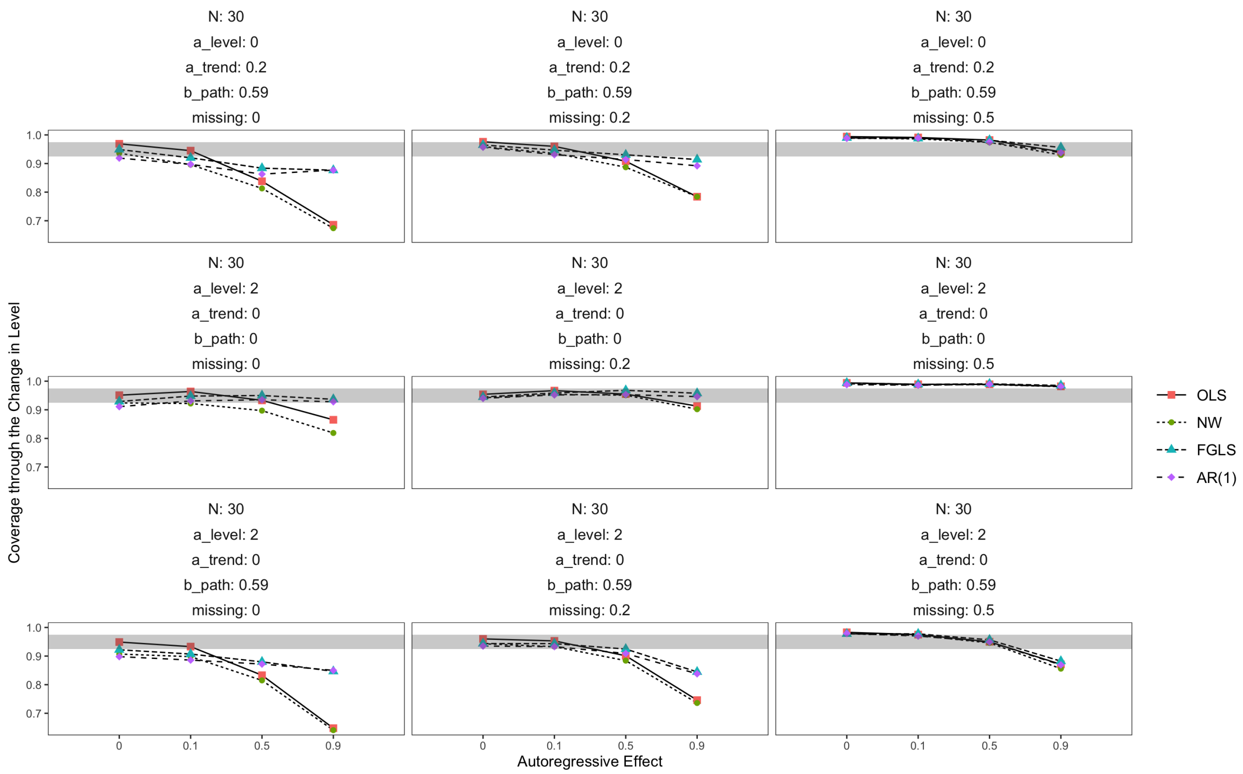
N: 30
a_level: 0
a_trend: 0.2
b_path: 0.59
missing: 0
N: 30
a_level: 0
a_trend: 0.2
b_path: 0.59
missing: 0.2
N: 30
a_level: 0
a_trend: 0.2
b_path: 0.59
missing: 0.5
N: 30
a_level: 2
a_trend: 0
b_path: 0
missing: 0
N: 30
a_level: 2
a_trend: 0
b_path: 0
missing: 0.2
N: 30
a_level: 2
a_trend: 0
b_path: 0
missing: 0.5
N: 30
a_level: 2
a_trend: 0
b_path: 0.59
missing: 0
N: 30
a_level: 2
a_trend: 0
b_path: 0.59
missing: 0.2
N: 30
a_level: 2
a_trend: 0
b_path: 0.59
missing: 0.5
Coverage through the Change in Level
1.0
0.9
0.8
0.7
0
0.1
0.5
0.9
Autoregressive Effect
OLS
NW
FGLS
AR(1)

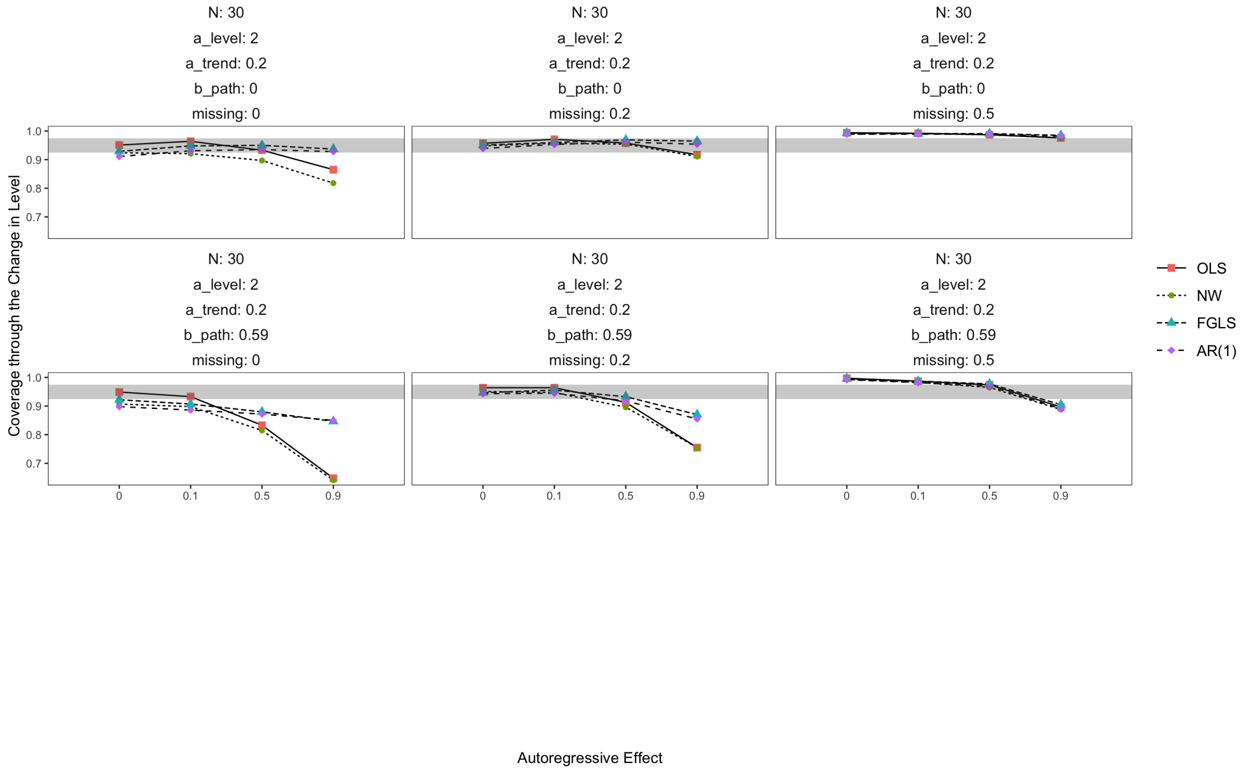
N: 30
a_level: 2
a_trend: 0.2
b_path: 0
missing: 0
N: 30
a_level: 2
a_trend: 0.2
b_path: 0
missing: 0.2
N: 30
a_level: 2
a_trend: 0.2
b_path: 0
missing: 0.5
N: 30
a_level: 2
a_trend: 0.2
b_path: 0.59
missing: 0
N: 30
a_level: 2
a_trend: 0.2
b_path: 0.59
missing: 0.2
N: 30
a_level: 2
a_trend: 0.2
b_path: 0.59
missing: 0.5
Coverage through the Change in Level
1.0
0.9
0.8
0.7
0
0.1
0.5
0.9
Autoregressive Effect
OLS
NW
FGLS
AR(1)

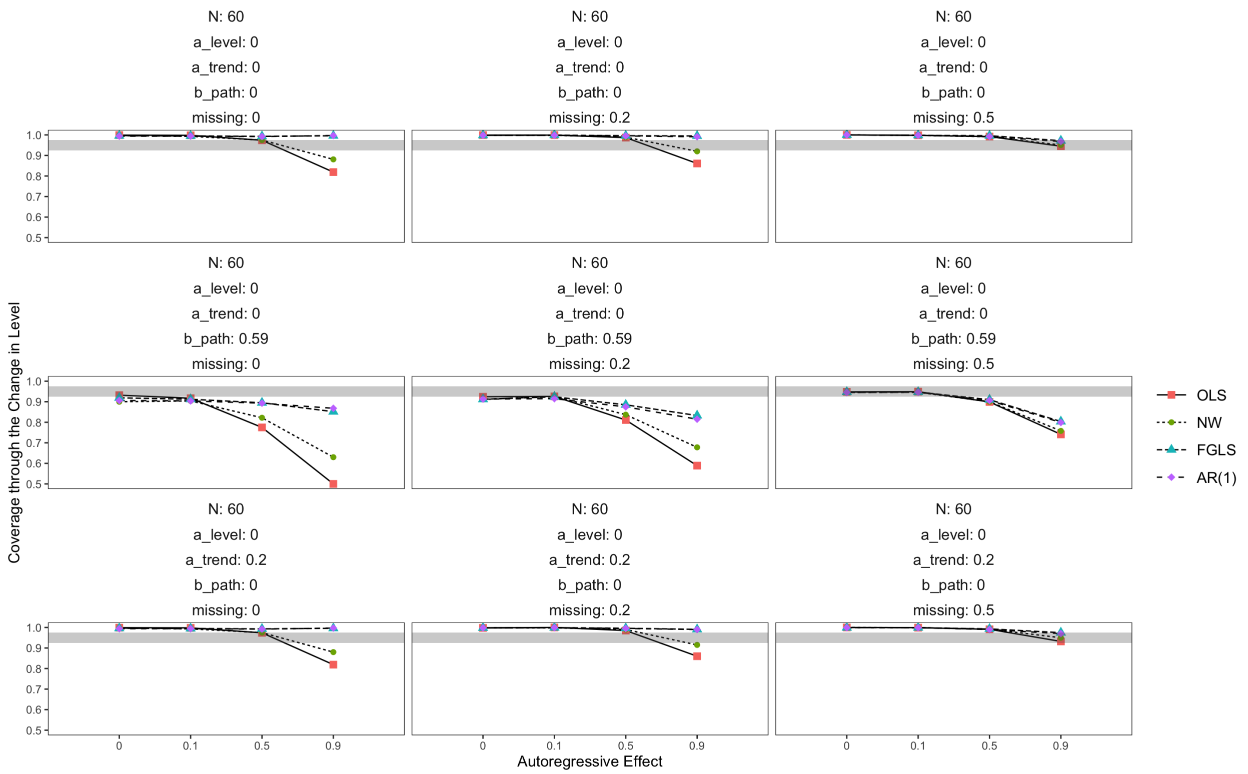

N: 60
a_level: 0
a_trend: 0
b_path: 0
missing: 0
N: 60
a_level: 0
a_trend: 0
b_path: 0
missing: 0.2
N: 60
a_level: 0
a_trend: 0
b_path: 0
missing: 0.5
N: 60
a_level: 0
a_trend: 0
b_path: 0.59
missing: 0
N: 60
a_level: 0
a_trend: 0
b_path: 0.59
missing: 0.2
N: 60
a_level: 0
a_trend: 0
b_path: 0.59
missing: 0.5
N: 60
a_level: 0
a_trend: 0.2
b_path: 0
missing: 0
N: 60
a_level: 0
a_trend: 0.2
b_path: 0
missing: 0.2
N: 60
a_level: 0
a_trend: 0.2
b_path: 0
missing: 0.5
1.0
0.9
0.8
0.7
0.6
0.5
0
0.1
0.5
0.9
Coverage through the Change in Level
Autoregressive Effect
OLS
NW
FGLS
AR(1)

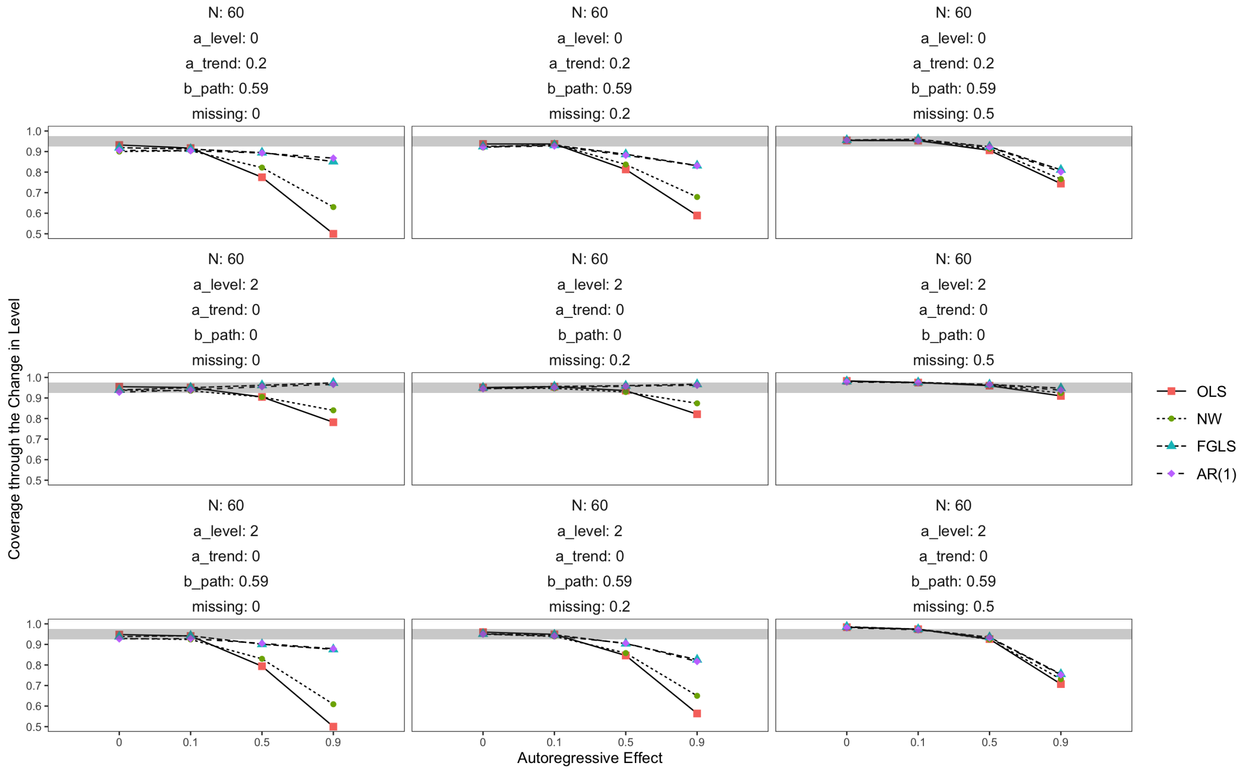

N: 60
a_level: 0
a_trend: 0.2
b_path: 0.59
missing: 0
N: 60
a_level: 0
a_trend: 0.2
b_path: 0.59
missing: 0.2
N: 60
a_level: 0
a_trend: 0.2
b_path: 0.59
missing: 0.5
N: 60
a_level: 2
a_trend: 0
b_path: 0
missing: 0
N: 60
a_level: 2
a_trend: 0
b_path: 0
missing: 0.2
N: 60
a_level: 2
a_trend: 0
b_path: 0
missing: 0.5
N: 60
a_level: 2
a_trend: 0
b_path: 0.59
missing: 0
N: 60
a_level: 2
a_trend: 0
b_path: 0.59
missing: 0.2
N: 60
a_level: 2
a_trend: 0
b_path: 0.59
missing: 0.5
1.0
0.9
0.8
0.7
0.6
0.5
0
0.1
0.5
0.9
Coverage through the Change in Level
Autoregressive Effect
OLS
NW
FGLS
AR(1)

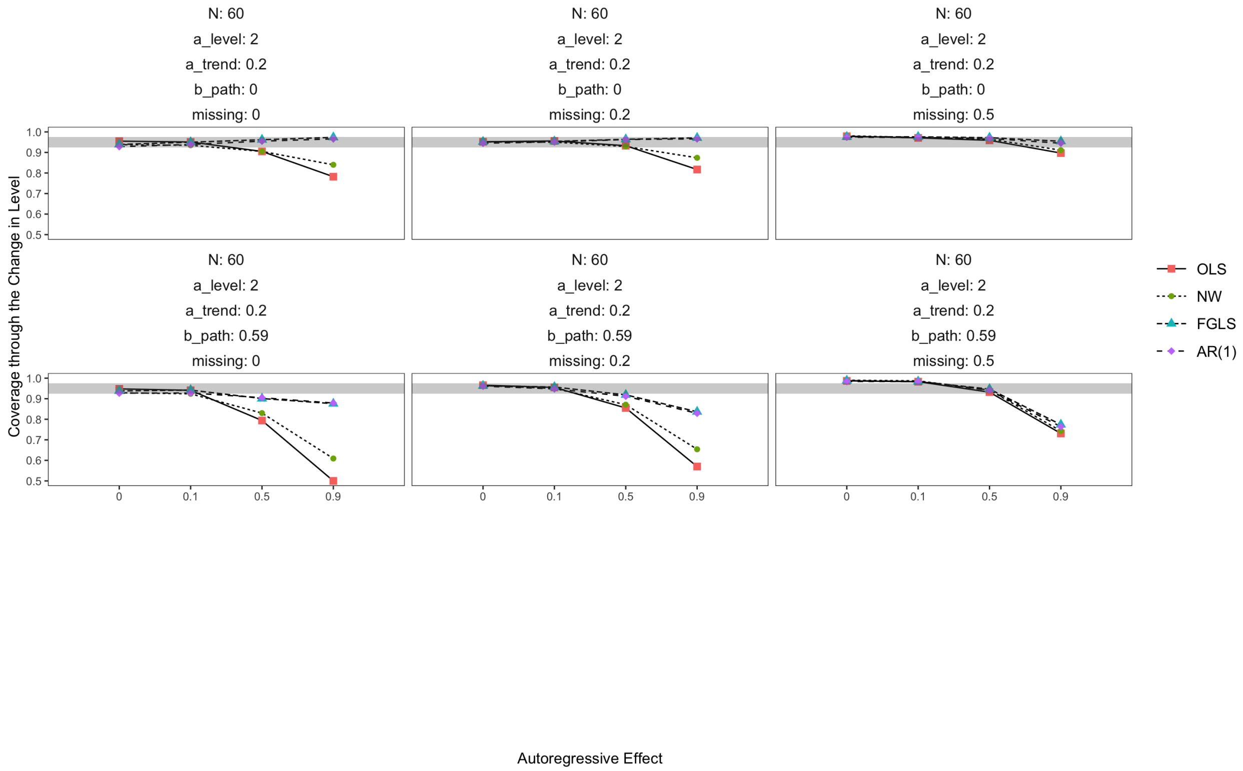
N: 60
a_level: 2
a_trend: 0.2
b_path: 0
missing: 0
missing: 0.2
missing: 0.5
b_path: 0.59
Coverage through the Change in Level
1.0
0.9
0.8
0.7
0.6
0.5
0
0.1
0.5
0.9
OLS
NW
FGLS
AR(1)
Autoregressive Effect

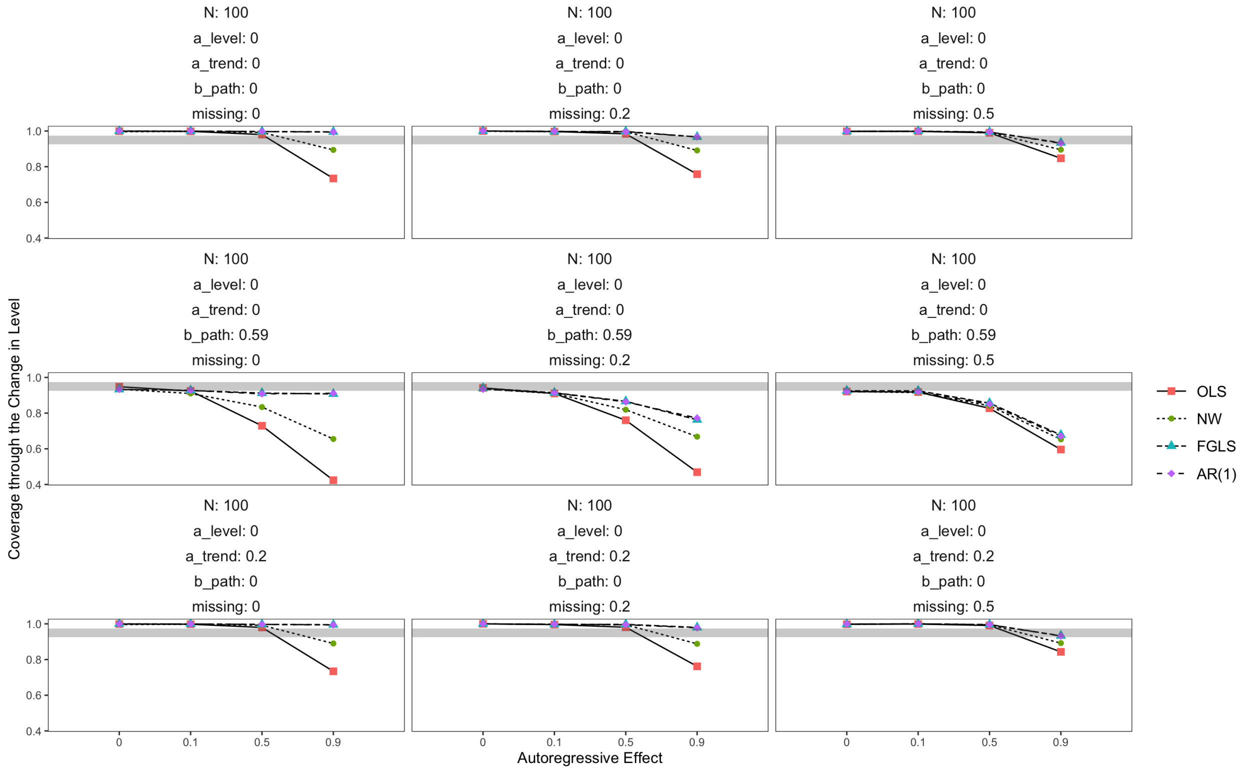
N: 100
a_level: 0
a_trend: 0
a_trend: 0.2
b_path: 0
b_path: 0.59
missing: 0
missing: 0.2
missing: 0.5
Coverage through the Change in Level
1.0
0.8
0.6
0.4
0
0.1
0.5
0.9
OLS
NW
FGLS
AR(1)
Autoregressive Effect

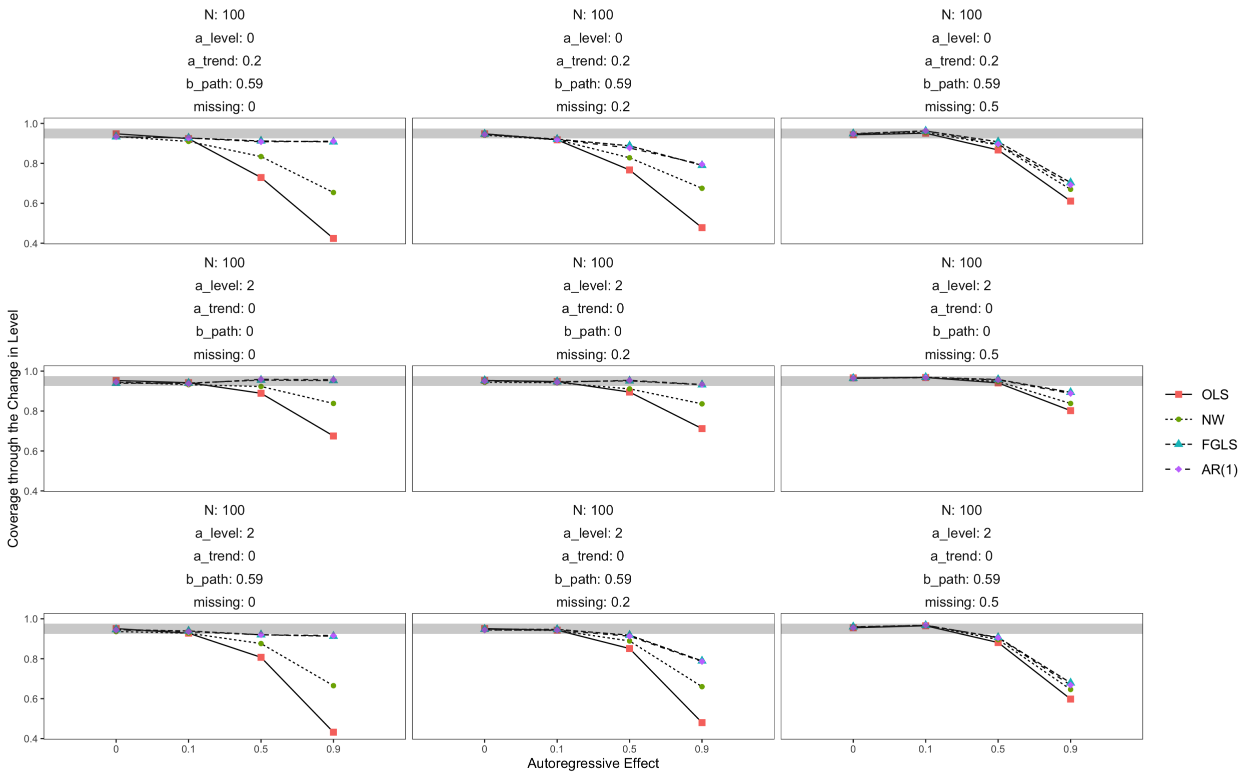

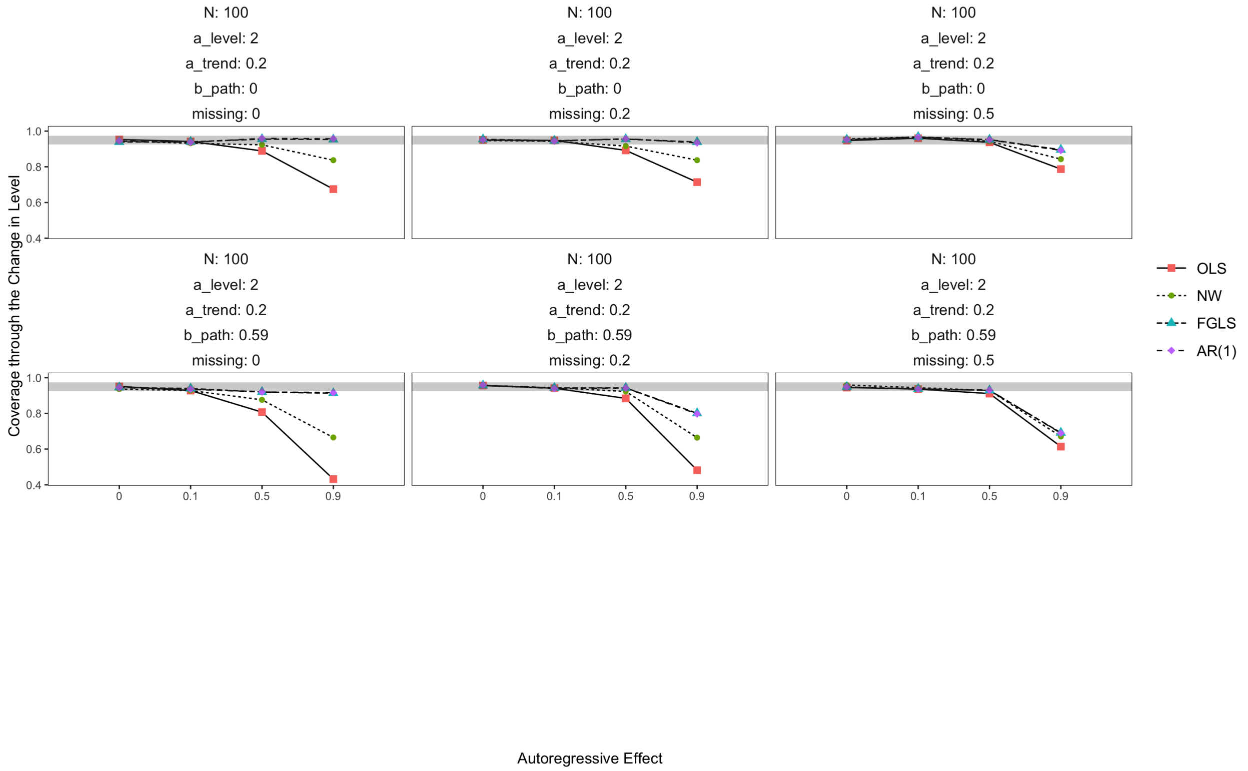

Coverage of the Indirect Effect through the Change in Trend

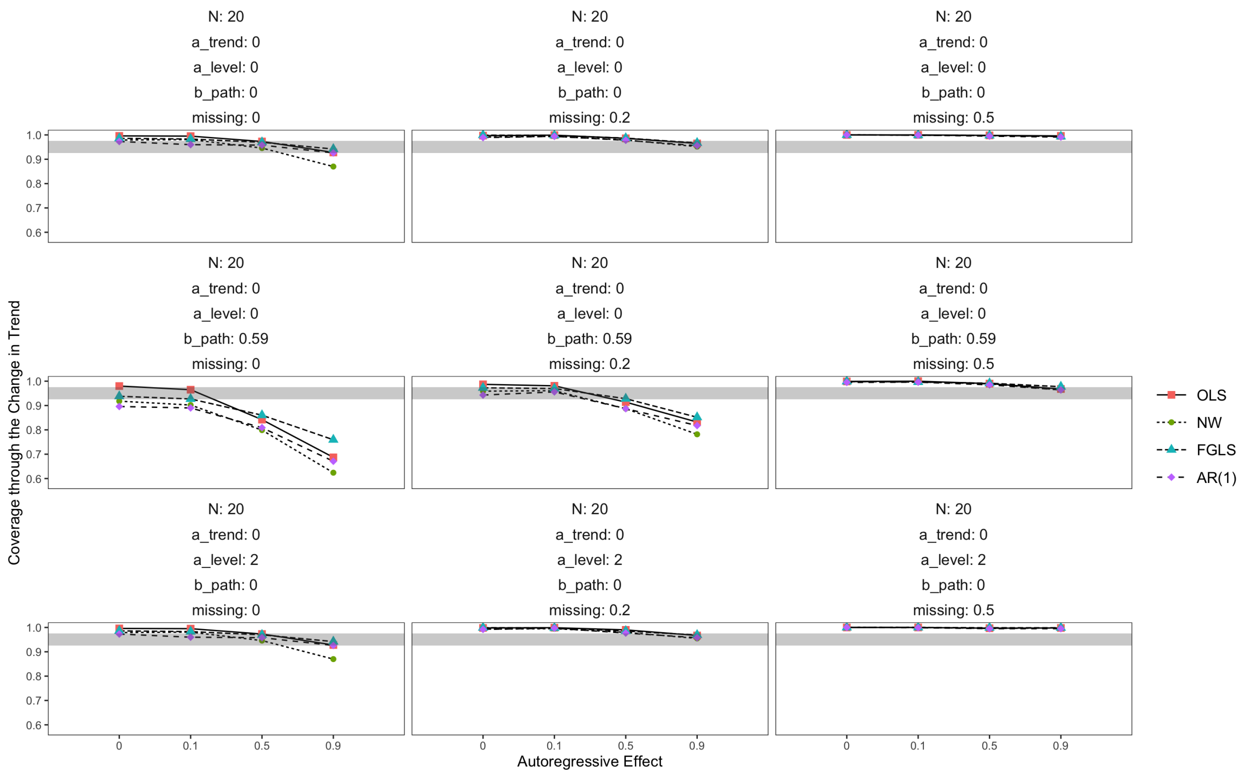
N: 20
a_trend: 0
a_level: 0
b_path: 0
missing: 0
missing: 0.2
missing: 0.5
b_path: 0.59
a_level: 2
Coverage through the Change in Trend
Autoregressive Effect
0
0.1
0.5
0.9
1.0
0.9
0.8
0.7
0.6
OLS
NW
FGLS
AR(1)

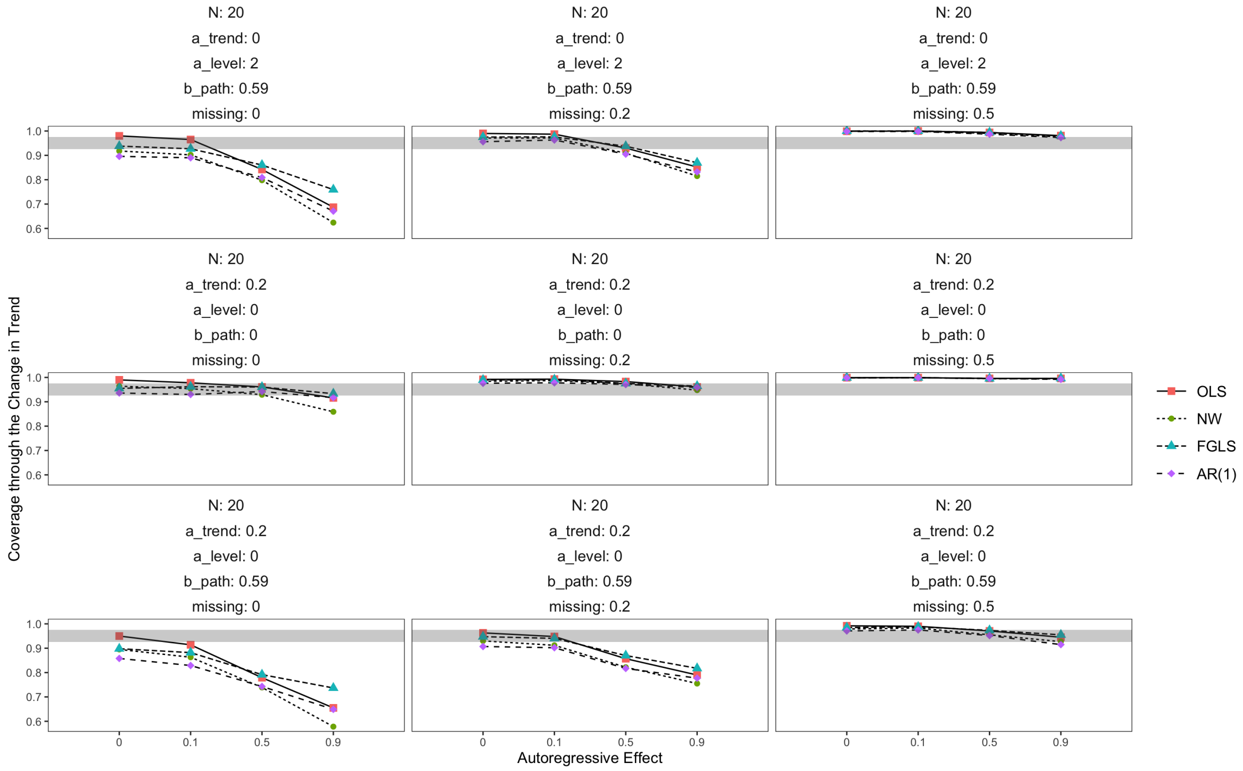
N: 20
a_trend: 0
a_level: 2
b_path: 0.59
missing: 0
missing: 0.2
missing: 0.5
a_trend: 0.2
a_level: 0
b_path: 0
Coverage through the Change in Trend
Autoregressive Effect
0
0.1
0.5
0.9
1.0
0.9
0.8
0.7
0.6
OLS
NW
FGLS
AR(1)

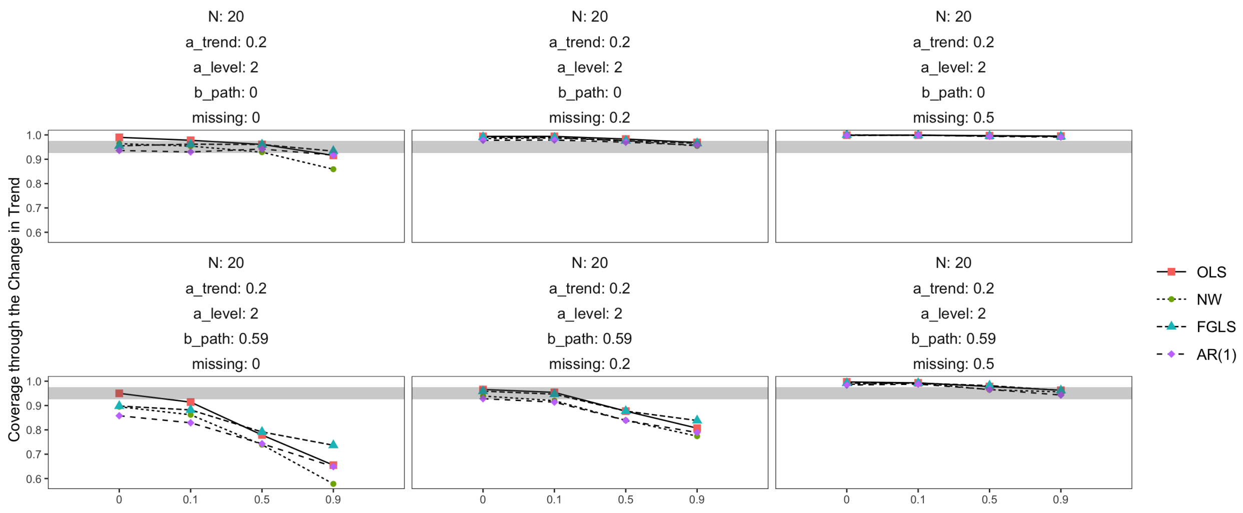

Autoregressive Effect

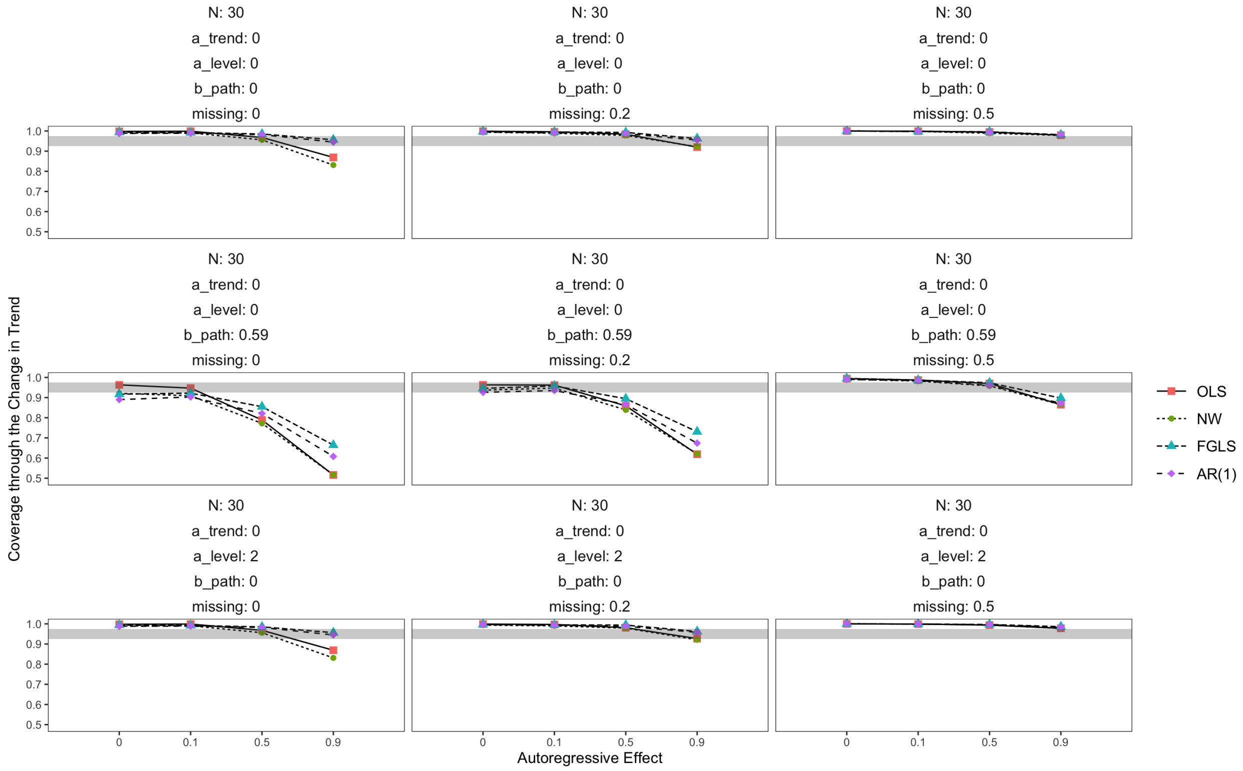

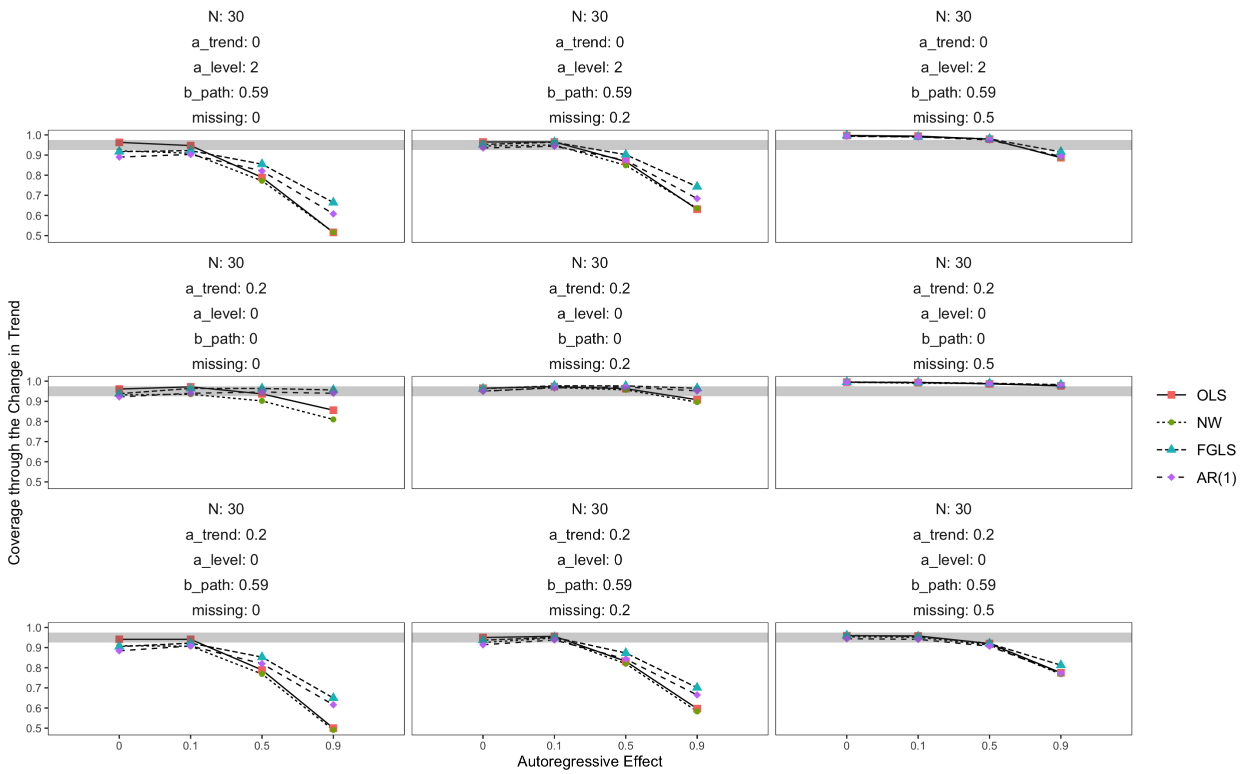
N: 30
a_trend: 0
a_level: 2
b_path: 0.59
missing: 0
missing: 0.2
missing: 0.5
a_trend: 0.2
a_level: 0
b_path: 0
b_path: 0.59
Coverage through the Change in Trend
Autoregressive Effect
OLS
NW
FGLS
AR(1)

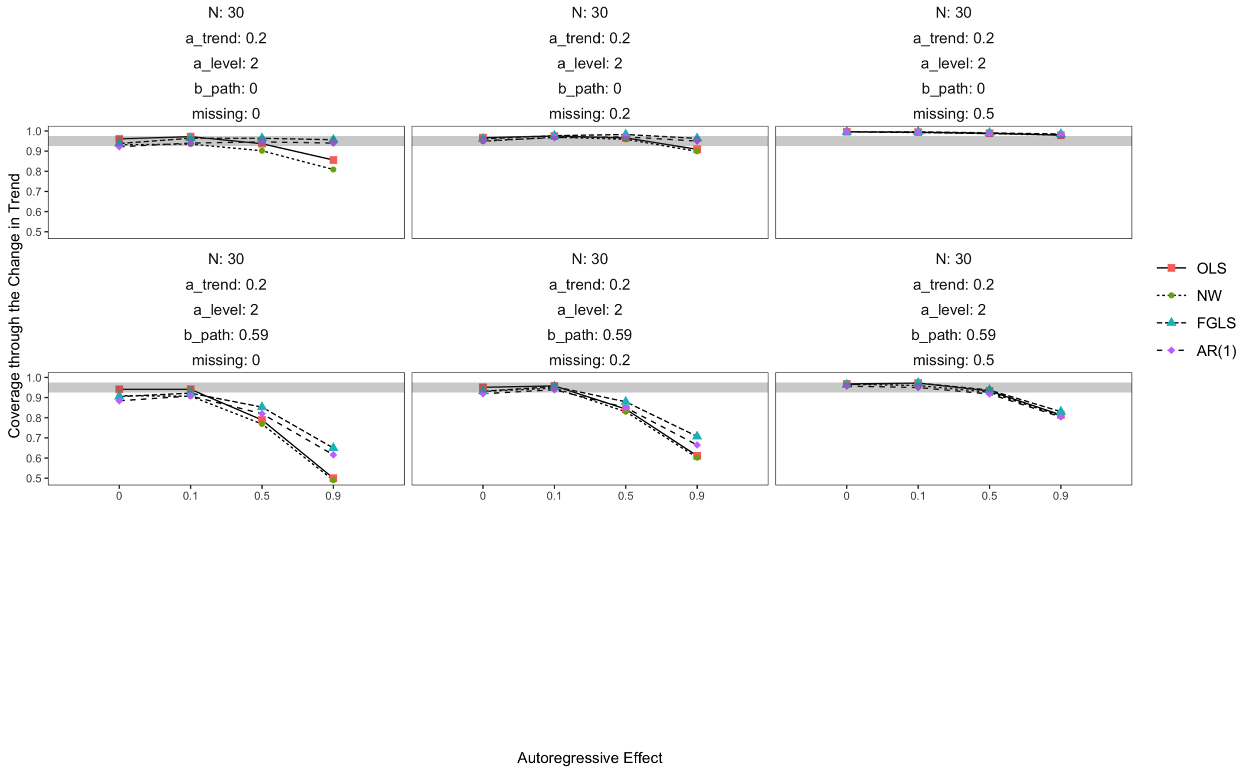
N: 30
a_trend: 0.2
a_level: 2
b_path: 0
b_path: 0.59
missing: 0
missing: 0.2
missing: 0.5
Coverage through the Change in Trend
Autoregressive Effect
OLS
NW
FGLS
AR(1)

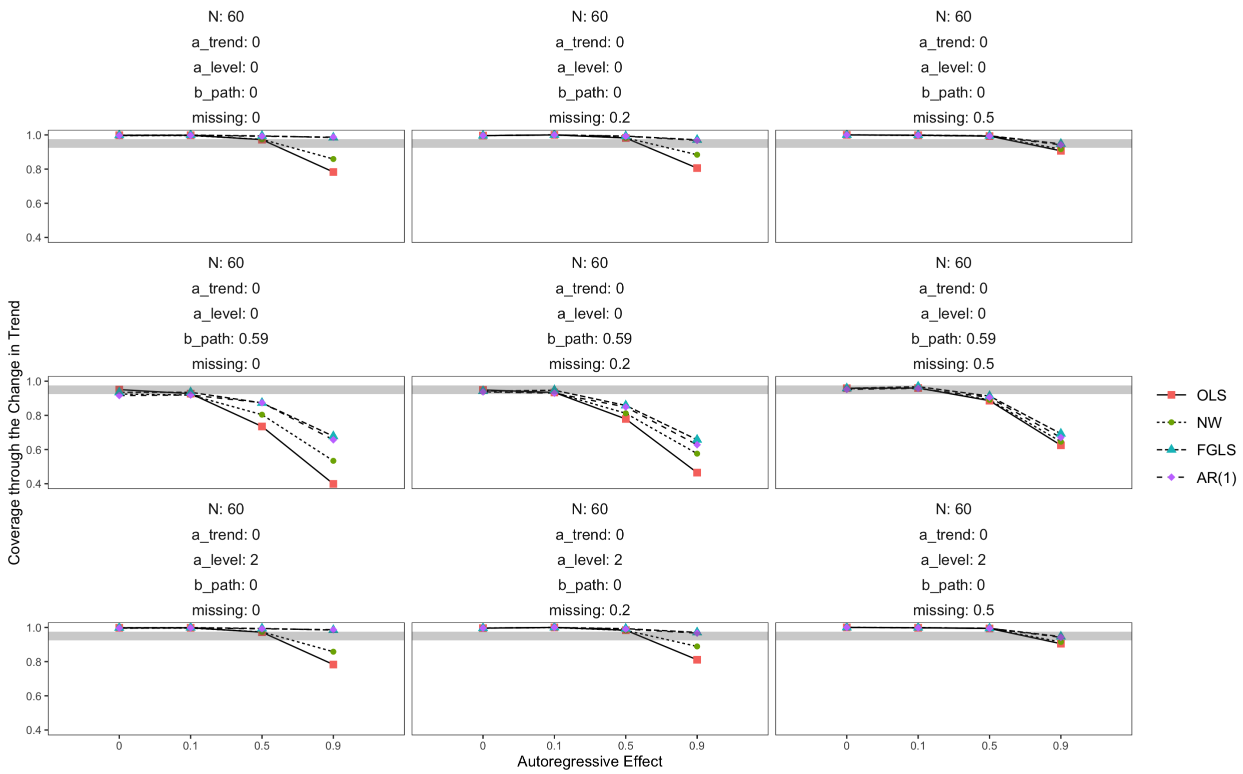
N: 60 a_trend: 0 a_level: 0 b_path: 0 missing: 0
N: 60 a_trend: 0 a_level: 0 b_path: 0 missing: 0.2
N: 60 a_trend: 0 a_level: 0 b_path: 0 missing: 0.5
N: 60 a_trend: 0 a_level: 0 b_path: 0.59 missing: 0
N: 60 a_trend: 0 a_level: 0 b_path: 0.59 missing: 0.2
N: 60 a_trend: 0 a_level: 0 b_path: 0.59 missing: 0.5
N: 60 a_trend: 0 a_level: 2 b_path: 0 missing: 0
N: 60 a_trend: 0 a_level: 2 b_path: 0 missing: 0.2
N: 60 a_trend: 0 a_level: 2 b_path: 0 missing: 0.5
Coverage through the Change in Trend
Autoregressive Effect
OLS
NW
FGLS
AR(1)

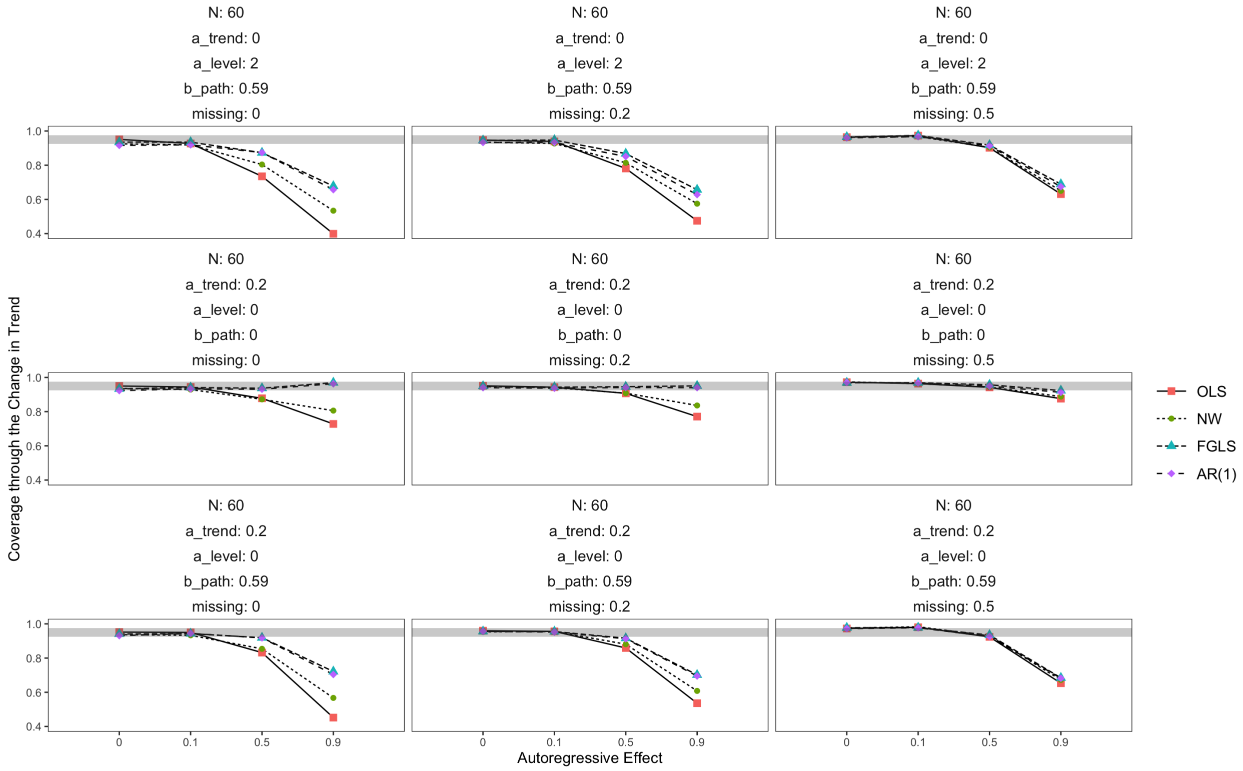
N: 60 a_trend: 0 a_level: 2 b_path: 0.59 missing: 0
N: 60 a_trend: 0 a_level: 2 b_path: 0.59 missing: 0.2
N: 60 a_trend: 0 a_level: 2 b_path: 0.59 missing: 0.5
N: 60 a_trend: 0.2 a_level: 0 b_path: 0 missing: 0
N: 60 a_trend: 0.2 a_level: 0 b_path: 0 missing: 0.2
N: 60 a_trend: 0.2 a_level: 0 b_path: 0 missing: 0.5
N: 60 a_trend: 0.2 a_level: 0 b_path: 0.59 missing: 0
N: 60 a_trend: 0.2 a_level: 0 b_path: 0.59 missing: 0.2
N: 60 a_trend: 0.2 a_level: 0 b_path: 0.59 missing: 0.5
Coverage through the Change in Trend
Autoregressive Effect
OLS
NW
FGLS
AR(1)

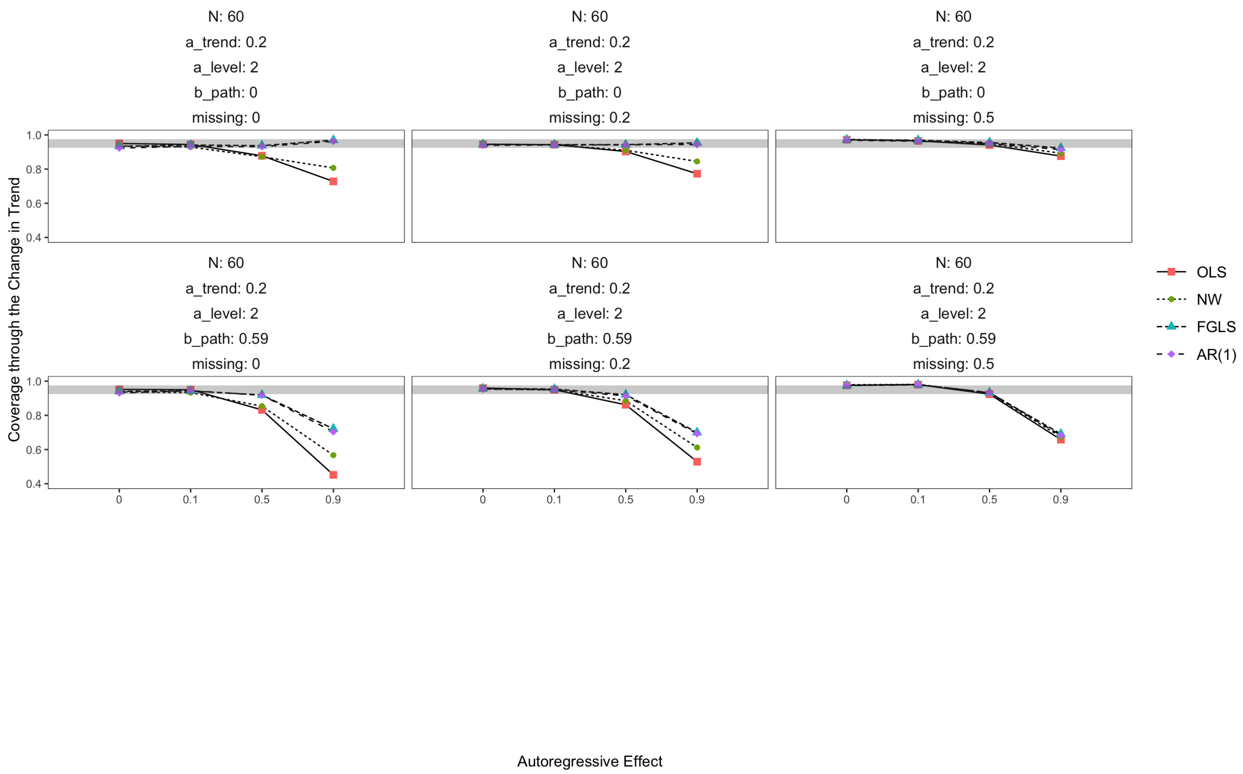
N: 60
a_trend: 0.2
a_level: 2
b_path: 0
missing: 0
missing: 0.2
missing: 0.5
b_path: 0.59
Coverage through the Change in Trend
1.0
0.8
0.6
0.4
0
0.1
0.5
0.9
OLS
NW
FGLS
AR(1)
Autoregressive Effect

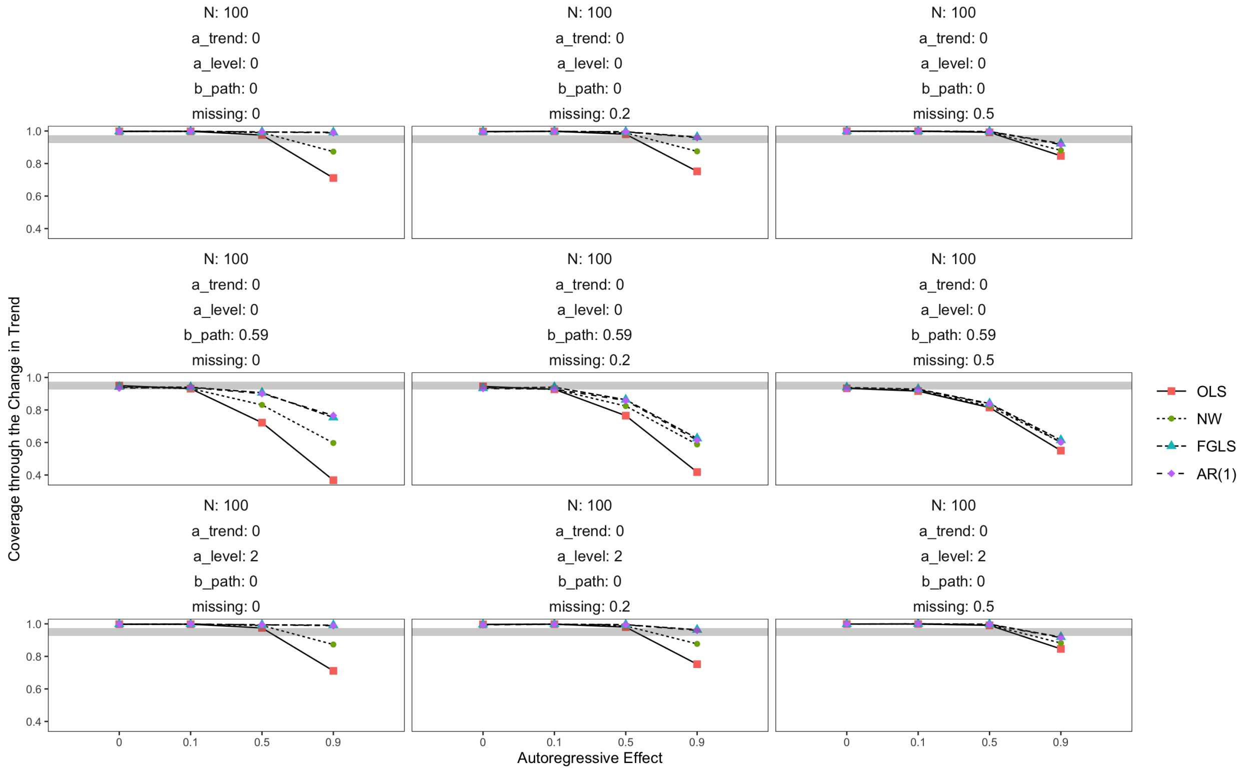
N: 100
a_trend: 0
a_level: 0
a_level: 2
b_path: 0
b_path: 0.59
missing: 0
missing: 0.2
missing: 0.5
Coverage through the Change in Trend
1.0
0.8
0.6
0.4
0
0.1
0.5
0.9
OLS
NW
FGLS
AR(1)
Autoregressive Effect

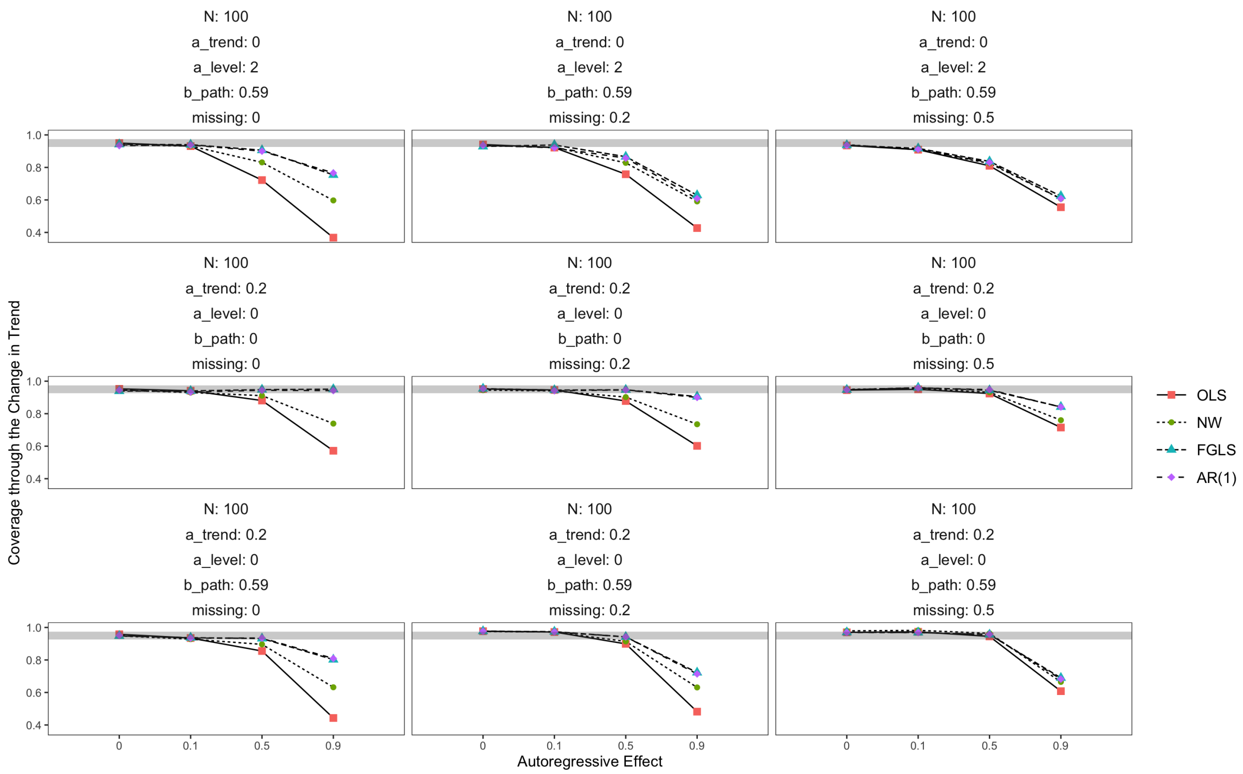

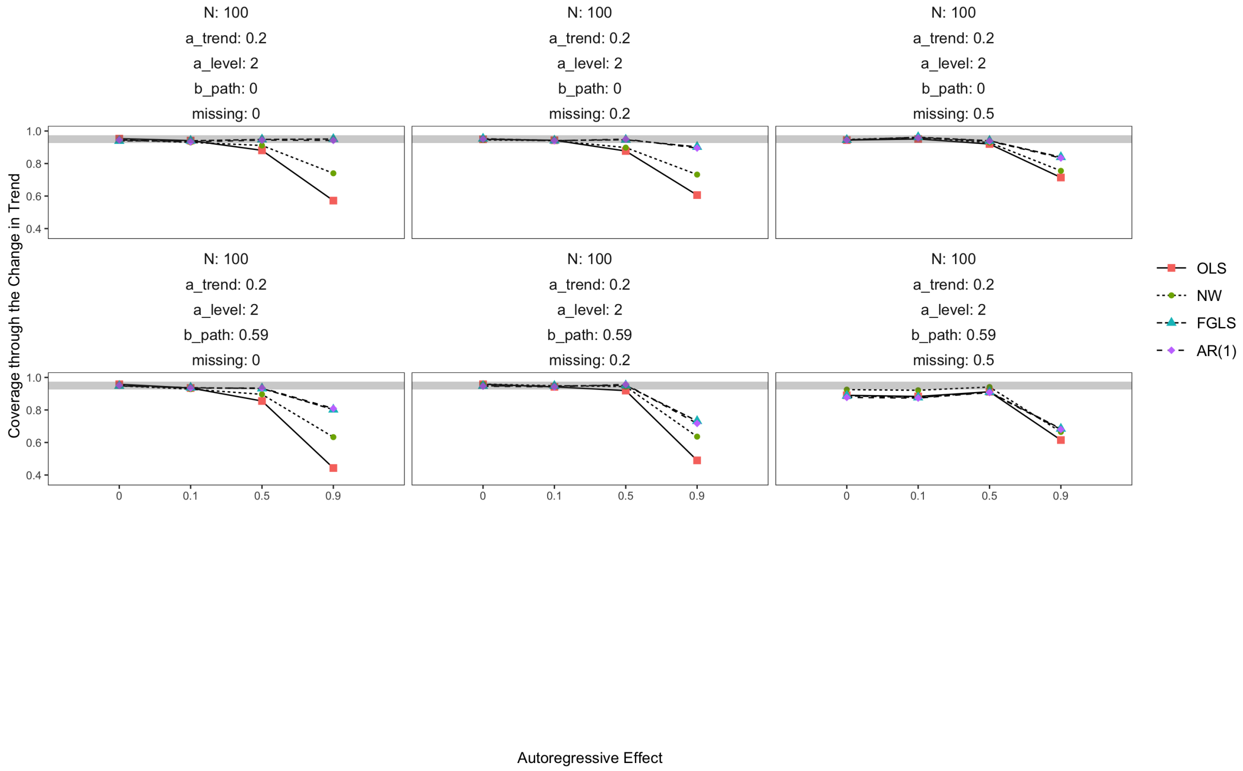

Interval Width of the Indirect Effect through the Change in Level

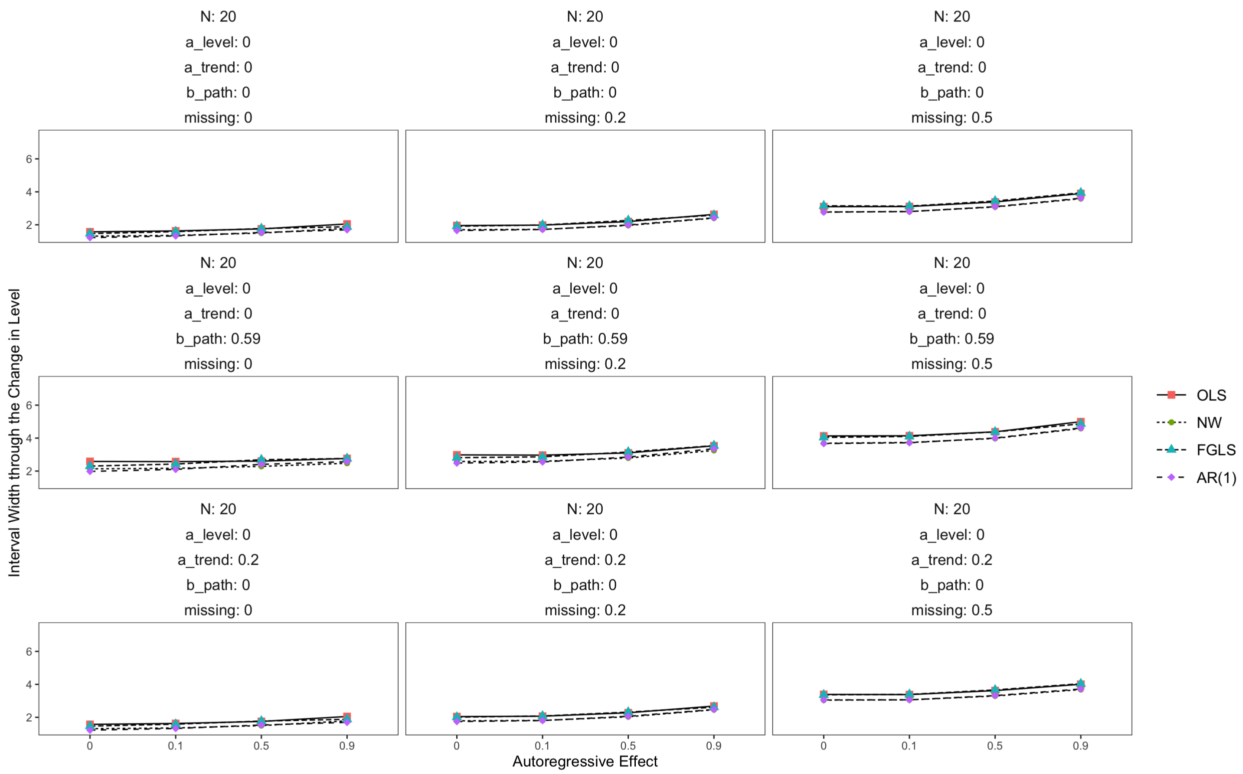
N: 20
a_level: 0
a_trend: 0
b_path: 0
missing: 0
N: 20
a_level: 0
a_trend: 0
b_path: 0
missing: 0.2
N: 20
a_level: 0
a_trend: 0
b_path: 0
missing: 0.5
N: 20
a_level: 0
a_trend: 0
b_path: 0.59
missing: 0
N: 20
a_level: 0
a_trend: 0
b_path: 0.59
missing: 0.2
N: 20
a_level: 0
a_trend: 0
b_path: 0.59
missing: 0.5
N: 20
a_level: 0
a_trend: 0.2
b_path: 0
missing: 0
N: 20
a_level: 0
a_trend: 0.2
b_path: 0
missing: 0.2
N: 20
a_level: 0
a_trend: 0.2
b_path: 0
missing: 0.5
Interval Width through the Change in Level
Autoregressive Effect
0
0.1
0.5
0.9
2
4
6
OLS
NW
FGLS
AR(1)

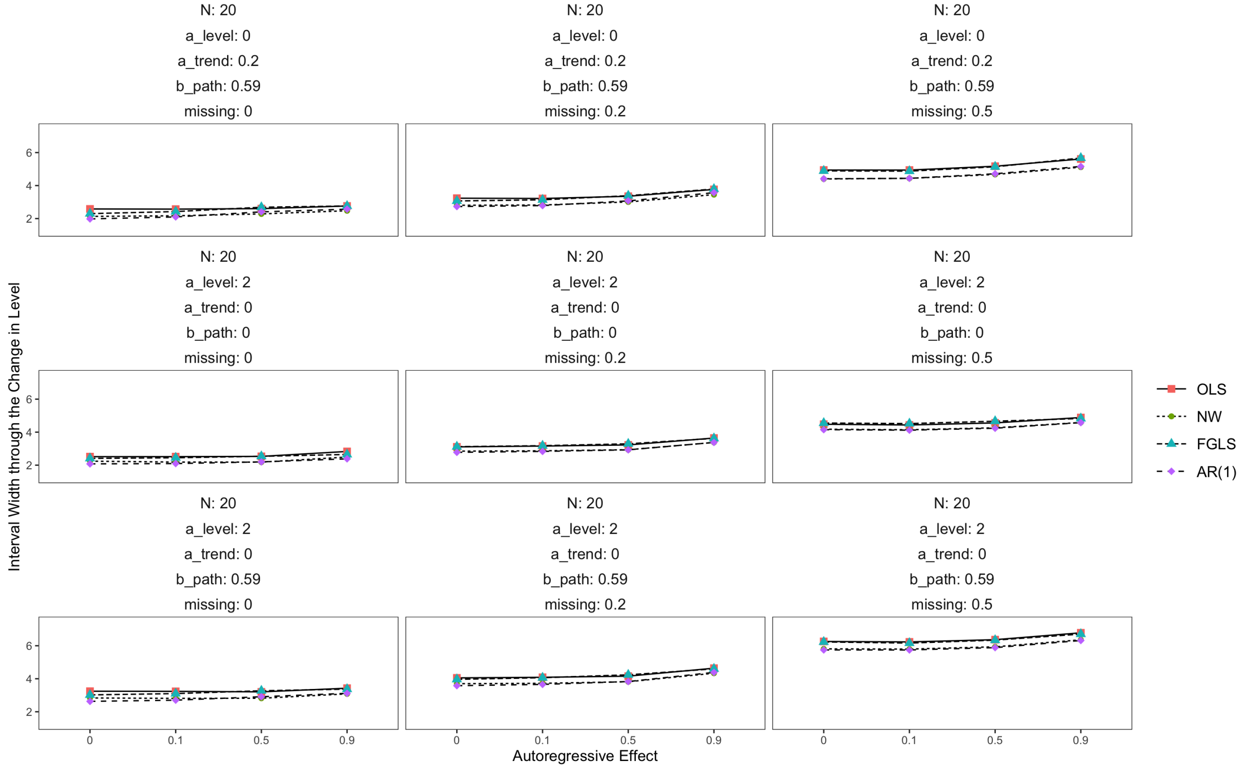
N: 20
a_level: 0
a_trend: 0.2
b_path: 0.59
missing: 0
N: 20
a_level: 0
a_trend: 0.2
b_path: 0.59
missing: 0.2
N: 20
a_level: 0
a_trend: 0.2
b_path: 0.59
missing: 0.5
N: 20
a_level: 2
a_trend: 0
b_path: 0
missing: 0
N: 20
a_level: 2
a_trend: 0
b_path: 0
missing: 0.2
N: 20
a_level: 2
a_trend: 0
b_path: 0
missing: 0.5
N: 20
a_level: 2
a_trend: 0
b_path: 0.59
missing: 0
N: 20
a_level: 2
a_trend: 0
b_path: 0.59
missing: 0.2
N: 20
a_level: 2
a_trend: 0
b_path: 0.59
missing: 0.5
Interval Width through the Change in Level
Autoregressive Effect
0
0.1
0.5
0.9
2
4
6
OLS
NW
FGLS
AR(1)

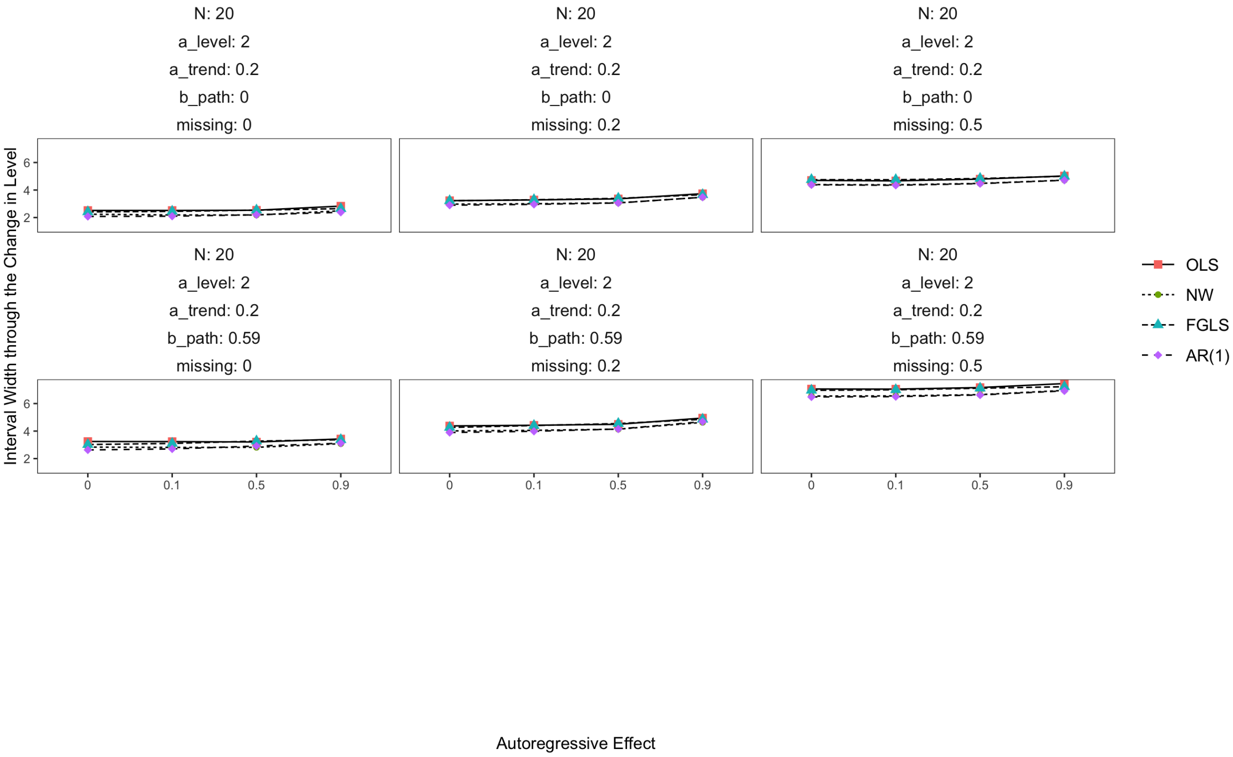
N: 20
a_level: 2
a_trend: 0.2
b_path: 0
missing: 0
missing: 0.2
missing: 0.5
b_path: 0.59
Interval Width through the Change in Level
2
4
6
0
0.1
0.5
0.9
OLS
NW
FGLS
AR(1)
Autoregressive Effect

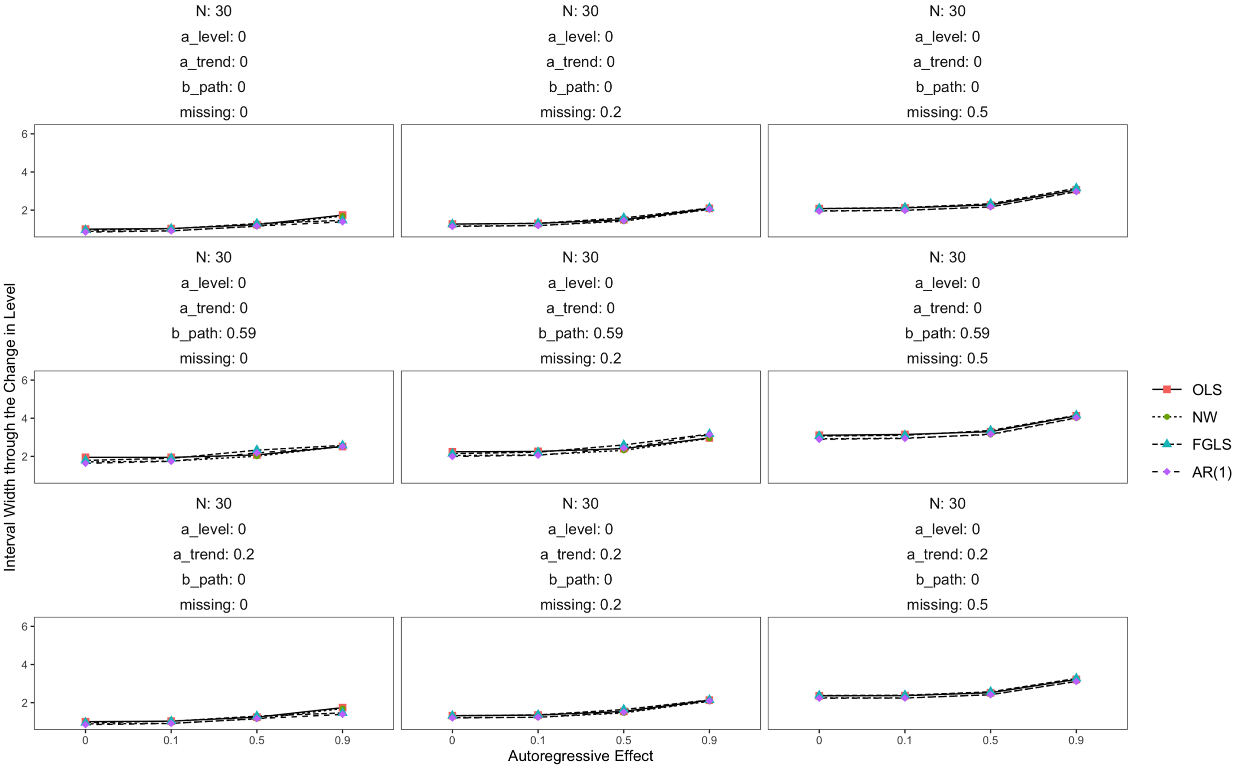
N: 30
a_level: 0
a_trend: 0
a_trend: 0.2
b_path: 0
b_path: 0.59
missing: 0
missing: 0.2
missing: 0.5
Interval Width through the Change in Level
2
4
6
0
0.1
0.5
0.9
OLS
NW
FGLS
AR(1)
Autoregressive Effect

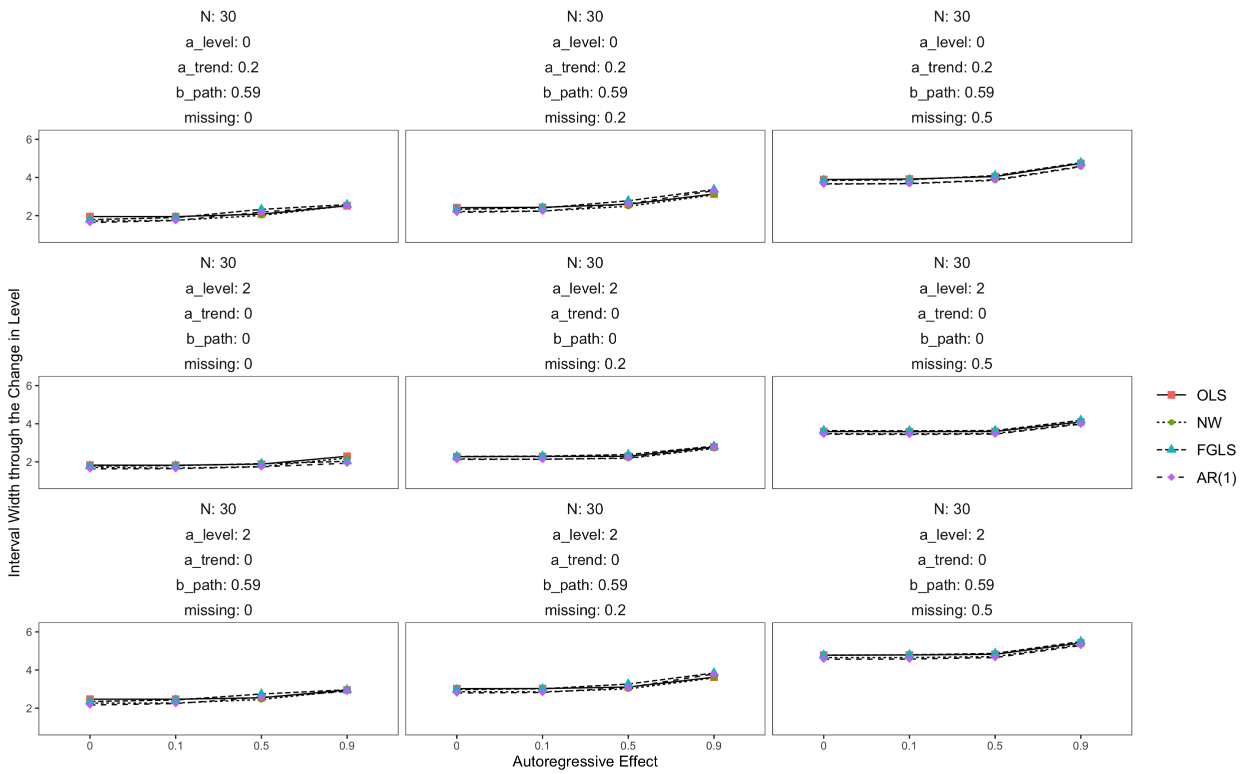
N: 30
a_level: 0
a_trend: 0.2
b_path: 0.59
missing: 0
missing: 0.2
missing: 0.5
N: 30
a_level: 2
a_trend: 0
b_path: 0
missing: 0
missing: 0.2
missing: 0.5
N: 30
a_level: 2
a_trend: 0
b_path: 0.59
missing: 0
missing: 0.2
missing: 0.5
Interval Width through the Change in Level
Autoregressive Effect
OLS
NW
FGLS
AR(1)

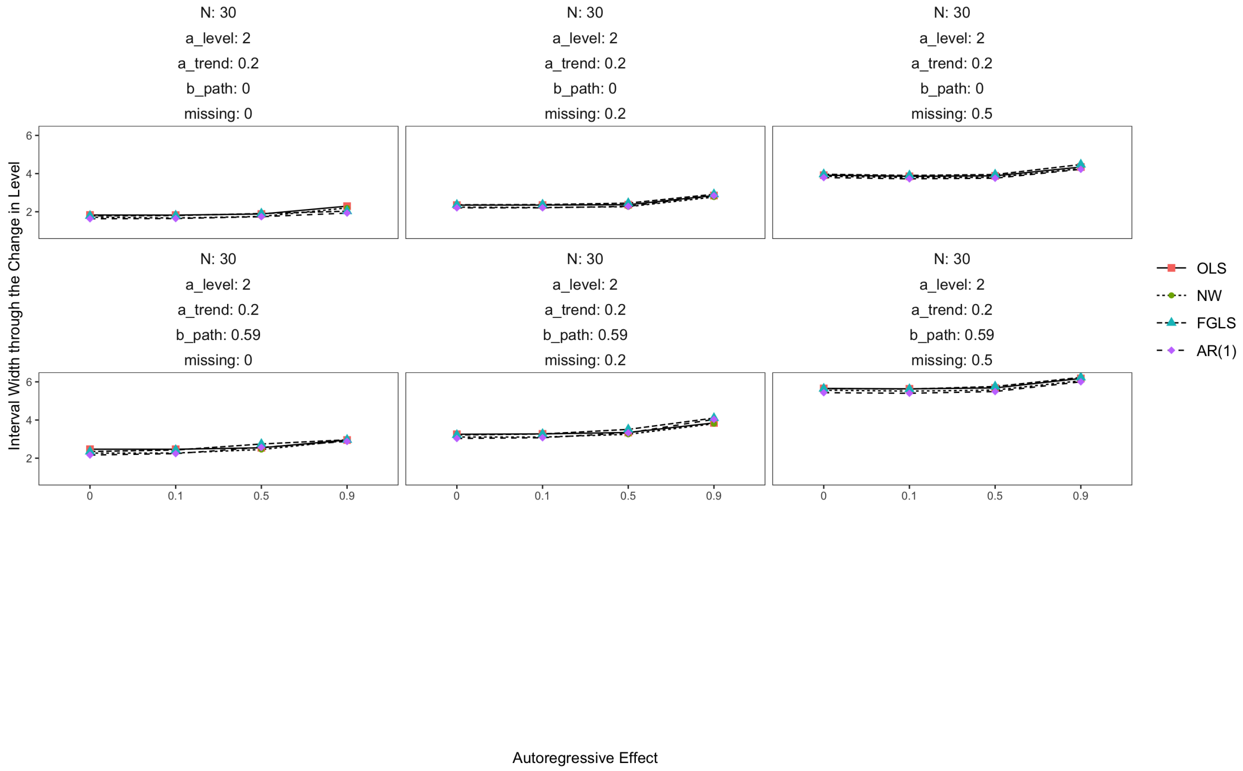
N: 30
a_level: 2
a_trend: 0.2
b_path: 0
missing: 0
missing: 0.2
missing: 0.5
N: 30
a_level: 2
a_trend: 0.2
b_path: 0.59
missing: 0
missing: 0.2
missing: 0.5
Interval Width through the Change in Level
Autoregressive Effect
OLS
NW
FGLS
AR(1)

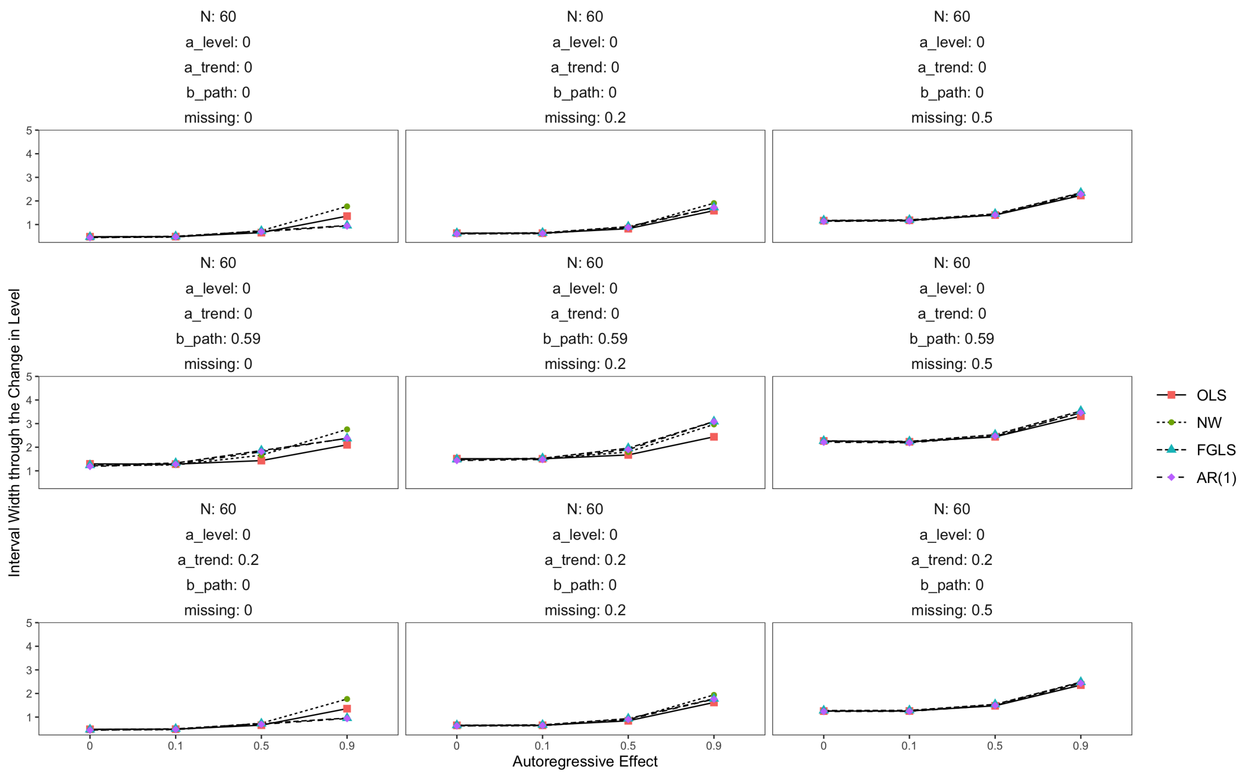
N: 60
a_level: 0
a_trend: 0
b_path: 0
missing: 0
missing: 0.2
missing: 0.5
b_path: 0.59
a_trend: 0.2
Interval Width through the Change in Level
Autoregressive Effect
0
0.1
0.5
0.9
1
2
3
4
5
OLS
NW
FGLS
AR(1)

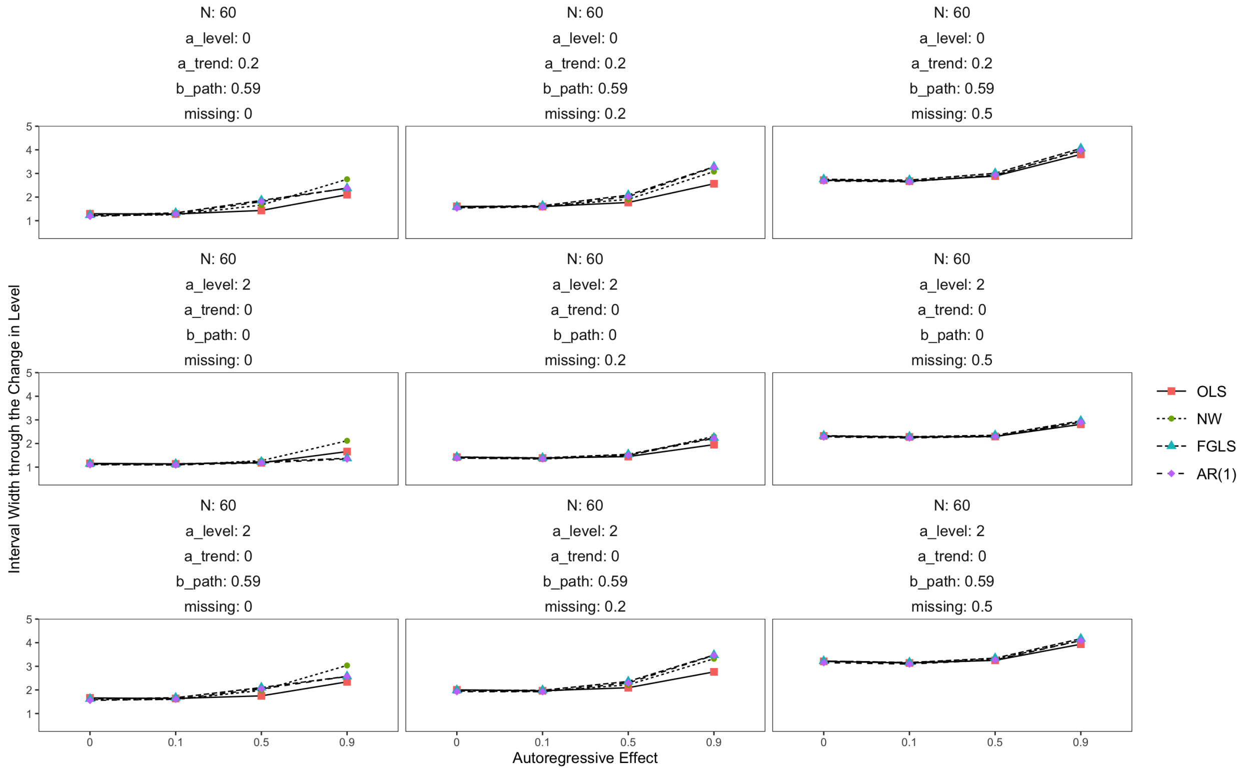
N: 60
a_level: 0
a_level: 2
a_trend: 0.2
a_trend: 0
b_path: 0
b_path: 0.59
missing: 0
missing: 0.2
missing: 0.5
Interval Width through the Change in Level
Autoregressive Effect
0
0.1
0.5
0.9
1
2
3
4
5
OLS
NW
FGLS
AR(1)

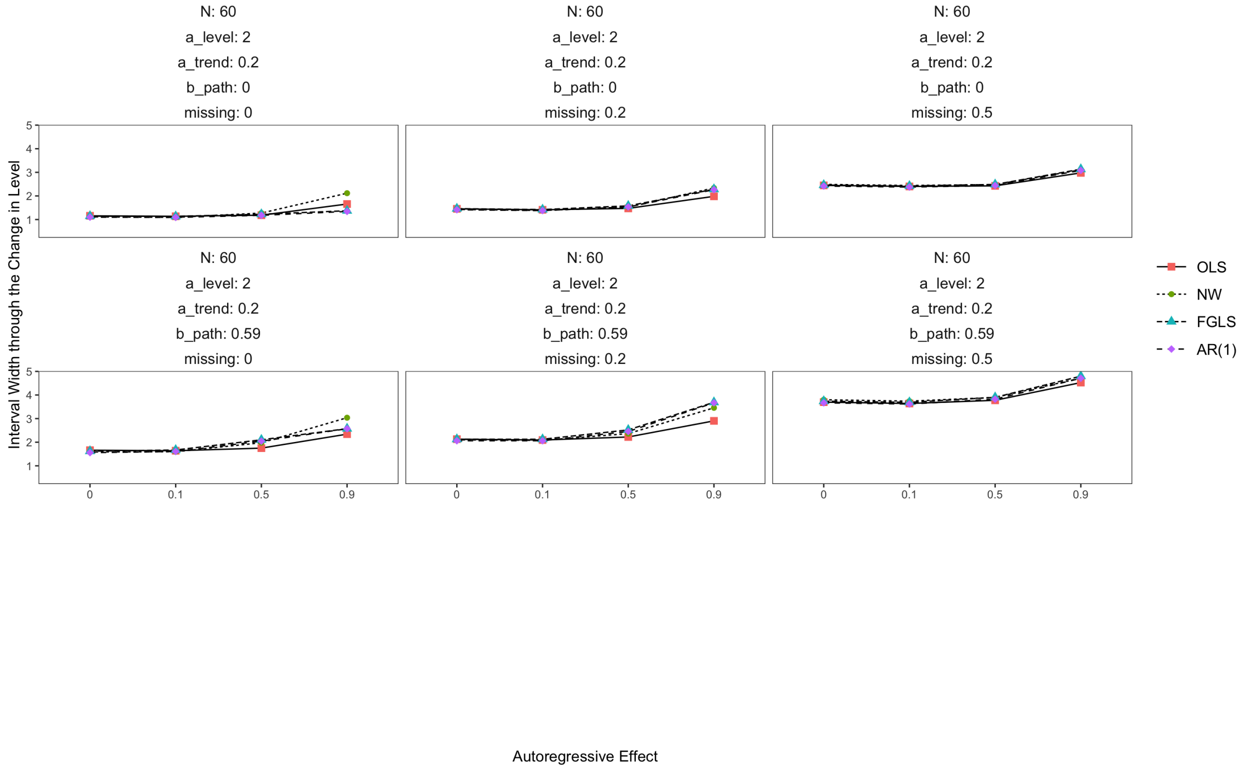
N: 60
a_level: 2
a_trend: 0.2
b_path: 0
missing: 0
N: 60
a_level: 2
a_trend: 0.2
b_path: 0
missing: 0.2
N: 60
a_level: 2
a_trend: 0.2
b_path: 0
missing: 0.5
N: 60
a_level: 2
a_trend: 0.2
b_path: 0.59
missing: 0
N: 60
a_level: 2
a_trend: 0.2
b_path: 0.59
missing: 0.2
N: 60
a_level: 2
a_trend: 0.2
b_path: 0.59
missing: 0.5
Interval Width through the Change in Level
OLS
NW
FGLS
AR(1)
0
0.1
0.5
0.9
Autoregressive Effect

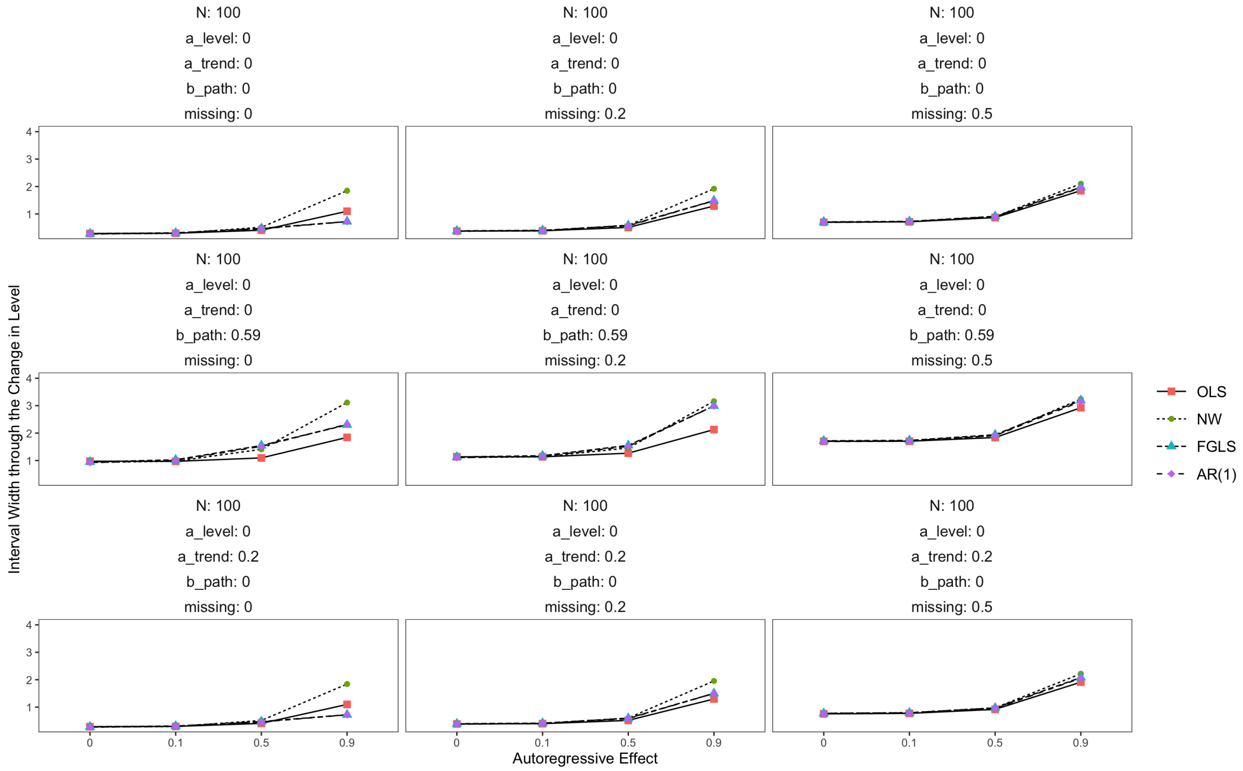
N: 100
a_level: 0
a_trend: 0
b_path: 0
missing: 0
N: 100
a_level: 0
a_trend: 0
b_path: 0
missing: 0.2
N: 100
a_level: 0
a_trend: 0
b_path: 0
missing: 0.5
N: 100
a_level: 0
a_trend: 0
b_path: 0.59
missing: 0
N: 100
a_level: 0
a_trend: 0
b_path: 0.59
missing: 0.2
N: 100
a_level: 0
a_trend: 0
b_path: 0.59
missing: 0.5
N: 100
a_level: 0
a_trend: 0.2
b_path: 0
missing: 0
N: 100
a_level: 0
a_trend: 0.2
b_path: 0
missing: 0.2
N: 100
a_level: 0
a_trend: 0.2
b_path: 0
missing: 0.5
Interval Width through the Change in Level
OLS
NW
FGLS
AR(1)
0
0.1
0.5
0.9
Autoregressive Effect

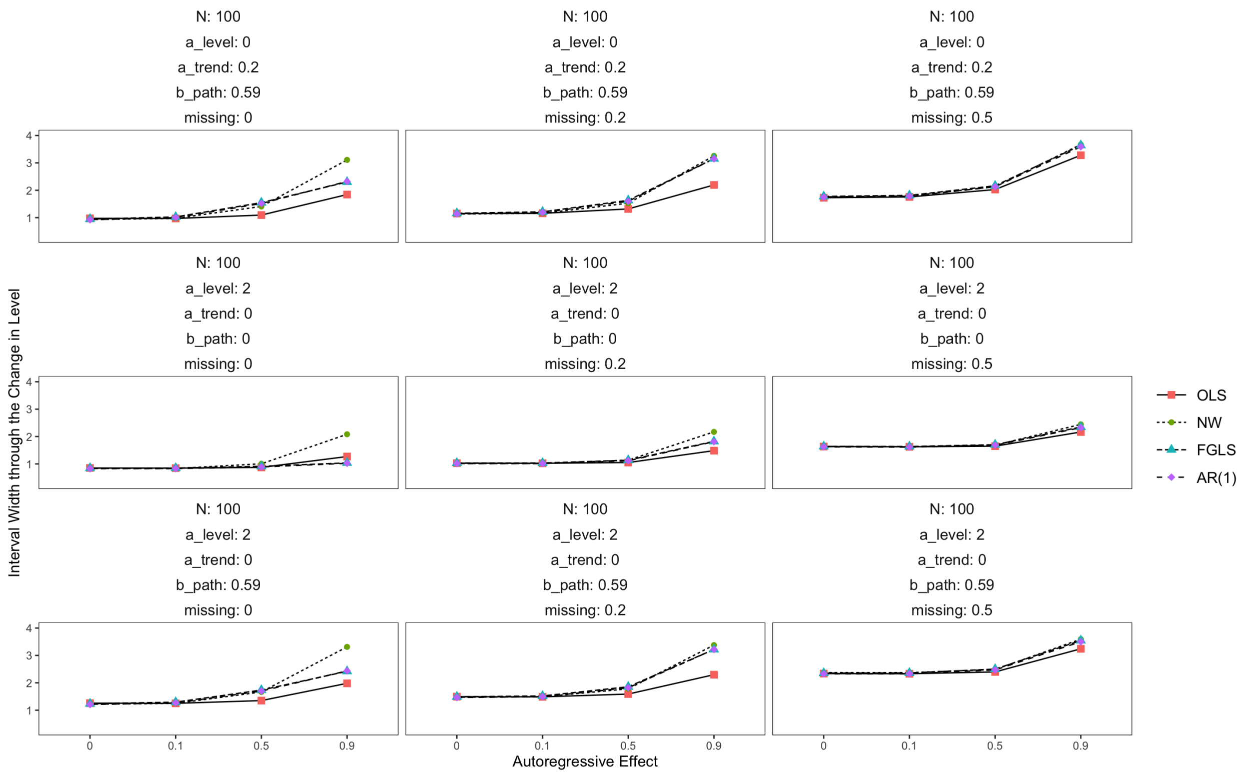

N: 100
a_level: 0
a_trend: 0.2
b_path: 0.59
missing: 0
N: 100
a_level: 0
a_trend: 0.2
b_path: 0.59
missing: 0.2
N: 100
a_level: 0
a_trend: 0.2
b_path: 0.59
missing: 0.5
N: 100
a_level: 2
a_trend: 0
b_path: 0
missing: 0
N: 100
a_level: 2
a_trend: 0
b_path: 0
missing: 0.2
N: 100
a_level: 2
a_trend: 0
b_path: 0
missing: 0.5
N: 100
a_level: 2
a_trend: 0
b_path: 0.59
missing: 0
N: 100
a_level: 2
a_trend: 0
b_path: 0.59
missing: 0.2
N: 100
a_level: 2
a_trend: 0
b_path: 0.59
missing: 0.5
Interval Width through the Change in Level
Autoregressive Effect
0
0.1
0.5
0.9
1
2
3
4
OLS
NW
FGLS
AR(1)

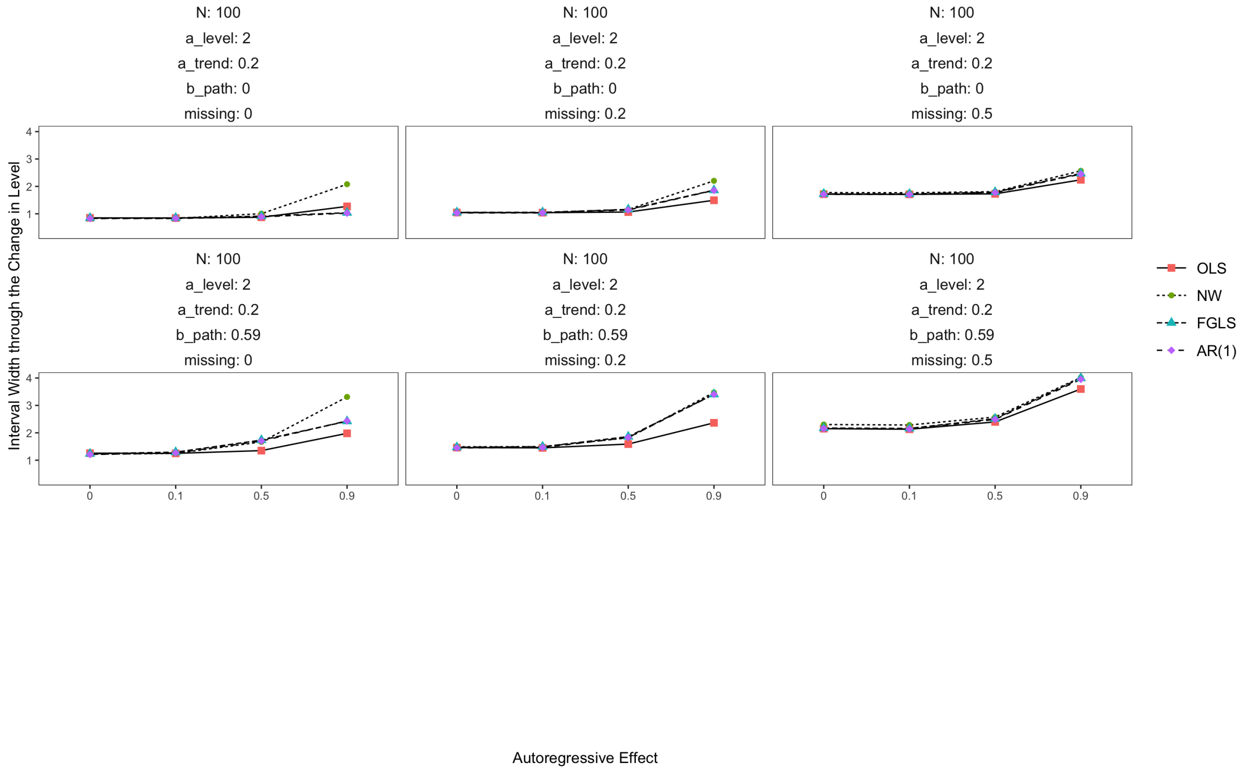

N: 100
a_level: 2
a_trend: 0.2
b_path: 0
missing: 0
N: 100
a_level: 2
a_trend: 0.2
b_path: 0
missing: 0.2
N: 100
a_level: 2
a_trend: 0.2
b_path: 0
missing: 0.5
N: 100
a_level: 2
a_trend: 0.2
b_path: 0.59
missing: 0
N: 100
a_level: 2
a_trend: 0.2
b_path: 0.59
missing: 0.2
N: 100
a_level: 2
a_trend: 0.2
b_path: 0.59
missing: 0.5
Interval Width through the Change in Level
Autoregressive Effect
0
0.1
0.5
0.9
1
2
3
4
OLS
NW
FGLS
AR(1)

Interval Width of the Indirect Effect through the Change in Trend

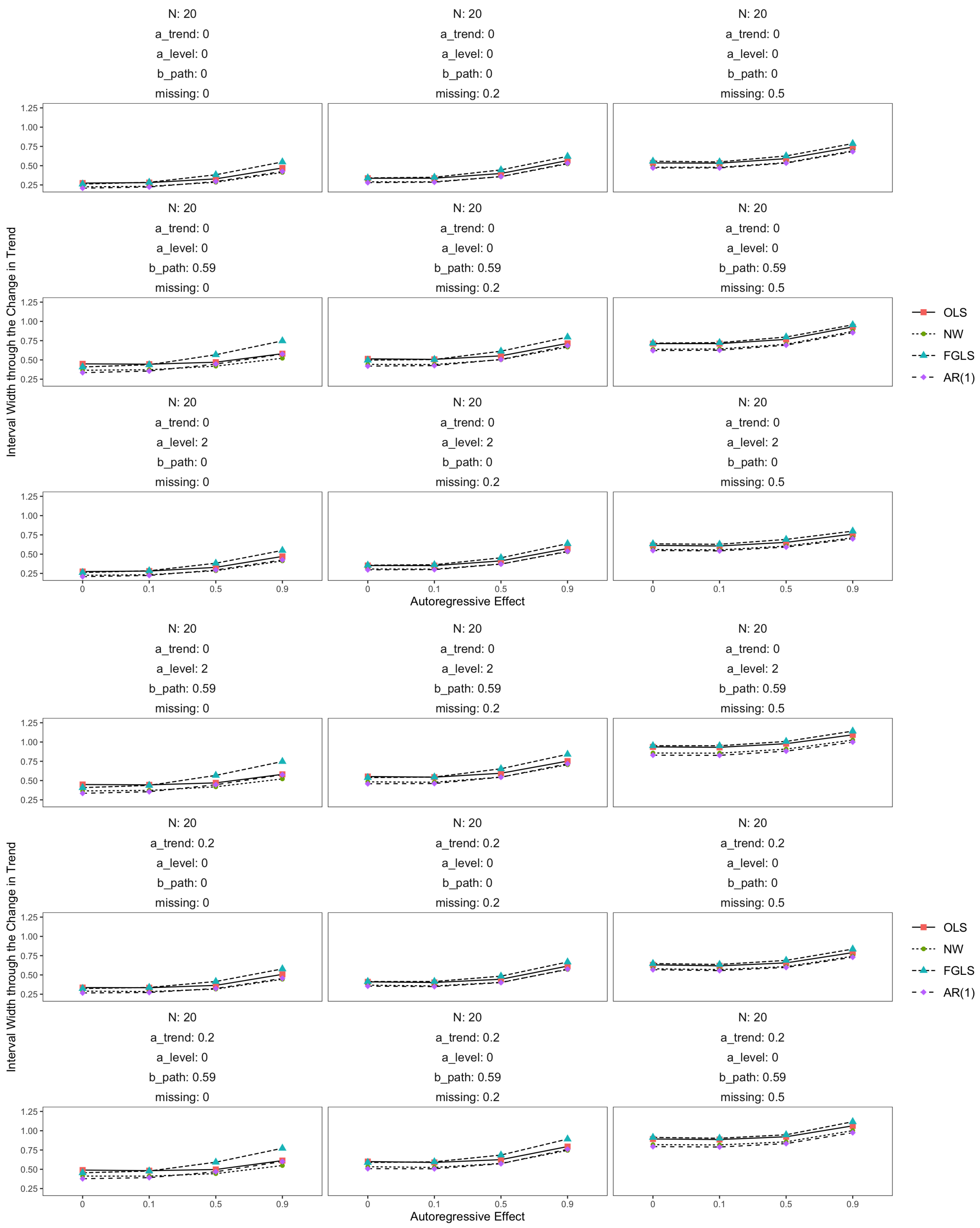

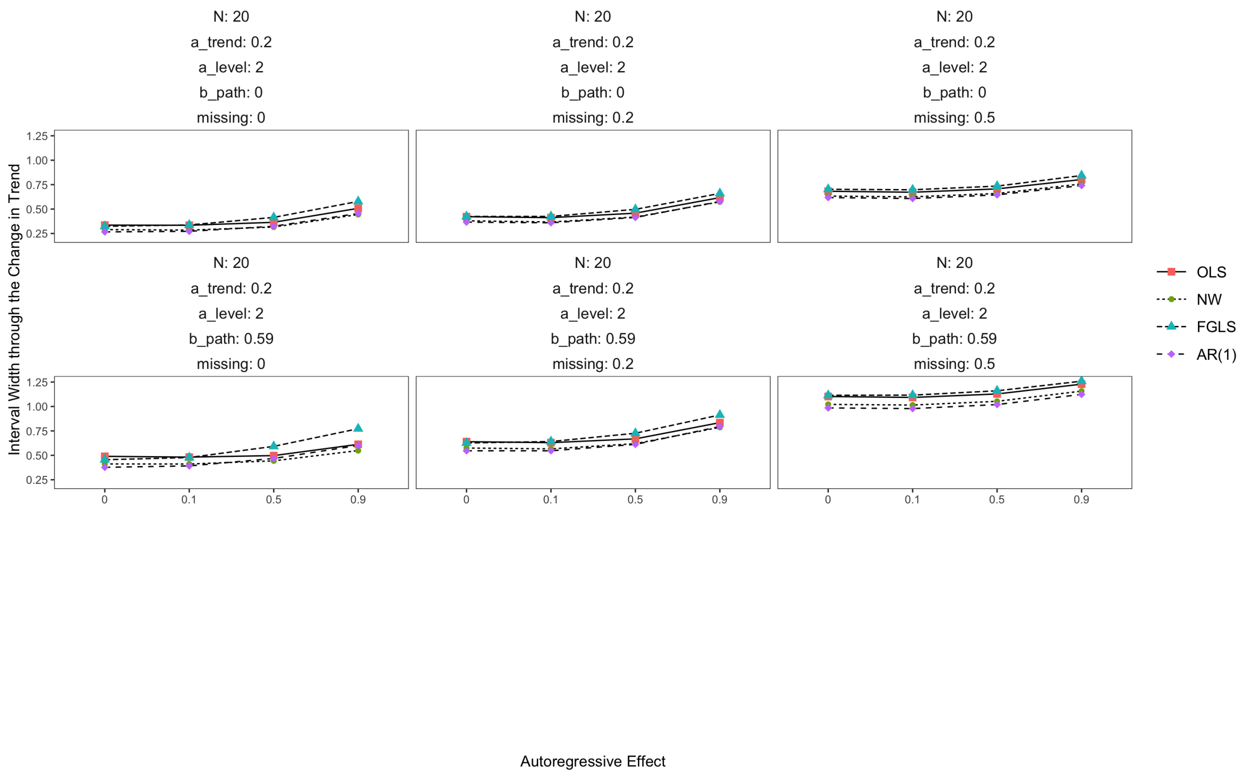
N: 20
a_trend: 0.2
a_level: 2
b_path: 0
missing: 0
N: 20
a_trend: 0.2
a_level: 2
b_path: 0
missing: 0.2
N: 20
a_trend: 0.2
a_level: 2
b_path: 0
missing: 0.5
N: 20
a_trend: 0.2
a_level: 2
b_path: 0.59
missing: 0
N: 20
a_trend: 0.2
a_level: 2
b_path: 0.59
missing: 0.2
N: 20
a_trend: 0.2
a_level: 2
b_path: 0.59
missing: 0.5
Interval Width through the Change in Trend
1.25
1.00
0.75
0.50
0.25
0
0.1
0.5
0.9
OLS
NW
FGLS
AR(1)
Autoregressive Effect

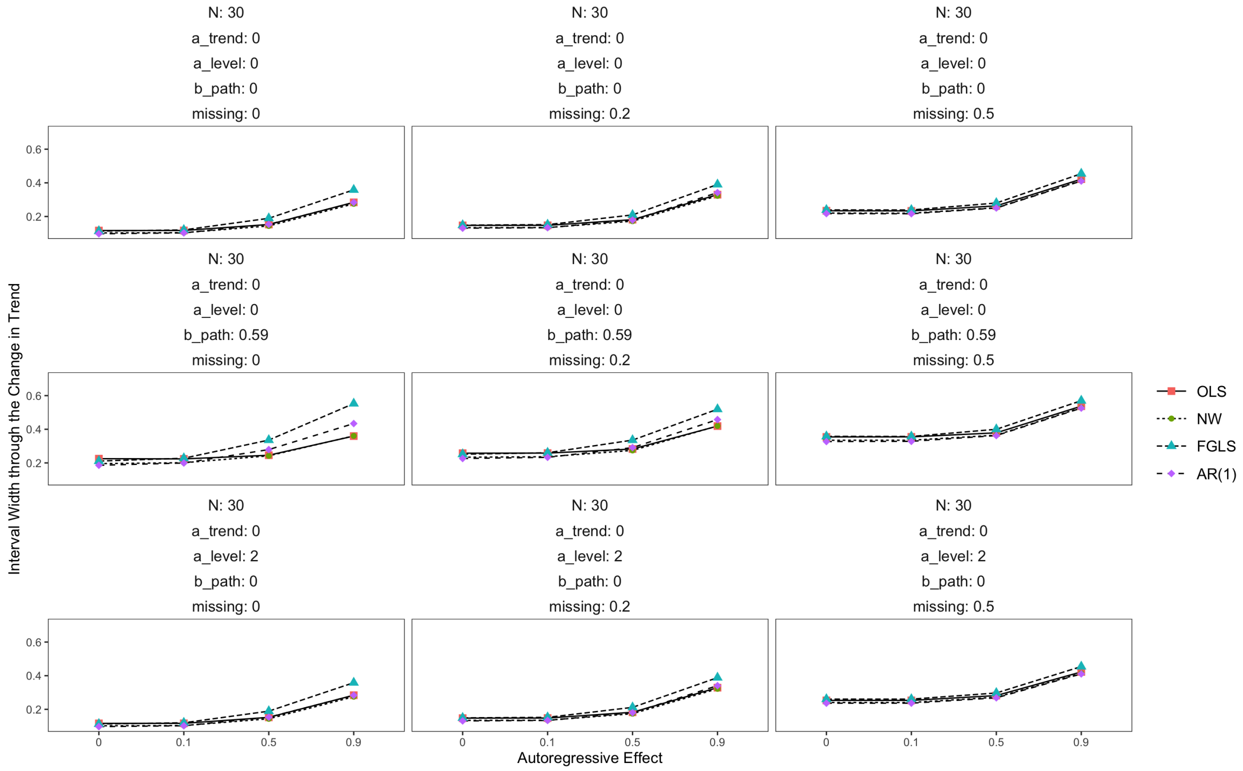
N: 30
a_trend: 0
a_level: 0
b_path: 0
missing: 0
N: 30
a_trend: 0
a_level: 0
b_path: 0
missing: 0.2
N: 30
a_trend: 0
a_level: 0
b_path: 0
missing: 0.5
N: 30
a_trend: 0
a_level: 0
b_path: 0.59
missing: 0
N: 30
a_trend: 0
a_level: 0
b_path: 0.59
missing: 0.2
N: 30
a_trend: 0
a_level: 0
b_path: 0.59
missing: 0.5
N: 30
a_trend: 0
a_level: 2
b_path: 0
missing: 0
N: 30
a_trend: 0
a_level: 2
b_path: 0
missing: 0.2
N: 30
a_trend: 0
a_level: 2
b_path: 0
missing: 0.5
Interval Width through the Change in Trend
0.6
0.4
0.2
0
0.1
0.5
0.9
OLS
NW
FGLS
AR(1)
Autoregressive Effect

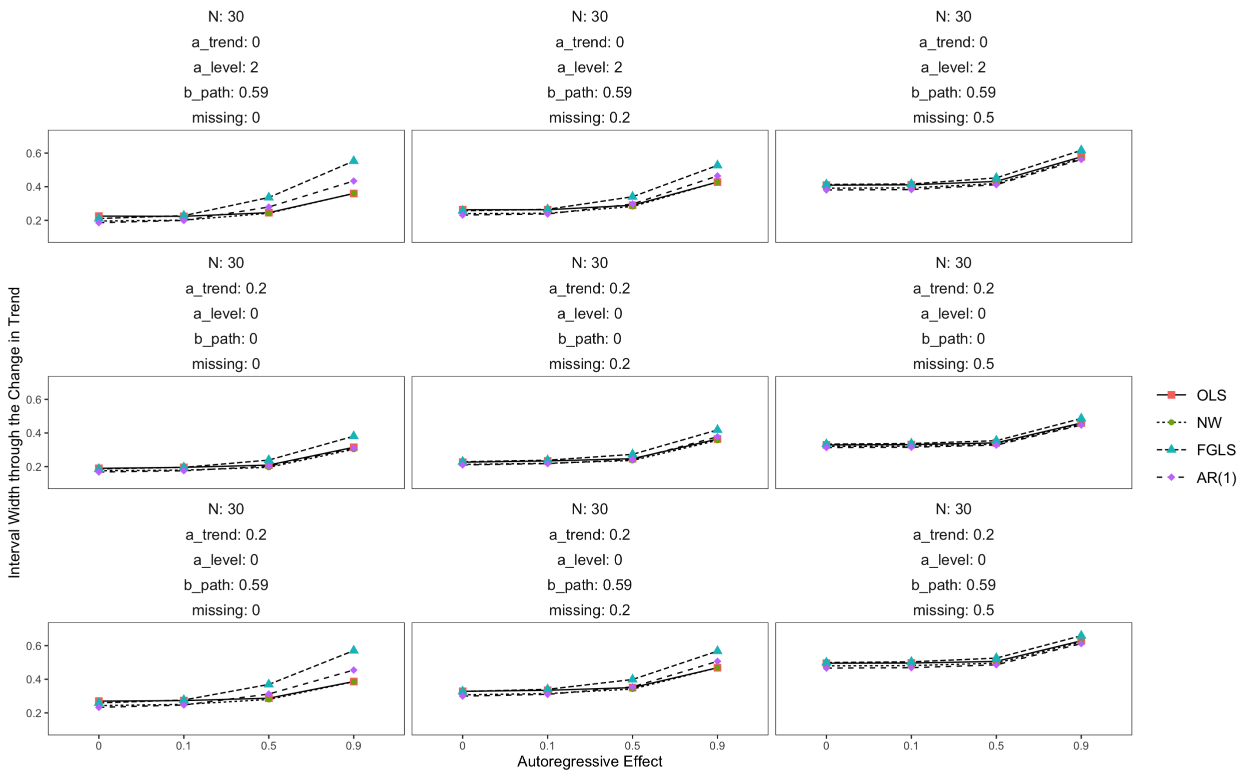
N: 30
a_trend: 0
a_level: 2
b_path: 0.59
missing: 0
N: 30
a_trend: 0
a_level: 2
b_path: 0.59
missing: 0.2
N: 30
a_trend: 0
a_level: 2
b_path: 0.59
missing: 0.5
N: 30
a_trend: 0.2
a_level: 0
b_path: 0
missing: 0
N: 30
a_trend: 0.2
a_level: 0
b_path: 0
missing: 0.2
N: 30
a_trend: 0.2
a_level: 0
b_path: 0
missing: 0.5
N: 30
a_trend: 0.2
a_level: 0
b_path: 0.59
missing: 0
N: 30
a_trend: 0.2
a_level: 0
b_path: 0.59
missing: 0.2
N: 30
a_trend: 0.2
a_level: 0
b_path: 0.59
missing: 0.5
Interval Width through the Change in Trend
0.2
0.4
0.6
0
0.1
0.5
0.9
Autoregressive Effect
OLS
NW
FGLS
AR(1)

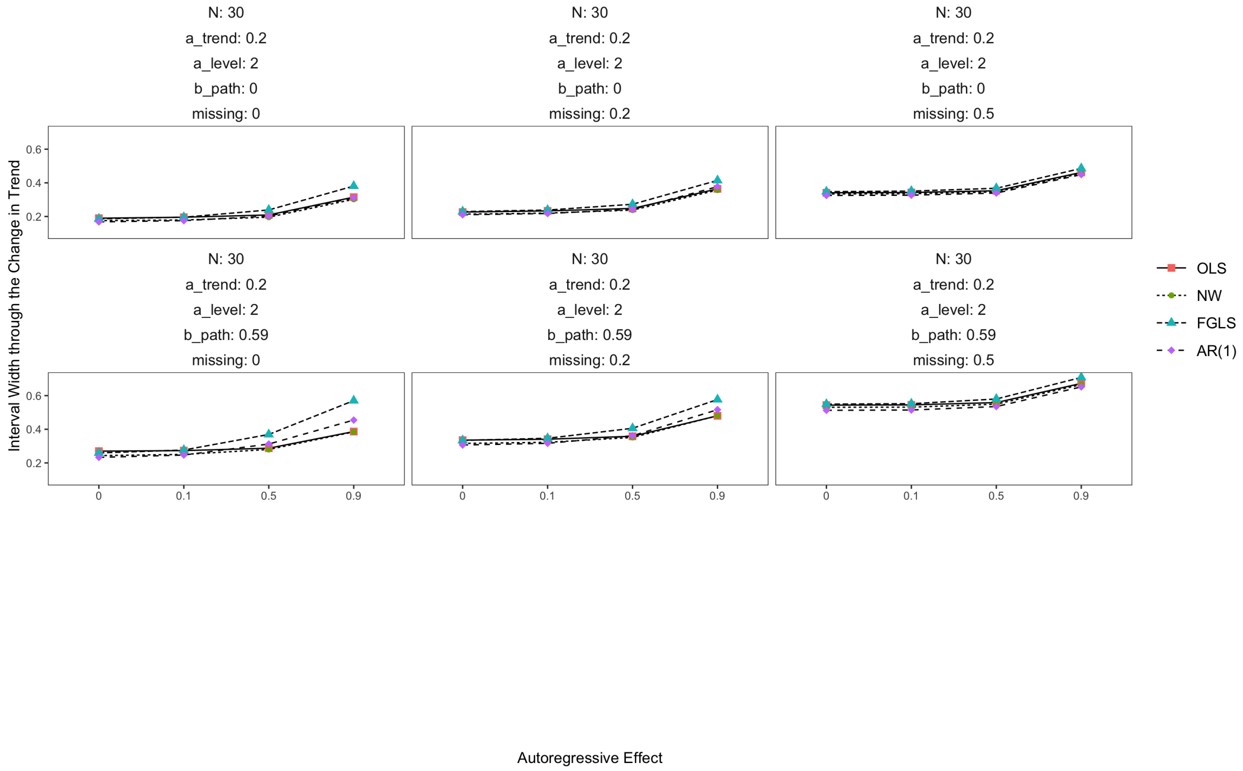
N: 30
a_trend: 0.2
a_level: 2
b_path: 0
missing: 0
N: 30
a_trend: 0.2
a_level: 2
b_path: 0
missing: 0.2
N: 30
a_trend: 0.2
a_level: 2
b_path: 0
missing: 0.5
N: 30
a_trend: 0.2
a_level: 2
b_path: 0.59
missing: 0
N: 30
a_trend: 0.2
a_level: 2
b_path: 0.59
missing: 0.2
N: 30
a_trend: 0.2
a_level: 2
b_path: 0.59
missing: 0.5
Interval Width through the Change in Trend
0.2
0.4
0.6
0
0.1
0.5
0.9
Autoregressive Effect
OLS
NW
FGLS
AR(1)

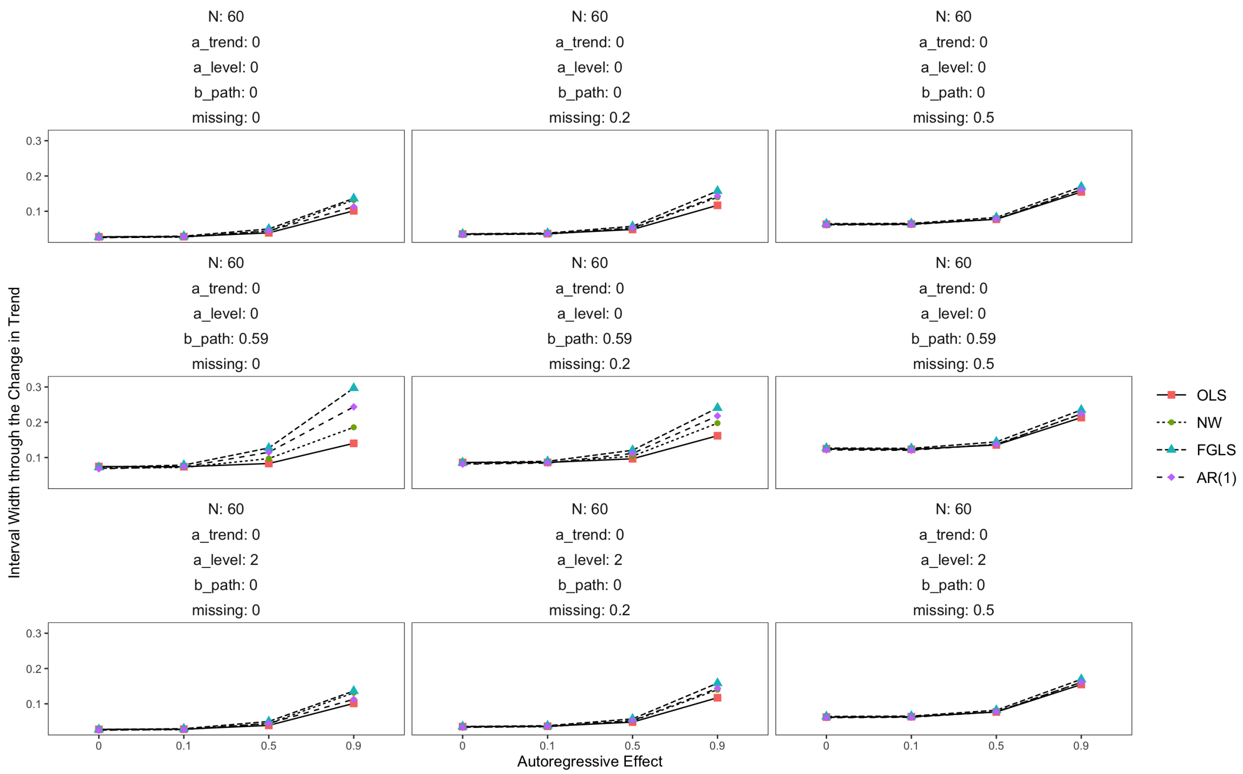

N: 60
a_trend: 0
a_level: 0
b_path: 0
missing: 0
N: 60
a_trend: 0
a_level: 0
b_path: 0
missing: 0.2
N: 60
a_trend: 0
a_level: 0
b_path: 0
missing: 0.5
N: 60
a_trend: 0
a_level: 0
b_path: 0.59
missing: 0
N: 60
a_trend: 0
a_level: 0
b_path: 0.59
missing: 0.2
N: 60
a_trend: 0
a_level: 0
b_path: 0.59
missing: 0.5
N: 60
a_trend: 0
a_level: 2
b_path: 0
missing: 0
N: 60
a_trend: 0
a_level: 2
b_path: 0
missing: 0.2
N: 60
a_trend: 0
a_level: 2
b_path: 0
missing: 0.5
Interval Width through the Change in Trend
0.1
0.2
0.3
0
0.1
0.5
0.9
Autoregressive Effect
OLS
NW
FGLS
AR(1)

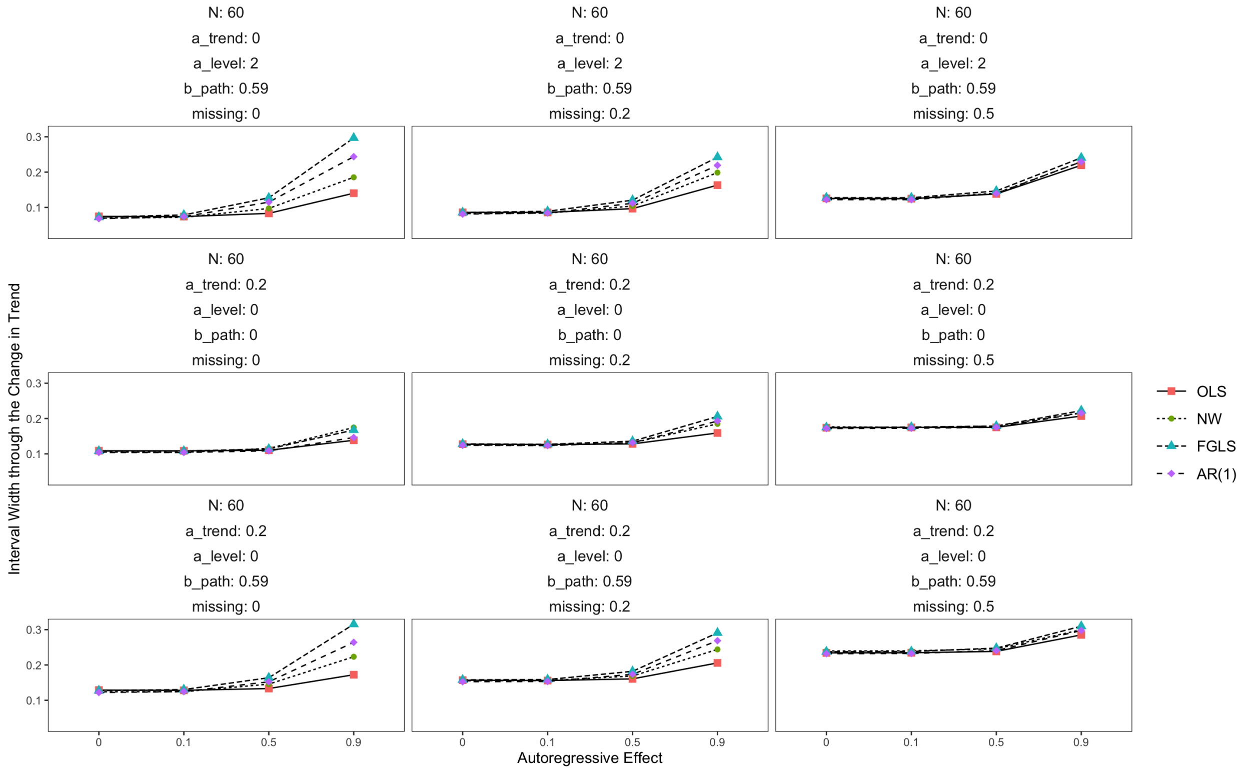

N: 60
a_trend: 0
a_level: 2
b_path: 0.59
missing: 0
N: 60
a_trend: 0
a_level: 2
b_path: 0.59
missing: 0.2
N: 60
a_trend: 0
a_level: 2
b_path: 0.59
missing: 0.5
N: 60
a_trend: 0.2
a_level: 0
b_path: 0
missing: 0
N: 60
a_trend: 0.2
a_level: 0
b_path: 0
missing: 0.2
N: 60
a_trend: 0.2
a_level: 0
b_path: 0
missing: 0.5
N: 60
a_trend: 0.2
a_level: 0
b_path: 0.59
missing: 0
N: 60
a_trend: 0.2
a_level: 0
b_path: 0.59
missing: 0.2
N: 60
a_trend: 0.2
a_level: 0
b_path: 0.59
missing: 0.5
Interval Width through the Change in Trend
0.1
0.2
0.3
0
0.1
0.5
0.9
Autoregressive Effect
OLS
NW
FGLS
AR(1)

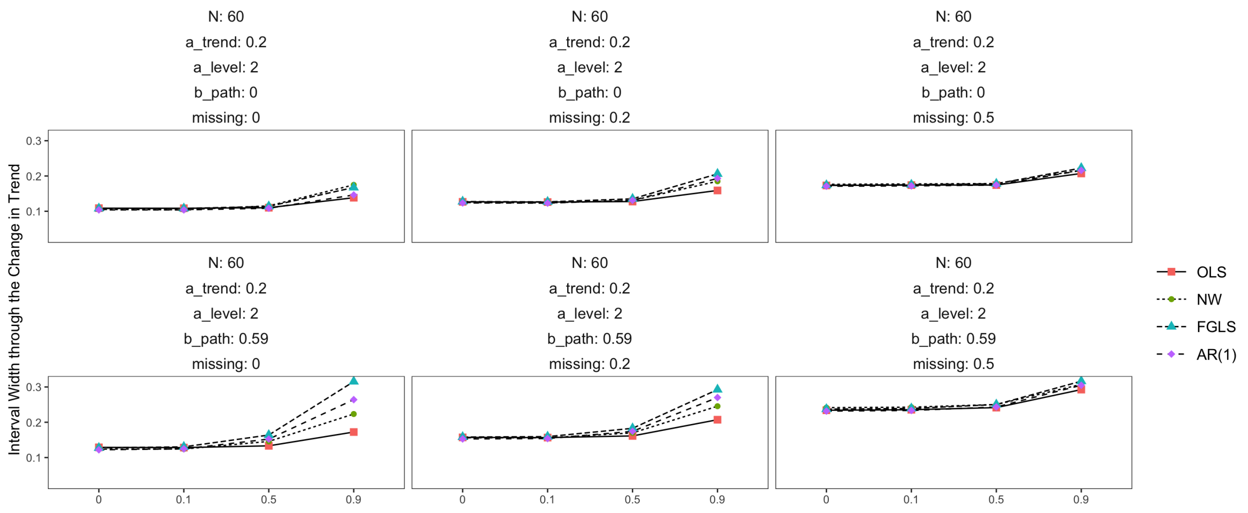

Autoregressive Effect

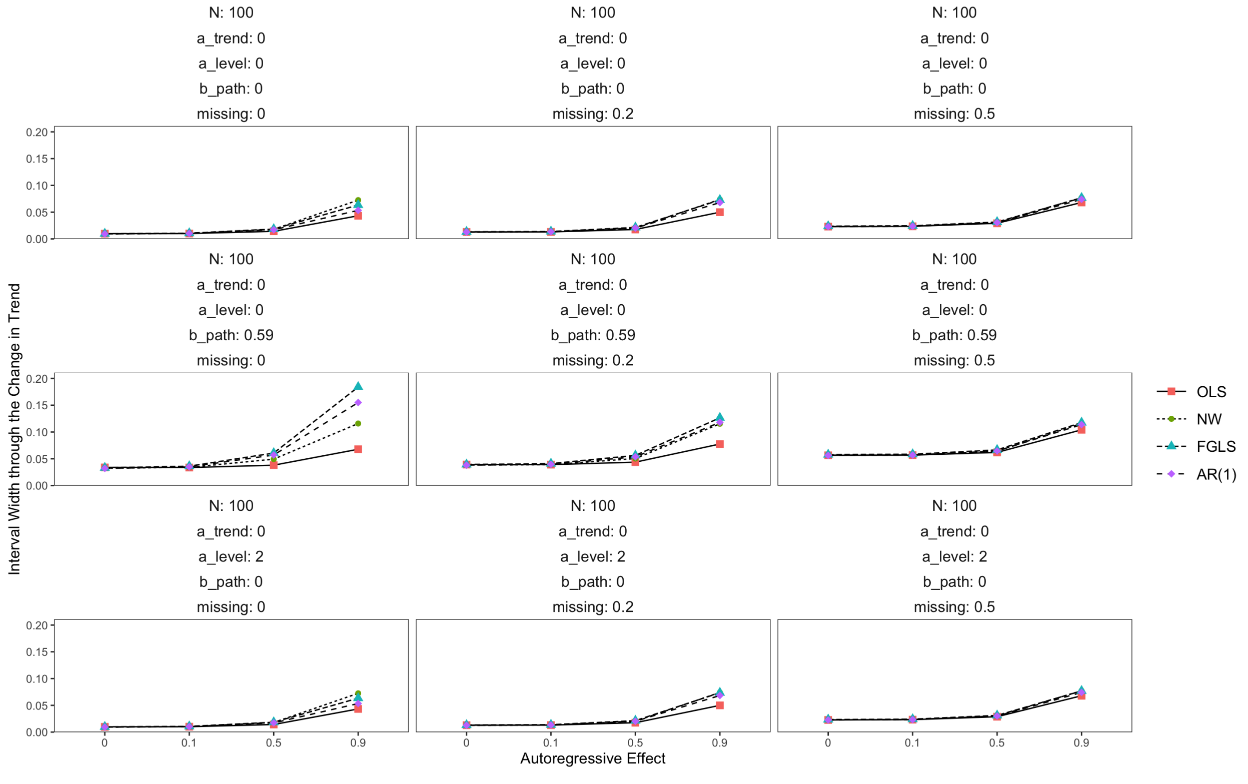

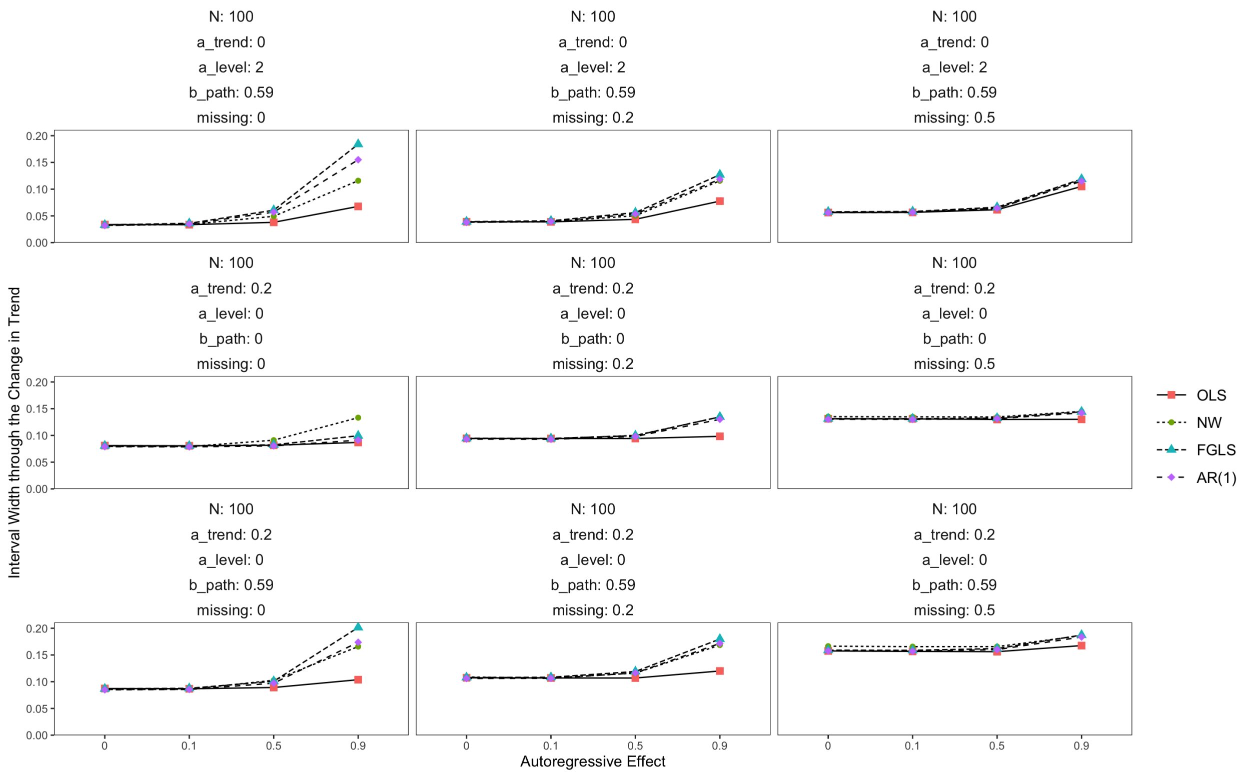
N: 100
a_trend: 0
a_level: 2
b_path: 0.59
missing: 0
missing: 0.2
missing: 0.5
a_trend: 0.2
a_level: 0
b_path: 0
Interval Width through the Change in Trend
Autoregressive Effect
OLS
NW
FGLS
AR(1)

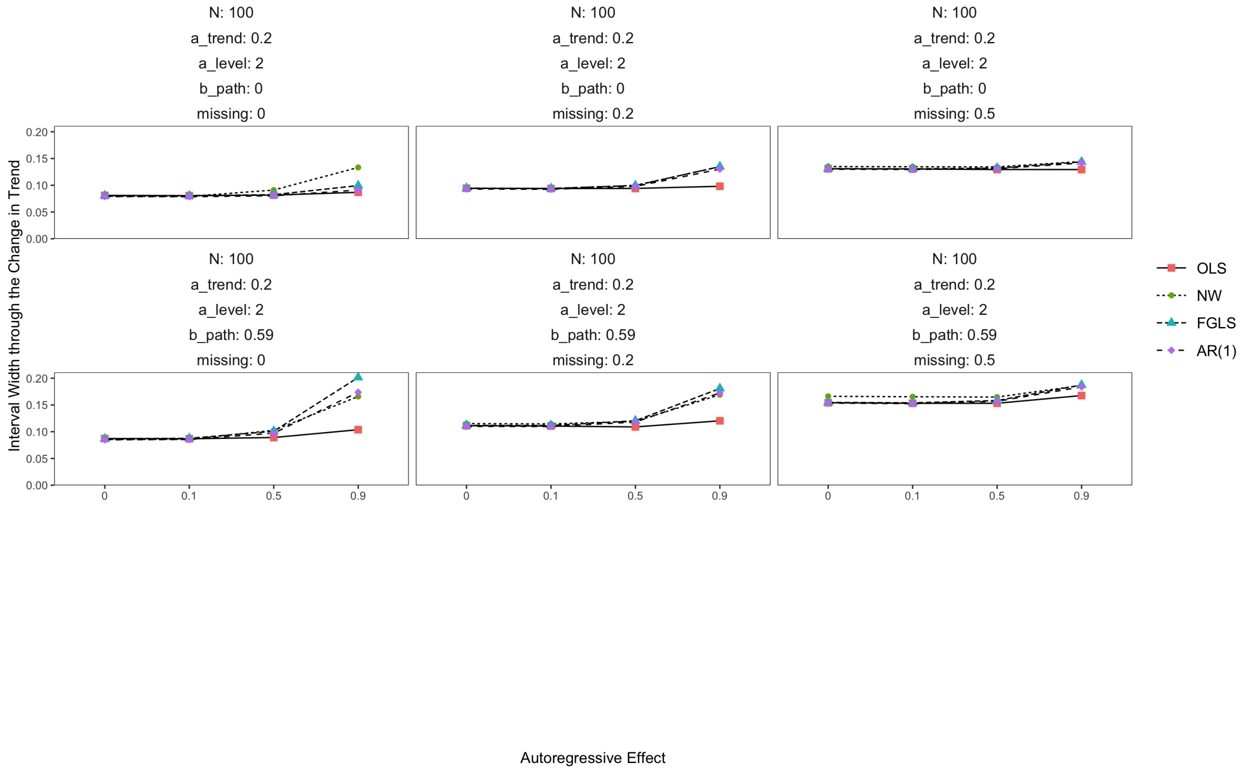
N: 100
a_trend: 0.2
a_level: 2
b_path: 0
b_path: 0.59
missing: 0
missing: 0.2
missing: 0.5
Interval Width through the Change in Trend
Autoregressive Effect
OLS
NW
FGLS
AR(1)
